# Synthetic Epoxyeicosatrienoic Acid Mimics Protect Mesangial Cells from Sorafenib-Induced Cell Death

**DOI:** 10.3390/molecules30071445

**Published:** 2025-03-24

**Authors:** Marcus de Bourg, Abhishek Mishra, Rawand S. Mohammad, Christophe Morisseau, Bruce D. Hammock, John D. Imig, Anders Vik

**Affiliations:** 1 Department of Pharmacy, Section for Pharmaceutical Chemistry, University of Oslo, N-0316 Oslo, Norway; 2 Department of Pharmaceutical Sciences, College of Pharmacy, University of Arkansas for Medical Sciences, Little Rock, AR 72205, USA; amishra@uams.edu (A.M.); jimig@uams.edu (J.D.I.); 3 Department of Entomology and Nematology and UC Davis Comprehensive Cancer Center, University of California Davis, Davis, CA 95616, USA; chmorisseau@ucdavis.edu (C.M.);

**Keywords:** epoxy fatty acids, eicosatrienoic acids, human renal mesangial cells, sorafenib, mimics

## Abstract

Nineteen potential mimics of 8,9-epoxyeicosatrienoic acid (8,9-EET), a natural bioactive oxylipin, were synthesized and evaluated for their ability to protect renal mesangial cells against sorafenib-induced cell death in a water-soluble tetrazolium (WST-8) assay. All compounds were also evaluated as inhibitors of soluble epoxide hydrolase. As expected of a potent pan-kinase inhibitor the drug sorafenib caused a significant decrease in cell viability in HRMCs. Several analogs containing amide and oxamide groups in place of the epoxide showed efficacy in reducing sorafenib induced human renal mesangial cell (HRMC) death. Oxamide containing analogs proved particularly effective, with the most promising analog increasing cell viability five-fold over control at 1 µM. These analogs, containing an oxamide group as a bioisostere for the epoxide in 8,9-EET, did not display significant inhibitory activity towards soluble epoxide hydrolase. This preliminary structure–activity relationship analysis reveals the oxamide group as a promising bioisostere for the epoxide in the 8,9-position of the fatty acid chain, producing protective effects against sorafenib-induced cell death in HRMCs. Collectively, these findings demonstrate the potential for using epoxide mimics and particularly oxamides as 8,9-EET analogs as bioisosteres of the corresponding epoxide in a therapeutic strategy against sorafenib-induced glomerular nephrotoxicity.

## 1. Introduction

Epoxyeicosatrienoic acids (EETs), metabolites derived from arachidonic acid through the action of cytochrome P450 (CYP) oxidase enzymes [1], play vital roles in cardiovascular and renal actions, particularly by influencing blood pressure regulation, sodium excretion, and inflammation control [2,3,4]. EETs exert vasodilatory effects that reduce renal vascular resistance, promoting proper kidney blood flow and alleviating hypertension [2,5,6]. A growing body of evidence indicates that lower plasma levels of EETs, frequently due to increased soluble epoxide hydrolase (sEH) activity, are linked with conditions such as hypertension, coronary artery disease, and endothelial dysfunction [5,6]. In disorders like renovascular hypertension and coronary artery disease, reduced EET levels may exacerbate disease progression, including hypertensive and diabetic nephropathy. Furthermore, polymorphisms in genes coding for enzymes involved in EET regulation, including CYP2C8, CYP2C9, CYP2J2, and EPHX2, are linked with altered risks of cardiovascular diseases [7]. Human studies suggest that inhibiting sEH could potentially protect against kidney injury and slow the progression of kidney diseases [8]. EETs play a protective role in preventing renal injury by inhibiting inflammatory pathways and reducing oxidative stress in kidney cells [9]. Targeting sEH, to increase EETs, for therapeutic intervention, appears promising, particularly for treating conditions such as diabetic nephropathy and drug-induced nephrotoxicity [8,10]. In addition, EETs have also demonstrated, via the use of sEH inhibitors [11], the ability to improve renal allograft dysfunction and cardiovascular alterations in kidney transplant recipients in vivo [12]. These findings indicate that strategies aimed at maintaining optimal EET levels by inhibiting sEH activity could offer therapeutic potential in treating cardiovascular and renal conditions [13]. However, usage of pharmaceutical inhibitors come with constraints.

**Figure 1:**
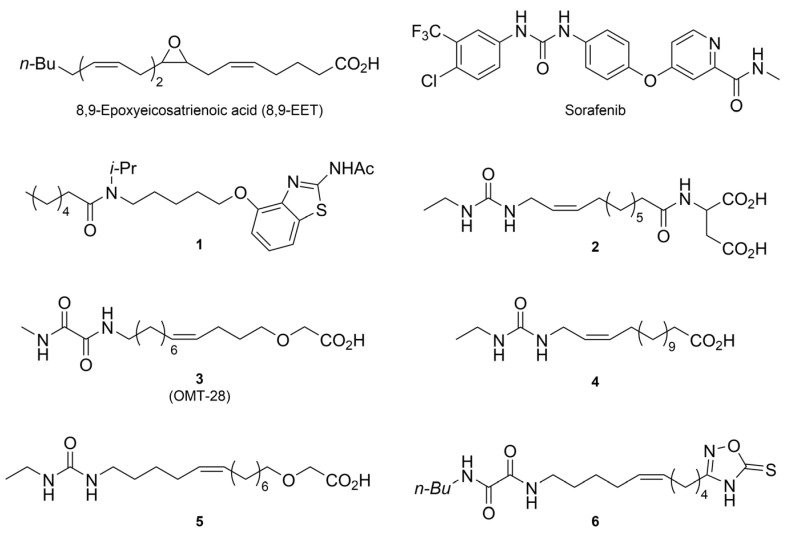
Structure of the endogenous 8,9-EET, sorafenib, and examples of some previously reported 14,15-EET and 17,18-EEQ mimics [14,24,30,31,32].

To overcome the limitations of handling inhibitors and metabolism by sEH of naturally occurring EETs, usage of analogs (also called mimics herein) to these oxylipins offer several advantages such as reduced susceptibility to enzymatic degradation [14], avoiding possible toxic metabolites of epoxy fatty acids (EpFAs) [15,16] and allowing for therapeutic effect where EpFA production is low, such as in kidney tissue [17]. Additionally, EpFA mimics can be utilized as probes to study the biological effects of EpFAs and possibly identify their putative target receptor(s) [18,19]. Research has shown that such analogs exhibit potent vasodilatory effects and help lower blood pressure. For instance, in hypertensive animal models, analogs like **1** and **2** (Figure 1) significantly reduced blood pressure in spontaneously hypertensive rats (SHRs) and angiotensin-II-induced hypertension models [20]. This development highlights the therapeutic potential of EET analogs in managing hypertension by preserving their beneficial vascular effects [21,22]. Structure–activity relationship activity studies into the cardioprotective effects and in vitro pharmacokinetic studies of 17,18-epoxy-5,8,11,14-eicosatetraenoic acid (17,18-EEQ) have led to the development of the 17,18-EEQ mimic OMT-28 (**3**) [23]. This mimic has shown a favorable toxicological and kinetic profile in phase I clinical trials and is currently in phase II clinical trials for patients with persistent atrial fibrillation [24].

Sorafenib (Figure 1) is a multi-kinase inhibitor with applications across challenging oncology cases, including hepatocellular carcinoma (HCC), renal cell carcinoma (RCC), and desmoid tumors. It works by inhibiting multiple signaling pathways that are essential for tumor growth and survival, primarily the Ras/MEK/ERK and PI3K/Akt/mTOR pathways. Additionally, sorafenib’s inhibition of VEGFR (vascular endothelial growth factor receptor) offers an anti-angiogenic effect, which is particularly beneficial for solid tumors [25]. However, this inhibition also leads to significant nephrotoxicity, resulting in glomerular injury and proteinuria, which pose major clinical challenges [26]. Inhibitors of VEGF and tyrosine kinases (TKIs), like sorafenib, causes a decrease in glomerular nephrin levels, and subsequent proteinurea [27]. In addition, renal inflammation is a contributing factor to the observed nephrotoxic effects of inhibitors of VEGF/TKI, causing several undesirable effects, such as acute tubular necrosis [28]. In this study, we aimed to investigate whether synthetic 8,9-EET analogs could help mitigate the nephrotoxic effects of sorafenib on glomerular mesangial cells, which are known to be sensitive to the presence of external 8,9-EET (Figure 1) in the medium [29].

## 2. Results and Discussion

### 2.1. Mimic Design

Previous studies have shown that EpFA mimics with fewer double bond than the endogenous EpFA are capable of retaining the activity (**3**, Figure 1 [31]). Placement of a double bond in a different position, however, can lead to antagonist-like activity (**4** has an antagonistic effect, while **5** has agonist activity) [31,33]. In a structure–activity relationship study of 8,9-EET, where the effect of removing double bonds was studied, it was found that two double bonds were required to exert nephroprotective effect [34]. Omission of double bonds provide enhanced stability against metabolic transformations such as those by cytochrome P450 enzymes leading to allylic hydroxylation and epoxidation or those catalyzed by cyclooxygenases, which can lead to undesirable biologically active metabolites [35]. Bisallylic positions are also highly prone to autoxidation and should, thus, be avoided, if possible. In order to ameliorate stability against sEH, the epoxide group has successfully been substituted with more metabolically robust isosteres, such as amides (**1**), ureas (**2**, **4**, **5**), and oxamides (**3**, **6**) while maintaining or even improving activity [14,31,33,36]. A plethora of isosteres for the carboxylate group in 14,15-EET have been explored (such as **6**) [14]. Some examples of favorable substitutions turned out to be a tetrazole and alpha-oxo carboxylate (for example **5**), which exhibited excellent activity, better metabolic stability and in the latter case better water solubility.

The focus of previous attempts at mimicking EpFAs have been targeted with 14,15-EET [14], 17,18-EEQ [30], as well as some for 19,20-EpDPA [37]. The studies discussed above have largely been aimed at the role of EpFAs as regulators of the cardiovascular system, not other desirable effects, such as their anti-inflammatory and antiangiogenic effects. Endogenous 8,9-EET has been shown to protect renal cells in vitro against nephrotoxicity caused by cisplatin [19]. Some 14,15-EET mimics have also demonstrated nephroprotective effects in vitro (e.g., **2** and **5**) [19,32].

**Figure 2:**
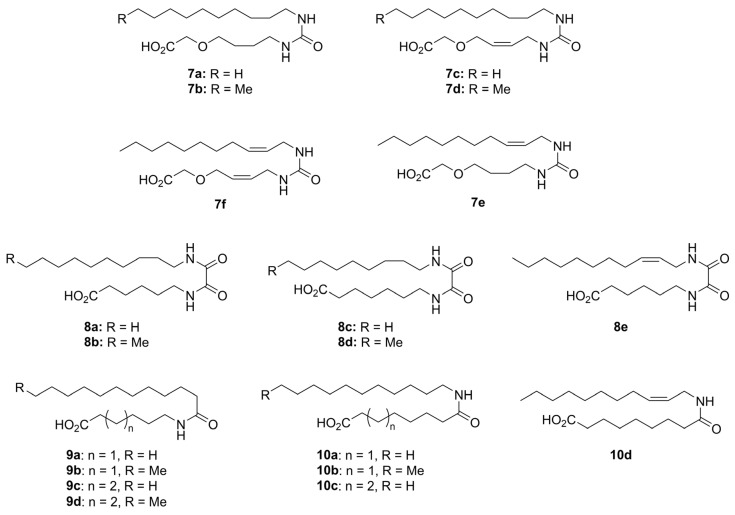
8,9-EET analogs synthesized in this study.

Inspired by previous studies of 14,15-EET and 17,18-EEQ mimics, a small library of potential 8,9-EET mimics was synthesized (Figure 2). The epoxide group in 8,9-EET was replaced with previously reported bioisosteres of the epoxide in EpFAs that would be stable towards sEH, such as urea (**7a**–**f**), oxamide (**8a**–**e**), and amide (**9a**–**d**, **10a**–**d**). Urea analogs containing (*Z*)-alkenes in the 5,6- and 12,13-position from the carboxylic acid were designed, as well as their saturated counterparts were envisaged. For the fully saturated analogs (**7a**–**b**), the carbon chain length from the urea group towards the omega end included two homologs (10 and 11 carbon atoms, respectively). Homologation towards the omega end has previously been shown to have a minor effect on activity [36]. An alpha-oxo group was included in all urea analogs (**7a**–**f**) to improve water solubility, prevent beta-oxidation, and simplify their syntheses. Alpha-oxo carboxylates have previously shown to retain the activity of the parent molecule, while having better water solubility and metabolic stability [30]. Oxamide groups have also been shown to be excellent substitutions for epoxides in EpFAs. Herein, we included five mimics containing oxamides (**8a**–**e**). The length of the carbon chain from carboxylate to first nitrogen varied from six (**8a**–**b, 8e**) to seven (**8c**–**d**). Two homologs of the carbon chain towards the omega end were included for each of the oxamide analogs (**8a** vs. **8b**, **8c** vs. **8d**). One analog also contained a (*Z*)-alkene in the allylic position towards the omega end (**8e**). The amides (**9a**–**d**, **10a**–**d**), much like before, featured variations in chain length, both between carboxylate and amide, as well as towards the omega end. Additionally, the amide group can have two different directions: the nitrogen atom can be facing the carboxylate end (e.g., **9d**) or the carbonyl group can be facing the carboxylate (e.g., **10b**).

### 2.2. Synthesis

**Scheme 1:**
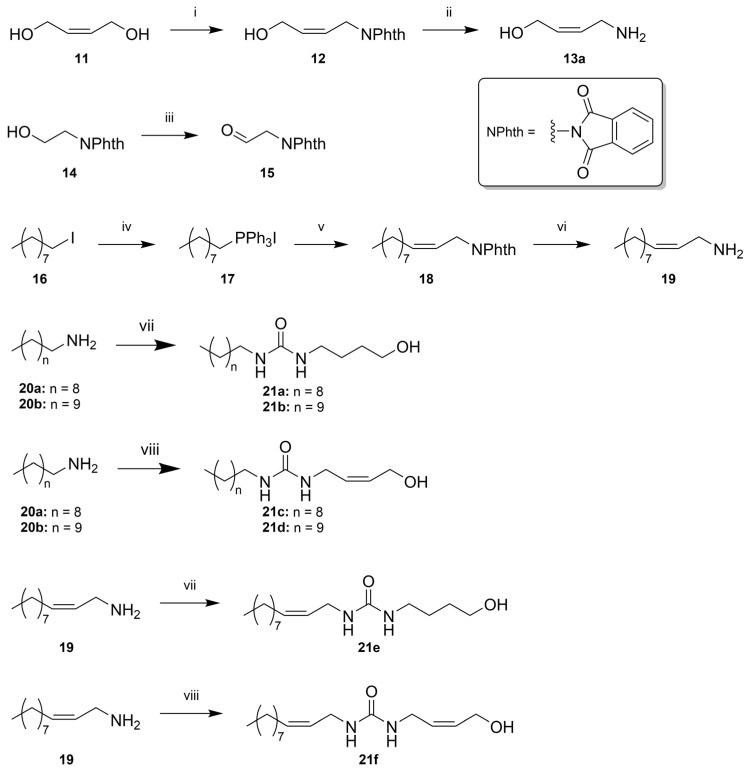
Reagents and conditions: (**i**) Phthalimide, PPh_3_, diisopropyl azodicarboxylate, rt, 16 h; (**ii**) N_2_H_4_·H_2_O, EtOH, reflux, 2 h; (**iii**) DMP, NaHCO_3_, CH_2_Cl_2_, rt, 18 h; (**iv**) PPh_3_, MeCN, reflux, 18 h; (**v**) Sodium bis(trimethylsilyl)amide (NaHMDS), THF, hexamethylphosphoramide, **15**, −78 °C, 18 h; (**vi**) N_2_H_4_·H_2_O, EtOH, reflux, 2 h; (**vii**) CDI, MeCN, 0 °C → rt, 30 min, then 4-aminobutan-1-ol (**13b**), rt, 30 min; (**viii**) CDI, MeCN, 0 °C → rt, 30 min, then **13a**, rt, 30 min.

The olefinic amines **13a** and **19** were synthesized as outlined in Scheme 1. Olefinic amine **13a** was synthesized from diol **11** in a two-step procedure. In the first step, one alcohol group was selectively functionalized using a Mitsunobu reaction with phthalimide [38], providing compound **12**. The reaction gave a moderate yield, due to a cumbersome purification. In the last step of the modified Gabriel synthesis, the phthalimide group was removed with hydrazine to give the amino alcohol **13a** in a good yield. Conversion of the free amine to its corresponding hydrochloride was indeed possible, but using hydrochlorides was not desirable, as it would require comprehensive optimization of the urea synthesis (see below).

A Wittig strategy was employed (Scheme 1) to synthesize the second olefinic amine **19**. Commercial phthalimide-protected ethanolamine **14** was oxidized to the aldehyde **15** using Dess-Martin periodinane (DMP) buffered with NaHCO_3_. The resulting aldehyde **15** was then reacted with the ylide of Wittig salt **17** (prepared from nonyl iodide (**16**) and PPh_3_) in a *Z*-selective Wittig reaction, giving the phthalimide protected olefinic amine **18**. Finally, removal of the phthalimide group was achieved with hydrazine, as before, affording the free amine **19** in a good yield.

All urea groups were introduced using 1,1′-carbonyldiimidazole (CDI) in conjunction with a fatty amine (**19** or **20a**–**b**) and an amino alcohol (**13a**–**b**) in a modified version of the procedure by Shore et al. [39], resulting in the asymmetric ureas shown in Scheme 1. The aliphatic amines **20a**–**b** and 4-aminobutanol were commercially available and was coupled to the olefinic amines (**13a** and 1**9**) synthesized above. Equimolar quantities of the two amines were added sequentially to a solution of CDI in acetonitrile, yielding the urea alcohols **21a**–**f**. The urea alcohols were highly polar and poorly soluble in all solvents tested (except **21f**) and were, thus, purified by recrystallization. When using commercial amines with known densities, which could be measured accurately, this methodology worked superbly. However, when using self-made amines, which were more difficult to measure accurately, significant quantities of symmetric ureas were sometimes produced. In those cases, work-up was laborious and yields were moderate to low.

**Scheme 2:**
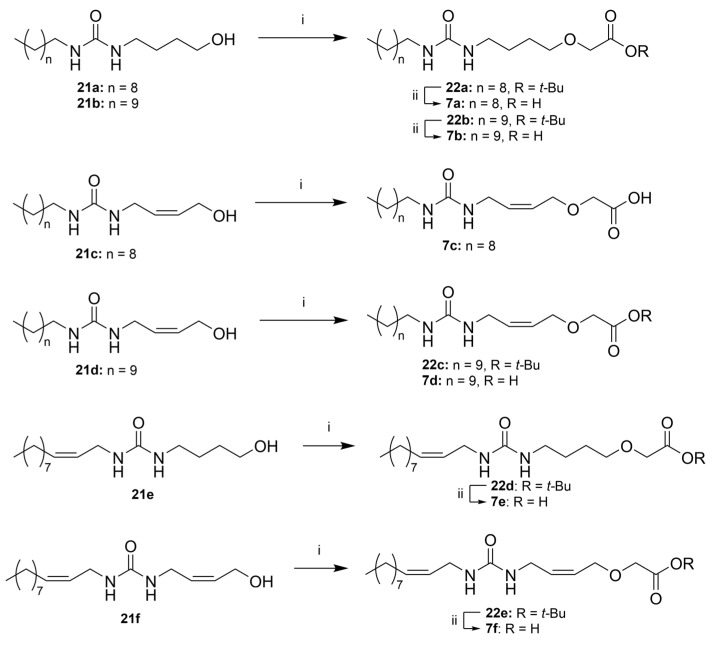
Reagents and conditions: (**i**) BrCH_2_CO_2_*t*Bu, Bu_4_NHSO_4_, CH_2_Cl_2_, NaOH (aq.), 0 °C → rt, 16 h—5 days; (**ii**) LiOH·H_2_O, H_2_O:THF:MeOH = 1:2:2, 0 °C, 3 h.

In the second to last step, synthesis of the fatty acid backbone was finalized via an ether synthesis, providing the target compounds **22a**–**e** as esters (Scheme 2). First, a common method for synthesis of alpha-oxo esters, using phase-transfer catalysis using Bu_4_NHSO_4_ and *tert*-butyl bromoacetate [40], was attempted. When employing this methodology on **21a**–**b**, conversion was poor and remaining undissolved starting material made phase separation during extraction very difficult. It was possible to force the reaction to completion by switching solvent from toluene to DCM, extending reaction time to several days and adding additional portions of *tert*-butyl bromoacetate numerous times. Obviously, these conditions were not optimal. Ester hydrolysis during the reaction was significant, as can be seen in the reaction of **21c**, where the carboxylic acid **7c** was the major product. Such copious use of *tert*-butyl bromoacetate is also undesirable. Further optimization of reaction conditions or a change in strategy is warranted if further analogs of this type are to be synthesized. Finally, hydrolyses of the esters were carried out under standard conditions (LiOH, H_2_O, MeOH). The reactions went smoothly, and the product could be purified by recrystallization (for **7a**–**e**) or chromatography (for **7f**).

**Scheme 3:**
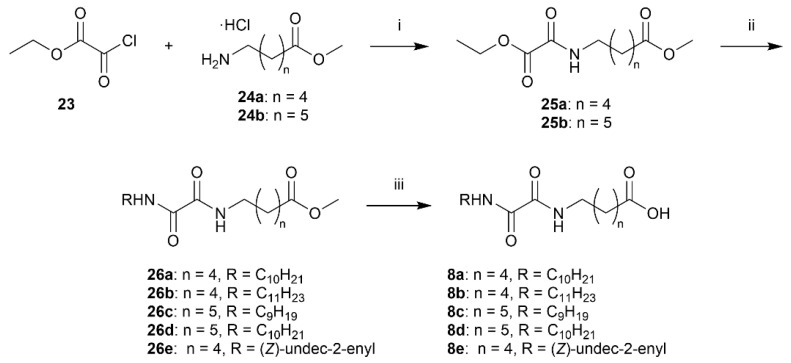
Reagents and conditions: (**i**) Et_3_N, CH_2_Cl_2_, rt, 2 h; (**ii**) *n*-nonylamine, *n*-decylamine or **19**, EtOH, rt, 3 h; (**iii**) LiOH·H_2_O, H_2_O:THF:MeOH = 1:2:2, rt, 3 h.

To produce oxamide analogs **8a**–**e**, a three-step procedure involving a selective condensation between an amine and the 2-oxamido substituted ester **25a**–**b** was envisaged (Scheme 3). In the first step, ethyl oxalyl chloride (**23**) was reacted with the hydrochloride salts of amino esters in the presence of the base triethylamine **24a**–**b**. Sufficient purity of the resulting intermediates **25a**–**b** could usually be achieved by merely washing the DCM with dilute aqueous acid. Next, the aliphatic amines (*n*-nonylamine, *n*-decylamine, *n*-undecylamine or **19**) reacted selectively [41,42] with the 2-oxamido substituted ester (**25a** or **25b**) over the aliphatic ester, giving oxamides **26a**–**e**. After hydrolysis, the oxamide analogs **8a**–**e** were obtained.

**Scheme 4:**
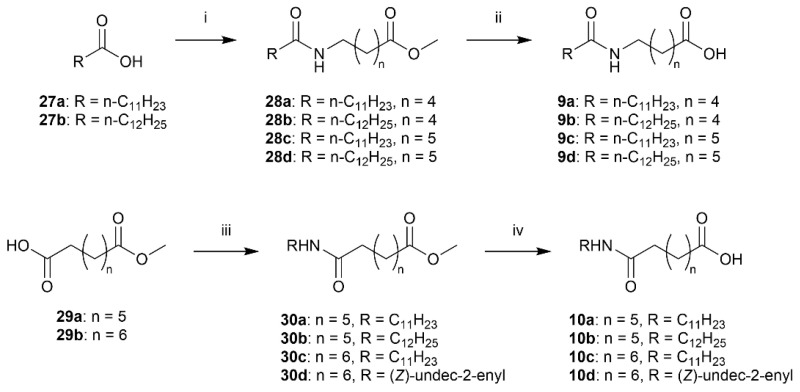
Reagents and conditions: (**i**) SOCl_2_, reflux, 2 h, then methyl 6-aminohexanoate**·**HCl (**24a**) or methyl 7-aminoheptanoate·HCl (**24b**), *i*Pr_2_NEt, MeCN, rt, 16 h; (**ii**) LiOH·H_2_O, H_2_O:THF:MeOH = 1:2:2, rt, 3 h; (**iii**) 1-Ethyl-3-(3-dimethylaminopropyl)carbodiimide hydrochloride (EDC·HCl), 4-dimethylaminopyridine, *n*-undecylamine, *n*-dodecylamine or amine **19**, CH_2_Cl_2_, rt, 16 h; (**iv**) LiOH·H_2_O, H_2_O:THF:MeOH 1:2:2, rt, 3 h.

Amide analogs **9a**–**d** were prepared in two steps (Scheme 4). The aliphatic carboxylic acids **27a**–**b** were converted to their corresponding acid chlorides by way of SOCl_2_, then reacted directly with the appropriate amino ester hydrochlorides in the presence of Hünig’s base, affording amides **28a**–**d** in typically good yields. Next, hydrolysis with LiOH gave the amide-containing analogs **9a**–**d**. The last four amide analogs were prepared (Scheme 4) by reacting the carboxy esters **29a**–**b** with the appropriate amines in an EDC-promoted peptide coupling [43], giving amides **30a**–**d** in moderate yields. Finally, ester hydrolysis afforded the final amide analogs **10a**–**d**.

### 2.3. Epoxide Hydrolase Inhibition

All compounds were evaluated as inhibitors of both human soluble epoxide hydrolase (sEH) and microsomal epoxide hydrolase (mEH). None of the compounds were significant (IC_50_ ≥ 50 µM) inhibitors of the mEH. However, many compounds were low to moderate inhibitors of sEH (Table 1). Analogs containing urea and amide isosteres were mediocre inhibitors (Entries 1–6 and 12–19), with IC_50_-values typically in the lower µM range. Those containing oxamide isosteres (Entries 7–11), however, were generally poor inhibitors of sEH. For reference, the most potent inhibitors here are several orders of magnitude less potent than, for example, the related 12-(3-(adamantan-1-yl)ureido)dodecanoic acid (AUDA) (IC_50_ 3 nM) [44]. Drug candidates targeting sEH, such as EC5026, are even more potent (IC_50_ < 0.05 nM) [45].

### 2.4. Evaluation of Nephroprotective Effects

**Figure 3:**
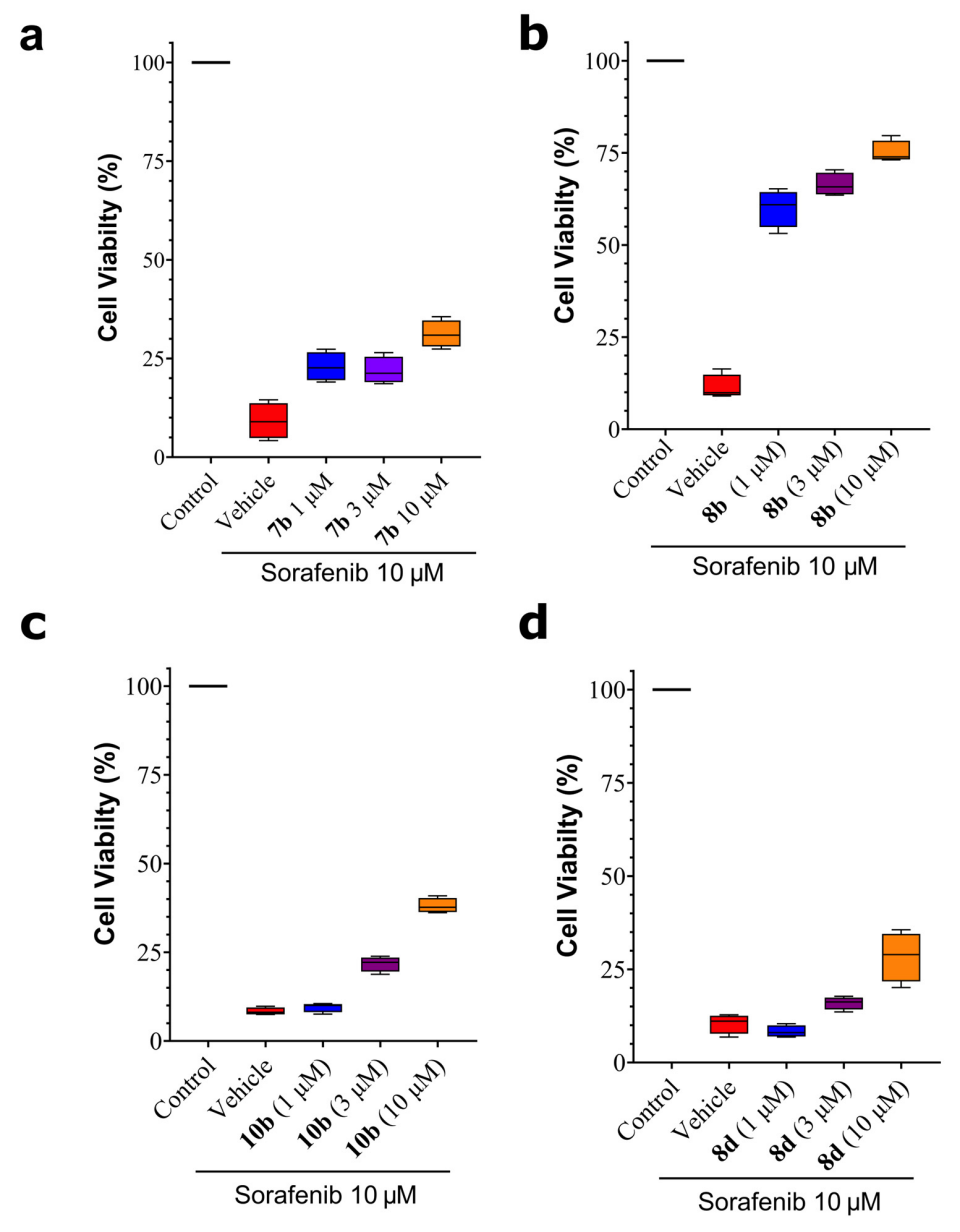
WST-8 assay of human renal mesangial cells treated with sorafenib and 8,9-EET mimics, (**a**) **7b**, (**b**) **8b**, (**c**) **10b**, (**d**) **8d**, at indicated concentrations after 48 h incubation, as percentages relative to control. Bars represent mean ± SEM (*n* = 4).

Cell viability of HRMCs treated with sorafenib was determined with a water-soluble tetrazolium salt (WST) assay (Table 1). Sorafenib exposure resulted in around 90% of the cell dying (Figure 3). Sorafenib has been reported as a potent sEH inhibitor [46]; however, it does not protect the cells because mesangial cells do not produce sufficient EETs, thus, providing a good background to test EET analogs. Analogs containing the urea isostere (Entries 1–6) were inactive, except for **7b** (Entry 3, Table 1 and Figure 3a), which exhibited significant activity even at low concentrations (1 µM). No explanation for why **7b** was active and none of the structural homologs (e.g., **7a**, Entry 2) were inactive could be found. The oxamide-containing analogs (Entries 7–11), however, all exhibited significant activity. Out of all the analogs tested, the saturated analog **8b** demonstrated superior efficacy and good potency, with an increase in cell viability of 64% at 10 µM and 50% at 1 µM (Entry 8, Table 1 and Figure 3b). Saturated analogs with the first nitrogen in 7-position (Entries 7–8), counting from the carboxylic acid, exhibited an increase in potency over their counterparts with the nitrogen in 8-position (Entries 9–10, Table 1 and Figure 3d). The effect of carbon chain length towards the omega end proved substantial for **8a**–**b**, with the C_11_-chain being superior. For **8c**–**d**, however, chain length towards the omega end made no significant difference. Interestingly, introduction of the *Z*-alkene in **8e** (Entry 11) resulted in diminished activity (cf. Entry 8) to the point of near insignificance. Analogs containing amide isosteres (Entries 12–19) exhibited activities ranging from good to none. Those compounds that had the amide group in 8,9-position relative to the carboxylic acid (Entries 14–17, Table 1 and Figure 3c) all showed activity at 10 µM, although none at 1 µM. Shifting of the amide group position to either 7,8-position (Entries 12–13) or 9,10-position (Entries 18–19) caused activity to drop to zero. The preference for 8,9-position observed here is not entirely surprising, as it mirrors that of an endogenous substrate (8,9-EET), at least on paper. Interestingly, it is worth noting that the distance between what is thought to be the primary pharmacophores, the carboxylic acid and oxamide, can be varied by one carbon atom (Entries 7–8 vs. 9–10) without greatly impacting activity. This phenomenon might be the result of the oxamide group consisting of two amides, which can interact with the target, rather molecular flexibility in the putative receptor.

Finally, the correlation between IC_50_ for sEH and nephroprotective effect (*E*_10µM_) was investigated, but none (*R*^2^ = 0.1) was found. Due to the most promising compound (**8b**) being an insignificant inhibitor of sEH and the general complete lack of correlation between sEH inhibition and efficacy, the results do indicate that the mode of action for the substances are not sEH inhibition, which is probably due to the lack of EETs production by the tested cells. The substances may be mimics of 8,9-EET or have some other yet unknown mechanism behind their nephroprotective effect that could be investigated in further studies.

## 3. Experimental

### 3.1. Synthetic Procedures

#### Materials and Equipment

All commercially available reagents and solvents were used without any further purification, unless otherwise stated. Analytical TLC was performed on silica gel 60 F254 aluminum-backed plates (Merck (Rahway, NJ, USA)). Column chromatography was performed on silica gel 60 (Merck). NMR spectra were recorded on either a Bruker AVI400, AVneo400, or Bruker AVI600 spectrometer. Chemical shifts (δ) are reported relative to the solvent residue signal in CDCl_3_ (7.27 ppm), CD_3_OD (3.31 ppm), DMSO-d_6_ (2.50 ppm) (^1^H-NMR), CDCl_3_ (77.0 ppm), CD_3_OD (49.0 ppm), and DMSO-d_6_ (39.52 ppm) (^13^C NMR). For mixtures of CDCl_3_ and CD_3_OD, CD_3_OD was used to lock the signal. Mass spectra were recorded by the Department of Chemistry, University of Oslo, on a Bruker maXis II and ESI ionization.

### 3.2. Synthetic Procedures

#### 3.2.1. General Procedure A: Urea Formation with 1,1′-Carbonyldiimidazole

1,1′-Carbonyldiimidazole (CDI) (1.00 equiv.) was dissolved in acetonitrile (0.8 M) and cooled to 0 °C under an argon atmosphere before adding a solution of the first amine (1.00 equiv.) dropwise. The solution was warmed to room temperature and stirred for 30 min. The second amine (1.00 equiv.) was added dropwise, and the reaction stirred for 90 min at room temperature. Work-up typically consisted of recrystallization.

#### 3.2.2. General Procedure B: Phase-Transfer Catalyzed Ether Synthesis

The alcohol was suspended/dissolved in CH_2_Cl_2_ (0.12 M), cooled to 0 °C and *n*-Bu_4_NHSO_4_ (0.50 equiv.) and NaOH (0.12 M) were added and stirred for 30 min. *tert*-Butyl bromoacetate (2.0 equiv.) was added and the mixture slowly warmed to room temperature. The reaction was stirred vigorously at room temperature and additional *tert*-butyl bromoacetate (1.0 equiv) was added. After the alcohol was fully consumed (The alcohol may be poorly soluble in CH_2_Cl_2_. In which case, prolonged reaction times and several additions of *tert*-butyl bromoacetate twice daily are necessary) as indicated by TLC, the solution was diluted with water, extracted three times with CH_2_Cl_2_, and the combined organic phases washed with brine. The solution was dried over MgSO_4_, evaporated in vacuo and purified by column chromatography.

#### 3.2.3. General Procedure C: Ester Hydrolysis

The ester was dissolved in tetrahydrofuran:methanol:water (2:2:1 *V*:*V*) (0.02 M for the total volume), cooled to 0 °C and LiOH·H_2_O (35 equiv.) was added. The solution/suspension was stirred for 3 h at 0 °C unless otherwise stated. The volatiles (chiefly methanol and tetrahydrofuran) were evaporated in vacuo at 25 °C, and the products were purified further according to the specific procedures.

#### 3.2.4. General Procedure D: Conversion of Carboxylic Acid to Acid Chloride

The carboxylic acid was taken up in SOCl_2_ (5.0 equiv.) and refluxed for two hours. Then, the solution was cooled and excess thionyl chloride evaporated in vacuo. The residue was used without further purification.

#### 3.2.5. General Procedure E: Condensation Between Amine and Oxoester

The ethyl 2-alkylamino-2-oxoacetate is taken up in EtOH (abs.). Amine (1.00 equiv.) is added in one portion under an argon atmosphere and then stirred for 3 h at room temperature. CHCl_3_ is added to the suspension until all was dissolved before concentrating in vacuo. The product is purified by column chromatography (SiO_2_).

##### (*Z*)-1-Phthalimido-4-hydroxybut-2-ene (**12**)

To a solution of (*Z*)-but-2-ene-1,4-diol (**11**) (2.00 g, 22.7 mmol) in dry THF (91 mL), phthalimide (2.23 g, 15.1 mmol, 0.67 equiv.) and triphenylphosphine (3.97 g, 15.1 mmol, 0.667 equiv.) were added. The solution was cooled to 0 °C and diisopropyl azodicarboxylate (3.0 mL, 15 mmol, 0.67 equiv.) was added over 1 h, slowly warmed to room temperature, and stirred overnight. Concentration in vacuo and purification using column chromatography (SiO_2_, 2:1 *n*-hexane:EtOAc) afforded **12** (2.26 g, 10.4 mmol, 46%) as colorless amorphous solids. R_f_ 0.50 (EtOAc, UV). ^1^H NMR (400 MHz, CDCl_3_) δ 7.84 (dd, *J* = 5.4, 3.1 Hz, 2H), 7.72 (dd, *J* = 5.4, 3.1, 2H), 5.90 (dtt, *J* = 10.8, 6.8, 1.2, 1H), 5.56 (dtt, *J* = 10.38 7.7, 1.2, 1H), 4.39 (ddd, *J* = 11.2, 7.3, 1.3 Hz, 4H); ^13^C NMR (101 MHz, CDCl_3_) δ 168.3, 134.3, 133.4, 132.2, 125.1, 123.5, 58.2, 34.6. Spectroscopic data are consistent with that reported in the literature [47].

##### (Z)-4-Aminobut-2-en-1-ol (**13a**)

Phthalimide **12** (1.81 g, 8.33 mmol) was dissolved in EtOH (42 mL), and hydrazine monohydrate (6.3 mL, 83 mmol, 10 equiv.) was added and refluxed for 2 h. The solution was cooled to room temperature, filtered, and the filtrate was concentrated in vacuo. Kugelrohr distillation (bp. 65 °C/0.8 mmHg) gave a colorless oil (532 mg, 6.11 mmol, 73%), which solidified at -18 °C. ^1^H NMR (400 MHz, CD_3_OD) δ 5.63 (m, 2H), 4.14 (m, 2H), 3.31 (m, 2H); ^13^C NMR (101 MHz, CD_3_OD) δ 131.3, 129.6, 57.0, 37.6. The ^1^H NMR spectrum is consistent with previously reported values [48].

##### N-(2-oxoethyl)-phthalimide (**15**)

*N*-(2-Hydroxyethyl)phthalimide (**14**) (0.961 g, 5.00 mmol, 1.00 equiv.) was dissolved in DCM (25 mL), and NaHCO_3_ (1.27 g, 30.0 mmol, 3.00 equiv.) was added, followed by DMP (2.82 g, 6.5 mmol, 1.30 equiv.) at room temperature. The reaction mixture was stirred overnight (18 h) and quenched by addition of a saturated aqueous solution of Na_2_S_2_O_3_ (50 mL). The layers were separated, and the aqueous layer was extracted with DCM (3 × 25 mL). The combined organic layers were washed with a saturated aq. NaHCO_3_ (20 mL), brine (20 mL), dried (MgSO_4_), filtered, and concentrated in vacuo. Purification by flash chromatography on silica gel (EtOAc:hexane 1:1) afforded the desired compound **15** as a white solid (0.648 g, 3.42 mmol, 63%). mp. 111–114 °C; R_f_: 0.28 (EtOAc:hexane 1:1); ^1^H NMR (400 MHz, CDCl_3_) δ 9.65 (s, 1H), 7.88 (dd, *J* = 5.5, 3.1 Hz, 2H), 7.75 (dd, *J* = 5.5, 3.1 Hz, 2H), 4.55 (s, 2H); ^13^C NMR (101 MHz, CDCl_3_) δ 194.0, 167.9 (2 × C), 134.8 (2 × C), 132.4 (2 × C), 124.1 (2 × C), 47.8. All spectroscopic and physical data were in agreement with those reported in the literature [49].

##### Nonyltriphenylphosphonium Iodide (**17**)

1-Iodononane (**16**) (1.27 g, 5.00 mmol, 1.00 equiv.) and PPh_3_ (2.62 g, 10.0 mmol, 2.00 equiv.) were dissolved in acetonitrile (45 mL) and stirred at reflux overnight (18 h). The mixture was allowed to cool down. After evaporation of the solvent, the crude was purified by flash chromatography on silica gel, starting with pure DCM, then changing the eluent to DCM 95:5 MeOH. This afforded the desired Wittig salt **17** as a viscous yellow oil (2.50 g, 4.85 mmol, 97%). R_f_ 0.62 (DCM:MeOH 95:5); ^1^H NMR (400 MHz, CDCl_3_) δ 7.87–7.62 (m, 15H), 3.68–3.49 (m, 2H), 1.63–1.55 (m, 4H), 1.31–1.05 (m, 10H), 0.81 (t, *J* = 6.8 Hz, 3H); ^13^C NMR (101 MHz, CDCl_3_) δ 135.2 (d, *J* = 3.0 Hz, 3 × C), 133.7 (d, *J* = 9.9 Hz, 6 × C), 130.6 (d, *J* = 12.5 Hz, 6 × C), 118.2 (d, *J* = 85.9 Hz, 3 × C), 31.8, 30.5 (d, *J* = 15.5 Hz), 29.2 (3 × C), 23.2 (d, *J* = 50.0 Hz), 22.7, 22.6, 14.1. All spectroscopic and physical data were in agreement with those reported in the literature [50].

##### (Z)-N-(Undec-2-en-1-yl)phthalimide (**18**)

The Wittig salt **17** (1.83 g, 3.55 mmol, 1.00 equiv.) was dissolved in dry THF (72 mL) and HMPA (12.0 mL). The solution was degassed and purged three times with nitrogen. Subsequently, the mixture was cooled to −78 °C and then NaHMDS (6.20 mL, 0.6 M in toluene, 3.73 mmol, 1.05 equiv.) was added dropwise. The reaction mixture changed from colorless to an orange color. The mixture was brought up to 0 °C, stirred for five minutes, and then re-cooled to −78 °C. A solution of aldehyde **15** (0.671 g, 3.55 mmol, 1.00 equiv.) dissolved in dry THF (8.00 mL) was added in a dropwise manner. The mixture was allowed to warm up to room temperature slowly in a dry ice/acetone bath for 18 h before being quenched by the addition of saturated aq. NaH_2_PO_4_ (30 mL). The phases were separated, and the aqueous phase was extracted with Et_2_O (3 × 40 mL). The combined organic phases were washed with brine (2 × 25 mL), dried (MgSO_4_), filtered, and the solvent was removed in vacuo. The crude product was purified by flash chromatography on silica gel (hexane:EtOAc 9:1) to afford the desired *Z*-alkene **18** as an oil (0.613 g, 2.05 mmol, 57%). R_f_ 0.48 (hexane:EtOAc 9:1); ^1^H NMR (400 MHz, CDCl_3_) δ 7.83 (dd, *J* = 5.4, 3.0 Hz, 2H), 7.70 (dd, *J* = 5.5, 3.0 Hz, 2H), 5.64 – 5.54 (m, 1H), 5.50 – 5.37 (m, 1H), 4.31 (m, 2H), 2.45 – 2.07 (m, 2H), 1.46 – 1.15 (m, 12H), 0.91 – 0.83 (m, 3H); ^13^C NMR (101 MHz, CDCl_3_) δ 168.5 (2 × C), 135.2 (2 × C), 134.3 (2 × C), 132.7 (2 × C), 123.6, 123.2, 35.3, 32.3, 29.9, 29.9, 29.8, 29.7, 27.8, 23.1, 14.6; HRMS (ESI/Q-TOF) *m*/*z*: [M+Na]^+^ calcd. for C_19_H_25_NO_2_Na 322.1777; found 322.1777.

##### (Z)-Undec-2-en-1-amine (**19**)

Phthalimide **18** (0.575 g, 1.93 mmol, 1.00 equiv.) was dissolved in EtOH (50 mL). Hydrazine monohydrate (1.54 mL, 19.3 mmol, 10.0 equiv.) was added and the mixture was refluxed until TLC revealed no starting material. After two hours, a white precipitate was formed, and the solution was cooled to 0 °C, filtrated, and the filtrate was concentrated in vacuo. Water (10 mL) was added, and the pH was adjusted to 12 with aq. NaOH (50%). Et_2_O (30 mL) was added, and the phases were separated. The aqueous phase was extracted with Et_2_O (3 × 30 mL), and the combined organic phases were washed with 1.0 M K_2_CO_3_ (20 mL) and brine (20 mL). The organic phases were dried over K_2_CO_3_, filtrated, and the solvent was removed in vacuo to afford the desired *Z*-amine **19** as a yellow oil (0.299 g, 1.77 mmol, 92%), which was used in the following step without further purification. ^1^H NMR (400 MHz, CDCl_3_) δ 5.67–5.18 (m, 2H), 3.30 (d, *J* = 6.2 Hz, 2H), 2.03 (q, *J* = 6.9 Hz, 2H), 1.51–1.39 (m, 2H), 1.37–1.19 (m, 12H), 0.90–0.83 (m, 3H); ^13^C NMR: (101 MHz, CDCl_3_) δ 131.4, 131.1, 39.3, 32.3, 30.1, 29.9, 29.7, 29.7, 27.7, 23.1, 14.5; IR: 3365 cm^−1^ and 3287 cm^−1^; HRMS (ESI/Q-TOF) *m*/*z*: [M+H]^+^ calcd. for C_11_H_24_N 170.1903; found 170.1903.

##### 1-Decyl-3-(4-hydroxybutyl)urea (**21a**)

Prepared according to *General Procedure A*, from 4-aminobutanol (**13b**) and n-decylamine (**20a**). The solids formed during the reaction were recrystallized from MeCN to afford urea **21a** (0.372 g, 1.37 mmol, 72%) as white needles. mp. 113–114; R_f_ 0.36 (9:1 DCM:MeOH); ^1^H NMR (400 MHz, CD_3_OD) δ 3.56 (m, 2H), 3.18–3.04 (m, 4H), 1.61–1.40 (m, 6H), 1.38–1.22 (m, 14H), 0.90 (m, 3H); ^13^C NMR (101 MHz, CD_3_OD) δ 160.7, 62.0, 40.4, 40.2, 32.4, 30.7, 30.2, 30.1, 30.1, 29.9, 29.8, 27.3, 27.2, 23.1, 13.8; HRMS (ESI/Q-TOF) *m*/*z*: [M+Na]^+^ calcd. for C_15_H_32_N_2_O_2_Na 295.2356; found 295.2355.

##### 1-(4-Hydroxybutyl)-3-undecylurea (**21b**)

Prepared according to *General Procedure A*, using *n*-undecylamine (**20b**) and 4-aminobutanol (**13b**). Recrystallization from MeCN gave **21b** (582 mg, 2.03 mmol, 91%) as white needles. mp. 116–117 °C; R_f_ 0.52 (9:1 DCM:MeOH). ^1^H NMR (400 MHz, CD_3_OD) δ 3.62–3.54 (m, 2H), 3.18–3.07 (m, 4H), 1.62–1.43 (m, 6H), 1.38–1.29 (m, 16H), 0.92 (t, *J* = 6.9 Hz, 3H). ^13^C NMR (101 MHz, CD_3_OD) δ 159.9, 61.2, 39.6, 39.4, 31.7, 30.0, 29.5, 29.3, 29.1, 29.1, 26.6, 26.4, 22.3, 13.0. HRMS (ESI/Q-TOF) *m*/*z*: [M+Na]^+^ calcd. for C_16_H_34_N_2_O_2_Na 309.2512; found 309.2512.

##### (Z)-1-Decyl-3-(4-hydroxybut-2-en-1-yl)urea (**21c**)

Prepared according to *General Procedure A*, from aminoalcohol **13a** and *n*-decylamine (**20a**). The solids formed during the reaction were recrystallized from MeCN to give the urea **21c** (223 mg, 72%) as white needles. mp 97–98 °C; R_f_ 0.41 (9:1 DCM:MeOH); ^1^H NMR (400 MHz, CD_3_OD) δ 5.65 (dtt, *J* = 11.1, 6.5, 1.6 Hz, 1H), 5.49 (dtt, *J* = 11.1, 6.8, 1.5 Hz, 1H), 4.16 (d, *J* = 6.6 Hz, 1H), 3.77 (d, *J* = 6.8 Hz, 2H), 3.09 (t, *J* = 7.0 Hz, 2H), 1.45 (q, *J* = 6.8 Hz, 2H), 1.38–1.23 (m, 14H), 0.90 (m, 3H); ^13^C NMR (101 MHz, CD_3_OD) δ 159.7, 130.3, 128.7, 57.0, 39.7, 36.6, 31.7, 29.9, 29.3, 29.3, 29.1, 29.1, 26.5, 22.3, 13.0; HRMS (ESI/Q-TOF) *m*/*z*: [M+Na]^+^ calcd. for C_15_H_30_N_2_O_2_Na 293.2199; found 293.2199.

##### (Z)-1-(4-Hydroxybut-2-en-1-yl)-3-undecylurea (**21d**)

Prepared according to *General Procedure A*, from *n*-undecylamine (**20b**) and aminoalcohol **13a**. The product was recrystallized from first MeCN, then CHCl_3_, to give **21d** (169 mg, 0.59 mmol, 52%), which was contaminated with diundecylurea (~20 mol%). mp. 100–102 °C; R_f_ 0.34 (9:1 DCM:MeOH); ^1^H NMR (400 MHz, CD_3_OD) δ 5.67 (ddd, *J* = 12.7, 8.1, 1.5 Hz, 1H), 5.51 (dtt, *J* = 11.0, 6.8, 1.5 Hz, 1H), 4.18 (d, *J* = 6.5 Hz, 2H), 3.80 (d, *J* = 7.2 Hz, 2H), 3.21–3.00 (m, 3H), 1.60–1.42 (m, 3H), 1.42–1.20 (m, 20H), 0.92 (t, *J* = 6.9 Hz, 4H); ^13^C NMR (101 MHz, CD_3_OD) δ 160.5, 131.1, 129.4, 57.8, 40.4, 37.3, 32.4, 30.7, 30.1, 29.9, 29.8, 27.3, 23.1, 13.8; HRMS (ESI/Q-TOF) *m*/*z*: [M+Na]^+^ calcd. for C_16_H_32_N_2_O_2_Na 307.2356; found 307.2355.

##### (Z)-1-(4-Hydroxybutyl)-3-(undec-2-en-1-yl)urea (**21e**)

Prepared according to *General Procedure A*, from 4-aminobutanol (**13b**) and allylic amine **19**. Work-up: crystallization from MeCN and washing with cold MeCN afforded urea **21e** (0.262 g, 0.92 mmol, 72%). mp. 90–92 °C; R_f_ 0.19 (DCM:MeOH 95:5); ^1^H NMR (400 MHz, CD_3_OD) δ 5.61–5.46 (m, 1H), 5.44–5.30 (m, 1H), 3.76 (dd, *J* = 6.7, 1.5 Hz, 2H), 3.63–3.50 (m, 2H), 3.18–3.09 (m, 2H), 2.10 (q, *J* = 6.8 Hz, 2H), 1.64–1.51 (m, 4H), 1.43–1.20 (m, 12H), 0.91 (t, *J* = 6.7, 3H); ^13^C NMR (101 MHz, CD_3_OD) δ 161.1, 133.3, 127.8, 62.6, 40.8, 38.0, 33.1, 30.9, 30.7, 30.6, 30.4, 30.4, 28.3, 27.8, 23.7, 14.4. HRMS (ESI/Q-TOF) *m*/*z*: [M+Na]^+^ calcd. for C_16_H_32_N_2_O_2_Na 307.2355; found 307.2356.

##### 1-((Z)-4-Hydroxybut-2-en-1-yl)-3-((Z)-undec-2-en-1-yl)urea (**21f**)

Prepared from allylic amine **19** and aminoalcohol **13a** according to *General Procedure A*. Column chromatography twice (SiO_2_, 6% MeOH in DCM) afforded **21f** (28 mg, 99 µmol, 20%) as a waxy solid. R_f_ 0.22 (94:6 DCM:MeOH); ^1^H NMR (400 MHz, CD_3_OD) δ 5.65 (dtt, *J* = 11.0, 6.5, 1.6 Hz, 1H), 5.54–5.44 (m, 2H), 5.39 (dtt, *J* = 10.9, 6.7, 1.4 Hz, 1H), 4.16 (d, *J* = 6.5 Hz, 2H), 3.82–3.71 (m, 4H), 2.10 (app. q, *J* = 7.2, 6.6 Hz, 2H), 1.42–1.24 (m, 15H), 0.89 (app. t, 3H); ^13^C NMR (101 MHz, CD_3_OD) δ 160.3, 132.7, 131.2, 129.3, 127.1, 57.8, 49.0, 48.8, 48.6, 48.6, 48.4, 48.4, 48.1, 47.9, 47.7, 37.4, 37.3, 32.4, 30.1, 30.0, 29.8, 29.7, 27.6, 23.1, 13.8; HRMS (ESI/Q-TOF) *m*/*z*: [M+Na]^+^ calcd. for C_16_H_30_N_2_O_2_Na 305.2199; found 305.2198.

##### *tert*-Butyl 2-(4-(3-decylureido)butoxy)acetate (**22a**)

Prepared according to *General Procedure B*, starting from **21a**. Reaction time: 5 days. Column chromatography (SiO_2_, 2:3 *n*-heptane:EtOAc) afforded **22a** (226 mg, 0.58 mmol, 64%) as a white wax; R_f_ 0.45 (EtOAc). ^1^H NMR (400 MHz, CDCl_3_) δ 5.20 (br, 2H), 3.96 (s, 2H), 3.52 (t, *J* = 5.5 Hz, 2H), 3.22 (t, *J* = 6.1 Hz, 2H), 3.15 (t, *J* = 7.1 Hz, 2H), 1.73–1.58 (m, 4H), 1.52–1.40 (m, 11H), 1.33–1.18 (m, 14H), 0.87 (m, 3H); ^13^C NMR (101 MHz, CDCl_3_) δ 170.3, 158.9, 82.2, 71.7, 69.0, 40.6, 40.5, 32.0, 30.4, 29.7, 29.6, 29.5, 28.2, 27.8, 27.1, 27.1, 22.8, 14.2; HRMS (ESI/Q-TOF) *m*/*z*: [M+Na]^+^ calcd. for C_21_H_42_N_2_O_4_Na 409.3037; found 409.3037. Acidification of the combined aqueous phases with conc. HCl (37%) to pH 2, twice extraction with EtOAc and evaporation in vacuo yielded the carboxylic acid **7a** (30 mg, 0.09 mmol, 10%) after recrystallization from MeOH. See the respective section for **7a** below for characterization data.

##### *tert*-Butyl 2-(4-(3-undecylureido)butoxy)acetate (**22b**)

Prepared from **21b** according to *General Procedure B*. Reaction time: 6 days. Purification by column chromatography (SiO_2_, EtOAc:*n*-heptane 3:1) gave **22b** (211 mg, 0.52 mmol, 72%) as a white wax. R_f_ 0.24 (2:1 hexane:EtOAc); ^1^H NMR (400 MHz, CDCl_3_) δ 3.97 (s, 2H), 3.53 (t, *J* = 5.5 Hz, 2H), 3.24 (t, *J* = 6.0 Hz, 2H), 3.16 (t, *J* = 7.1 Hz, 2H), 1.72–1.60 (m, 4H), 1.48 (s, 10H), 1.34–1.22 (m, 20H), 0.87 (t, *J* = 6.8 Hz, 3H); ^13^C NMR (101 MHz, CDCl_3_) δ 170.3, 158.9, 82.2, 71.7, 69.0, 40.6, 40.6, 32.0, 30.4, 29.8, 29.7, 29.6, 29.5, 28.3, 27.8, 27.1, 22.8, 14.3; HRMS (ESI/Q-TOF) *m*/*z*: [M+Na]^+^ calcd. for C_22_H_44_N_2_O_4_Na 423.319; found 423.319.

##### (Z)-2-((4-(3-Decylureido)but-2-en-1-yl)oxy)acetic Acid (**7c**)

Employing *General Procedure B*, starting from **21c**. Reaction time: 2 days. The reaction resulted in primarily the carboxylic acid **7c**. The aqueous phase was acidified with HCl (conc.) to pH 1–2 and extracted three times with EtOAc. Evaporation in vacuo and recrystallization from MeOH afforded **7c** (115 mg, 0.35 mmol, 44%) as white solids. mp. 82–84 °C; ^1^H NMR (400 MHz, CD_3_OD) δ 5.70–5.57 (m, 2H), 4.23–4.14 (m, 2H), 4.08 (s, 2H), 3.83 – 3.75 (m, 2H), 3.09 (t, *J* = 7.0 Hz, 2H), 1.52–1.41 (m, 2H), 1.38–1.23 (m, 14H), 0.90 (app. t, 3H); ^13^C NMR (101 MHz, CD_3_OD) δ 173.4, 160.4, 131.8, 127.6, 67.3, 66.9, 40.4, 37.5, 32.4, 30.7, 30.1, 30.1, 29.9, 29.8, 27.3, 23.1, 13.8; HRMS (ESI/Q-TOF) *m*/*z*: [M+Na]^+^ calcd. for C_17_H_32_N_2_O_4_Na 351.2254; found 351.2253.

##### *tert*-Butyl (Z)-2-((4-(3-undecylureido)but-2-en-1-yl)oxy)acetate (**22c**)

Prepared according to *General Procedure B*, starting from **21d**. Reaction time: 2 days. Column chromatography twice (SiO_2_, EtOAc:*n*-heptane 3:2 and CH_2_Cl_2_:EtOAc 1:1, respectively) gave **22c** as a wax (111 mg, 0.28 mmol, 49%). The product was still contaminated with diundecylurea (~15 mol%). R_f_ 0.49 (EtOAc); ^1^H NMR (400 MHz, CDCl_3_) δ 5.84–5.67 (m, 2H), 4.12 (d, *J* = 5.9 Hz, 2H), 3.98 (s, 2H), 3.87 (d, *J* = 6.5 Hz, 2H), 3.15 (t, *J* = 7.1 Hz, 2H), 1.48 (s, 10H), 1.35–1.22 (m, 17H), 0.87 (t, *J* = 6.8 Hz, 3H); ^13^C NMR (101 MHz, CDCl_3_) δ 170.1, 158.5, 132.4, 127.3, 82.3, 68.2, 66.8, 40.7, 37.6, 32.0, 30.4, 29.8, 29.7, 29.5, 29.5, 28.2, 28.2, 27.1, 22.8, 14.3; HRMS (ESI/Q-TOF) *m*/*z*: [M+Na]^+^ calcd. for C_22_H_42_N_2_O_4_Na 421.3037; found 421.3036.

##### *tert*-Butyl (Z)-2-(4-(3-(undec-2-en-1-yl)ureido)butoxy)acetate (22d) and (Z)-2-(4-(3-(undec-2-en-1-yl)ureido)butoxy)acetic Acid (**7e**)

Prepared according to *General procedure B*, starting from **21e**. Reaction time: overnight. Work-up: purification of the product from the organic phase by column chromatography (SiO_2_, hexane:EtOAc 1:1) gave the *tert*-butyl ester **22d** (69.0 mg, 0.17 mmol, 19%) as a clear oil. R_f_ 0.28 (hexane:EtOAc 4:1); ^1^H NMR (400 MHz, CDCl_3_) δ 5.51–5.33 (m, 2H), 3.94 (s, 2H), 3.78 (dd, *J* = 6.5, 1.3 Hz, 2H), 3.50 (t, *J* = 5.7 Hz, 2H), 3.20 (t, *J* = 6.2 Hz, 2H), 2.03 (q, *J* = 6.9 Hz, 2H), 1.78–1.58 (m, 4H), 1.45 (s, 9H), 1.34–1.23 (m, 12H), 0.85 (t, *J* = 6.8 Hz, 3H); ^13^C NMR (101 MHz, CDCl_3_) δ 170.2, 158.9, 132.7, 126.8, 82.1, 71.6, 68.9, 40.4, 37.6, 32.0, 29.7, 29.6, 29.4 (2 × C), 28.2 (3 × C), 27.7, 27.4, 27.0, 22.7, 14.2; HRMS (ESI/Q-TOF) *m*/*z*: [M+Na]^+^ calcd. for C_22_H_42_N_2_O_4_Na 421.3037; found 421.3036.

The aqueous phase was acidified with HCl (conc.) to pH 2 and extracted with EtOAc (3 × 10 mL), and the combined organic phases were dried (MgSO_4_), filtrated, and concentrated in vacuo. The crude product was recrystallized using MeOH as a solvent. This afforded carboxylic acid **7e** (29.1 mg, 0.08 mmol, 9%) as a crystalline solid. See the section for **7e** below for characterization data.

##### *tert*-Butyl 2-(((Z)-4-(3-((Z)-undec-2-en-1-yl)ureido)but-2-en-1-yl)oxy)acetate (**22e**)

Prepared from **21f**, according to *General procedure B*. Elution with EtOAc:*n*-heptane 1:1 gave **22e** (12 mg, 29 µmol, 32%) as a clear oil. R_f_ 0.59 (EtOAc). ^1^H NMR (400 MHz, CDCl_3_) δ 5.84–5.68 (m, 2H), 5.55–5.45 (m, 1H), 5.44–5.33 (m, 1H), 4.12 (d, *J* = 5.8 Hz, 2H), 3.97 (s, 2H), 3.88 (d, *J* = 6.2 Hz, 2H), 3.81 (d, *J* = 5.4 Hz, 2H), 2.05 (q, *J* = 7.3 Hz, 2H), 1.48 (s, 9H), 1.36–1.21 (m, 13H), 0.87 (t, *J* = 7.0 Hz, 3H). ^13^C NMR (101 MHz, CDCl_3_) δ 170.1, 158.4, 133.2, 132.2, 127.4, 126.3, 82.3, 68.2, 66.8, 37.8, 37.7, 32.0, 29.7, 29.6, 29.4, 28.3, 27.5, 22.8, 14.2. HRMS (ESI/Q-TOF) *m*/*z*: [M+Na]^+^ calcd. for C_22_H_40_N_2_O_4_Na 419.2880; found 419.2879.

##### 2-(4-(3-Decylureido)butoxy)acetic Acid (**7a**)

Prepared according to *General procedure C*, from *tert*-butyl ester **22a**. Afterwards, HCl (conc.) was added under cooling until pH 2, and the solution extracted with EtOAc (4 × 50 mL (the product is poorly soluble in EtOAc)). Evaporation in vacuo and recrystallization from MeOH afforded **7a** (101 mg, 0.31 mmol, 64%) as a white solid, mp. 103–105 °C. ^1^H NMR (400 MHz, CD_3_OD) δ 4.05 (s, 2H), 3.55 (t, *J* = 6.1 Hz, 2H), 3.14 (t, *J* = 6.7 Hz, 2H), 3.09 (t, *J* = 7.0 Hz, 2H), 1.69–1.52 (m, 4H), 1.50–1.42 (m, 2H), 1.30 (m, 14H), 0.90 (m, 3H); ^13^C NMR (101 MHz, CD_3_OD) δ 173.7, 160.7, 71.7, 68.2, 40.4, 40.1, 32.4, 30.7, 30.1, 30.1, 29.9, 29.8, 27.3, 27.3, 23.1, 13.8. HRMS (ESI/Q-TOF) *m*/*z*: [M+Na] ^+^ calcd. for C_17_H_34_N_2_O_4_Na 353.2411; found 353.2410.

##### 2-(4-(3-Undecylureido)butoxy)acetic Acid (**7b**)

Prepared according to *General procedure C*, starting from **21b**. Evaporation of the organic phase and recrystallization from MeOH gave **7b** (82 mg, 0.24 mmol, 52%) as white solids. mp. 109–110 °C. ^1^H NMR (400 MHz, CD_3_OD) δ 4.06 (s, 2H), 3.54 (t, *J* = 6.1 Hz, 2H), 3.14 (t, *J* = 6.9 Hz, 2H), 3.09 (t, *J* = 7.0 Hz, 2H), 1.69–1.50 (m, 4H), 1.52–1.41 (m, 2H), 1.37–1.22 (m, 16H), 0.89 (app. t, 3H). ^13^C NMR (101 MHz, CD_3_OD) δ 173.6, 160.7, 71.7, 68.1, 40.4, 40.1, 32.4, 30.7, 30.1, 29.9, 29.8, 27.3, 27.3, 27.3, 23.1, 13.8. HRMS (ESI/Q-TOF) *m*/*z*: [M+Na]^+^ calcd. for C_18_H_36_N_2_O_4_Na 367.2567; found 367.2567.

##### *tert*-Butyl (Z)-2-((4-(3-undecylureido)but-2-en-1-yl)oxy)acetate (**7d**)

Prepared according to *General procedure C*, starting from **21d**. Column chromatography twice (SiO_2_, EtOAc:*n*-heptane 3:2 and CH_2_Cl_2_:EtOAc 1:1, respectively) gave **7d** as a waxy solid (111 mg, 0.28 mmol, 49%). R_f_ 0.49 (EtOAc). The product was still contaminated with some diundecylurea (~15 mol%). ^1^H NMR (400 MHz, CDCl_3_) δ 5.84–5.67 (m, 2H), 4.12 (d, *J* = 5.9 Hz, 2H), 3.98 (s, 2H), 3.87 (d, *J* = 6.5 Hz, 2H), 3.15 (t, *J* = 7.1 Hz, 2H), 1.48 (s, 10H), 1.35–1.22 (m, 17H), 0.87 (t, *J* = 6.8 Hz, 3H). ^13^C NMR (101 MHz, CDCl_3_) δ 170.1, 158.5, 132.4, 127.3, 82.3, 68.2, 66.8, 40.7, 37.6, 32.0, 30.4, 29.8, 29.7, 29.5, 29.5, 28.2, 28.2, 27.1, 22.8, 14.3. HRMS (ESI/Q-TOF) *m*/*z*: [M+Na]^+^ calcd. for C_22_H_42_N_2_O_4_Na 421.3037; found 421.3036.

##### (Z)-2-(4-(3-(undec-2-en-1-yl)ureido)butoxy)acetic Acid (**7e**):

See the procedure for **22d** above. The procedure gave **7e** (29.1 mg, 0.08 mmol, 9%) as a crystalline solid. R_f_ 0.29 (DCM:MeOH 9:1); mp. 89–90 °C; ^1^H NMR (400 MHz, CD_3_OD) δ 5.54–5.45 (m, 1H), 5.44–5.35 (m, 1H), 4.05 (s, 2H), 3.75 (dd, *J* = 6.6, 1.4 Hz, 2H), 3.54 (t, *J* = 6.1 Hz, 2H), 3.14 (t, *J* = 6.6 Hz, 2H), 2.09 (q, *J* = 7.0 Hz, 2H), 1.75–1.51 (m, 4H), 1.40–1.13 (m, 12H), 0.90 (t, *J* = 6.6 Hz, 3H); ^13^C NMR (101 MHz, CD_3_OD) δ 174.3, 161.2, 133.3, 127.9, 72.3, 68.8, 40.8, 38.1, 33.1, 30.7, 30.7, 30.6, 30.4, 30.4, 28.3, 27.9, 23.7, 14.4; HRMS (ESI/Q-TOF) *m*/*z*: [M + 2Na]^+^ calcd. for C_18_H_33_NO_4_Na_2_ 387.2230; found 387.2230.

##### 2-(((Z)-4-(3-((Z)-undec-2-en-1-yl)ureido)but-2-en-1-yl)oxy)acetic Acid (**7f**)

Prepared from **22e** according to *General procedure C*. The reaction mixture was diluted with water and acidified with HCl (conc.) until pH 2–3. The solution was extracted with EtOAc (2 × 30 mL), dried over MgSO_4_, and evaporated in vacuo. Column chromatography (SiO_2_, AcOH:EtOAc:heptane 1:5:5) gave **7f** (5.5 mg, 16 µmol, 55%) as a waxy solid. ^1^H NMR (400 MHz, CD_3_OD) δ 5.71–5.57 (m, 1H), 5.55–5.33 (m, 1H), 4.18 (d, *J* = 5.0 Hz, 1H), 4.06 (s, 1H), 3.80 (d, *J* = 5.2 Hz, 1H), 3.75 (d, *J* = 6.7 Hz, 1H), 2.10 (app. q, *J* = 7.0 Hz, 1H), 1.42–1.27 (m, 6H), 0.94–0.86 (m, 1H). ^13^C NMR (101 MHz, CD_3_OD) δ 173.8, 160.3, 132.7, 131.7, 127.7, 127.1, 67.6, 66.8, 37.5, 37.5, 32.4, 30.1, 30.0, 29.8, 29.7, 27.6, 23.1, 13.8. HRMS (ESI/Q-TOF) *m*/*z*: [M+Na]^+^ calcd. for C_18_H_32_N_2_O_4_Na 363.2254; found 363.2254.

##### Methyl 6-(2-ethoxy-2-oxoacetamido)hexanoate (**25a**)

Methyl 6-aminohexanoate hydrochloride (**24a**) (99 mg, 0.56 mmol) is dissolved in DCM (6.9 mL) and cooled in an ice bath. TEA (169 µL, 1.22 mmol, 2.2 equiv.) and ethyl chlorooxoacetate (**23**) (67.7 µL, 0.61 mmol, 1.1 equiv.) is added. After 5 min, the ice bath is removed and the reaction is stirred for 2 h. The solution is transferred to a separatory funnel and washed with HCl (1 M). The organic phase is dried over Na_2_SO_4_ and evaporated in vacuo (Excess acid chloride is removed by evaporation) to afford **25a** (127 mg, 0.53 mmol, 94%) as a yellow oil. ^1^H NMR (400 MHz, CDCl_3_) δ 7.14 (s, 1H), 4.32 (q, *J* = 7.1 Hz, 2H), 3.65 (s, 3H), 3.32 (app. q, 2H), 2.30 (t, *J* = 7.4 Hz, 2H), 1.72–1.50 (m, 4H), 1.44–1.29 (m, 5H). ^13^C NMR (101 MHz, CDCl_3_) δ 174.0, 160.9, 156.7, 63.3, 51.6, 39.7, 33.9, 28.9, 26.4, 24.5, 14.1. HRMS (ESI/Q-TOF) *m*/*z*: [M+Na]^+^ calcd. for C_11_H_19_NO_5_Na 268.1155; found 268.1155.

##### Methyl 7-(2-ethoxy-2-oxoacetamido)heptanoate (**25b**)

Methyl 6-aminoheptanoate hydrochloride (**24b**) (600 mg, 3.07 mmol) is dissolved in DCM (38 mL) and cooled in an ice bath. TEA (941 µL, 6.75 mmol, 2.2 equiv.) and ethyl chlorooxoacetate (**23**) (377 µL, 3.37 mmol, 1.1 equiv.) is added. After 5 min, the ice bath is removed and the reaction stirred for 2 h. The solution is transferred to a separatory funnel and washed with HCl (1 M). The organic phase is dried over Na_2_SO_4_ and evaporated in vacuo (excess acid chloride is removed by evaporation) to afford **25b** as a yellow oil. R_f_ 0.51 (EtOAc) ^1^H NMR (400 MHz, CDCl_3_) δ 7.03 (s, 1H), 4.28 (q, *J* = 7.1 Hz, 2H), 3.60 (s, 3H), 3.27 (app. q, 2H), 2.24 (t, *J* = 7.5 Hz, 2H), 1.65–1.43 (m, 5H), 1.38–1.21 (m, 7H). ^13^C NMR (101 MHz, CDCl_3_) δ 174.1, 160.9, 156.6, 63.2, 51.5, 39.8, 33.9, 29.0, 28.7, 26.4, 24.7, 14.0. HRMS (ESI/Q-TOF) *m*/*z*: [M+Na]^+^ calcd. for C_12_H_21_NO_5_Na 282.1312; found 282.1314.

##### Methyl 6-(2-(decylamino)-2-oxoacetamido)hexanoate (**26a**)

*General procedure E* is employed using **25a** and *n*-decylamine. Column chromatography (SiO_2_, 3:2 EtOAc:heptane) afforded **26a** (158 mg, 0.44 mmol, 82%) as a white solid, mp. 100–101 °C. R_f_ 0.64 (EtOAc). ^1^H NMR (400 MHz, CDCl_3_) δ 7.64–7.42 (m, 2H), 3.65 (s, 3H), 3.34–3.23 (m, 4H), 2.30 (t, *J* = 7.4 Hz, 2H), 1.70–1.47 (m, 6H), 1.40–1.18 (m, 16H), 0.86 (t, *J* = 6.8 Hz, 3H). ^13^C NMR (101 MHz, CDCl_3_) δ 174.0, 160.0, 159.9, 51.6, 39.8, 39.5, 33.9, 32.0, 29.6, 29.6, 29.4, 29.3, 29.0, 26.9, 26.4, 24.6, 22.8, 14.2. HRMS (ESI/Q-TOF) *m*/*z*: [M+Na]^+^ calcd. for C_19_H_36_N_2_O_4_Na 379.2567; found 379.2567.

##### Methyl 6-(2-oxo-2-(undecylamino)acetamido)hexanoate (**26b**)

Prepared from **25a** and *n*-undecylamine according to *General procedure E*. Column chromatography (SiO_2_, 3:2 EtOAc:heptane) afforded **26b** (162 mg, 0.44 mmol, 94%) as a white solid. mp. 104 °C. R_f_ 0.59 (EtOAc). ^1^H NMR (400 MHz, CDCl_3_) δ 7.63–7.37 (m, 2H), 3.65 (s, 3H), 3.28 (app. p, 4H), 2.30 (t, *J* = 7.4 Hz, 2H), 1.70–1.48 (m, 6H), 1.42–1.17 (m, 18H), 0.86 (t, *J* = 6.8 Hz, 3H). ^13^C NMR (101 MHz, CDCl_3_) δ 174.0, 160.0, 159.9, 51.6, 39.8, 39.6, 33.9, 32.0, 29.7, 29.7, 29.6, 29.4, 29.3, 29.0, 27.0, 26.4, 24.6, 22.8, 14.2. HRMS (ESI/Q-TOF) *m*/*z*: [M+Na]^+^ calcd. for C_20_H_38_N_2_O_4_Na 393.2724; found 393.2723.

##### Methyl 7-(2-(nonylamino)-2-oxoacetamido)heptanoate (**26c**)

Prepared from **25b** and *n*-nonylamine according to *General procedure E*. Column chromatography (SiO_2_, 3:2 EtOAc:heptane) afforded **26c** (419 mg, 1.18 mmol, 84%) as a white solid. mp. 114 °C. R_f_ 0.63 (EtOAc). ^1^H NMR (400 MHz, CDCl_3_) δ 7.63–7.38 (m, 2H), 3.65 (s, 3H), 3.29 (q, *J* = 6.6 Hz, 4H), 2.29 (t, *J* = 7.5 Hz, 2H), 1.66–1.49 (m, 6H), 1.39–1.19 (m, 16H), 0.86 (t, *J* = 7.0 Hz, 3H). ^13^C NMR (101 MHz, CDCl_3_) δ 174.2, 160.0, 159.9, 51.6, 39.8, 39.7, 34.0, 32.0, 29.6, 29.3, 29.2, 28.8, 26.9, 26.6, 24.9, 22.8, 14.2. HRMS (ESI/Q-TOF) *m*/*z*: [M+Na]^+^ calcd. for C_19_H_36_N_2_O_4_Na 379.2567; found 379.2567.

##### Methyl 7-(2-(decylamino)-2-oxoacetamido)heptanoate (**26d**)

Prepared from **25b** and *n*-decylamine according to *General procedure E*. Column chromatography (SiO_2_, 3:2 EtOAc:heptane) afforded **26d** (411 mg, 1.11 mmol, 83%) as a white solid, mp. 111 °C. R_f_ 0.61 (EtOAc). ^1^H NMR (400 MHz, CDCl_3_) δ 7.59–7.42 (m, 2H), 3.65 (s, 3H), 3.33–3.23 (m, 4H), 2.29 (t, *J* = 7.5 Hz, 2H), 1.66–1.48 (m, 6H), 1.39–1.18 (m, 18H), 0.86 (app. t, *J* = 6.8 Hz, 3H). ^13^C NMR (101 MHz, CDCl_3_) δ 174.2, 160.0, 159.9, 51.6, 39.8, 39.7, 34.0, 32.0, 29.6, 29.6, 29.4, 29.3, 29.2, 28.8, 27.0, 26.6, 24.9, 22.8, 14.2. HRMS (ESI/Q-TOF) *m*/*z*: [M+Na]^+^ calcd. for C_20_H_38_N_2_O_4_Na 393.2724; found 393.2723.

##### Methyl (Z)-6-(2-oxo-2-(undec-2-en-1-ylamino)acetamido)hexanoate (**26e**)

The aminooxoacetate **25a** (74.0 mg, 0.300 mmol, 1.00 equiv.) was dissolved in EtOH (0.5 mL) and added *Z*-amine **19** (51.0 mg, 0.300 mmol, 1.00 equiv.) in one portion at rt. The product precipitates quickly, and the reaction was stirred for 2.5 h. The solvent was evaporated, and the crude was purified by flash chromatography on silica gel (heptane:EtOAc 2:1) to afford the desired oxamide **26e** (75.0 mg, 0.204 mmol, 68%) as a white crystalline solid. R_f_ 0.28 (heptane:EtOAc 2:1); mp. 85–86 °C; ^1^H NMR (400 MHz, CDCl_3_) δ 7.49–7.33 (m, 2H), 5.61 (dtt, *J* = 10.7, 7.4, 1.6 Hz, 1H), 5.39 (dtt, *J* = 10.4, 7.0, 1.6 Hz, 1H), 4.32–3.82 (m, 2H), 3.67 (s, 3H), 3.31 (q, *J* = 6.9 Hz, 2H), 2.31 (t, *J* = 7.4 Hz, 2H), 2.17–1.95 (m, 2H), 1.70–1.52 (m, 4H), 1.45–1.19 (m, 14H), 0.94–0.82 (m, 3H); ^13^C NMR (101 MHz, CDCl_3_) δ 173.7, 159.6, 159.5, 134.7, 123.4, 51.4, 39.3, 36.6, 33.7, 31.7, 29.3, 29.2, 29.1 (2 × C), 28.8, 27.2, 26.1, 24.3, 22.5, 13.9; HRMS (ESI/Q-TOF) *m*/*z*: [M+Na]^+^ calcd. for C_20_H_36_N_2_O_4_Na 391.2567; found 391.2567.

##### 6-(2-(Decylamino)-2-oxoacetamido)hexanoic Acid (**8a**)

Ester **26a** was hydrolyzed using *General procedure C*. After cooling to 0 °C, the solution was filtered and washed with cold water. The solids were taken up in diluted HCl, extracted with EtOAc (3 × 100 mL) and dried over MgSO_4_. Concentration in vacuo afforded the acid **8a** (115 mg, 0.31 mmol, 82%) as a pale yellow solid. mp. 142–144 °C; ^1^H NMR (400 MHz, DMSO) δ 11.98 (s(br), 1H), 8.68 (app. q, 2H), 3.10 (q, *J* = 6.7 Hz, 4H), 2.18 (t, *J* = 7.4 Hz, 2H), 1.58–1.36 (m, 6H), 1.34–1.14 (m, 16H), 0.85 (t, *J* = 6.7 Hz, 3H); ^13^C NMR (101 MHz, DMSO) δ 174.4, 160.0, 160.0, 38.8, 38.6, 33.6, 31.3, 29.0, 28.7, 28.7, 28.5, 26.3, 25.9, 24.2, 22.1, 14.0; HRMS (ESI/Q-TOF) *m*/*z*: [M+Na]^+^ calcd. for C_18_H_34_N_2_O_4_Na 365.2411; found 365.2410.

##### 6-(2-Oxo-2-(undecylamino)acetamido)hexanoic Acid (**8b**)

Prepared by hydrolyzing **26b** using *General procedure C*. After cooling to 0 °C, the solution was filtered and washed with cold water. The solids were taken up in dilute HCl, extracted with EtOAc (3 × 100 mL), and dried over MgSO_4_. Concentration in vacuo afforded the acid **8b** (104 mg, 0.29 mmol, 79%) as a pale yellow solid. mp. 142–145 °C. ^1^H NMR (400 MHz, DMSO) δ 11.97 (s, 1H), 8.78–8.59 (m, 2H), 3.11 (app. q, *J* = 6.8 Hz, 4H), 2.19 (t, *J* = 7.4 Hz, 2H), 1.46 (dq, *J* = 15.3, 7.6 Hz, 6H), 1.24 (s, 18H), 0.86 (t, *J* = 6.7 Hz, 3H); ^13^C NMR (101 MHz, DMSO) δ 174.4, 160.0, 160.0, 38.8, 38.6, 33.6, 31.3, 29.0, 29.0, 28.7, 28.7, 28.5, 26.3, 25.9, 24.2, 22.1, 14.0; HRMS (ESI/Q-TOF) *m*/*z*: [M+Na]^+^ calcd. for C_19_H_36_N_2_O_4_Na 379.2567; found 379.2567.

##### 7-(2-(Nonylamino)-2-oxoacetamido)heptanoic Acid (**8c**)

Prepared by hydrolyzing **26c** using *General procedure C*. After cooling to 0 °C, the solution was filtered and washed with cold water. The solids were taken up in dilute HCl, extracted with EtOAc (3 × 100 mL), and dried over MgSO_4_. Concentration in vacuo and recrystallization from first MeOH then CHCl_3_ afforded the acid **8c** (130 mg, 0.38 mmol, 33%) as a white solid. mp. 148 °C; ^1^H NMR (400 MHz, DMSO) δ 11.96 (s, 1H), 8.76–8.57 (m, 2H), 3.10 (app. q, *J* = 6.8 Hz, 4H), 2.18 (t, *J* = 7.4 Hz, 2H), 1.52–1.37 (m, 6H), 1.32–1.16 (m, 16H), 0.85 (t, *J* = 6.7 Hz, 3H); ^13^C NMR (101 MHz, DMSO) δ 174.5, 160.0, 160.0, 38.8, 38.7, 33.6, 31.3, 28.9, 28.7, 28.6, 28.6, 28.2, 26.3, 26.0, 24.4, 22.1, 13.9; HRMS (ESI/Q-TOF) *m*/*z*: [M+Na]^+^ calcd. for C_18_H_34_N_2_O_4_Na 365.2411; found 365.2410.

##### 7-(2-(Decylamino)-2-oxoacetamido)heptanoic Acid (**8d**)

Prepared by hydrolyzing **27d** using *General procedure C*. After cooling to 0 °C, the solution was filtered and washed with cold water. The solids were taken up in dilute HCl, extracted with EtOAc (3 × 100 mL), and dried over MgSO_4_. Concentration in vacuo afforded the acid **8d** (288 mg, 0.81 mmol, 74%) as a white solid. mp. 146–147 °C; ^1^H NMR (400 MHz, DMSO) δ 11.95 (s, 1H), 8.75–8.61 (m, 2H), 3.18–3.04 (m, 4H), 2.19 (t, *J* = 7.4 Hz, 2H), 1.54–1.39 (m, 6H), 1.32–1.15 (m, 19H), 0.91–0.80 (m, 3H); ^13^C NMR (101 MHz, DMSO) δ 174.9, 160.5, 39.2, 34.1, 31.8, 29.4, 29.2, 28.7, 26.8, 26.5, 24.9, 22.6, 14.4; HRMS (ESI/Q-TOF) *m*/*z*: [M+Na]^+^ calcd. for C_19_H_36_N_2_O_4_Na 379.2567; found 379.2567.

##### (Z)-6-(2-Oxo-2-(undec-2-en-1-ylamino)acetamido)hexanoic Acid (**8e**)

Prepared by hydrolyzing **26e** using *General procedure C*. Work-up: the reaction mixture was acidified with 1.0 M HCl (7.0 mL) to pH 2 and extracted with EtOAc (3 × 10 mL). The combined organic phases were dried (Na_2_SO_4_), and the solvent was removed in vacuo. The crude mixture was purified by flash chromatography on silica gel (1:1 → 0:1 heptane:EtOAc) to afford the desired product **8e** (20.1 mg, 0.057 mmol, 77%) as a white crystalline solid. R_f_ 0.49 (EtOAc); mp. 124–126 °C; ^1^H NMR (400 MHz, CDCl_3_) δ 7.74–7.58 (m, 1H), 7.57–7.46 (m, 1H), 5.75–5.48 (m, 1H), 5.46–5.33 (m, 1H), 4.11–3.82 (m, 2H), 3.32 (q, *J* = 6.9 Hz, 2H), 2.36 (t, *J* = 7.4 Hz, 2H), 2.15–2.01 (m, 2H), 1.73–1.50 (m, 4H), 1.47–1.13 (m, 14H), 0.88 (t, *J* = 6.7 Hz, 3H); ^13^C NMR (101 MHz, CDCl_3_) δ 178.2, 159.9, 159.9, 135.0, 123.7, 39.6, 36.9, 33.7, 32.0, 29.6, 29.6, 29.4 (2 × C), 29.0, 27.6, 26.3, 24.3, 22.8, 14.2; HRMS (ESI/Q-TOF) *m*/*z*: [M+Na]^+^ calcd. for C_19_H_34_N_2_O_4_Na 377.2411; found 377.2410.

##### Methyl 6-dodecanamidohexanoate (**28a**)

The acid chloride of dodecanoic acid (**27a**) (0.79 g, 4.0 mmol, 2.0 equiv.) was prepared according to *General procedure D*. Afterwards, methyl 6-aminohexanoate·HCl (**24a**) (363 mg, 2.00 mmol) was dissolved in MeCN (10 mL) and cooled to 0 °C. The acid chloride in DCM (3.0 mL) and DIPEA (0.70 mL, 4.0 mmol, 2.0 equiv.) was added and the ice bath removed. After stirring overnight, the suspension was concentrated in vacuo and taken up in DCM. The organic phase was washed twice with diluted HCl (1 M) and diluted K_2_CO_3_, before drying the organic phase over MgSO_4_ and evaporating in vacuo. Purification by column chromatography (SiO_2_, 1:1 EtOAc:*n*-heptane) afforded amide **28a** (309 mg, 0.94 mmol, 47%) as a white solid. mp. 62–63 °C; R_f_ 0.50 (EtOAc); ^1^H NMR (400 MHz, CDCl_3_) δ 5.58 (s, 1H), 3.66 (s, 3H), 3.30–3.18 (m, 2H), 2.31 (t, *J* = 7.4 Hz, 2H), 2.15 (app. t, 2H), 1.70–1.56 (m, 4H), 1.51 (p, *J* = 7.8 Hz, 2H), 1.40–1.18 (m, 18H), 0.87 (t, *J* = 6.8 Hz, 3H); ^13^C NMR (101 MHz, CDCl_3_) δ 174.1, 173.5, 51.5, 39.3, 36.7, 33.8, 31.9, 29.6, 29.6, 29.5, 29.4, 29.3, 29.3, 29.2, 26.3, 25.9, 24.4, 22.7, 14.1; HRMS (ESI/Q-TOF) *m*/*z*: [M+Na]^+^ calcd. for C_19_H_37_NO_3_Na 350.2666; found 350.2665.

##### Methyl 6-tridecanamidohexanoate (**28b**)

The acid chloride of tridecanoic acid (**27b**) (499 mg, 2.33 mmol, 1.6 equiv.) was prepared according to *General procedure D*. Afterwards, methyl 6-aminohexanoate·HCl (**24a**) (265 mg, 1.45 mmol) was dissolved in MeCN (7.3 mL) and cooled to 0 °C. The acid chloride in DCM (2.0 mL) and DIPEA (0.60 mL, 3.4 mmol, 2.4 equiv.) was added and the ice bath removed. After stirring overnight, the suspension was concentrated in vacuo. Purification by column chromatography (SiO_2_, 3:2 EtOAc:*n*-heptane) afforded amide **28b** (439 mg, 1.29 mmol, 88%) as a white solid. mp. 68–69 °C; R_f_ 0.54 (EtOAc); ^1^H NMR (400 MHz, CDCl_3_) δ 5.75 (s, 1H), 3.68 (s, 3H), 3.27 (app. q, 2H), 2.33 (t, *J* = 7.4 Hz, 2H), 2.18 (app. t, 2H), 1.72–1.58 (m, 4H), 1.58–1.48 (m, 2H), 1.43–1.21 (m, 20H), 0.89 (t, *J* = 6.7 Hz, 3H); ^13^C NMR (101 MHz, CDCl_3_) δ 174.1, 173.4, 51.5, 39.3, 36.8, 33.8, 31.9, 29.7, 29.6, 29.6, 29.5, 29.4, 29.4, 29.3, 29.2, 26.3, 25.9, 24.4, 22.7, 14.1; HRMS (ESI/Q-TOF) *m*/*z*: [M+Na]^+^ calcd. for C_20_H_39_NO_3_Na 364.2822; found 364.2821.

##### Methyl 7-dodecanamidoheptanoate (**28c**)

The acid chloride of dodecanoic acid (**27a**) (300 mg, 1.50 mmol, 1.33 equiv.) was prepared according to *General procedure D*. Methyl 7-aminoheptanoate·HCl (**24b**) (220 mg, 1.12 mmol) was dissolved in MeCN (7.5 mL) and cooled to 0 °C. The acid chloride in DCM (1.0 mL) and TEA (0.37 mL, 2.6 mmol, 2.3 equiv.) were added and the ice bath removed. After stirring overnight, the suspension was concentrated in vacuo and taken up in DCM. The organic phase was washed with dilute HCl (1 M), dried over MgSO_4_, and evaporated in vacuo. Purification by column chromatography (SiO_2_, 3:2 EtOAc:*n*-heptane) afforded amide **28c** (370 mg, 1.08 mmol, 96%) as a white solid. mp. 73 °C; R_f_ 0.55 (EtOAc); ^1^H NMR (400 MHz, CDCl_3_) δ 5.66 (s, 1H), 3.65 (s, 3H), 3.22 (app. q, *J* = 6.7 Hz, 2H), 2.29 (t, *J* = 7.5 Hz, 2H), 2.14 (t, *J* = 7.7 Hz, 2H), 1.67–1.54 (m, 4H), 1.54–1.42 (m, 2H), 1.39–1.17 (m, 20H), 0.86 (t, *J* = 6.8 Hz, 3H); ^13^C NMR (101 MHz, CDCl_3_) δ 174.3, 173.5, 51.6, 39.5, 36.9, 34.0, 32.0, 29.7, 29.7, 29.6, 29.5, 29.5, 29.4, 29.4, 28.8, 26.6, 26.0, 24.9, 22.8, 14.2; HRMS (ESI/Q-TOF) *m*/*z*: [M+Na]^+^ calcd. for C_20_H_39_NO_3_Na 364.2822; found 364.2822.

##### Methyl 7-tridecanamidoheptanoate (**28d**)

The acid chloride of tridecanoic acid (**27b**) (321 mg, 1.52 mmol, 1.33 equiv.) was prepared according to *General procedure D*. Afterwards, methyl 7-aminoheptanoate·HCl (**24b**) (221 mg, 1.12 mmol) was dissolved in MeCN (7.5 mL) and cooled to 0 °C. The acid chloride in DCM (1.0 mL) and TEA (0.37 mL, 2.6 mmol, 2.3 equiv.) were added, and the ice bath was removed. After stirring overnight, the suspension was concentrated in vacuo. Purification by column chromatography (SiO_2_, 1:1 EtOAc:*n*-heptane) afforded amide **28d** (348 mg, 0.98 mmol, 91%) as a white solid. mp 72–73 °C; R_f_ 0.54 (EtOAc); ^1^H NMR (400 MHz, CDCl_3_) δ 5.62 (s, 1H), 3.64 (s, 3H), 3.21 (app. q/m, *J* = 6.7 Hz, 2H), 2.28 (t, *J* = 7.5 Hz, 2H), 2.13 (t, *J* = 7.8 Hz, 2H), 1.66–1.54 (m, 4H), 1.53–1.42 (m, 2H), 1.37–1.17 (m, 22H), 0.86 (t, *J* = 7.0 Hz, 3H); ^13^C NMR (101 MHz, CDCl_3_) δ 174.3, 173.4, 51.6, 39.5, 36.9, 34.0, 32.0, 29.8, 29.7, 29.7, 29.6, 29.5, 29.5, 29.5, 29.4, 28.8, 26.6, 26.0, 24.9, 22.8, 14.2; HRMS (ESI/Q-TOF) *m*/*z*: [M+Na]^+^ calcd. for C_21_H_41_NO_3_Na 378.2979; found 378.2978.

##### 6-Dodecanamidohexanoic Acid (**9a**)

Ester **28a** was hydrolyzed according to *General procedure C*. After cooling to 0 °C, the solution was filtered and washed with cold water. The solids were taken up in diluted HCl, extracted thrice with EtOAc and dried over MgSO_4_. Concentration in vacuo afforded carboxylic acid **9a** (104 mg, 0.33 mmol, 47%) as white solids. mp. 89–90 °C; ^1^H NMR (400 MHz, CD_3_OD) δ 3.16 (t, *J* = 7.0 Hz, 2H), 2.29 (t, *J* = 7.4 Hz, 2H), 2.16 (t, *J* = 7.5 Hz, 2H), 1.67–1.46 (m, 6H), 1.42–1.23 (m, 18H), 0.89 (app. t, 3H); ^13^C NMR (101 MHz, CD_3_OD) δ 176.9, 175.6, 39.5, 36.5, 34.2, 32.4, 30.1, 30.0, 29.8, 29.8, 29.6, 29.5, 26.9, 26.5, 25.1, 23.1, 13.8; HRMS (ESI/Q-TOF) *m*/*z*: [M+Na]^+^ calcd. for C_18_H_35_NO_3_Na 336.2509; found 336.2508.

##### 6-Tridecanamidohexanoic Acid (**9b**)

Ester **28b** was hydrolyzed using *General procedure C*. After cooling to 0 °C, the solution was filtered and washed with cold water. The solids were taken up in diluted HCl, extracted thrice with EtOAc and dried over MgSO_4_. Concentration in vacuo afforded the acid **9b** (172 mg, 0.53 mmol, 45%) as a white solid. mp. 93–94 °C; ^1^H NMR (400 MHz, CD_3_OD) δ 3.16 (t, *J* = 7.0 Hz, 2H), 2.29 (t, *J* = 7.4 Hz, 2H), 2.16 (t, *J* = 7.5 Hz, 2H), 1.69–1.46 (m, 6H), 1.42–1.21 (m, 20H), 0.95–0.84 (m, 3H); ^13^C NMR (101 MHz, CD_3_OD) δ 176.8, 175.6, 39.5, 36.5, 34.2, 32.4, 30.1, 30.1, 30.1, 30.0, 29.8, 29.8, 29.6, 29.5, 26.9, 26.5, 25.1, 23.1, 13.8; HRMS (ESI/Q-TOF) *m*/*z*: [M+Na]^+^ calcd. for C_20_H_39_NO_3_Na 364.2822; found 364.2821.

##### 7-Dodecanamidoheptanoic Acid (**9c**)

Prepared by hydrolyzing **28c** using *General procedure C*. After cooling to 0 °C, the solution was filtered and washed with cold water. The solids were taken up in dilute HCl, extracted with EtOAc (3 × 30 mL) and dried over MgSO_4_. Concentration in vacuo afforded the acid **9c** (280 mg, 0.85 mmol, 81%) as a white solid. mp. 104–105 °C; ^1^H NMR (400 MHz, CD_3_OD) δ 3.23–3.13 (m, 2H), 2.30 (t, *J* = 7.4 Hz, 2H), 2.18 (t, *J* = 7.4 Hz, 2H), 1.58 (dp, *J* = 43.5, 7.2 Hz, 6H), 1.42–1.25 (m, 20H), 0.92 (t, *J* = 6.7 Hz, 3H); ^13^C NMR (101 MHz, CD_3_OD) δ 176.9, 175.6, 39.6, 36.5, 34.2, 32.4, 30.1, 30.0, 29.8, 29.8, 29.6, 29.6, 29.2, 27.0, 26.5, 25.4, 23.1, 13.8; HRMS (ESI/Q-TOF) *m*/*z*: [M+Na]^+^ calcd. for C_19_H_37_NO_3_Na 350.2666; found 350.2665.

##### 7-Tridecanamidoheptanoic Acid (**9d**)

Prepared by hydrolyzing **28d** using *General procedure C*. After cooling to 0 °C, the solution was filtered and washed with cold water. The solids were taken up in dilute HCl, extracted with EtOAc (3 × 30 mL) and dried over MgSO_4_. Concentration in vacuo afforded the acid **9d** (278 mg, 0.81 mmol, 85%) as a white solid. mp. 105 °C; ^1^H NMR (400 MHz, CD_3_OD) δ 3.16 (t, *J* = 7.1 Hz, 2H), 2.28 (t, *J* = 7.4 Hz, 2H), 2.16 (t, *J* = 7.4 Hz, 2H), 1.68–1.43 (m, 6H), 1.43–1.17 (m, 22H), 0.97–0.83 (m, 3H); ^13^C NMR (101 MHz, CD_3_OD) δ 177.6, 176.2, 40.3, 37.2, 34.9, 33.1, 30.8, 30.7, 30.7, 30.5, 30.4, 30.3, 30.3, 29.9, 27.7, 27.1, 26.0, 23.7, 14.4; HRMS (ESI/Q-TOF) *m*/*z*: [M+Na]^+^ calcd. for C_20_H_39_NO_3_Na 364.2822; found 364.2822.

##### Methyl 8-oxo-8-(undecylamino)octanoate (**30a**)

Methyl 7-carboxyheptanoate (187 µL, ~200 mg, 1.1 mmol) was taken up in dry DCM (5.3 mL). EDC hydrochloride (0.461 mg, 2.40 mmol, 2.2 equiv.) and *N*,*N*-dimethylaminopyridine (DMAP) (261 mg, 2.40 mmol, 2.0 equiv.) were added and the mixture stirred for 30 min. Then, *n*-undecylamine (229 µL, 1.06 mmol, 1.0 equiv.) was added in one portion and the reaction stirred overnight. The reaction was quenched with water (5 mL) and the phases separated. The organic phase was washed with dilute HCl, dried over MgSO_4_ and concentrated in vacuo. Column chromatography (SiO_2_, 1:1 EtOAc:heptane) gave **30a** (202 mg, 0.59 mmol, 56%) as a white solid. mp. 71–72 °C; R_f_ 0.49 (EtOAc); ^1^H NMR (400 MHz, CDCl_3_) δ 5.49 (s, 1H), 3.65 (s, 3H), 3.27–3.17 (m, 2H), 2.29 (t, *J* = 7.5 Hz, 2H), 2.14 (t, *J* = 7.6 Hz, 2H), 1.69–1.54 (m, 4H), 1.47 (p, *J* = 7.1 Hz, 2H), 1.39–1.15 (m, 20H), 0.87 (t, *J* = 6.8 Hz, 3H); ^13^C NMR (101 MHz, CDCl_3_) δ 174.3, 173.1, 51.6, 39.7, 36.8, 34.1, 32.0, 29.8, 29.7, 29.7, 29.5, 29.4, 29.0, 28.9, 27.1, 25.7, 24.9, 22.8, 14.2; HRMS (ESI/Q-TOF) *m*/*z*: [M+Na]^+^ calcd. for C_20_H_39_NO_3_Na 364.2822; found 364.2822.

##### Methyl 8-(dodecylamino)-8-oxooctanoate (**30b**)

Methyl 7-carboxyheptanoate (195 mg, 1.04 mmol) was taken up in dry DCM (5.3 mL). EDC hydrochloride (415 mg, 2.16 mmol, 2.0 equiv.) and DMAP (260 mg, 2.13 mmol, 2.0 equiv.) were added and the mixture stirred for 30 min. Then, *n*-dodecylamine (190 mg, 1.03 mmol, 1.0 equiv.) was added in one portion and the reaction stirred overnight. The reaction was quenched with water (5 mL) and the phases separated. The organic phase was washed with dilute HCl, dried over MgSO_4_ and concentrated in vacuo. Column chromatography (SiO_2_, 1:1 EtOAc:heptane) gave **30b** (250 mg, 0.70 mmol, 66%) as a white solid. mp. 73–74 °C; R_f_ 0.56 (EtOAc); ^1^H NMR (400 MHz, CDCl_3_) δ 5.53 (s, 1H), 3.65 (s, 3H), 3.21 (app. q, 2H), 2.28 (t, *J* = 7.5 Hz, 2H), 2.14 (t, *J* = 7.6 Hz, 2H), 1.68–1.55 (m, 4H), 1.53–1.41 (m, 2H), 1.39–1.19 (m, 22H), 0.86 (t, *J* = 6.8 Hz, 3H); ^13^C NMR (101 MHz, CDCl_3_) δ 174.3, 173.1, 51.6, 39.7, 36.8, 34.1, 32.0, 29.8, 29.8, 29.7, 29.7, 29.7, 29.5, 29.4, 29.0, 28.9, 27.0, 25.7, 24.9, 22.8, 14.2; HRMS (ESI/Q-TOF) *m*/*z*: [M+Na]^+^ calcd. for C_21_H_41_NO_3_Na 378.2979; found 378.2978.

##### Methyl 9-oxo-9-(undecylamino)nonanoate (**30c**)

*n*-Undecylamine (0.432 mL, 2.00 mmol 4.00 equiv.), DMAP (0.244 g, 2.00 mmol, 4.00 equiv.), and methyl 8-carboxyoctanoate (0.100 g, 0.49 mmol, 1.00 equiv.) were dissolved in dry DMF (4 mL) and EDCI hydrochloride (0.382 g, 2.00 mmol, 4.00 equiv.) was added portion-wise. After 18 h, the reaction mixture was diluted with water (10 mL) and extracted with Et_2_O (3 × 10 mL). The combined organic phases were washed with brine (10 mL), dried (Na_2_SO_4_), filtrated, and the solvent was removed in vacuo. The residue was purified by flash chromatography on silica gel (heptane:EtOAc 1:1) to afford **30c** (0.108 g, 0.30 mmol, 61%) as a crystalline solid. R_f_ 0.19 (heptane:EtOAc 1:1); mp.: 72–73 °C; ^1^H NMR (400 MHz, CDCl_3_) δ 5.52 (br s, 1H), 3.66 (s, 3H), 3.23 (td, *J* = 7.2, 5.5 Hz, 2H), 2.29 (t, *J* = 7.5 Hz, 2H), 2.15 (t, *J* = 7.5 Hz, 2H), 1.73–1.53 (m, 4H), 1.53–1.42 (m, 3H), 1.35–1.18 (m, 22H), 0.87 (t, *J* = 6.7 Hz, 3H); ^13^C NMR: (101 MHz, CDCl_3_) δ = 174.1, 173.1, 51.3, 39.5, 36.5, 33.8, 31.7, 29.4 (3 × C), 29.4, 29.1, 29.1, 28.8, 28.7 (2 × C), 26.7, 25.6, 24.7, 22.5, 13.9; HRMS (ESI/Q-TOF) *m*/*z*: [M+Na]^+^ calcd. for C_21_H_41_NO_3_Na 378.2978; found 378.2979.

##### Methyl (Z)-9-oxo-9-(undec-2-en-1-ylamino)nonanoate (**30d**)

EDCI hydrochloride (54.0 mg, 0.28 mmol, 1.20 equiv.) and DIPEA (76.0 mg, 0.591 mmol, 2.50 equiv.) were added to a solution of *Z*-amine **19** (40.0 mg, 0.243 mmol, 1.00 equiv.) and methyl 8-carboxyoctanoate (47.0 mg, 0.24 mmol, 1.00 equiv.) in dry DMF (2.00 mL). The solution was stirred overnight at rt. The solvent was removed in vacuo and the residue was purified by flash chromatography on silica gel (heptane:EtOAc 1:1) to afford **30d** (31.0 mg, 0.09 mmol, 36%) as a crystalline solid. R_f_ 0.33 (heptane:EtOAc 1:1); mp.: 54–55 °C; ^1^H NMR (400 MHz, CDCl_3_) δ 5.61–5.42 (m, 2H), 5.43–5.29 (m, 1H), 3.91–3.83 (m, 2H), 3.64 (s, 3H), 2.28 (t, *J* = 7.5 Hz, 2H), 2.14 (t, *J* = 7.6 Hz, 2H), 2.05 (q, *J* = 7.3 Hz, 2H), 1.70–1.49 (m, 4H), 1.36–1.13 (m, 19H), 0.86 (t, *J* = 6.8 Hz, 3H); ^13^C NMR (101 MHz, CDCl_3_) δ 174.4, 173.0, 134.1, 125.1, 51.6, 36.8, 36.8, 34.1, 32.0, 29.6, 29.6, 29.4, 29.4, 29.2, 29.0, 29.0, 27.5, 25.8, 25.0, 22.8, 14.2; HRMS (ESI/Q-TOF) *m*/*z*: [M+Na]^+^ calcd. for C_21_H_39_NO_3_Na 376.2822; found 376.2822.

##### 8-Oxo-8-(undecylamino)octanoic Acid (**10a**)

Prepared by hydrolyzing **30a** using *General procedure C*. After cooling to 0 °C, the solution was filtered and washed with cold water. The solids were taken up in dilute HCl, extracted with EtOAc (3 × 30 mL) and dried over MgSO_4_. Concentration in vacuo afforded **10a** (117 mg, 0.36 mmol, 68%) as a white solid. mp. 104–105 °C; ^1^H NMR (400 MHz, CD_3_OD) δ 3.15 (t, *J* = 7.0 Hz, 2H), 2.28 (t, *J* = 7.4 Hz, 2H), 2.17 (t, *J* = 7.5 Hz, 2H), 1.67–1.55 (m, 4H), 1.55–1.43 (m, 2H), 1.40–1.22 (m, 20H), 0.90 (t, *J* = 7.1 Hz, 3H); ^13^C NMR (101 MHz, CD_3_OD) δ 176.9, 175.5, 48.1, 47.9, 47.7, 39.7, 36.4, 34.2, 32.4, 30.1, 30.1, 30.1, 29.8, 29.8, 29.3, 29.2, 27.4, 26.3, 25.3, 23.1, 13.8; HRMS (ESI/Q-TOF) *m*/*z*: [M+Na]^+^ calcd. for C_19_H_37_NO_3_Na 350.2666; found 350.2665.

##### 8-(Dodecylamino)-8-oxooctanoic Acid (**10b**)

Prepared by hydrolyzing **30b** using *General procedure C*. After cooling to 0 °C, the solution was filtered and washed with cold water. The solids were taken up in dilute HCl, extracted with EtOAc (3 × 30 mL) and dried over MgSO_4_. Concentration in vacuo afforded **10b** (201 mg, 0.59 mmol, 86%) as a white solid. mp. 104–105 °C; ^1^H NMR (400 MHz, CD_3_OD) δ 7.92 (s, 1H), 3.20–3.11 (m, 2H), 2.28 (t, *J* = 7.4 Hz, 2H), 2.17 (t, *J* = 7.5 Hz, 2H), 1.67–1.55 (m, 4H), 1.55–1.43 (m, 2H), 1.39–1.23 (m, 22H), 0.90 (t, *J* = 7.0 Hz, 3H); ^13^C NMR (101 MHz, CD_3_OD) δ 176.9, 175.5, 39.7, 36.4, 34.2, 32.4, 30.1, 30.1, 30.1, 29.8, 29.8, 29.3, 29.2, 27.4, 26.3, 25.3, 23.1, 13.8; HRMS (ESI/Q-TOF) *m*/*z*: [M+Na]^+^ calcd. for C_20_H_39_NO_3_Na 364.2822; found 364.2822.

##### 9-Oxo-9-(undecylamino)nonanoic Acid (**10c**)

Prepared by hydrolyzing **30c** using *General procedure C*. The aqueous solution was acidified with HCl (conc.) to pH 2 and extracted with EtOAc (3 × 10 mL). The combined organic phases were washed with brine (25 mL), dried (Na_2_SO_4_), and the solvent was removed in vacuo. The crude product was recrystallized using MeOH as a solvent, and the crystals were washed with cold MeOH to afford the desired product **10c** (41.0 mg, 0.120 mmol, 85%) as a white crystalline solid. R_f_ 0.21 (DCM:MeOH 96:4; mp.: 96–98 °C; ^1^H NMR (400 MHz, CD_3_OD) δ 3.16 (t, *J* = 7.0 Hz, 2H), 2.29 (t, *J* = 7.4 Hz, 2H), 2.18 (t, *J* = 7.4 Hz, 2H), 1.72–1.55 (m, 4H), 1.55–1.44 (m, 2H), 1.41–1.23 (m, 22H), 0.91 (t, *J* = 6.8 Hz, 3H); ^13^C NMR (101 MHz, CD_3_OD) δ 177.7, 176.2, 40.3, 37.1, 35.0, 33.1, 30.7 (3 × C), 30.5, 30.4 (2 × C), 30.1 (3 × C), 28.0, 27.1, 26.1, 23.7, 14.4; HRMS (ESI/Q-TOF) *m*/*z*: [M+Na]^+^ calcd. for C_20_H_39_NO_3_Na 364.2822; found 364.2821.

##### (Z)-9-Oxo-9-(undec-2-en-1-ylamino)nonanoic Acid (**10d**)

Prepared by hydrolyzing **30d** using *General procedure C*. The reaction mixture was acidified with 1.0 M HCl (6.0 mL) to pH 2 and extracted with EtOAc (3 × 10 mL). The combined organic phases were dried (Na_2_SO_4_), and the solvent was removed in vacuo. The crude mixture was purified by flash chromatography on silica gel (DCM, 1:0 → 0:1 heptane:EtOAc) to afford the desired product **10d** (21.0 mg, 0.062 mmol, 84%) as a white crystalline solid. R_f_ 0.41 (EtOAc); mp. 73–74 °C; ^1^H NMR: (400 MHz, CDCl_3_) δ 5.62–5.51 (m, 1H), 5.47 (br. s, 1H), 5.43–5.31 (m, 1H), 3.88 (t, *J* = 6.3 Hz, 2H), 2.33 (t, *J* = 7.5 Hz, 2H), 2.24–2.12 (m, 2H), 2.07 (q, *J* = 7.2 Hz, 2H), 1.69–1.55 (m, 4H), 1.38–1.17 (m, 18H), 0.87 (t, *J* = 6.8 Hz, 3H); ^13^C NMR (101 MHz, CDCl_3_) δ 179.0, 173.4, 134.2, 125.0, 36.9, 36.8, 34.1, 32.0, 29.6, 29.6, 29.4, 29.4, 29.1, 29.0, 29.0, 27.5, 25.8, 24.8, 22.8, 14.2; HRMS (ESI/Q-TOF) *m*/*z*: [M+Na]^+^ calcd. for C_20_H_37_NO_3_Na 362.2666; found 362.2665.

##### Measurement of Human sEH and mEH Inhibition

The inhibition potency of the compounds against the recombinant purified human and mouse sEH and human mEH were measured using sensitive fluorescent assays [51,52]. The enzymes were diluted to the proper buffer and aliquoted in black 96-well plates. The enzymes were incubated with the inhibitors (0.4 < [I] < 50,000 nM) for 5 min at 37 °C before the introduction of the reporting substrate. For the sEH, nonfluorescent cyano(6-methoxy-naphthalen-2-yl)methyl trans-[(3-phenyloxiran-2-yl)methyl] carbonate MNPC was used as the reporting substrate at a final concentration of 5 μM [51]. For the mEH, cyano(6-methoxy-naphthalen-2-yl)methyl glycidyl carbonate (MNGC) was used at a final concentration of 5 µM. The formation of the product (6-methoxynaphthaldehyde) was measured (λ_em_ = 330 nm, λ_ex_ = 465 nm) every 30 s for 10 min by a Molecular Device M-2 plate reader. All measurements were performed in triplicate. The inhibitory potency (IC_50_) was calculated by regression of at least four data points on both sides of the 50% mark.

### 3.3. In Vitro Cell Viability Assay

#### 3.3.1. Chemicals

Sorafenib was obtained from LC Laboratories (catalog no S-8502) (Woburn, MA, USA). For cell viability, the WST assay Cell Counting Kit 8 (WST-8/CCK8) (ab228554) from Abcam Limited was used.

#### 3.3.2. Methodology

Human renal mesangial cells (4200, ScienCell, Carlsbad, CA, USA) were cultured at 37 °C (in 5% CO2) in RPMI 1640 medium (Gibco™, LS11875093) containing 10% FBS, 100 U/mL of penicillin, and 0.1 mg/mL of streptomycin. The cells were subculture following the protocol of the manufacturer.

**WST assay:** To evaluate the effect of 8,9-EET analogs and sorafenib on the viability of human renal mesangial cells, a 96-well TPP plate is seeded with cells, which are then allowed to adhere. Over the course of 24 h, they undergo serum starvation before being subject to one of five treatments: Control (no treatment), Sorafenib (10 µM), Sorafenib plus 8,9-EET analogs (1 µM, 3 µM or 10 µM). After 48 h incubation, a WST assay [53] used to gain information on cell survival; we added 10 μL WST reagent per well and incubated for 2 h. WST-8 tetrazolium salt is reduced by cellular dehydrogenases to an orange formazan product that is soluble in tissue culture medium. The amount of formazan produced is directly proportional to the number of living cells and is measured by absorbance at 460 nm. The absorbance at 460 nm is determined using a Synergy 4 Microplate Reader.

## 4. Conclusions

A library of nineteen 8,9-EET analogs was synthesized and each compound evaluated for its ability to protect renal mesangial cells against sorafenib-induced toxicity and as human sEH and mEH inhibitors. All compounds feature amide-like substitutions of the epoxide group, which confers stability against degradation by sEH. In this preliminary structure–activity relationship (SAR) study, it was shown that the oxamide group could act as a good substitution for the epoxide group in 8,9-EET as nephroprotective agents. Amide substituents could result in moderately good nephroprotective effect, whereas urea-containing analogs were largely inactive. Oxamide analog **8b** was the most effective and provided good protective effect even at 1 µM, while not being an inhibitor of sEH. No correlation between the nephroprotective effect and sEH inhibition was found during this project. A more elaborative SAR of **8b** is warranted to further explore the structural requirements for the observed nephroprotective effect.

## Figures and Tables

**Table 1:** Cell viability of human renal mesangial cells measured by a WST assay (*N* = 4). *E_10µM_*: median increase in cell viability in percentage points upon treatment with EET analog (10 µM) relative to sorafenib (10 µM) only. *E_1µM_*: EET-analog (1 µM). Missing entries denote insignificant (*p* > 0.05, Student’s *t*) increase in cell viability. *HsEH*: IC_50_ for human soluble epoxide hydrolase (HsEH).

Entry	Name	Structure	E_10µM_ [%]	E_1µM_ [%]	HsEH IC_50_ [µM]
1	**7a**	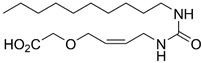	-	-	0.83
2	**7b**	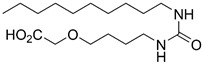	22	14	1.8
3	**7c**	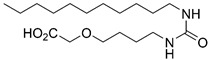	-	-	2.1
4	**7d**	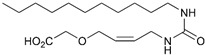	-	-	2.5
5	**7e**	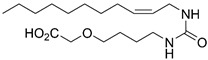	-	-	1.6
6	**7f**	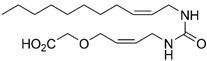	-	-	2.6
7	**8a**	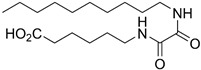	24	13	51
8	**8b**	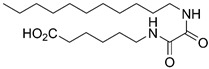	64	51	>100
9	**8c**	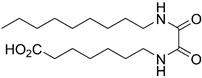	24	-	64
10	**8d**	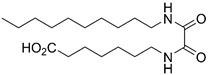	18	-	49
11	**8e**	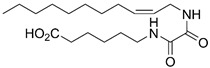	6	-	19
12	**9a**	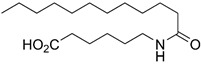	-	-	9.6
13	**9b**	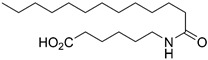	-	-	6.4
14	**9c**	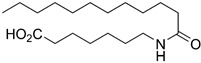	4	-	2.3
15	**9d**	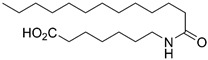	19	-	2.4
16	**10a**	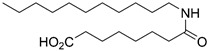	16	-	3.5
17	**10b**	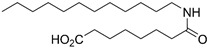	30	-	2.7
18	**10c**	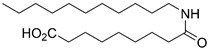	-	-	0.90
19	**10d**	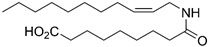	-	-	0.82

## Data Availability

The data supporting this article have been included as part of the ESI in Supplementary Materials.

## Supplementary Materials

The following supporting information can be downloaded at: https://www.mdpi.com/article/10.3390/molecules30071445/s1, Figures S1–S121: ^1^H NMR, ^13^C NMR spectra and all WST graphs.
