# Supplementary material for: Synthetic Epoxyeicosatrienoic Acid Mimics Protect Mesangial Cells from Sorafenib-Induced Cell Death
Source: Molecules. 2025 Mar 24;30(7):1445. doi: 10.3390/molecules30071445 (PMC11990158; doi:10.3390/molecules30071445)
Supplement: Supplementary file 1 [file molecules-30-01445-s001.zip › molecules-3466861-supplementary.pdf]

Supporting information
Synthetic Epoxyeicosatrienoic Acid Mimics Protect Mesangial Cells from Sorafenib-Induced Cell Death

Marcus de Bourg 1, Abhishek Mishra 2, Rawand S. Mohammad 1, Christophe Morisseau 3, Bruce D. Hammock 3, John D. Imig 2 and Anders Vik 1,*

1 Department of Pharmacy, Section for Pharmaceutical Chemistry, University of Oslo, N-0316 Oslo, Norway;

2 Department of Pharmaceutical Sciences, College of Pharmacy, University of Arkansas for Medical Sciences, Little Rock, AR 72205, USA

3 Department of Entomology and Nematology and UC Davis Comprehensive Cancer Center, University of California Davis, Davis, CA 95616, USA

* Correspondence: anders.vik@farmasi.uio.no

Contents

Table of figures 1

^{1}H- and ^{13}C-NMR spectra 5

Cell viability assays (WST) 103

Table of figures

Figure S-1 ^{1}H NMR spectrum of 12.. 5
Figure S-2 ^{13}C NMR spectrum of 12.. 6
Figure S-3 ^{1}H NMR spectrum of 13a.. 7
Figure S-4 ^{13}C NMR spectrum of **13a**.. 7
Figure S-5 ^{1}H NMR spectrum of **15**.. 8
Figure S-6 ^{13}C NMR spectrum of **15**.. 9
Figure S-7 ^{1}H NMR spectrum of **17**.. 9
Figure S-8 ^{13}C NMR spectrum of **17**.. 10
Figure S-9 ^{1}H NMR spectrum of **18**.. 11
Figure S-10 ^{13}C NMR spectrum of **18**.. 12
Figure S-11 ^{1}H NMR spectrum of **19**.. 13
Figure S-12 ^{13}C NMR spectrum of **19**.. 14
Figure S-13 ^{1}H NMR spectrum of **21a**.. 15
Figure S-14 ^{13}C NMR spectrum of **21a**.. 16
Figure S-15 ^{1}H NMR spectrum of **21b**. 17
Figure S-16 ^{13}C NMR spectrum of **21b**. 18
Figure S-17 ^{1}H NMR spectrum of **21c**.. 19
Figure S-18 ^{13}C NMR spectrum of **21c**.. 20
Figure S-19 ^{1}H NMR spectrum of **21d**. 21
Figure S-20 ^{13}C NMR spectrum of **21d**. 22
Figure S-21 ^{1}H NMR spectrum of **21e**.. 22
Figure S-22 ^{13}C NMR spectrum of **21e**.. 23
Figure S-23 ^{1}H NMR spectrum of **21f**. 24
Figure S-24 ^{13}C NMR spectrum of **21f**. 25
Figure S-25 ^{1}H NMR spectrum of **22a**.. 26
Figure S-26 ^{13}C NMR spectrum of **22a**.. 27

Figure S-27 ^{1}H NMR spectrum of **22b**. 28
Figure S-28 ^{13}C NMR spectrum of **22b**. 29
Figure S-29 ^{1}H NMR spectrum of **7c**. 30
Figure S-30 ^{13}C NMR spectrum of **7c**. 31
Figure S-31 ^{1}H NMR spectrum of **22c**. 32
Figure S-32 ^{13}C NMR spectrum of **22c**. 33
Figure S-33 ^{1}H NMR spectrum of **22d**. 33
Figure S-34 ^{13}C NMR spectrum of **22d**. 34
Figure S-35 ^{1}H NMR spectrum of **7e**. 35
Figure S-36 ^{13}C NMR spectrum of **7e**. 36
Figure S-37 ^{1}H NMR spectrum of **22e**. 37
Figure S-38 ^{13}C NMR spectrum of **22e**. 38
Figure S-39 ^{1}H NMR spectrum of **7a**. 39
Figure S-40 ^{13}C NMR spectrum of **7a**. 40
Figure S-41 ^{1}H NMR spectrum of **7b**. 41
Figure S-42 ^{13}C NMR spectrum of **7b**. 42
Figure S-43 ^{1}H NMR spectrum of **7d**. 43
Figure S-44 ^{13}C NMR spectrum of **7d**. 44
Figure S-45 ^{1}H NMR spectrum of **7f**. 45
Figure S-46 ^{13}C NMR spectrum of **7f**. 46
Figure S-47 ^{1}H NMR spectrum of **25a**. 47
Figure S-48 ^{13}C NMR spectrum of **25a**. 48
Figure S-49 ^{1}H NMR spectrum of **25b**. 49
Figure S-50 ^{13}C NMR spectrum of **25b**. 50
Figure S-51 ^{1}H NMR spectrum of **26a**. 51
Figure S-52 ^{13}C NMR spectrum of **26a**. 52
Figure S-53 ^{1}H NMR spectrum of **26b**. 53
Figure S-54 ^{13}C NMR spectrum of **26b**. 54
Figure S-55 ^{1}H NMR spectrum of **26c**. 55
Figure S-56 ^{13}C NMR spectrum of **26c**. 56
Figure S-57 ^{1}H NMR spectrum of **26d**. 57
Figure S-58 ^{13}C NMR spectrum of **26d**. 58
Figure S-59 ^{1}H NMR spectrum of **26e**. 59
Figure S-60 ^{13}C NMR spectrum of **26e**. 60
Figure S-61 ^{1}H NMR spectrum of **8a**. 61
Figure S-62 ^{13}C NMR spectrum of **8a**. 62
Figure S-63 ^{1}H NMR spectrum of **8b**. 63
Figure S-64 ^{13}C NMR spectrum of **8b**. 64
Figure S-65 ^{1}H NMR spectrum of **8c**. 65
Figure S-66 ^{13}C NMR spectrum of **8c**. 66
Figure S-67 ^{1}H NMR spectrum of **8d**. 67
Figure S-68 ^{13}C NMR spectrum of **8d**. 68
Figure S-69 ^{1}H NMR spectrum of **8e**. 69
Figure S-70 ^{13}C NMR spectrum of **8e**. 70
Figure S-71 ^{1}H NMR spectrum of **28a**. 71
Figure S-72 ^{13}C NMR spectrum of **28a**. 72
Figure S-73 ^{1}H NMR spectrum of **28b**. 73
Figure S-74 ^{13}C NMR spectrum of **28b**. 74
Figure S-75 ^{1}H NMR spectrum of **28c**. 75
Figure S-76 ^{13}C NMR spectrum of **28c**. 76
Figure S-77 ^{1}H NMR spectrum of **28d**. 77

Figure S-78 ^{13}C NMR spectrum of **28d**. 78
Figure S-79 ^{1}H NMR spectrum of **9a**........ 79
Figure S-80 ^{13}C NMR spectrum of **9a**........ 80
Figure S-81 ^{1}H NMR spectrum of **9b**. 81
Figure S-82 ^{13}C NMR spectrum of **9b**. 82
Figure S-83 ^{1}H NMR spectrum of **9c**........ 83
Figure S-84 ^{13}C NMR spectrum of **9c**........ 84
Figure S-85 ^{1}H NMR spectrum of **9d**. 85
Figure S-86 ^{13}C NMR spectrum of **9d**. 86
Figure S-87 ^{1}H NMR spectrum of **30a**........ 87
Figure S-88 ^{13}C NMR spectrum of **30a**........ 88
Figure S-89 ^{1}H NMR spectrum of **30b**. 89
Figure S-90 ^{13}C NMR spectrum of **30b**. 90
Figure S-91 ^{1}H NMR spectrum of **30c**........ 91
Figure S-92 ^{13}C NMR spectrum of **30c**........ 92
Figure S-93 ^{1}H NMR spectrum of **30d**. 93
Figure S-94 ^{13}C NMR spectrum of **30d**. 94
Figure S-95 ^{1}H NMR spectrum of **10a**........ 95
Figure S-96 ^{13}C NMR spectrum of **10a**........ 96
Figure S-97 ^{1}H NMR spectrum of **10b**. 97
Figure S-98 ^{13}C NMR spectrum of **10b**. 98
Figure S-99 ^{1}H NMR spectrum of **10c**........ 99
Figure S-100 ^{13}C NMR spectrum of **10c**........ 100
Figure S-101 ^{1}H NMR spectrum of **10d**. 101
Figure S-102 ^{13}C NMR spectrum of **10d**. 102
Figure S-103 WST-8 assay of human renal mesangial cells treated with sorafenib and 8,9-EET mimics at indicated concentrations after 48 h incubation, as percentages relative to control. Bars represent mean ± SEM (n = 4). 103
Figure S-104 WST-8 assay of human renal mesangial cells treated with sorafenib and 8,9-EET mimics at indicated concentrations after 48 h incubation, as percentages relative to control. Bars represent mean ± SEM (n = 4). 104
Figure S-105 WST-8 assay of human renal mesangial cells treated with sorafenib and 8,9-EET mimics at indicated concentrations after 48 h incubation, as percentages relative to control. Bars represent mean ± SEM (n = 4). 105
Figure S-106 WST-8 assay of human renal mesangial cells treated with sorafenib and 8,9-EET mimics at indicated concentrations after 48 h incubation, as percentages relative to control. Bars represent mean ± SEM (n = 4). 106
Figure S-107 WST-8 assay of human renal mesangial cells treated with sorafenib and 8,9-EET mimics at indicated concentrations after 48 h incubation, as percentages relative to control. Bars represent mean ± SEM (n = 4). 107
Figure S-108 WST-8 assay of human renal mesangial cells treated with sorafenib and 8,9-EET mimics at indicated concentrations after 48 h incubation, as percentages relative to control. Bars represent mean ± SEM (n = 4). 108
Figure S-109 WST-8 assay of human renal mesangial cells treated with sorafenib and 8,9-EET mimics at indicated concentrations after 48 h incubation, as percentages relative to control. Bars represent mean ± SEM (n = 4). 109
Figure S-110 WST-8 assay of human renal mesangial cells treated with sorafenib and 8,9-EET mimics at indicated concentrations after 48 h incubation, as percentages relative to control. Bars represent mean ± SEM (n = 4). 110

Figure S-111 WST-8 assay of human renal mesangial cells treated with sorafenib and 8,9-EET mimics at indicated concentrations after 48 h incubation, as percentages relative to control. Bars represent mean ± SEM (n = 4). 111
Figure S-112 WST-8 assay of human renal mesangial cells treated with sorafenib and 8,9-EET mimics at indicated concentrations after 48 h incubation, as percentages relative to control. Bars represent mean ± SEM (n = 4). 112
Figure S-113 WST-8 assay of human renal mesangial cells treated with sorafenib and 8,9-EET mimics at indicated concentrations after 48 h incubation, as percentages relative to control. Bars represent mean ± SEM (n = 4). 113
Figure S-114 WST-8 assay of human renal mesangial cells treated with sorafenib and 8,9-EET mimics at indicated concentrations after 48 h incubation, as percentages relative to control. Bars represent mean ± SEM (n = 4). 114
Figure S-115 WST-8 assay of human renal mesangial cells treated with sorafenib and 8,9-EET mimics at indicated concentrations after 48 h incubation, as percentages relative to control. Bars represent mean ± SEM (n = 4). 115
Figure S-116 WST-8 assay of human renal mesangial cells treated with sorafenib and 8,9-EET mimics at indicated concentrations after 48 h incubation, as percentages relative to control. Bars represent mean ± SEM (n = 4). 116
Figure S-117 WST-8 assay of human renal mesangial cells treated with sorafenib and 8,9-EET mimics at indicated concentrations after 48 h incubation, as percentages relative to control. Bars represent mean ± SEM (n = 4). 117
Figure S-118 WST-8 assay of human renal mesangial cells treated with sorafenib and 8,9-EET mimics at indicated concentrations after 48 h incubation, as percentages relative to control. Bars represent mean ± SEM (n = 4). 118
Figure S-119 WST-8 assay of human renal mesangial cells treated with sorafenib and 8,9-EET mimics at indicated concentrations after 48 h incubation, as percentages relative to control. Bars represent mean ± SEM (n = 4). 119
Figure S-120 WST-8 assay of human renal mesangial cells treated with sorafenib and 8,9-EET mimics at indicated concentrations after 48 h incubation, as percentages relative to control. Bars represent mean ± SEM (n = 4). 120
Figure S-121 WST-8 assay of human renal mesangial cells treated with sorafenib and 8,9-EET mimics at indicated concentrations after 48 h incubation, as percentages relative to control. Bars represent mean ± SEM (n = 4). 121

^{1}H- and ^{13}C-NMR spectra

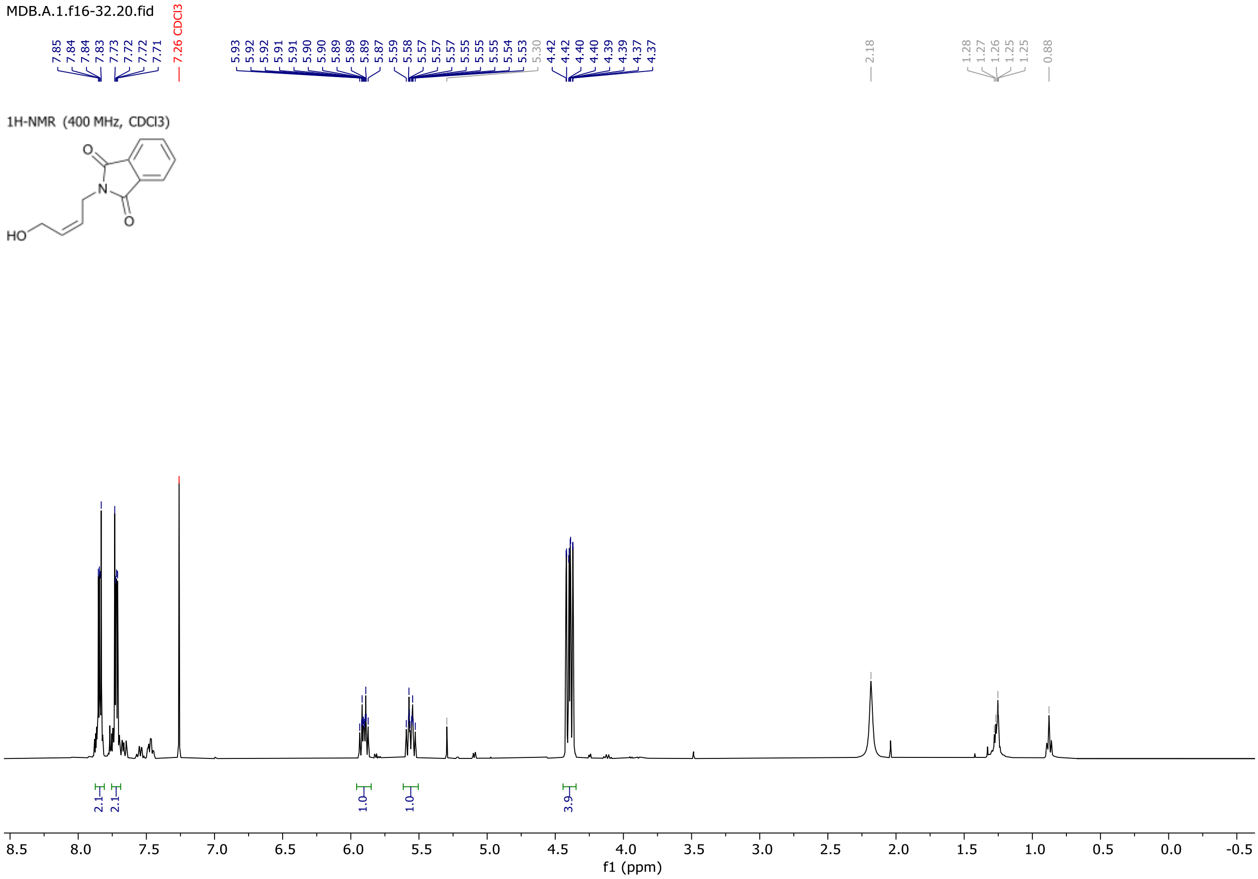

Figure S-1 ^{1}H NMR spectrum of 12.

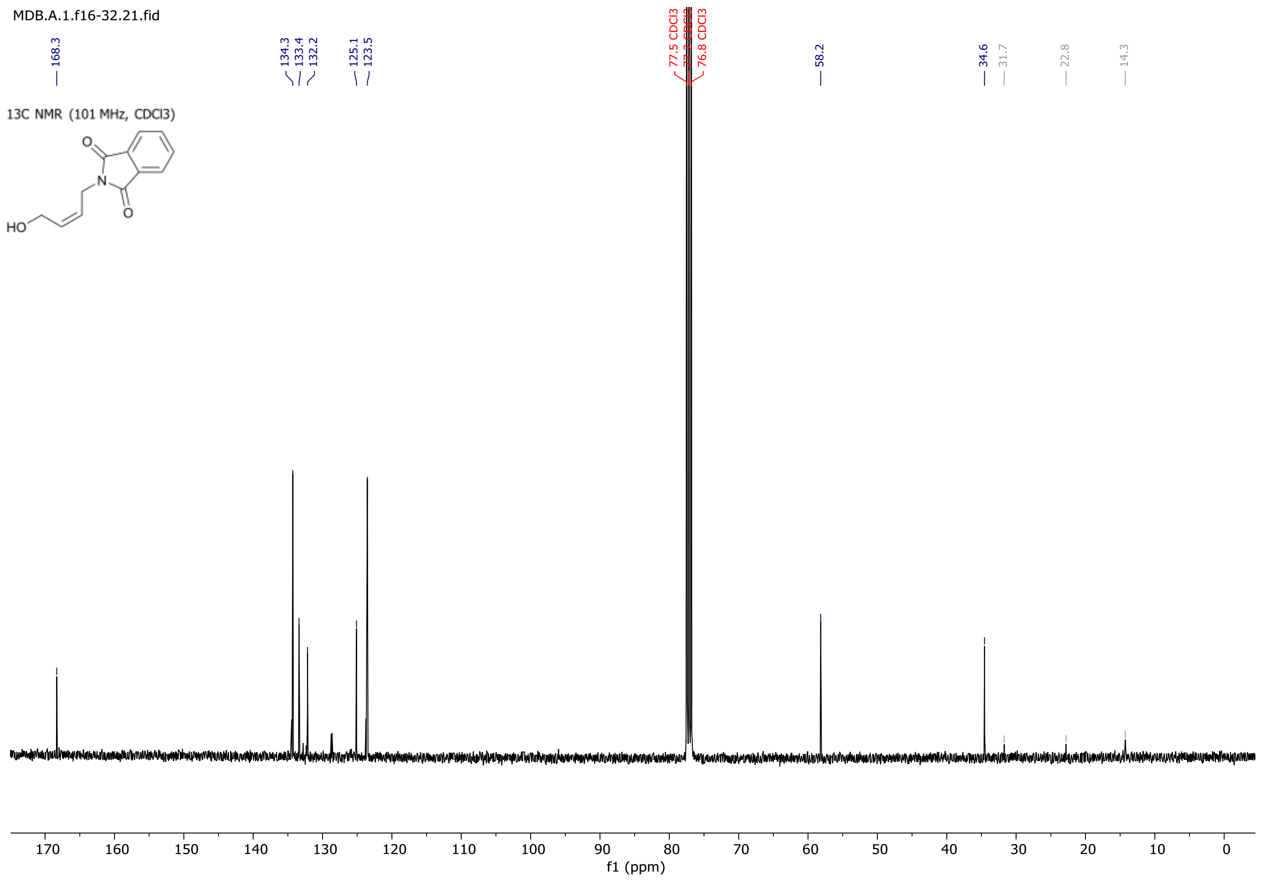

Figure S-2 ^{13}C NMR spectrum of 12.

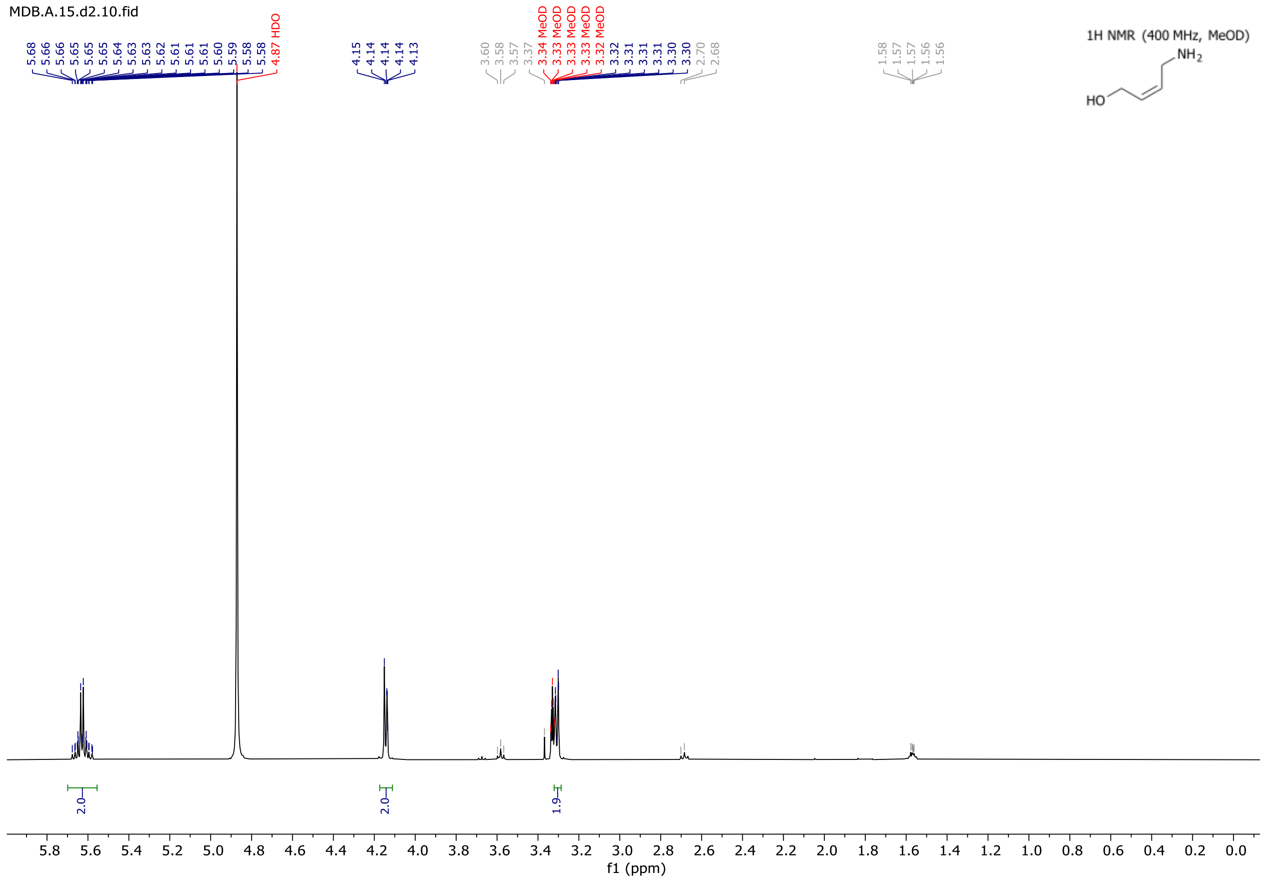

Figure S-3 ^{1}H NMR spectrum of 13a.

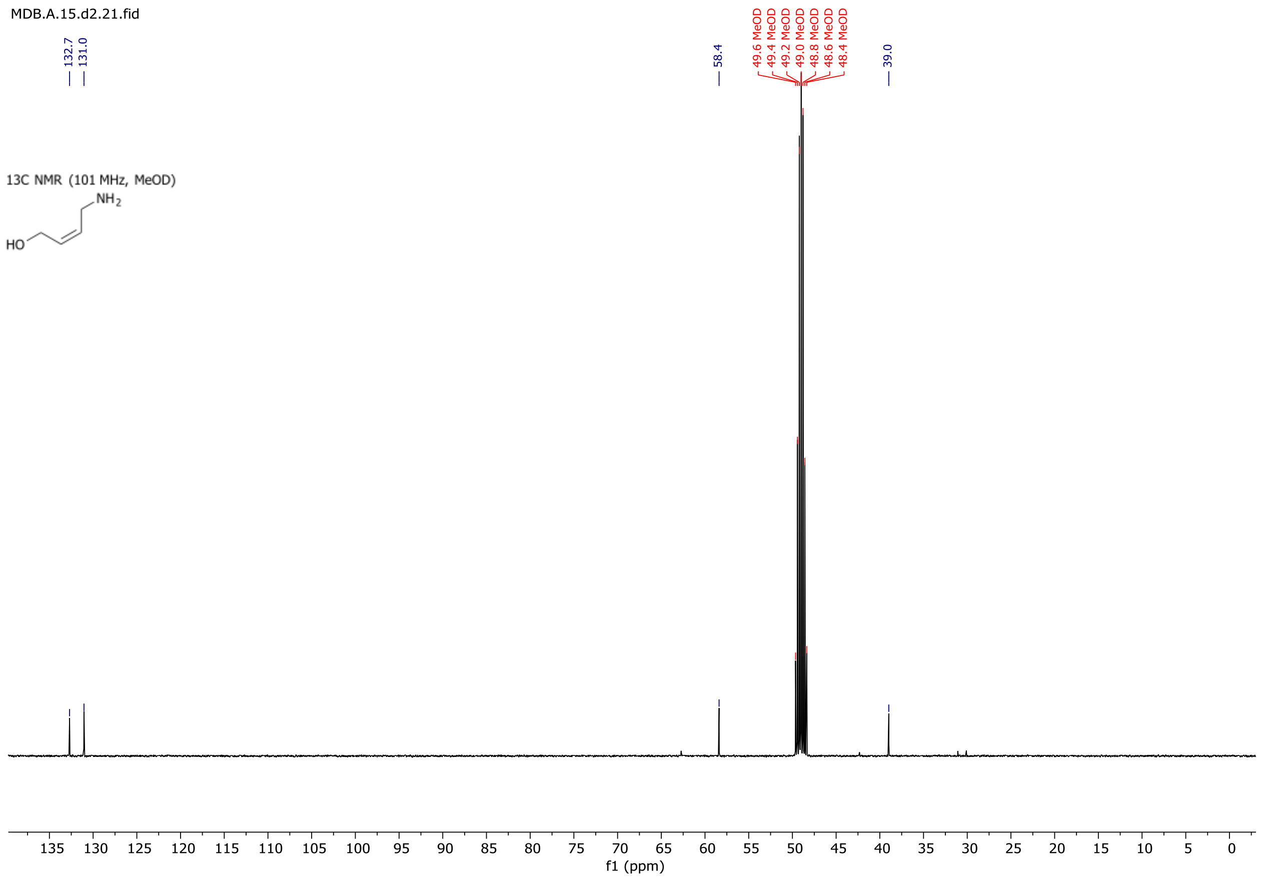

Figure S-4 ^{13}C NMR spectrum of **13a**.

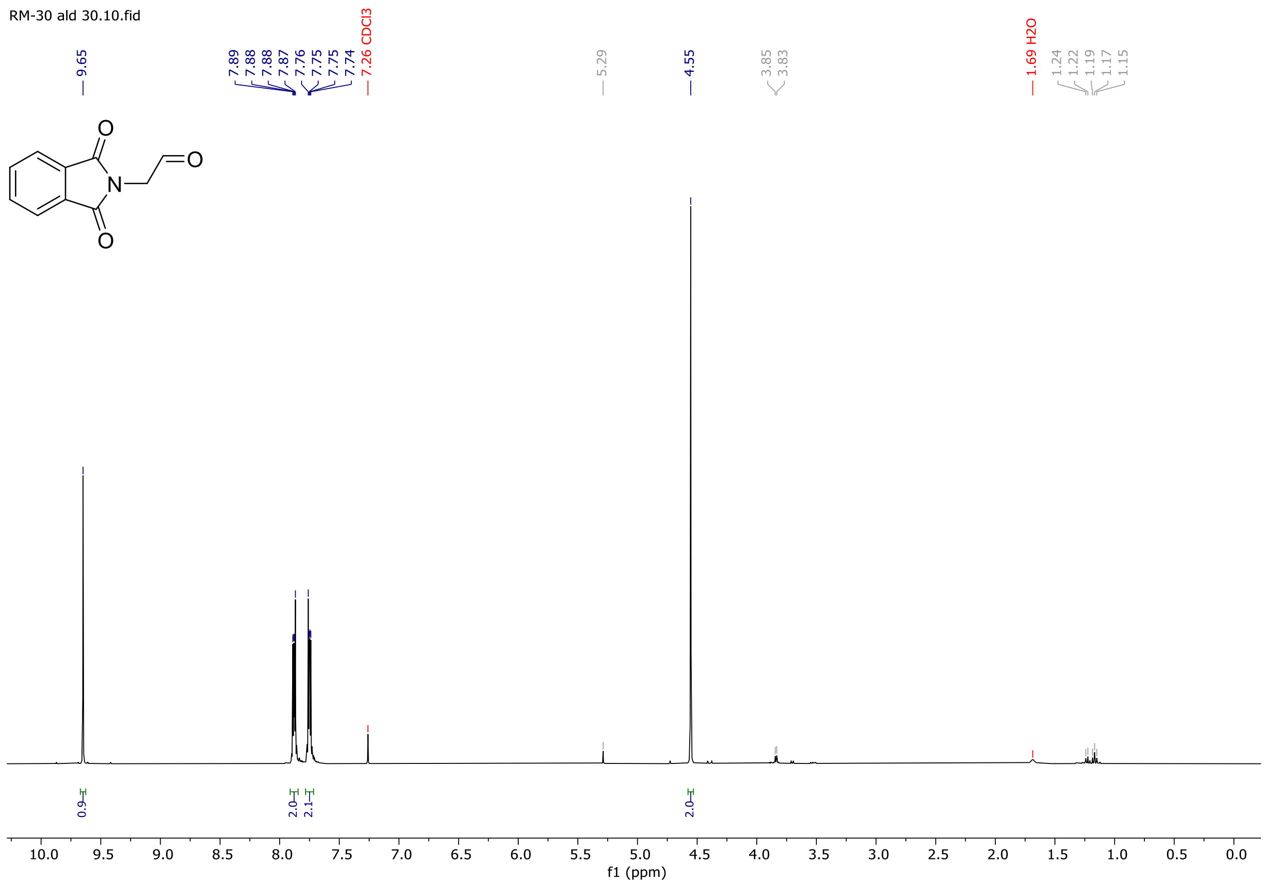

Figure S-5 ^{1}H NMR spectrum of **15**.

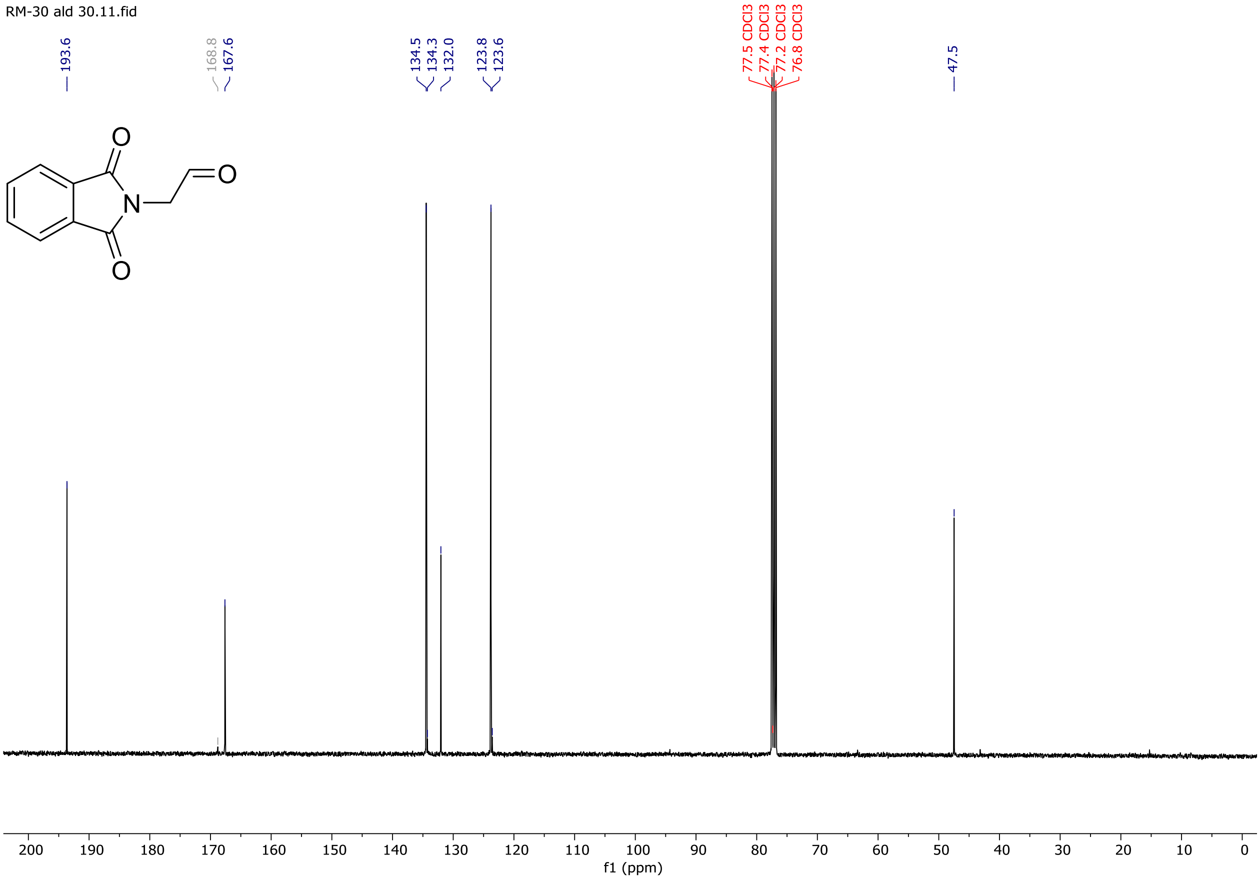

Figure S-6 ^{13}C NMR spectrum of **15**.

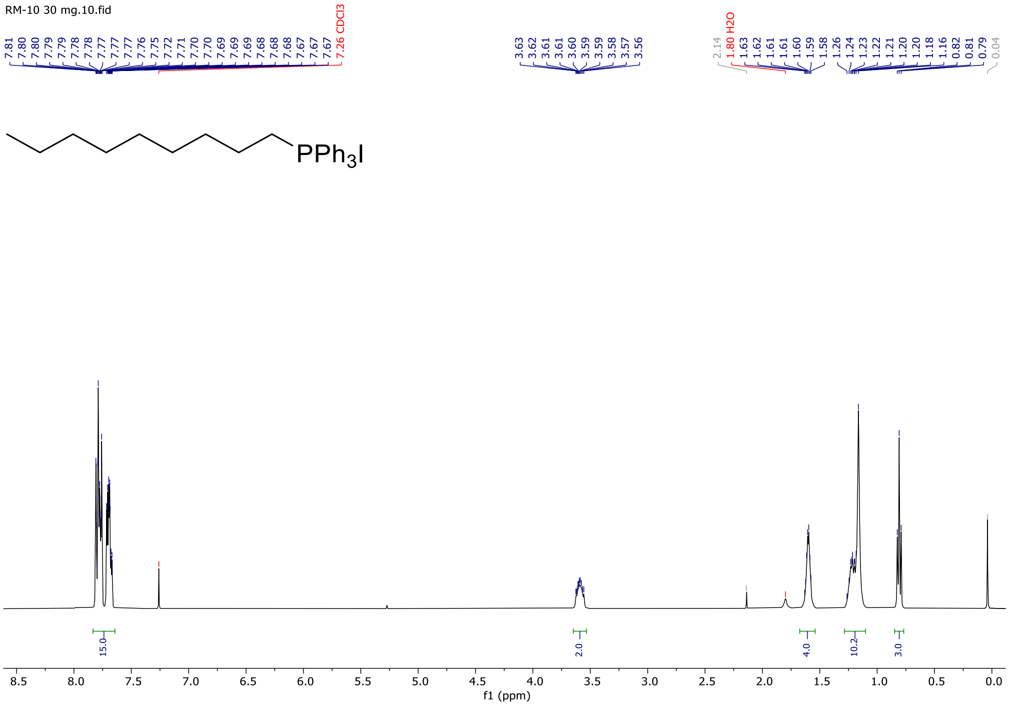

Figure S-7 ^{1}H NMR spectrum of **17**.

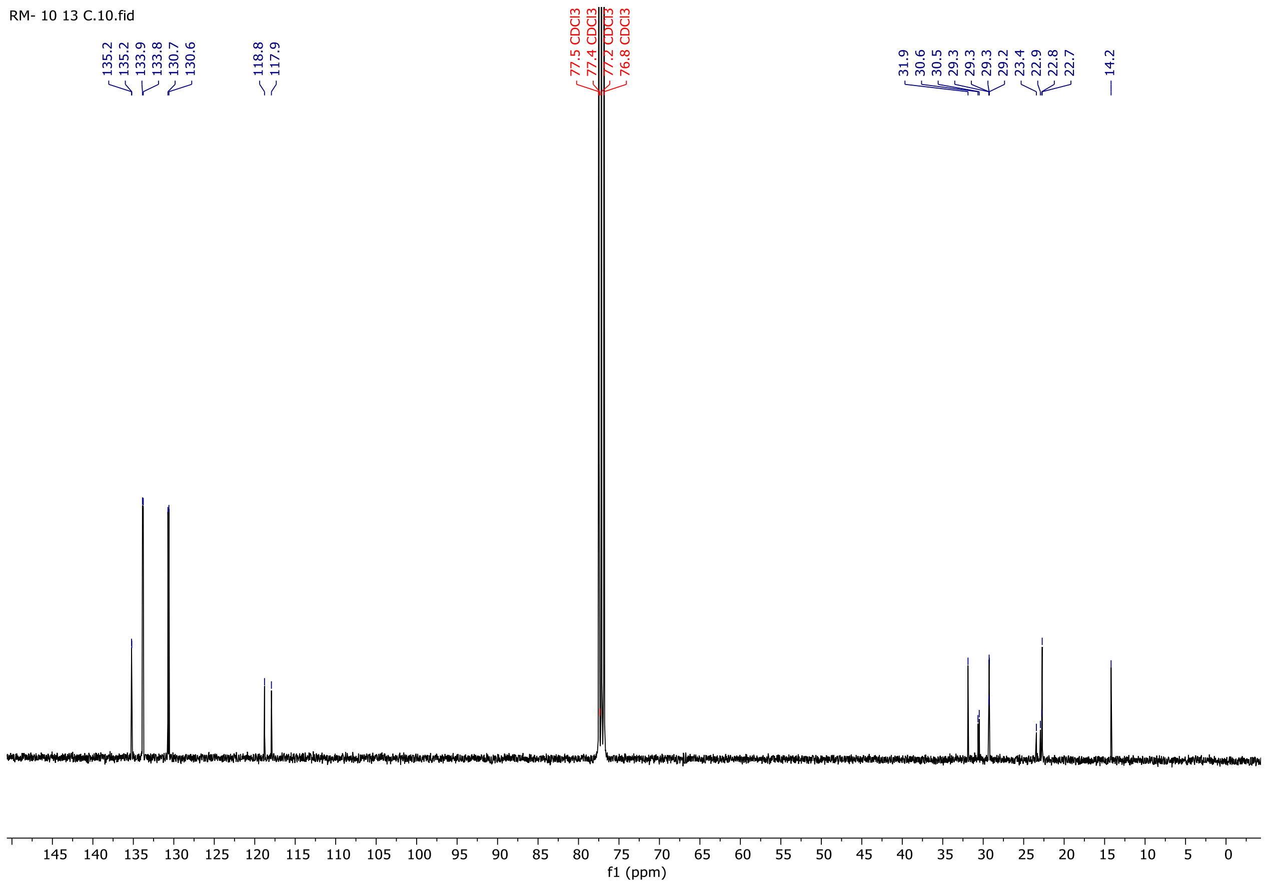

Figure S-8 ^{13}C NMR spectrum of **17**.

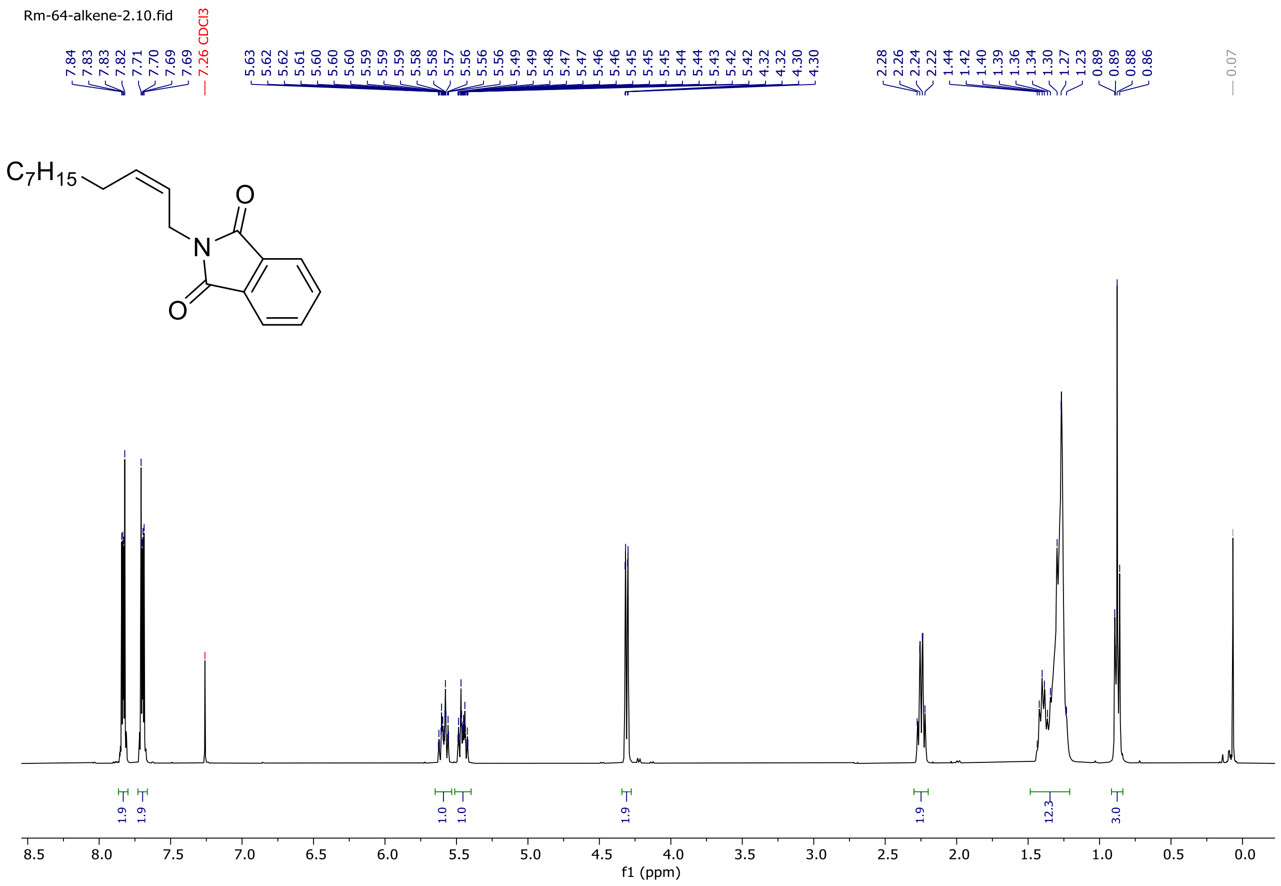

Figure S-9 ^{1}H NMR spectrum of **18**.

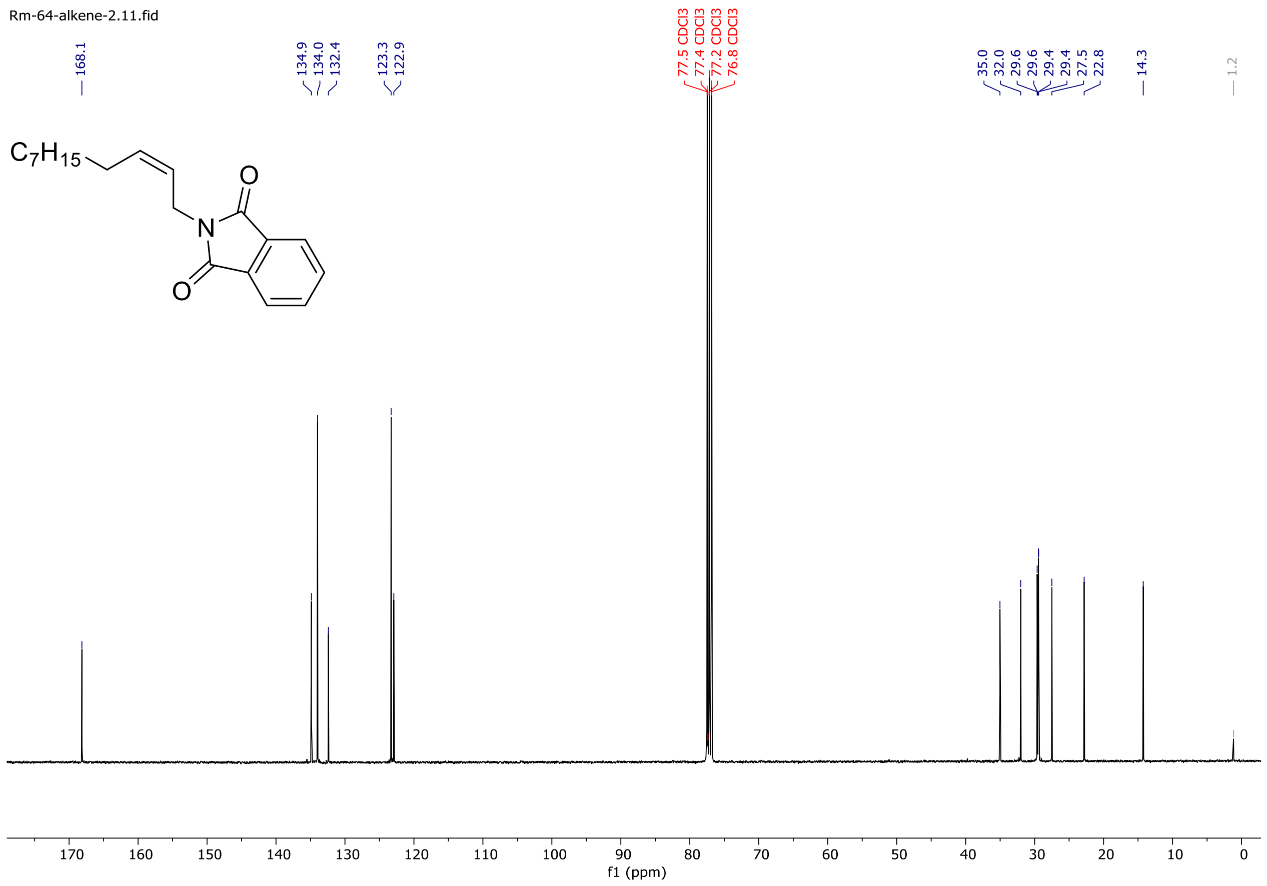

Figure S-10 ^{13}C NMR spectrum of **18**.

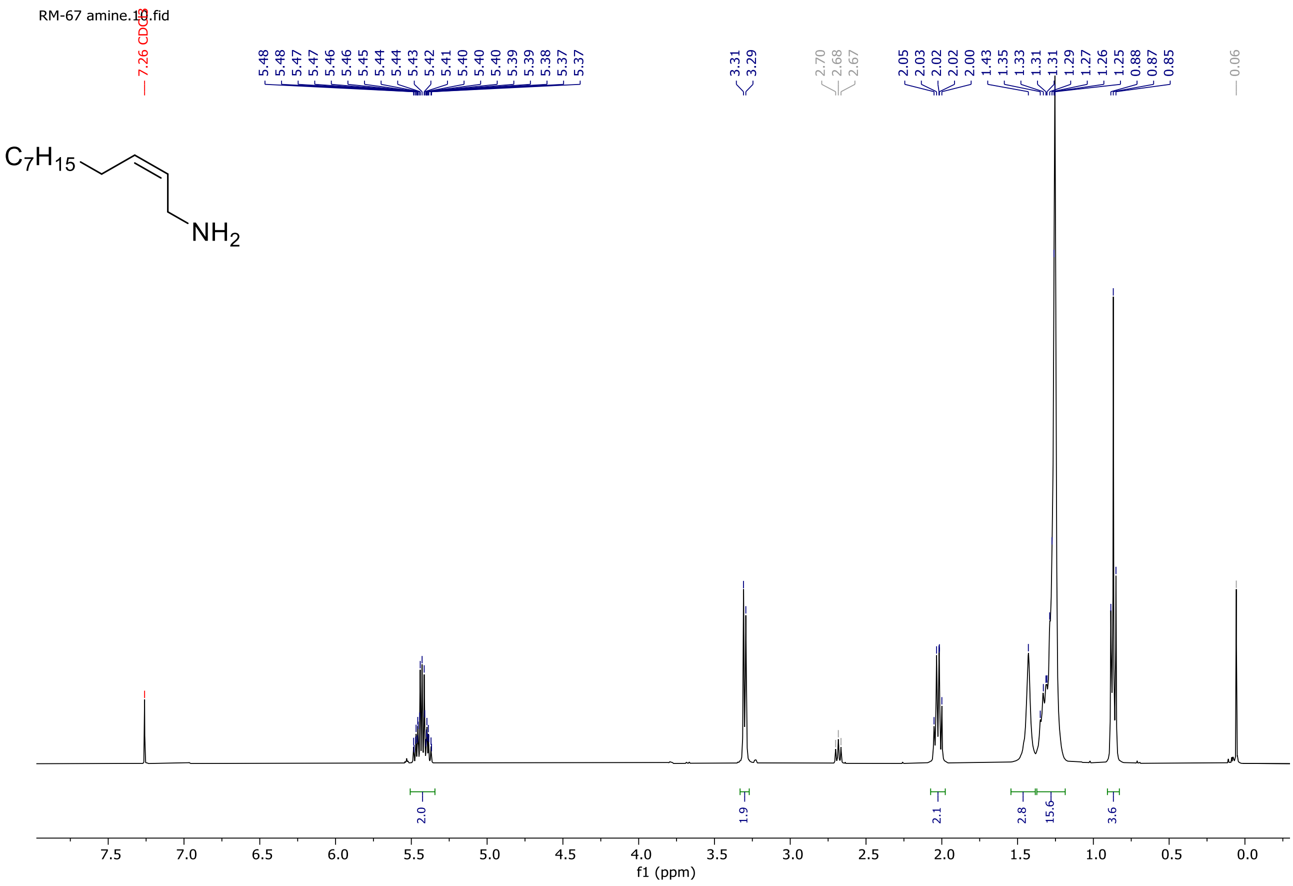

Figure S-11 ^{1}H NMR spectrum of **19**.

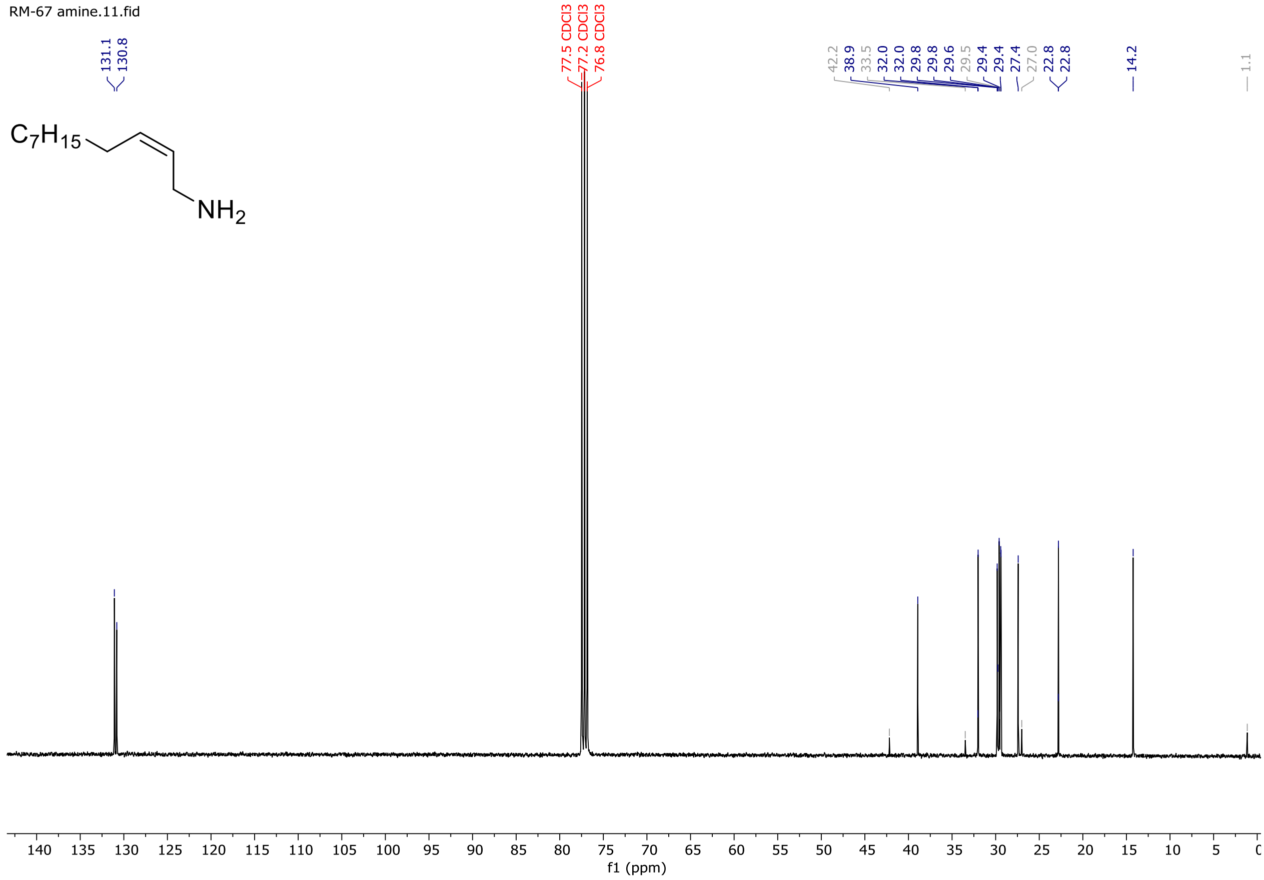

Figure S-12 ^{13}C NMR spectrum of **19**.

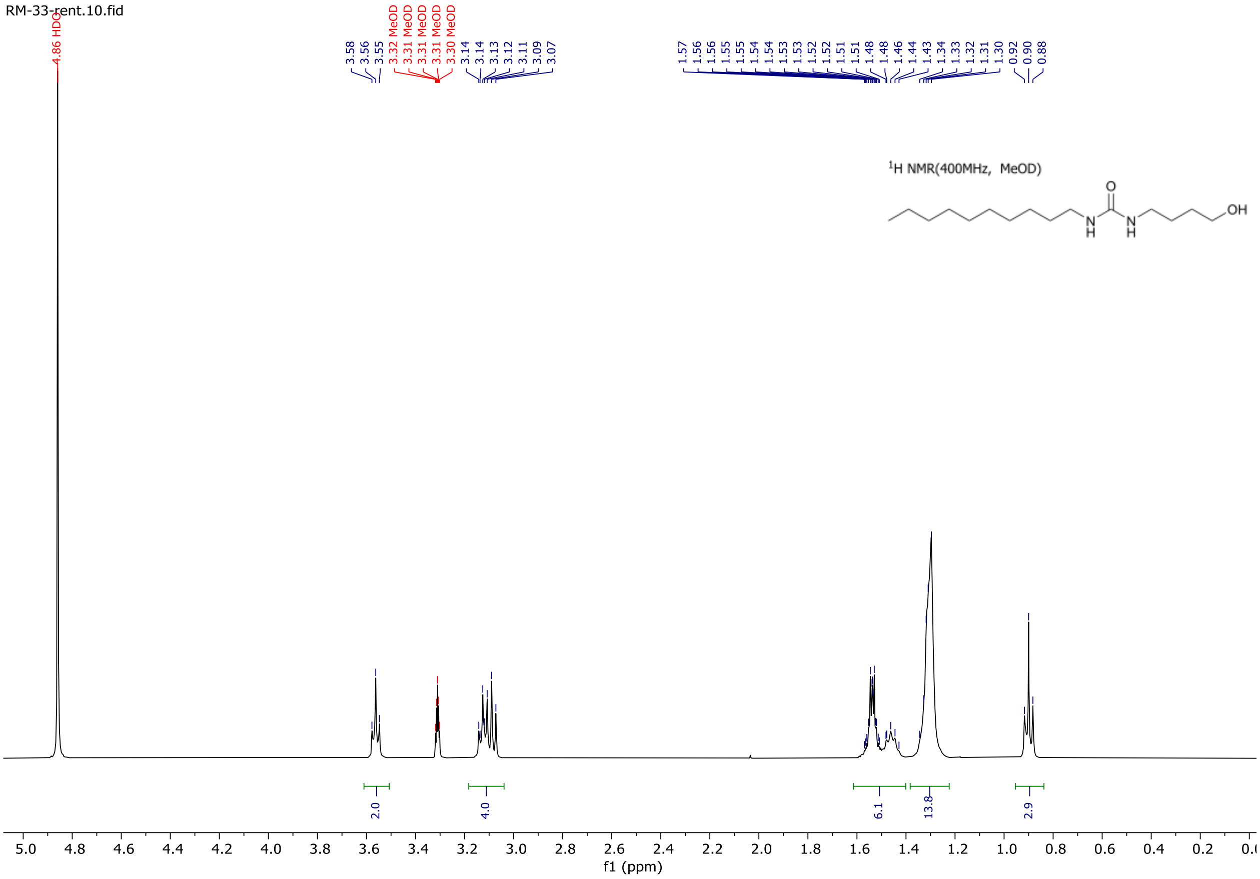

Figure S-13 ^{1}H NMR spectrum of **21a**.

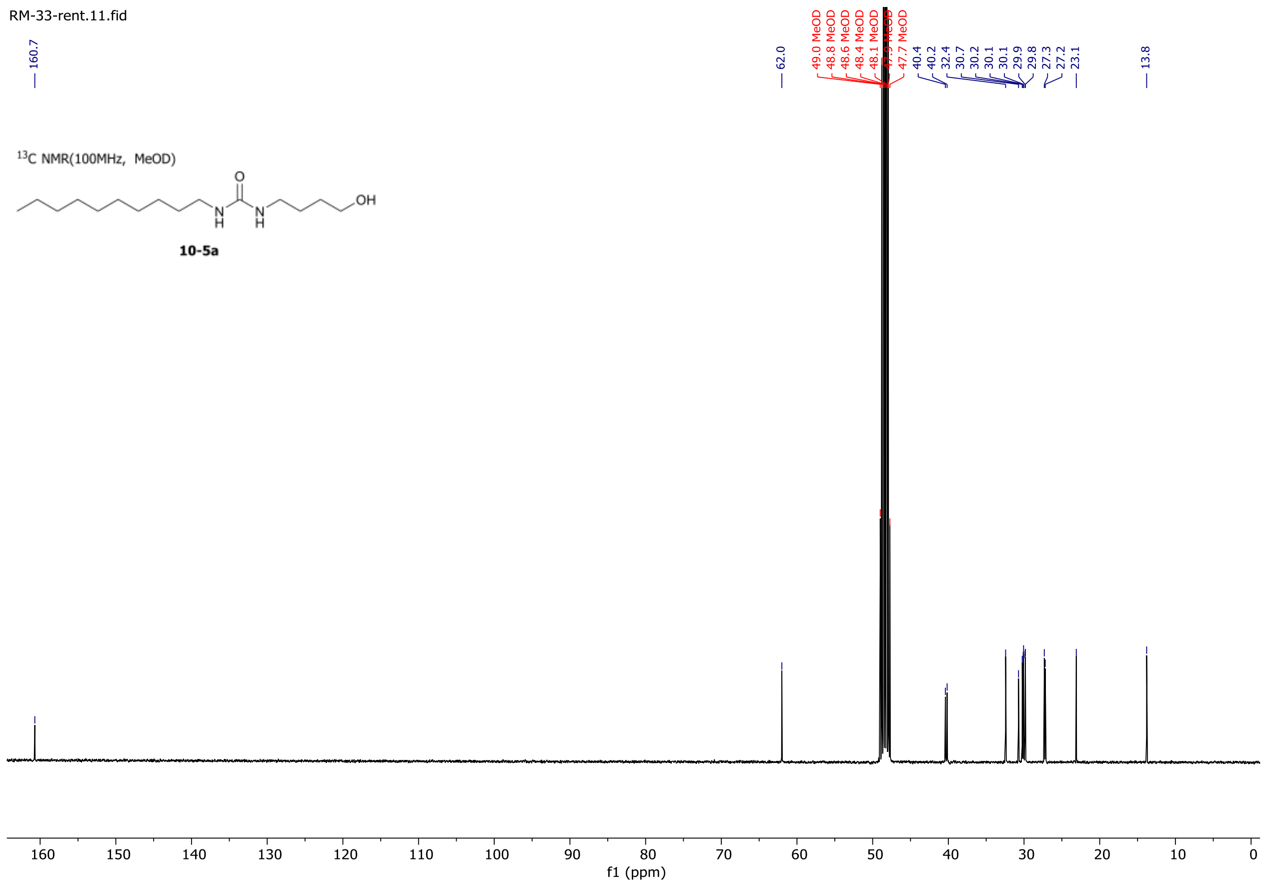

Figure S-14 ^{13}C NMR spectrum of **21a**.

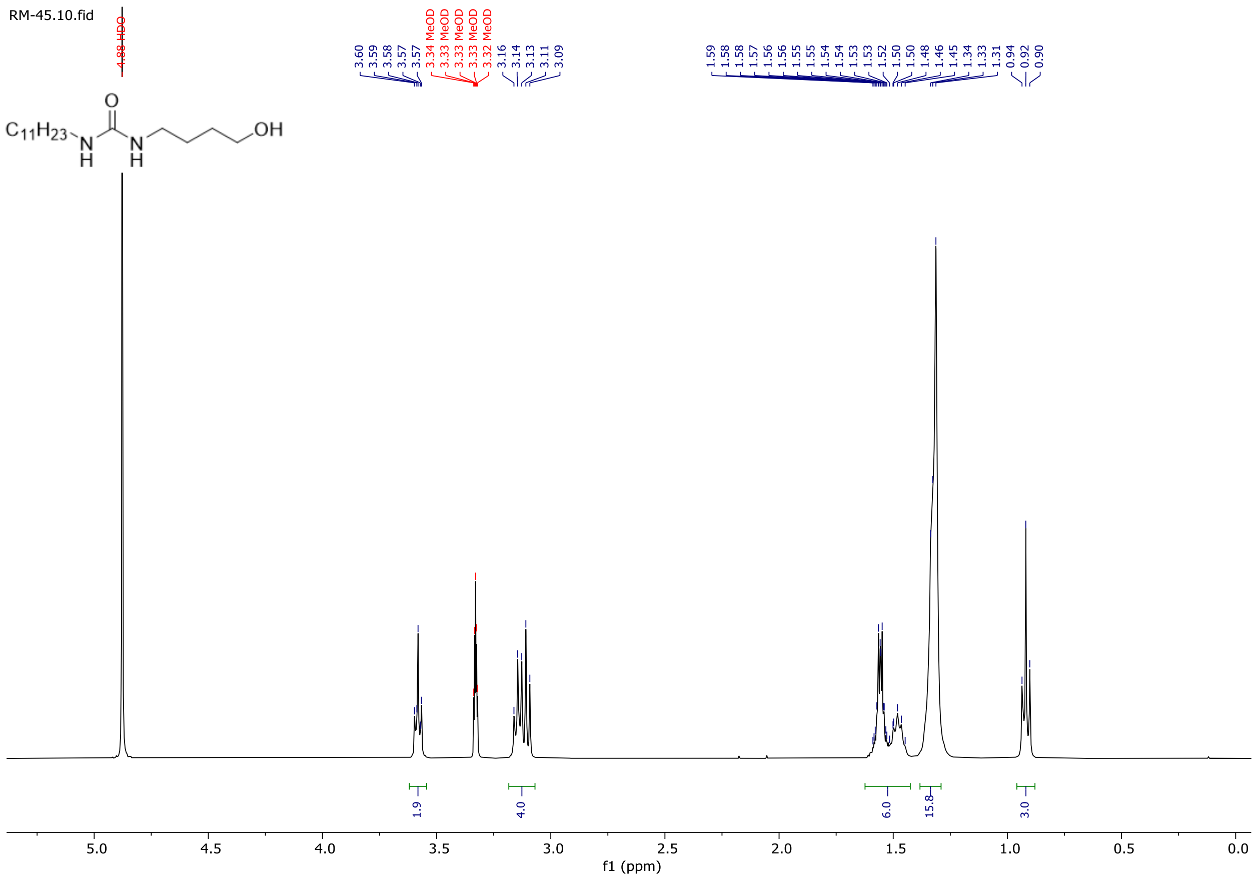

Figure S-15 ^{1}H NMR spectrum of **21b**.

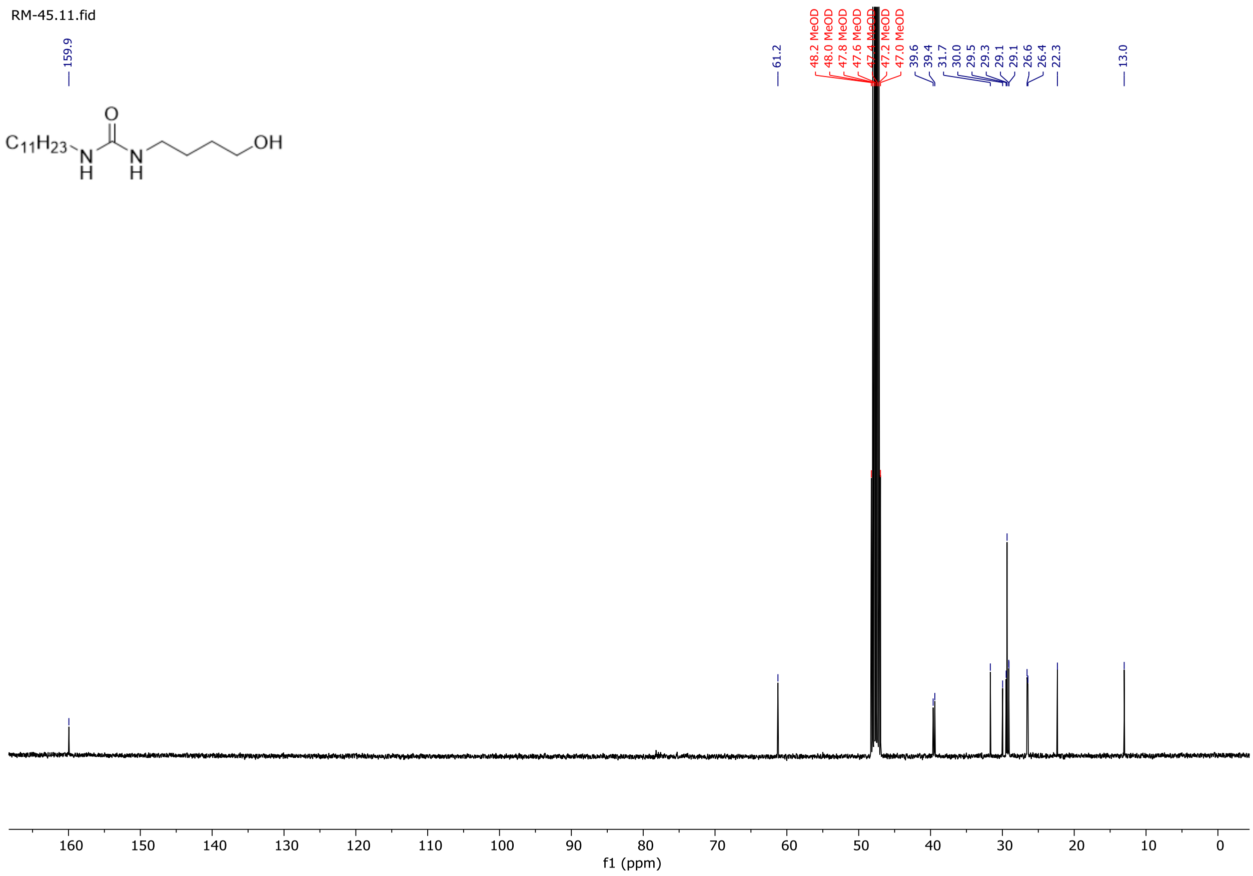

Figure S-16 ^{13}C NMR spectrum of **21b**.

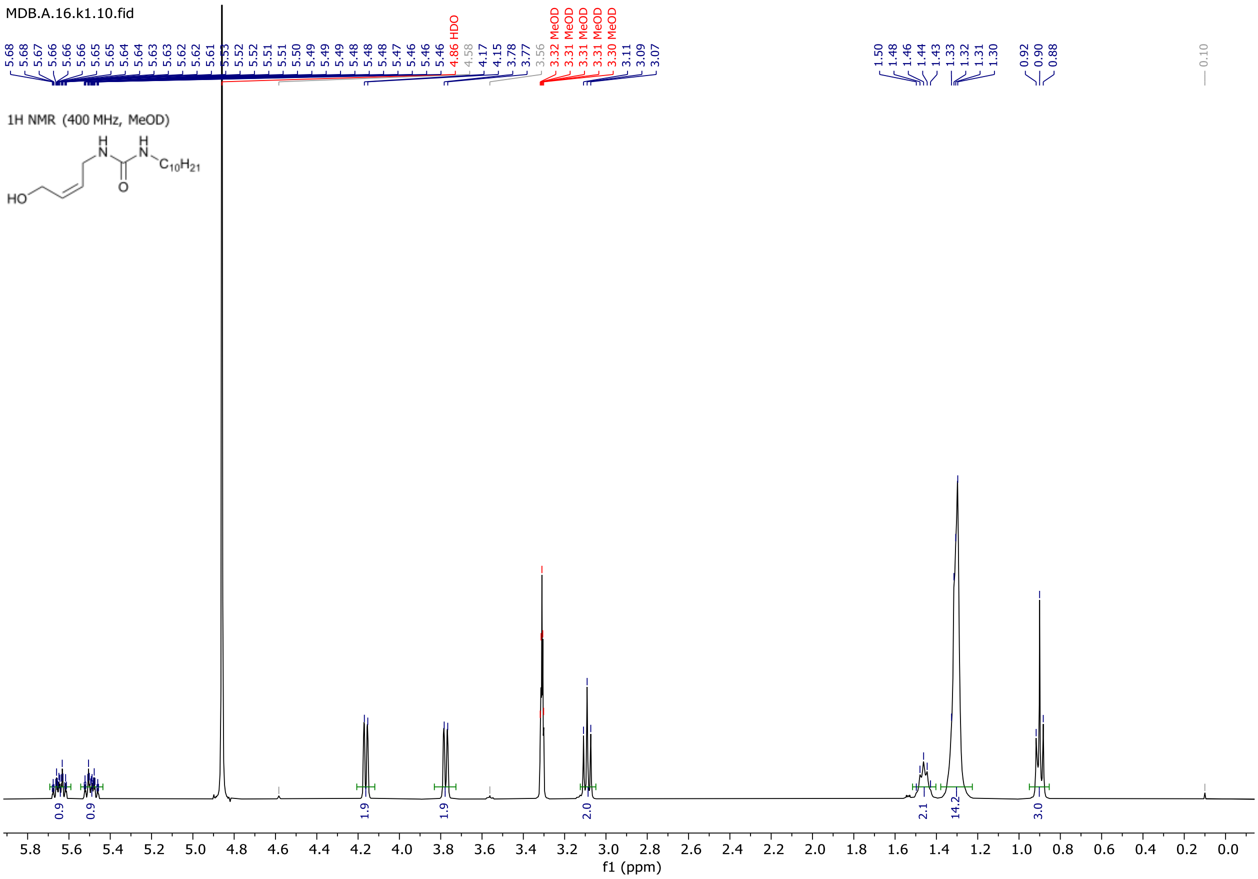

Figure S-17 ^{1}H NMR spectrum of **21c**.

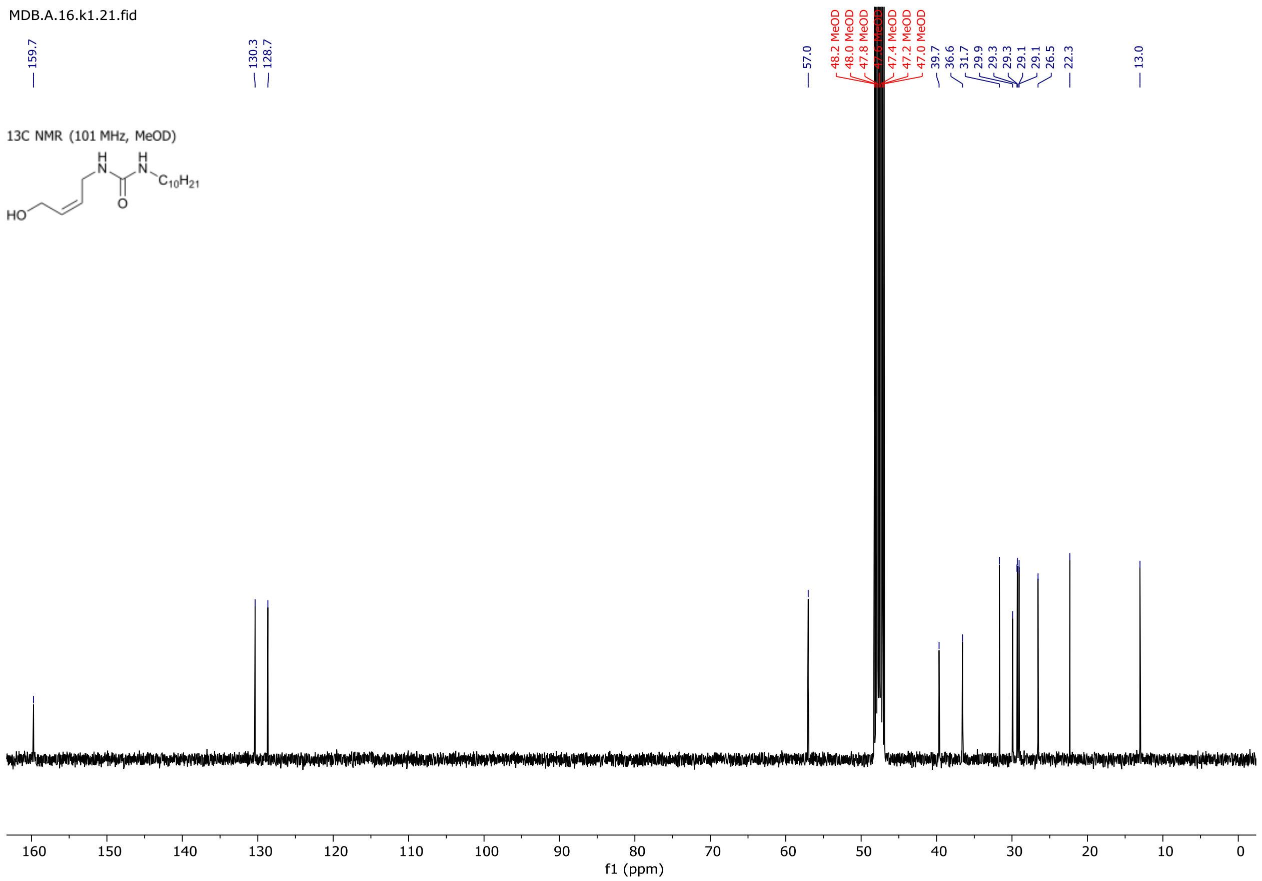

Figure S-18 ^{13}C NMR spectrum of **21c**.

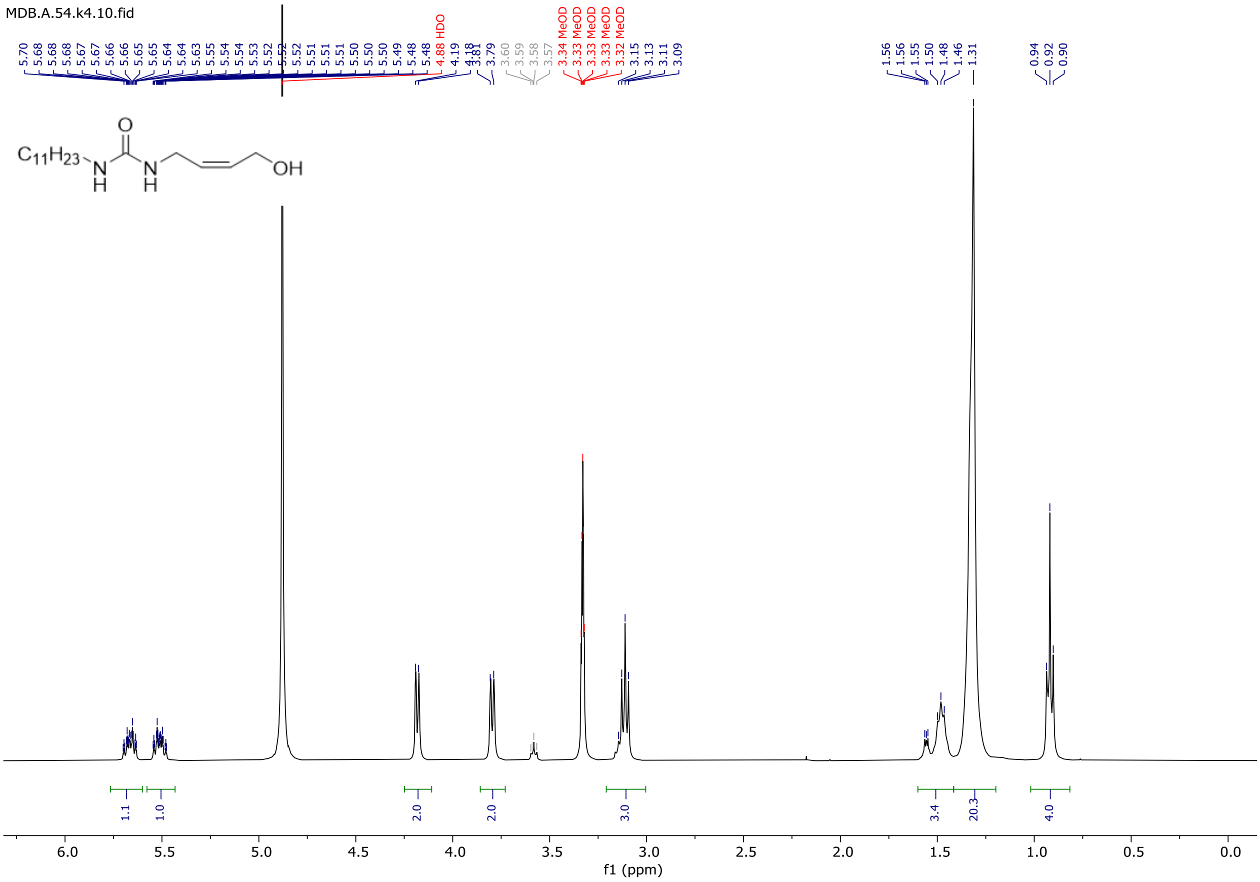

Figure S-19 ^{1}H NMR spectrum of **21d**.

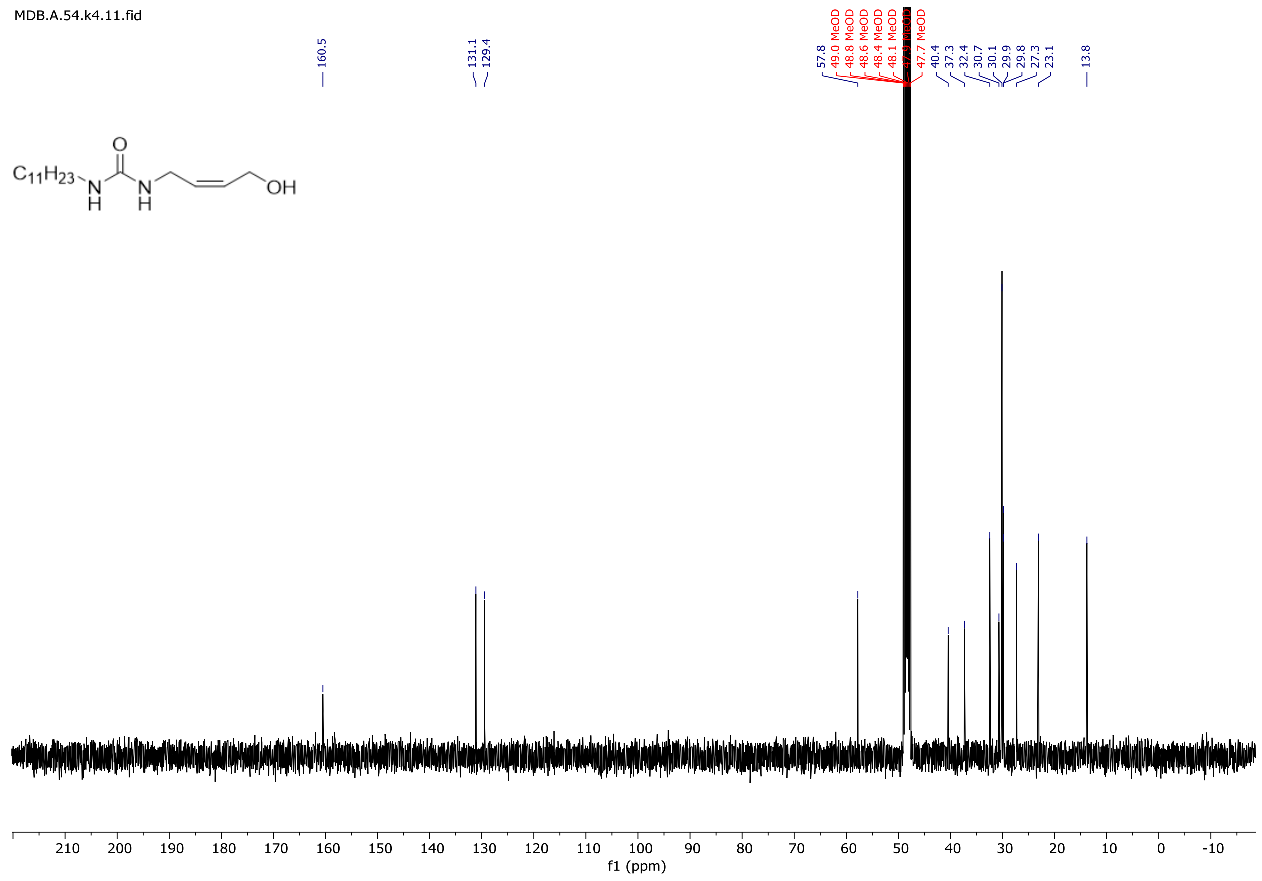

Figure S-20 ^{13}C NMR spectrum of **21d**.

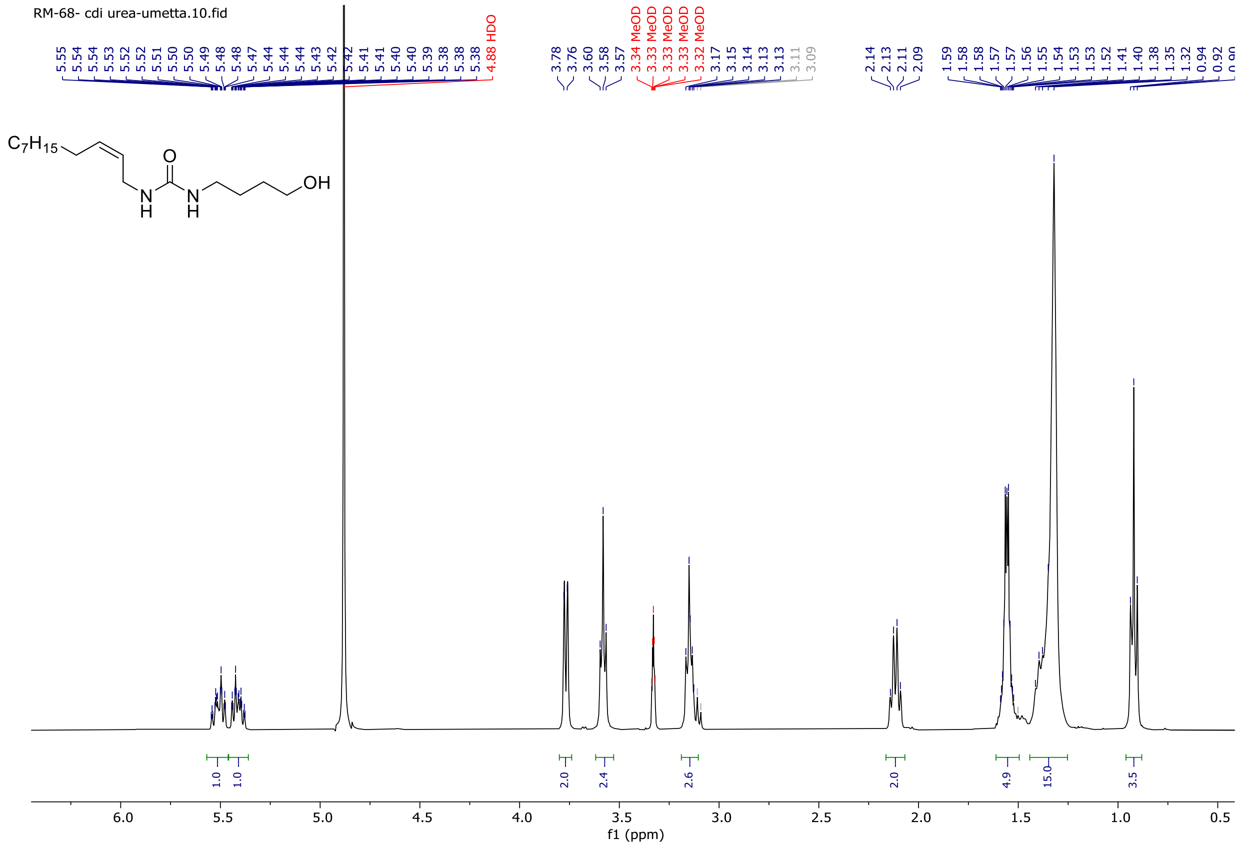

Figure S-21 ^{1}H NMR spectrum of **21e**.

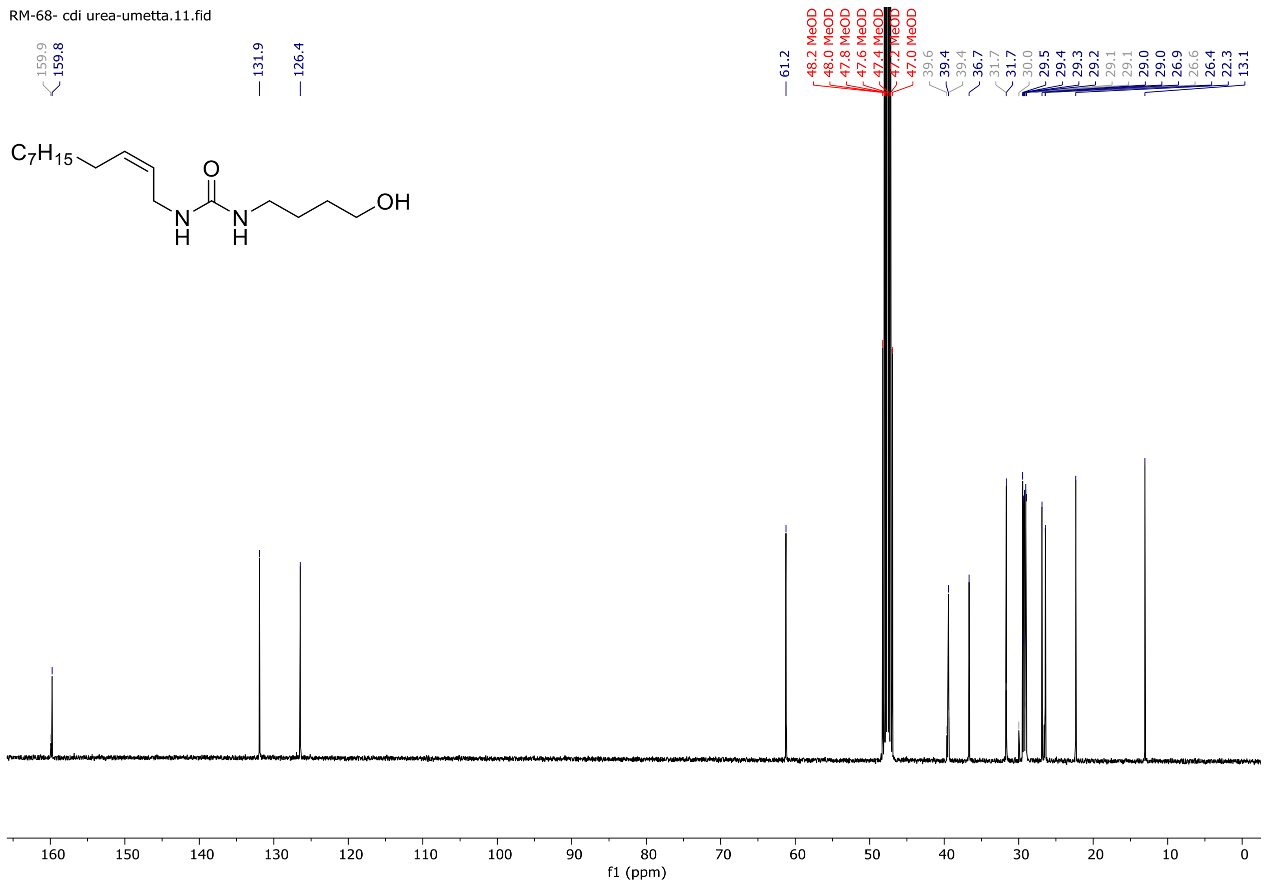

Figure S-22 ^{13}C NMR spectrum of **21e**.

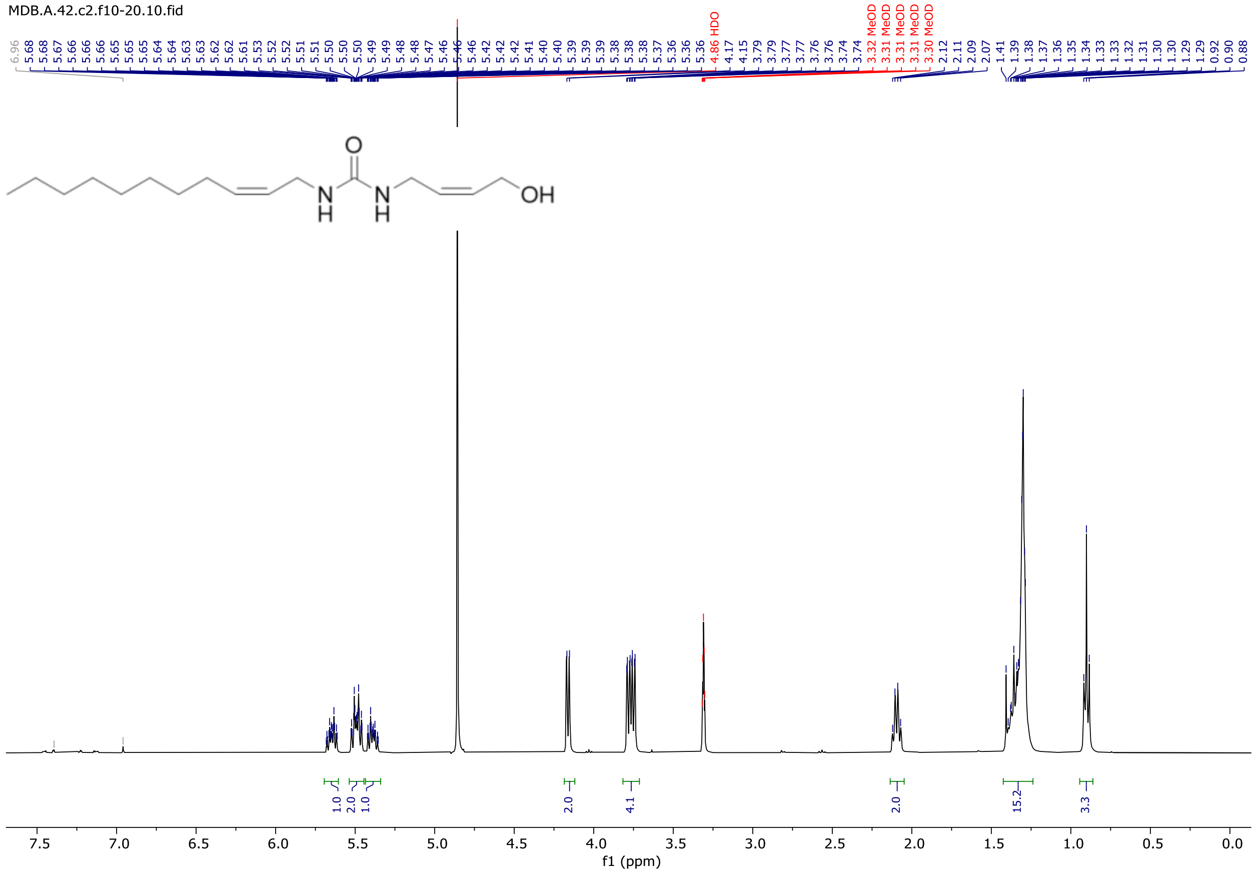

Figure S-23 ^{1}H NMR spectrum of **21f**.

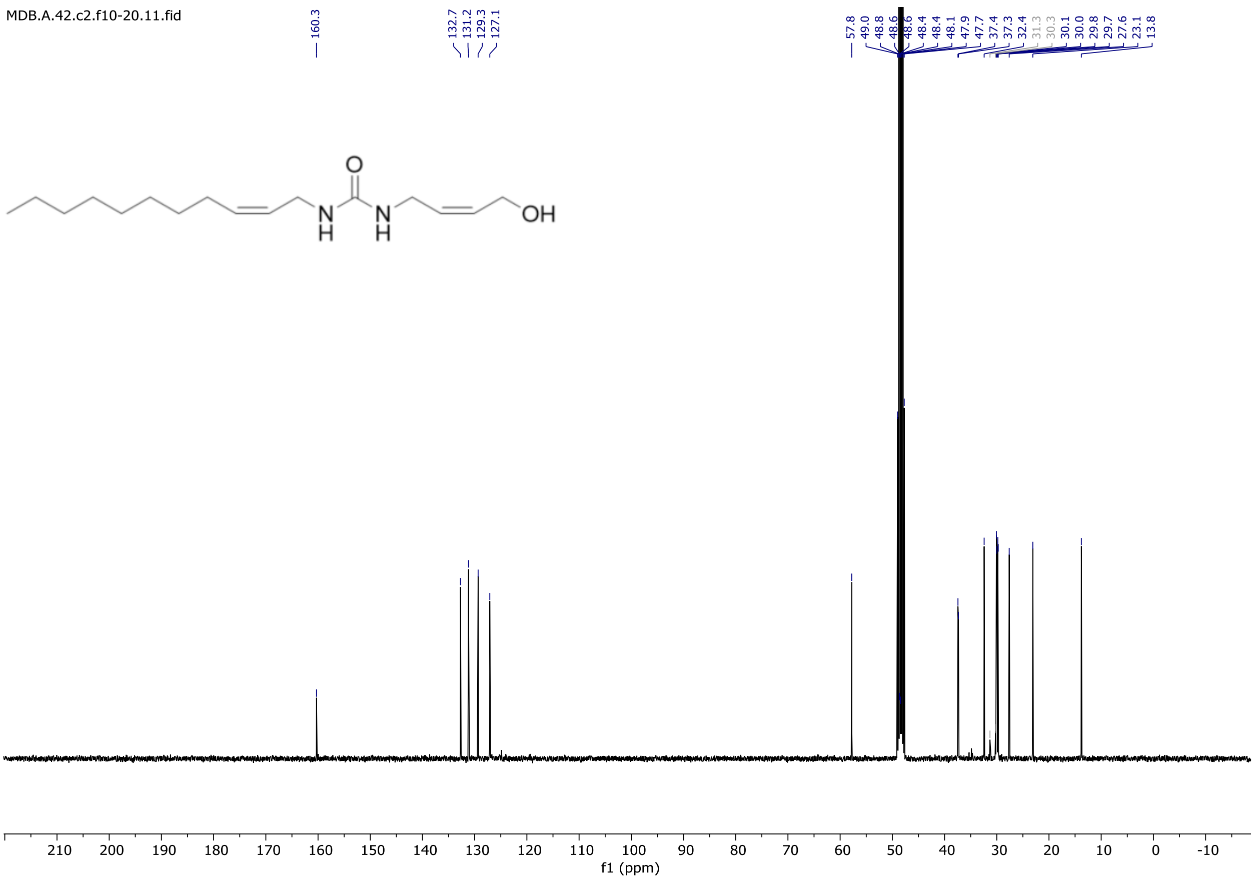

Figure S-24 ^{13}C NMR spectrum of **21f**.

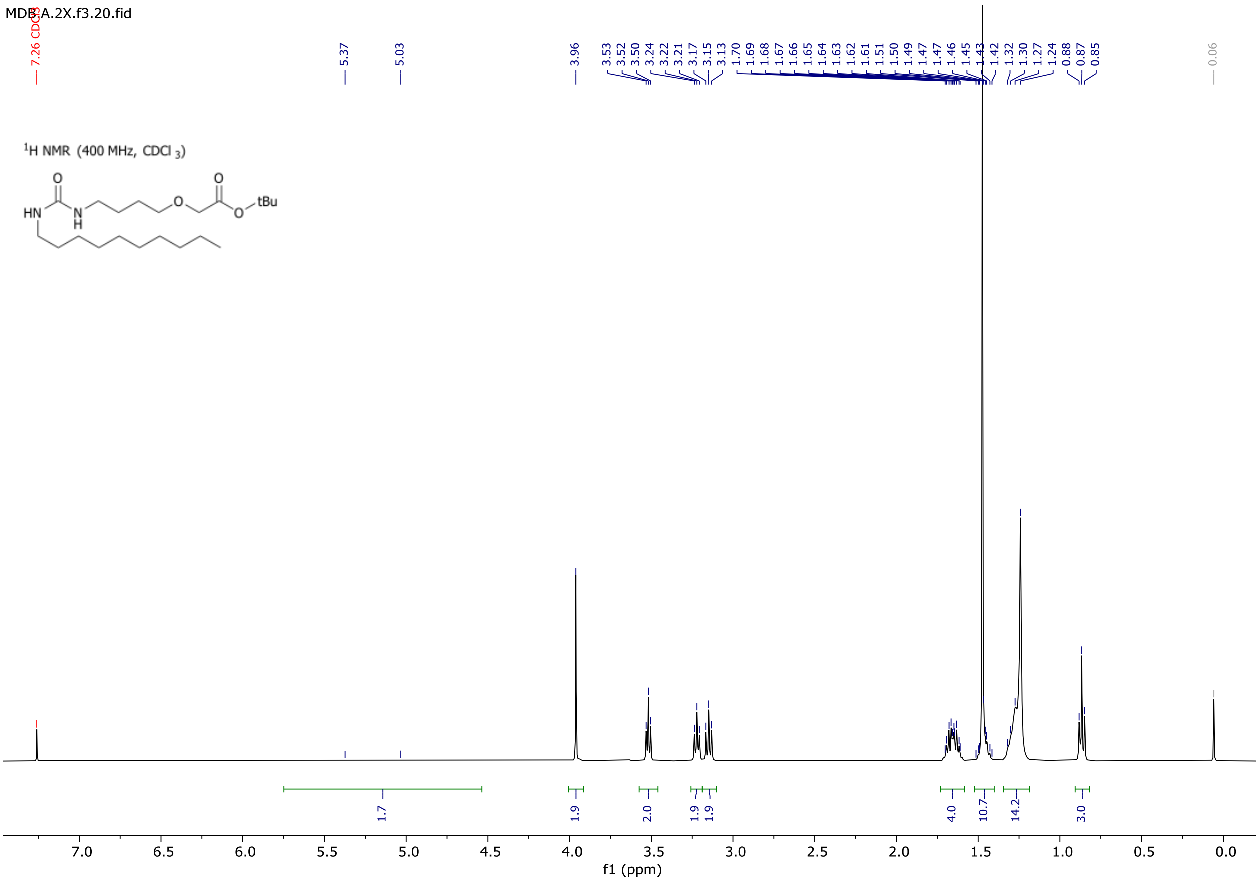

Figure S-25 ^{1}H NMR spectrum of **22a**.

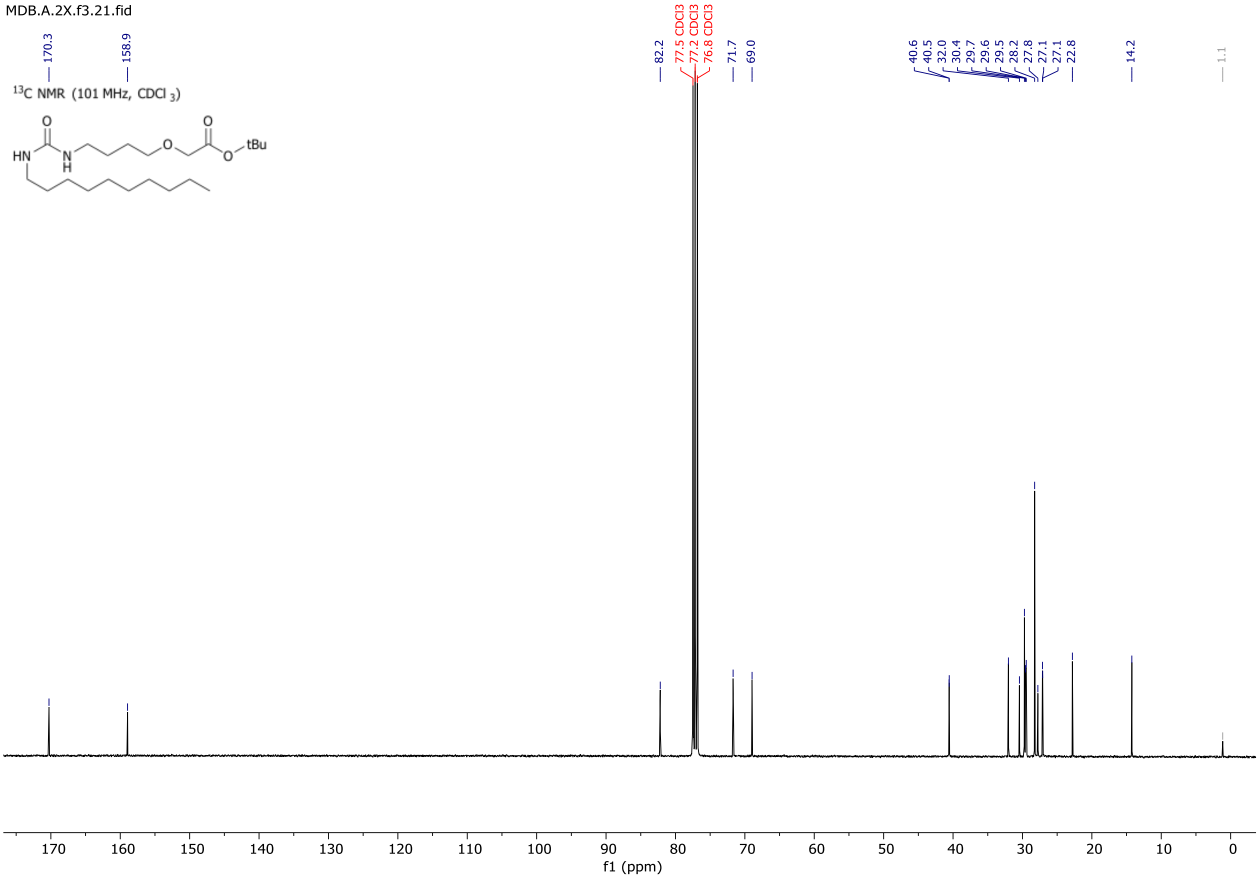

Figure S-26 ^{13}C NMR spectrum of **22a**.

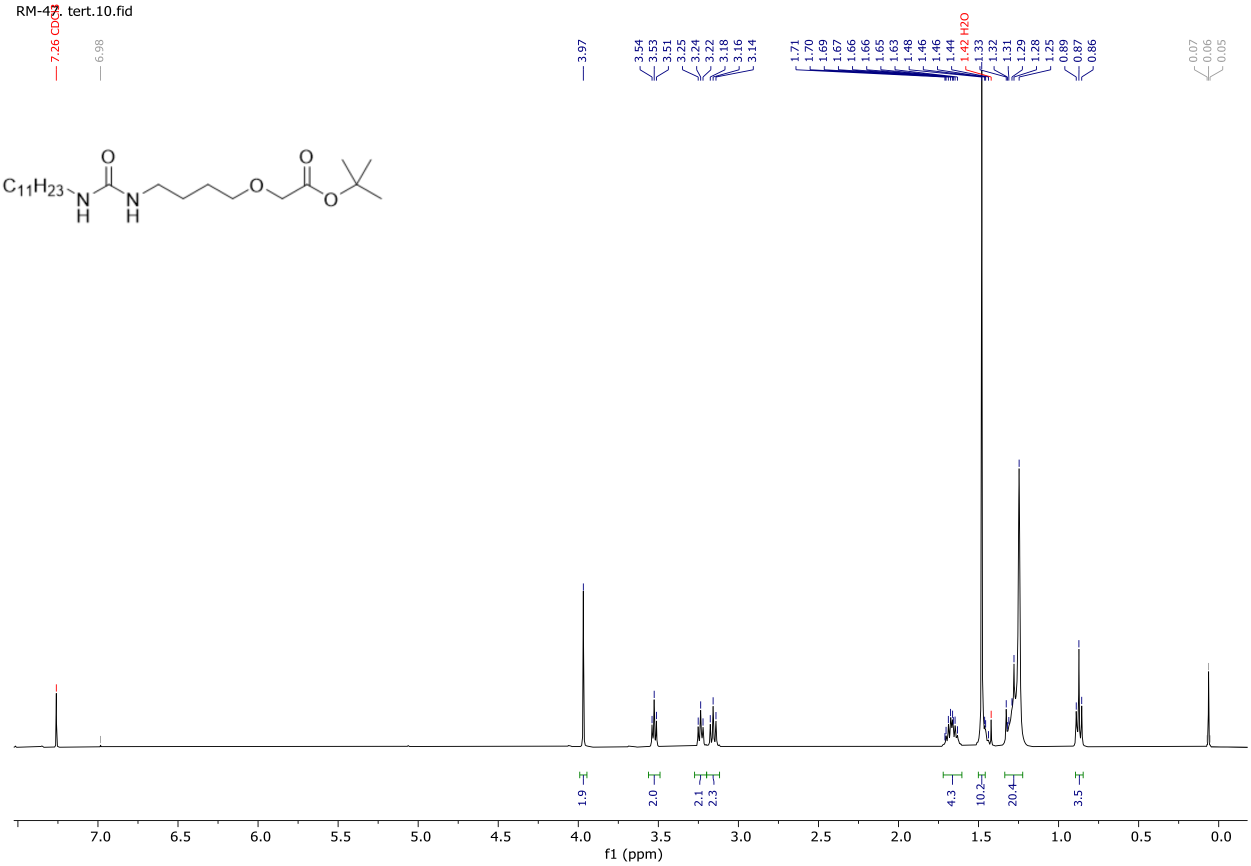

Figure S-27 ^{1}H NMR spectrum of **22b**.

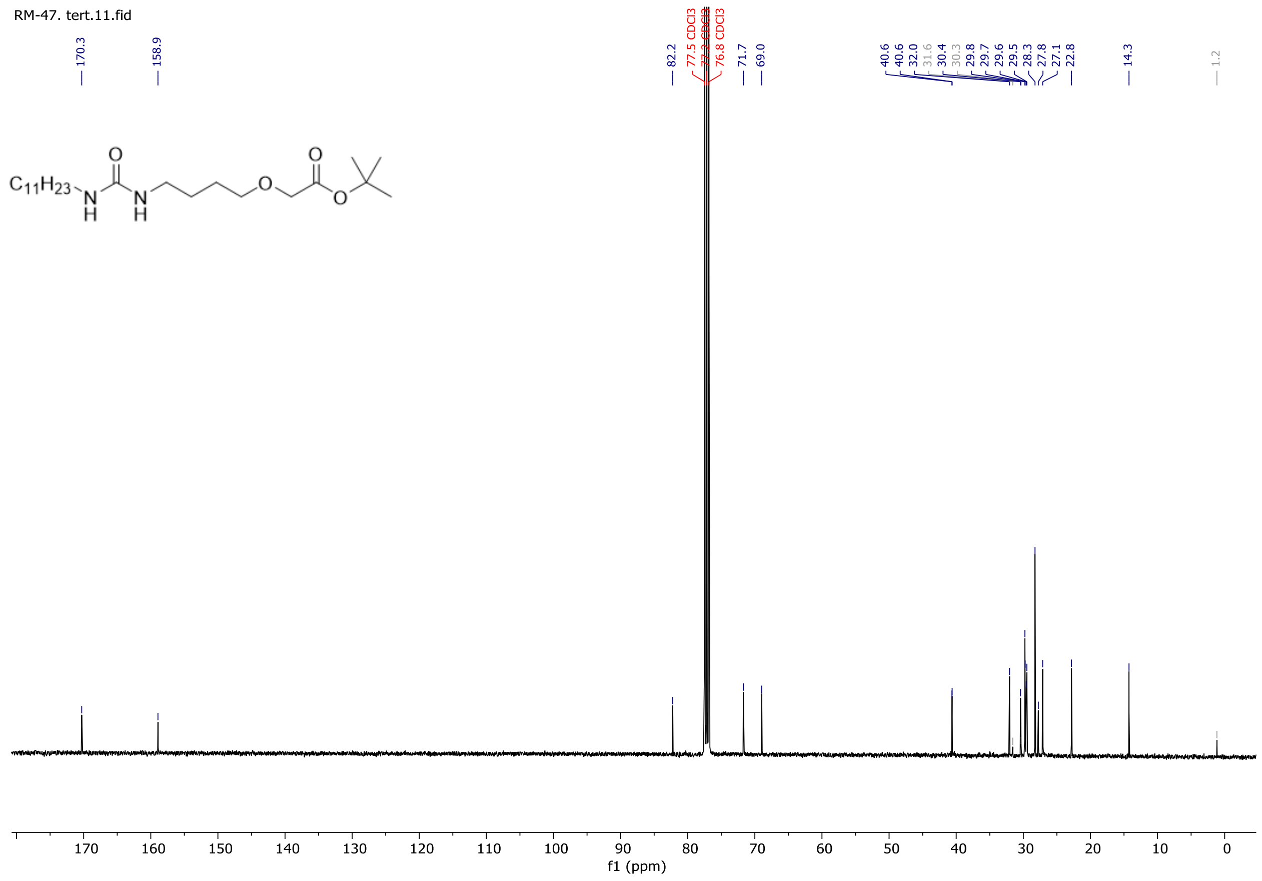

Figure S-28 ^{13}C NMR spectrum of **22b**.

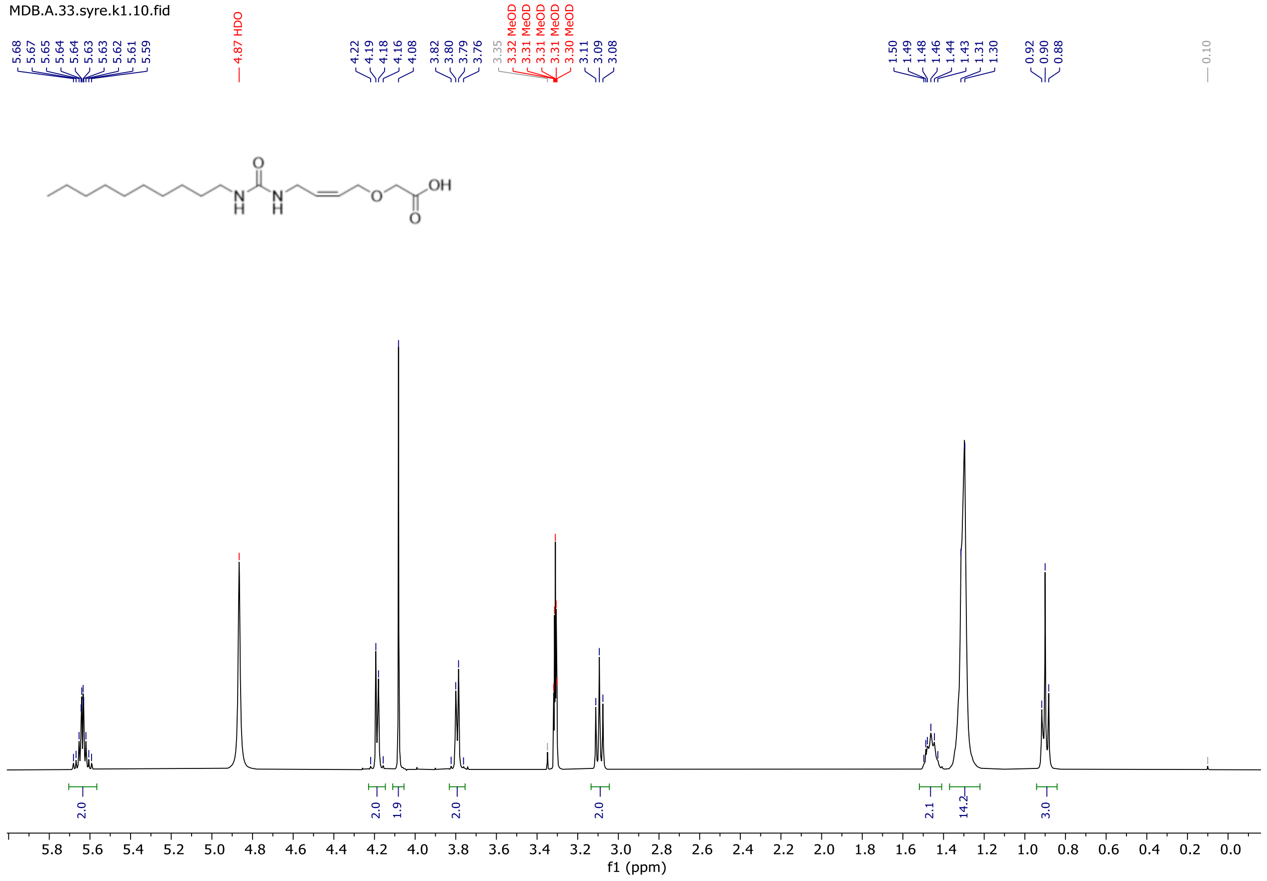

Figure S-29 ^{1}H NMR spectrum of **7c**.

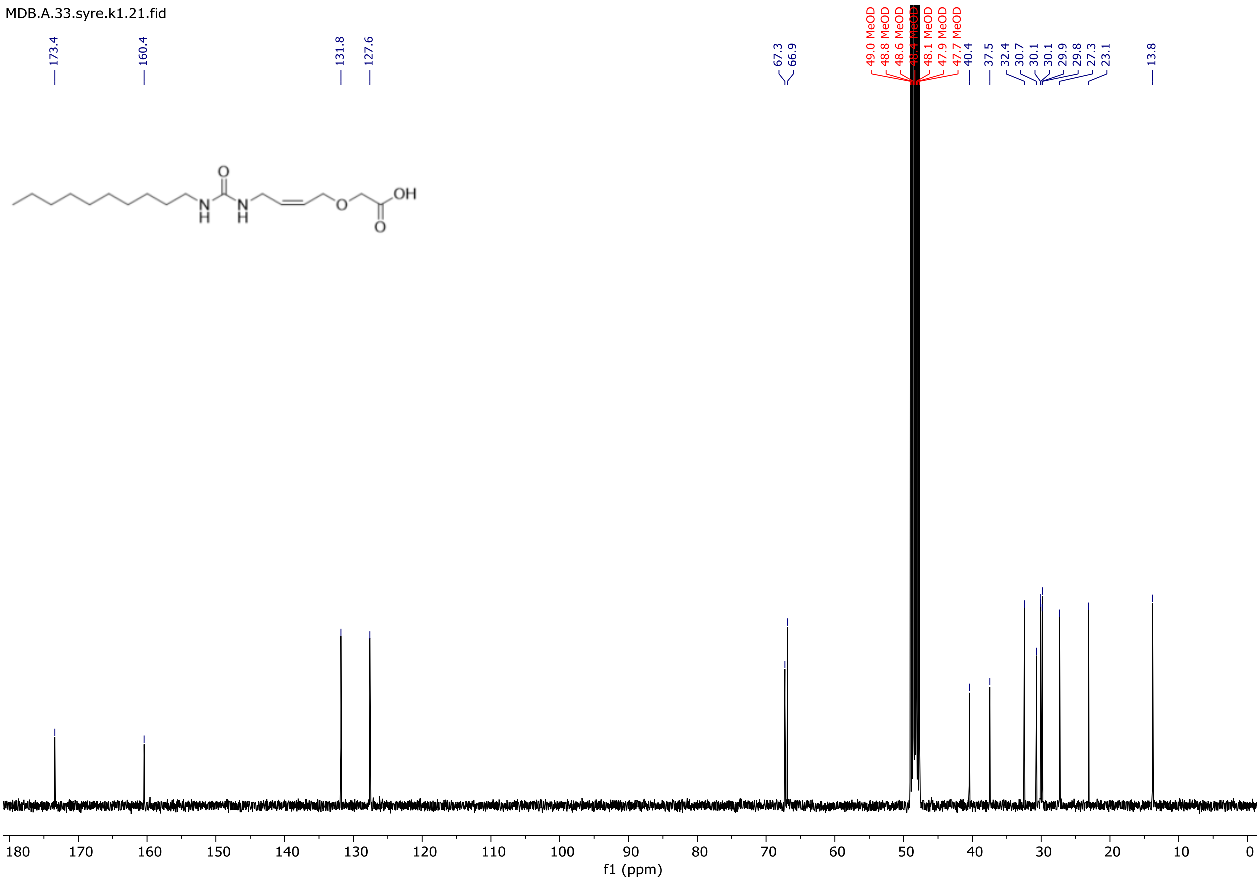

Figure S-30 ^{13}C NMR spectrum of **7c**.

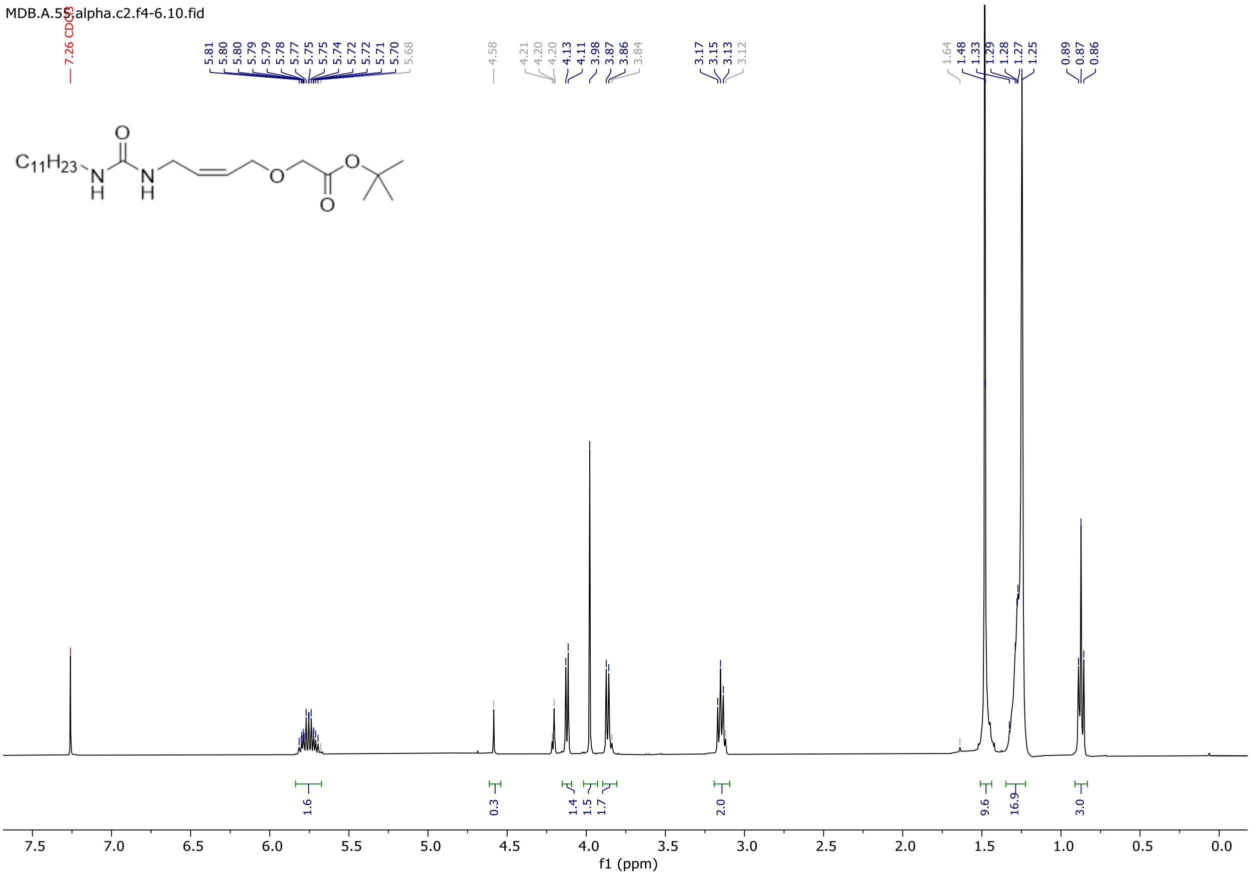

Figure S-31 ^{1}H NMR spectrum of **22c**.

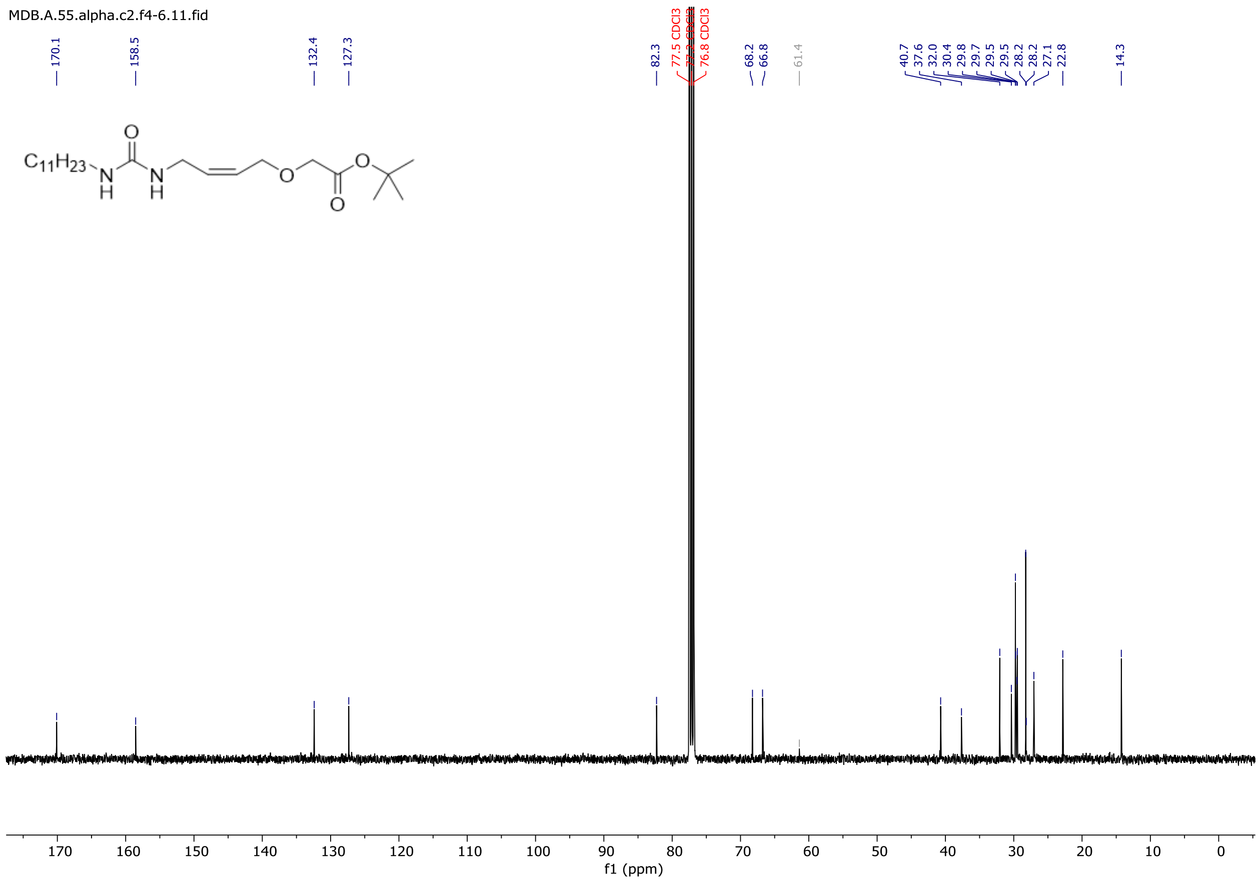

Figure S-32 ^{13}C NMR spectrum of **22c**.

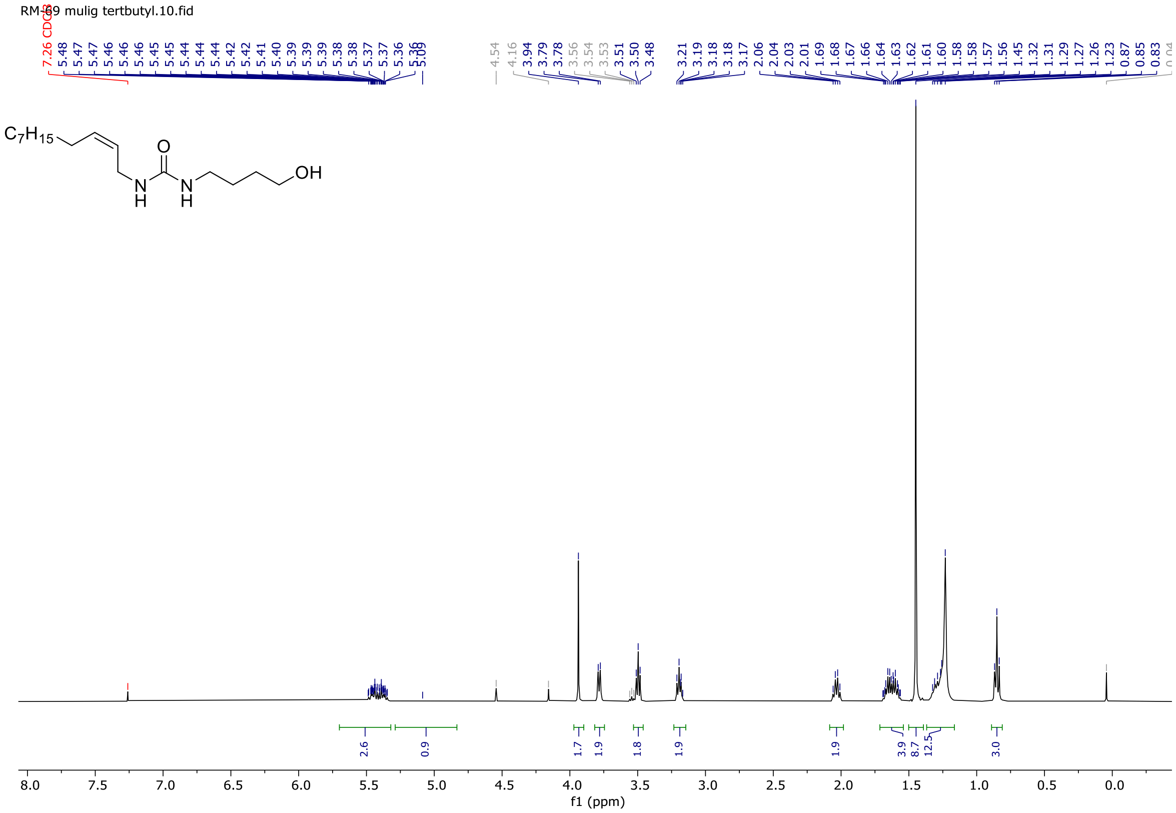

Figure S-33 ^{1}H NMR spectrum of **22d**.

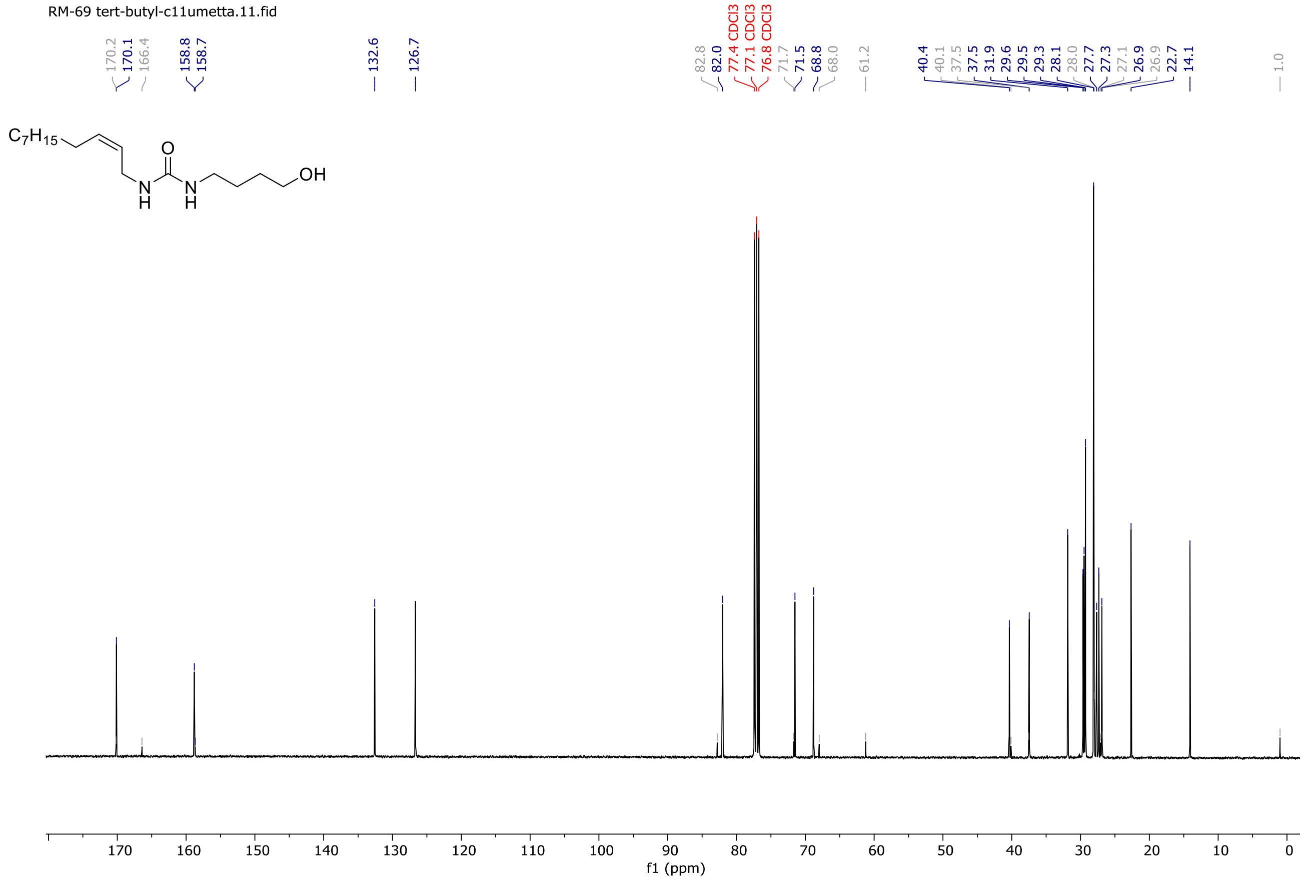

Figure S-34 ^{13}C NMR spectrum of **22d**.

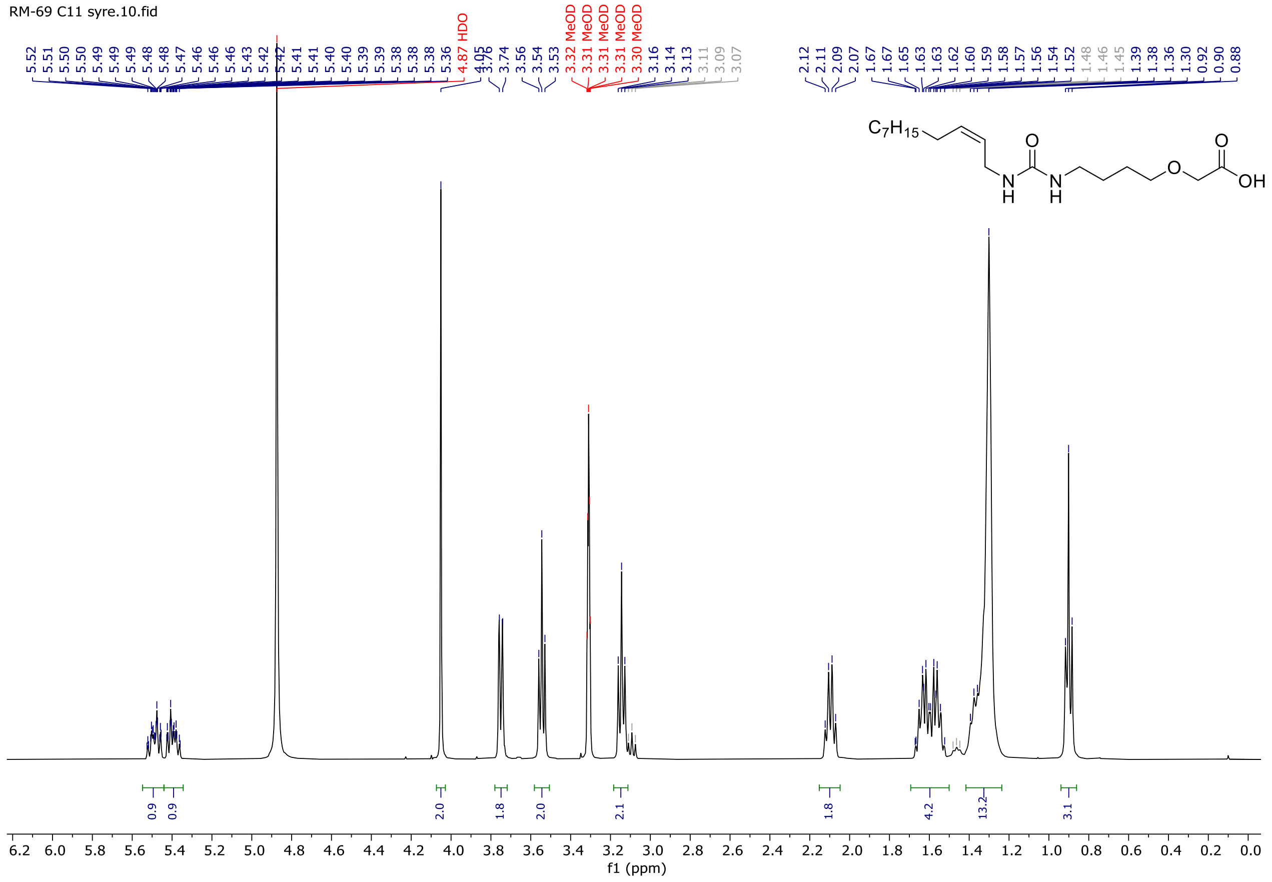

Figure S-35 ^{1}H NMR spectrum of **7e**.

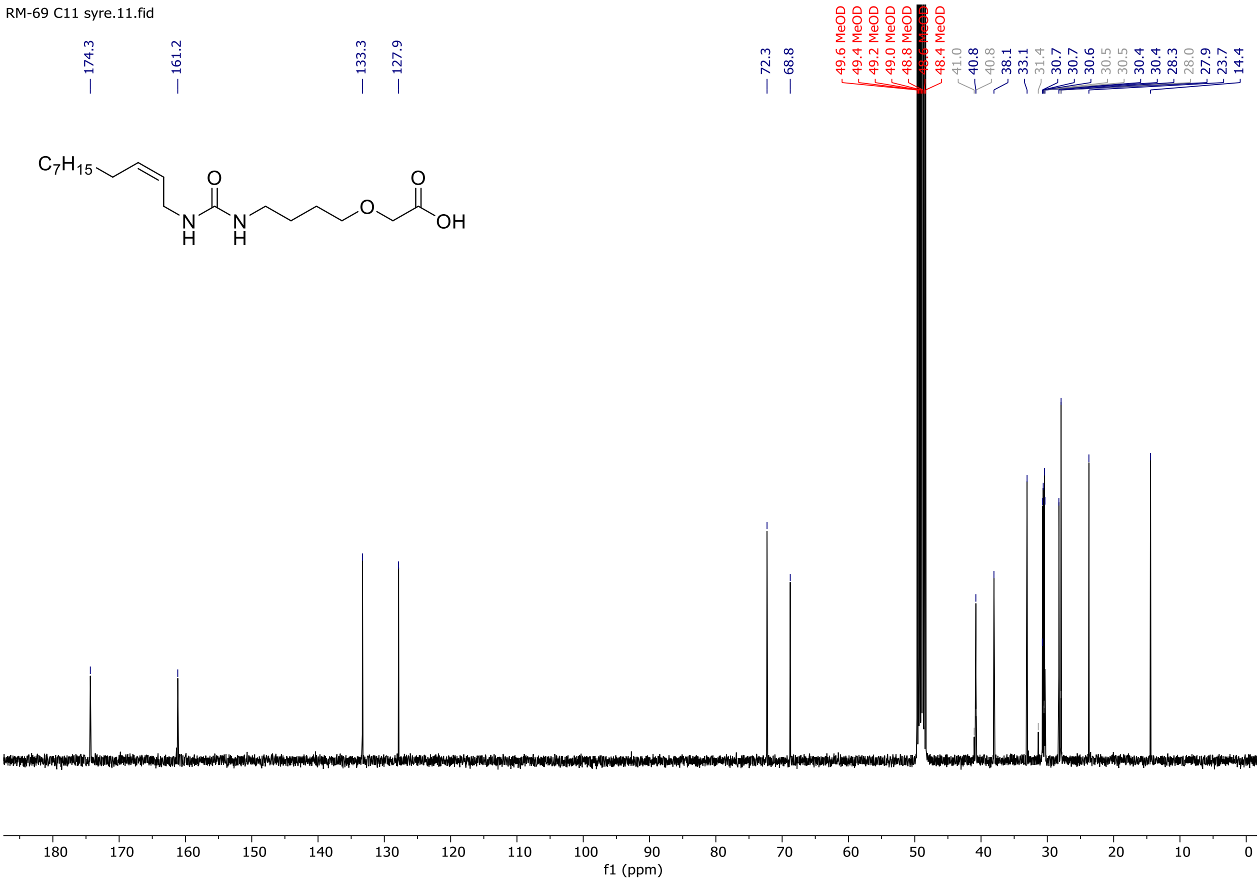

Figure S-36 ^{13}C NMR spectrum of **7e**.

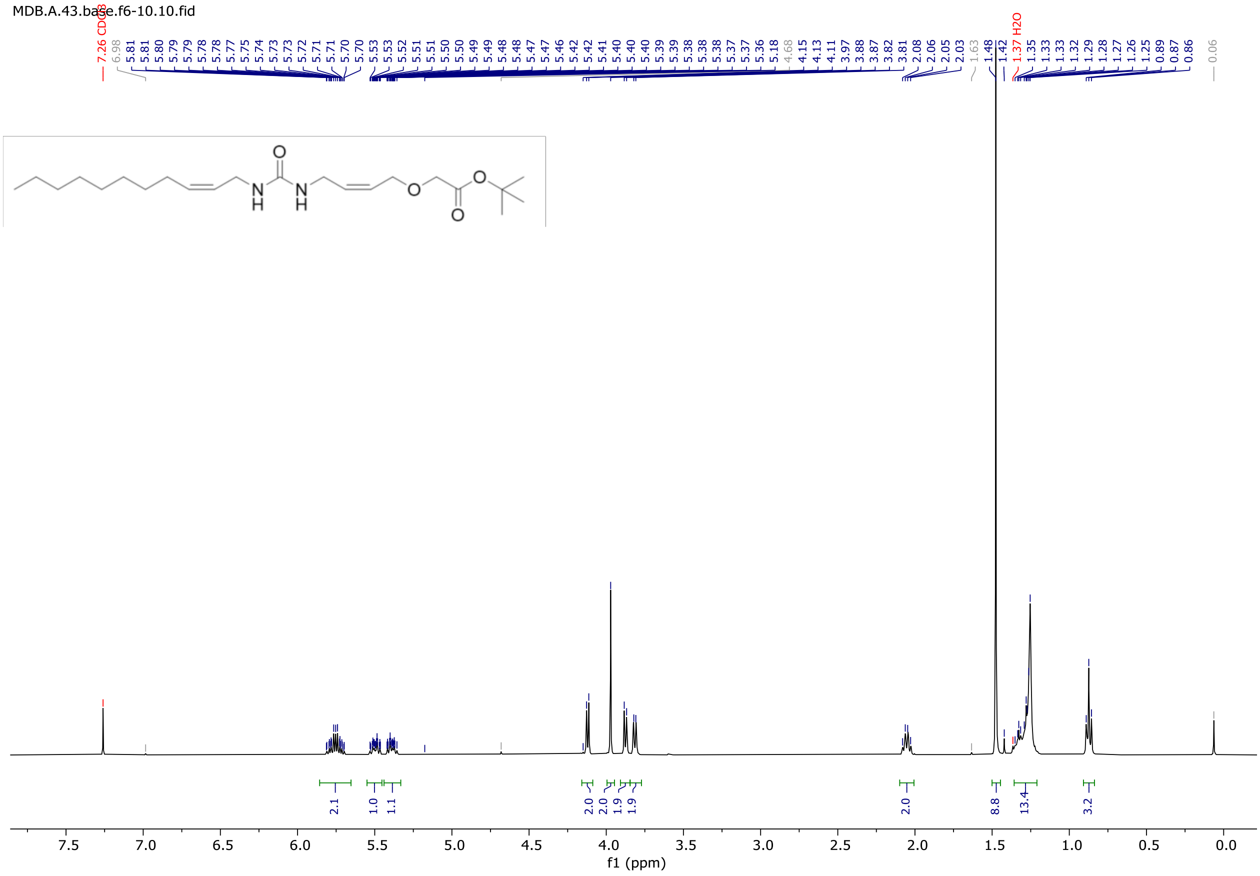

Figure S-37 ^{1}H NMR spectrum of **22e**.

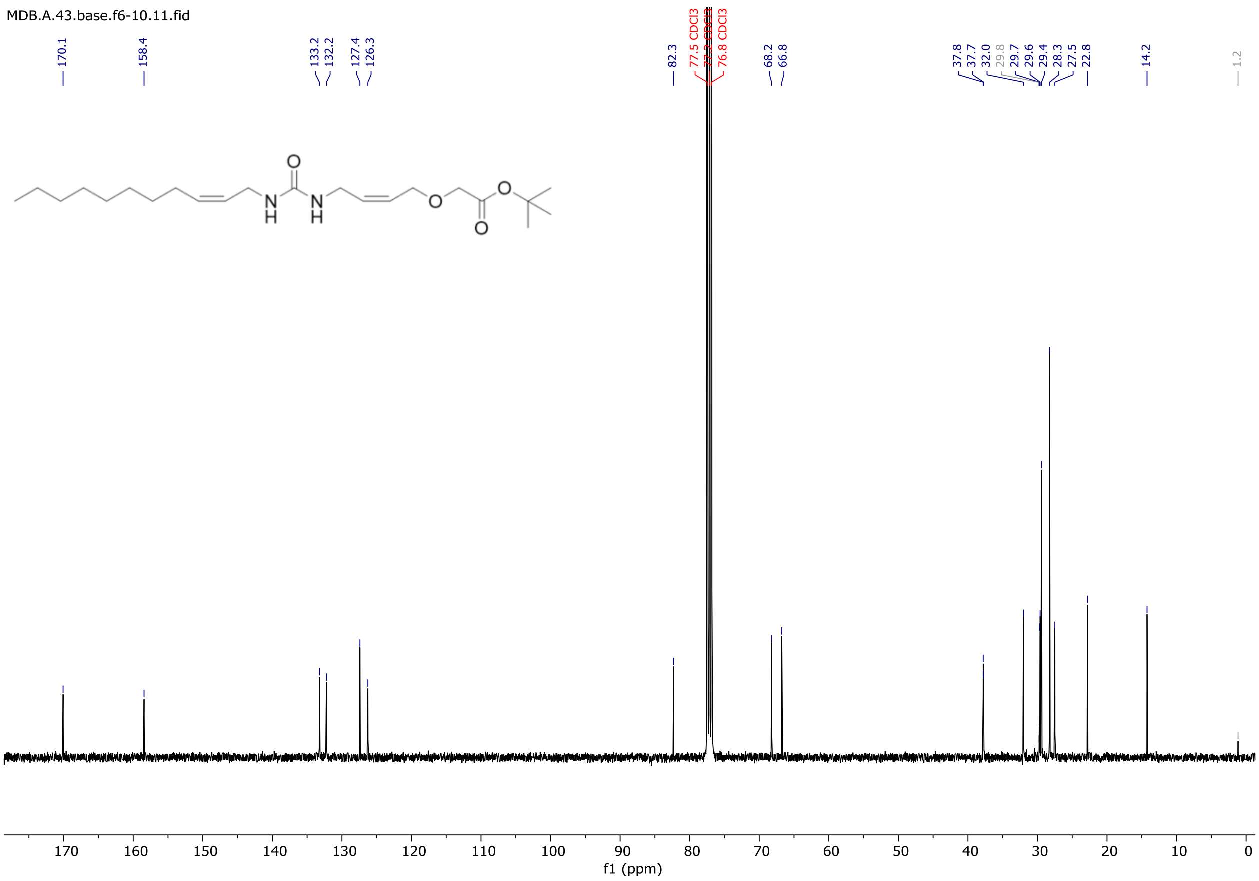

Figure S-38 ^{13}C NMR spectrum of **22e**.

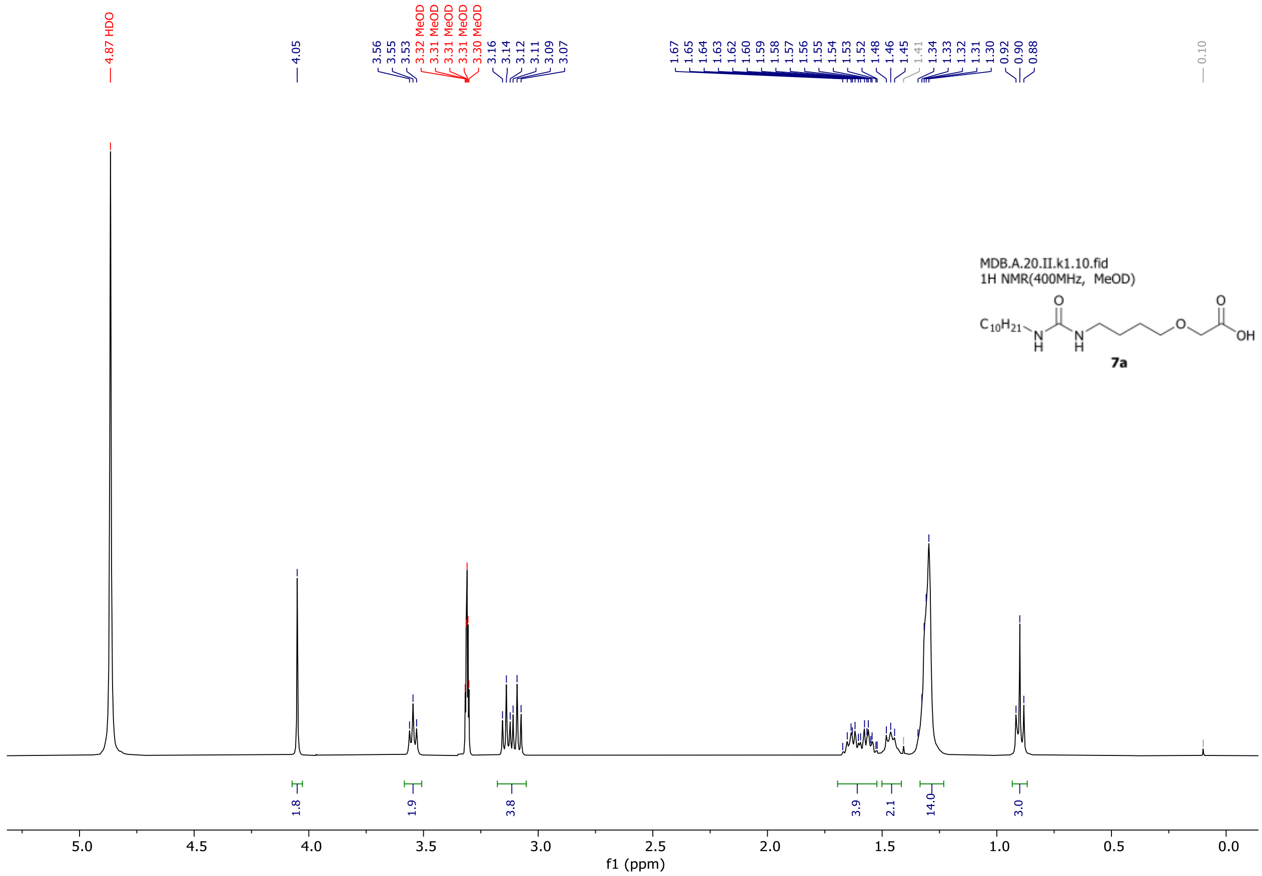

Figure S-39 ^{1}H NMR spectrum of **7a**.

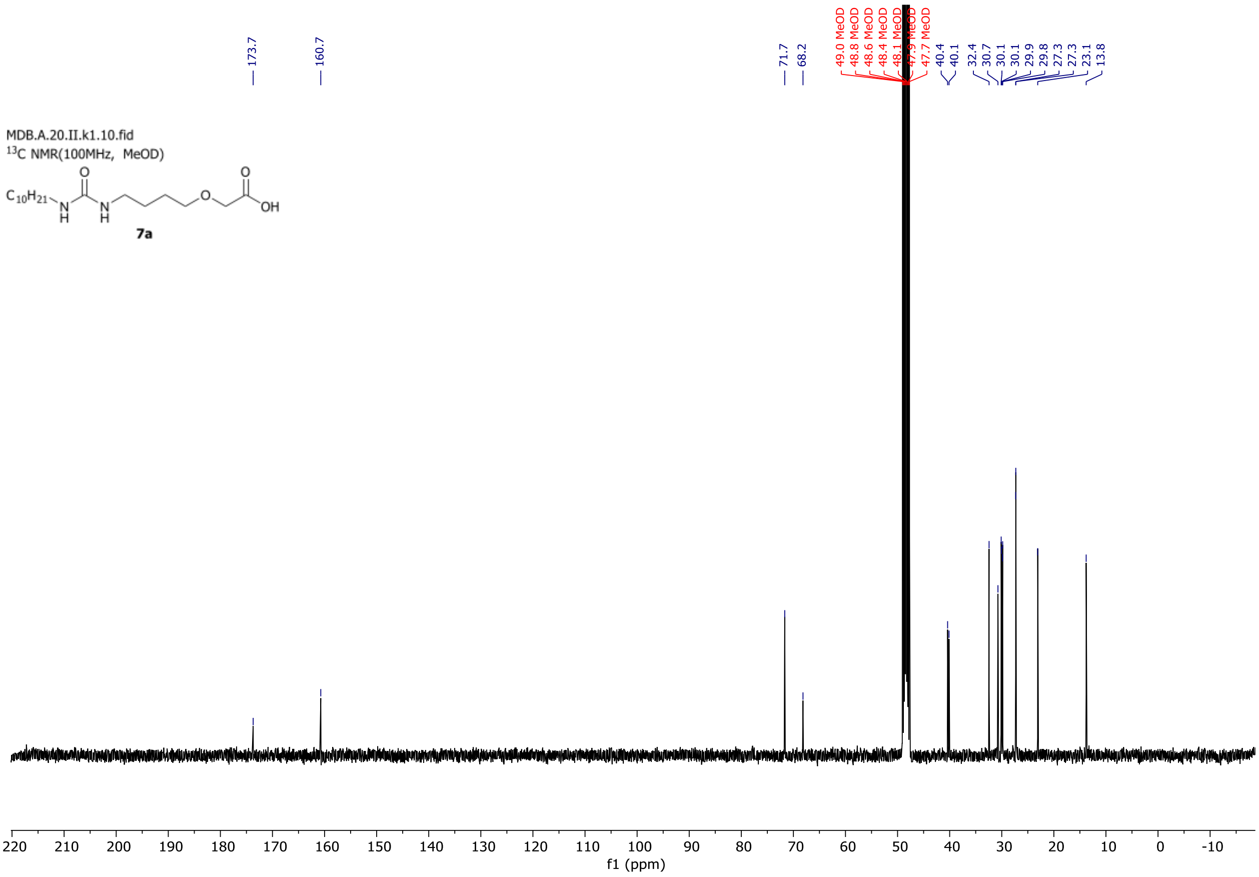

Figure S-40 ^{13}C NMR spectrum of **7a**.

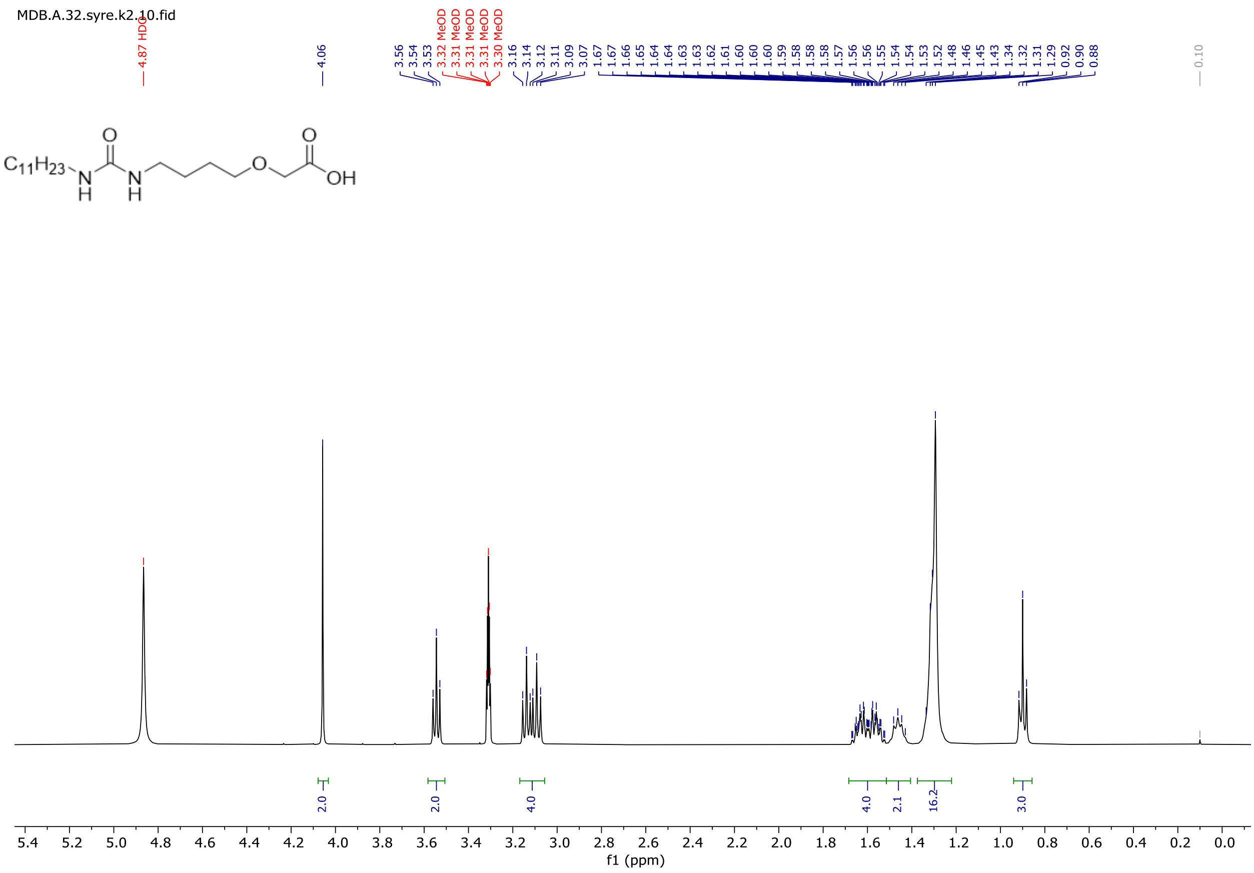

Figure S-41 ^{1}H NMR spectrum of **7b**.

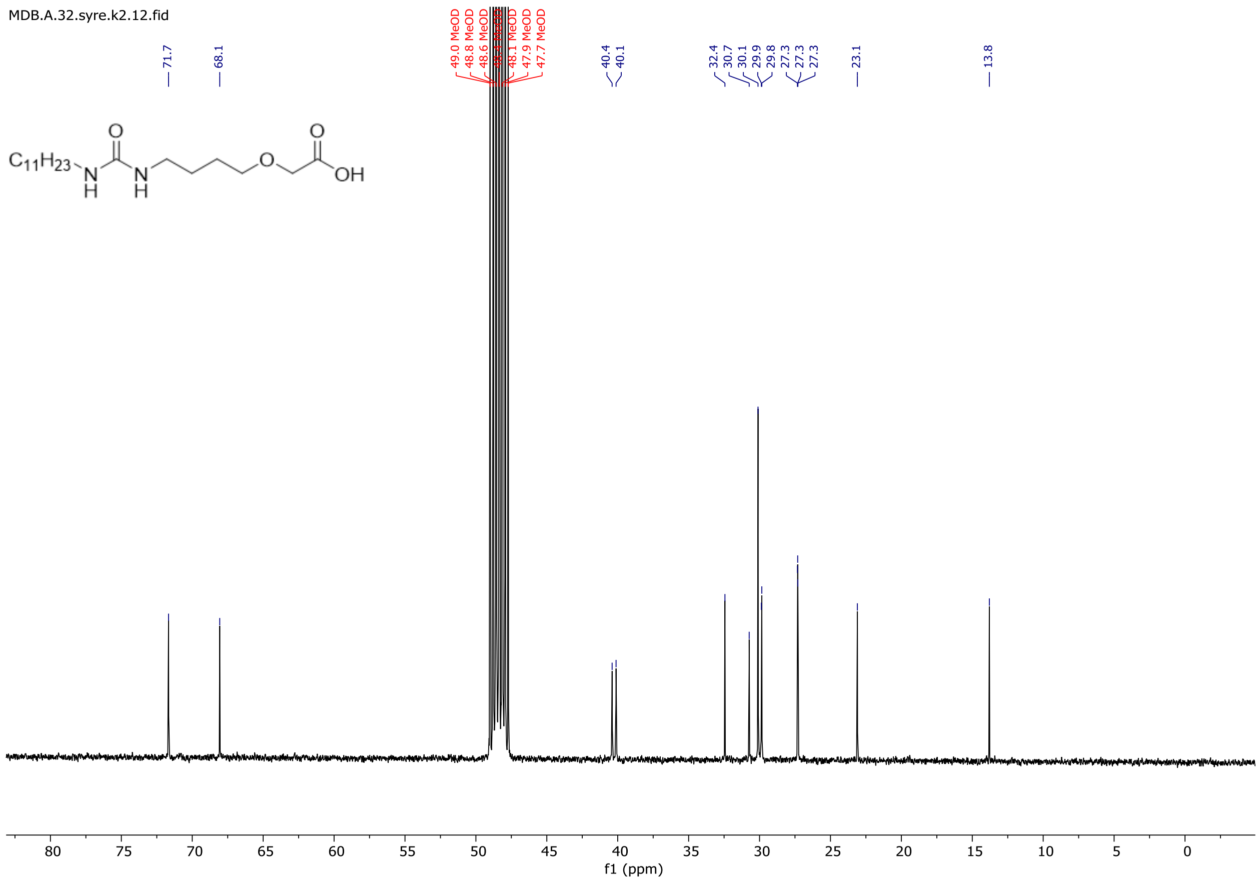

Figure S-42 ^{13}C NMR spectrum of **7b**.

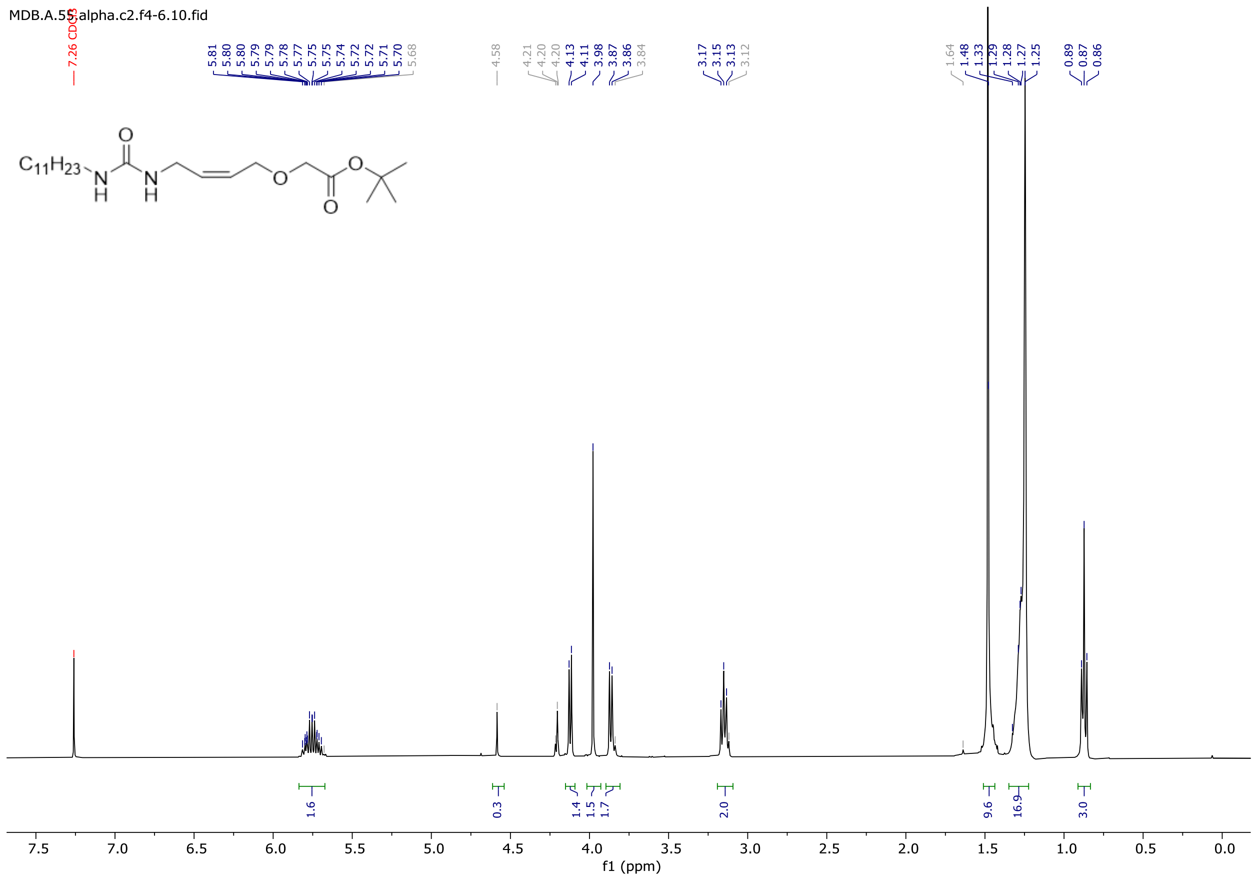

Figure S-43 ^{1}H NMR spectrum of **7d**.

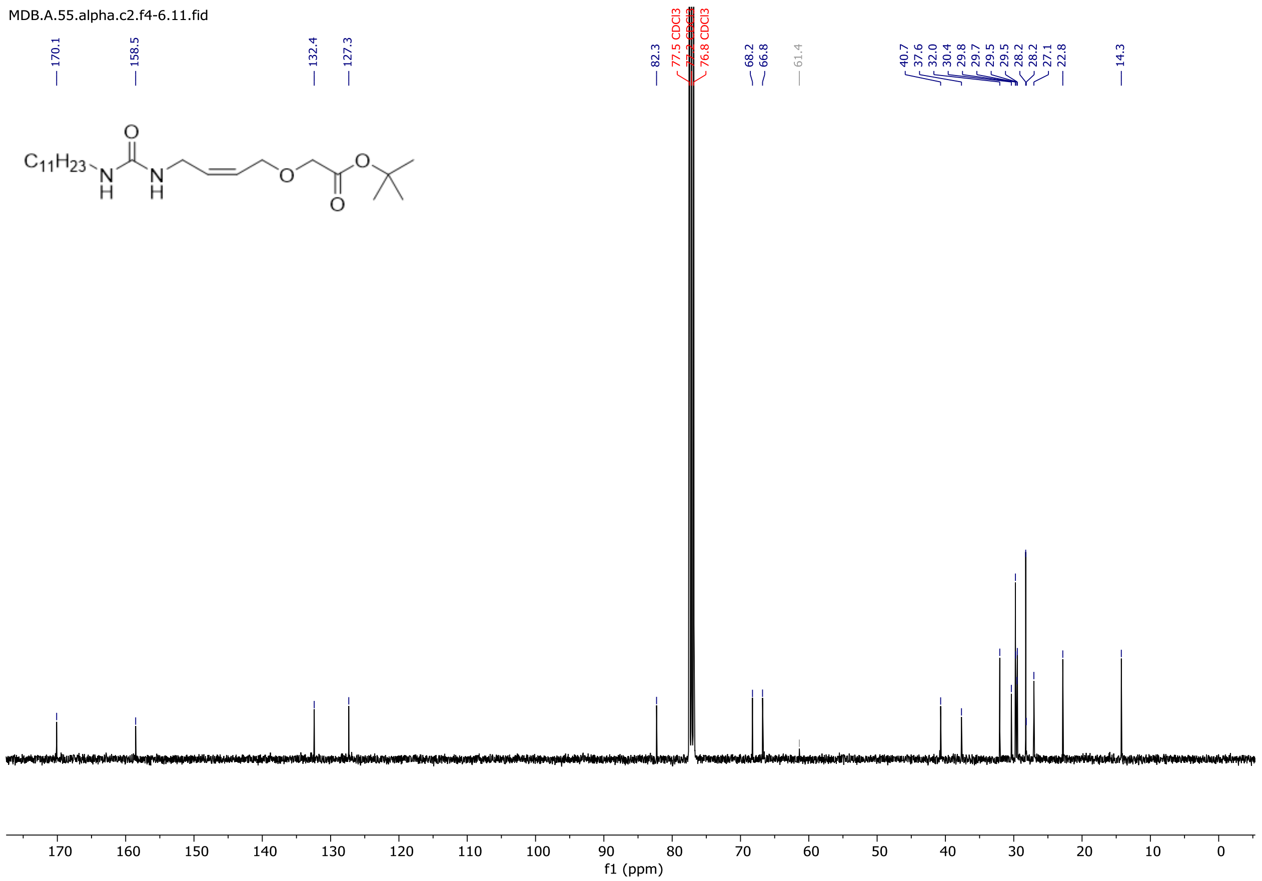

Figure S-44 ^{13}C NMR spectrum of **7d**.

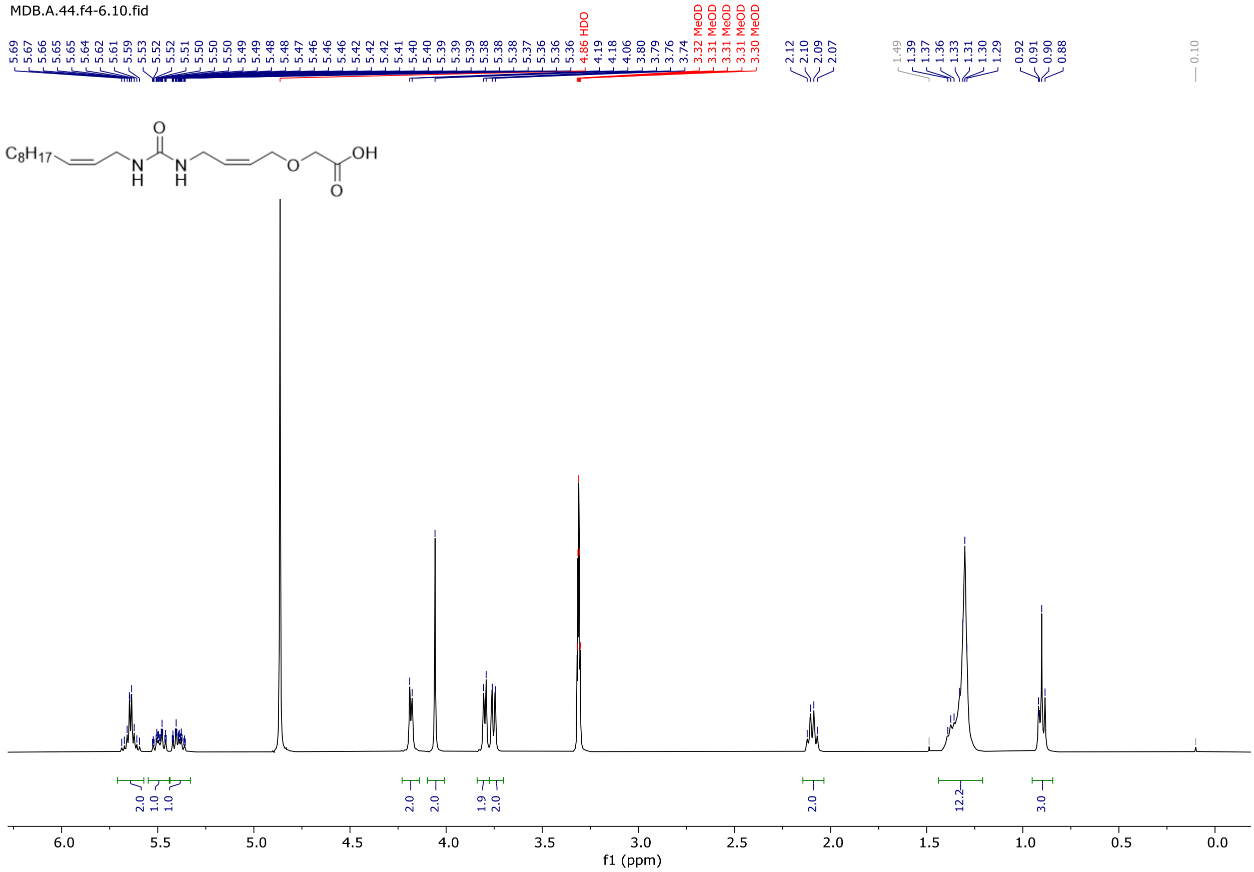

Figure S-45 ^{1}H NMR spectrum of **7f**.

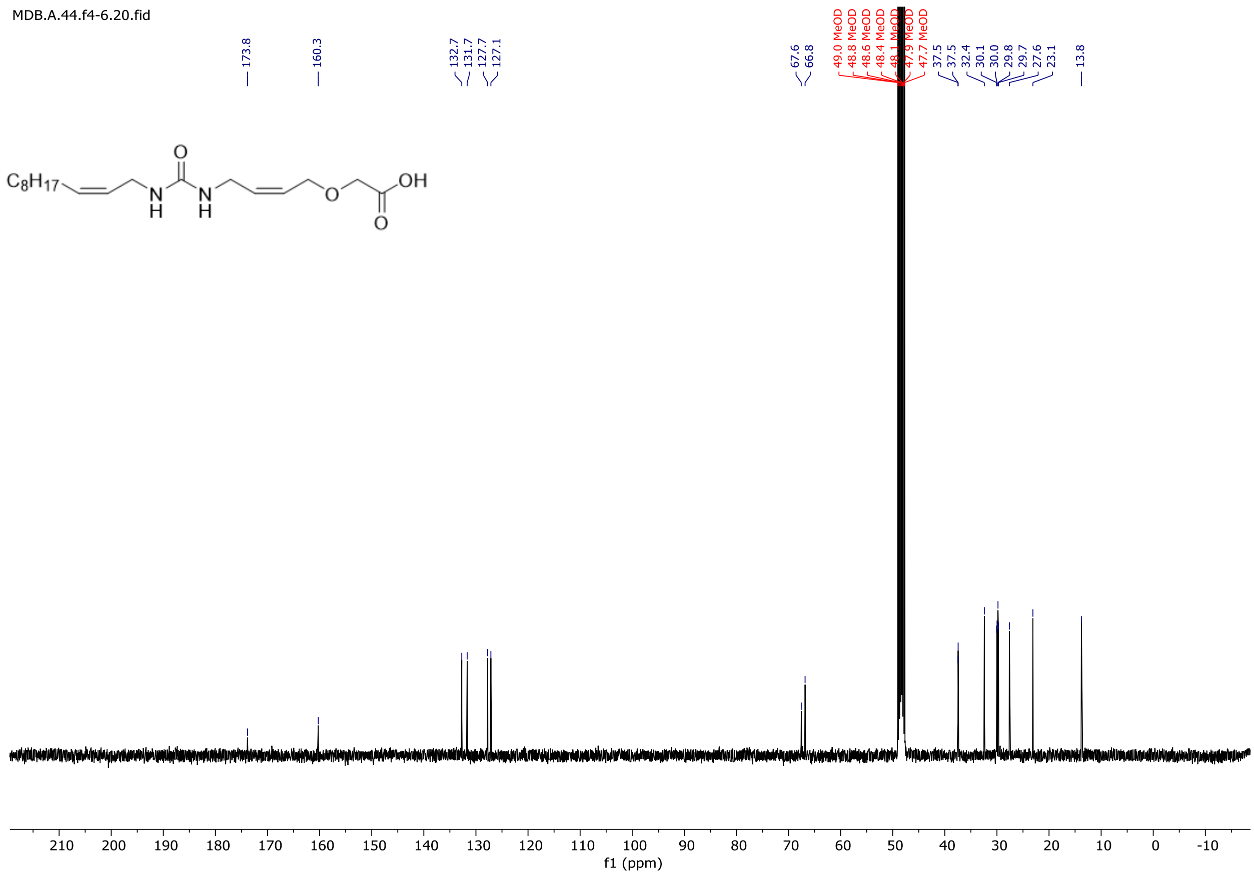

Figure S-46 ^{13}C NMR spectrum of **7f**.

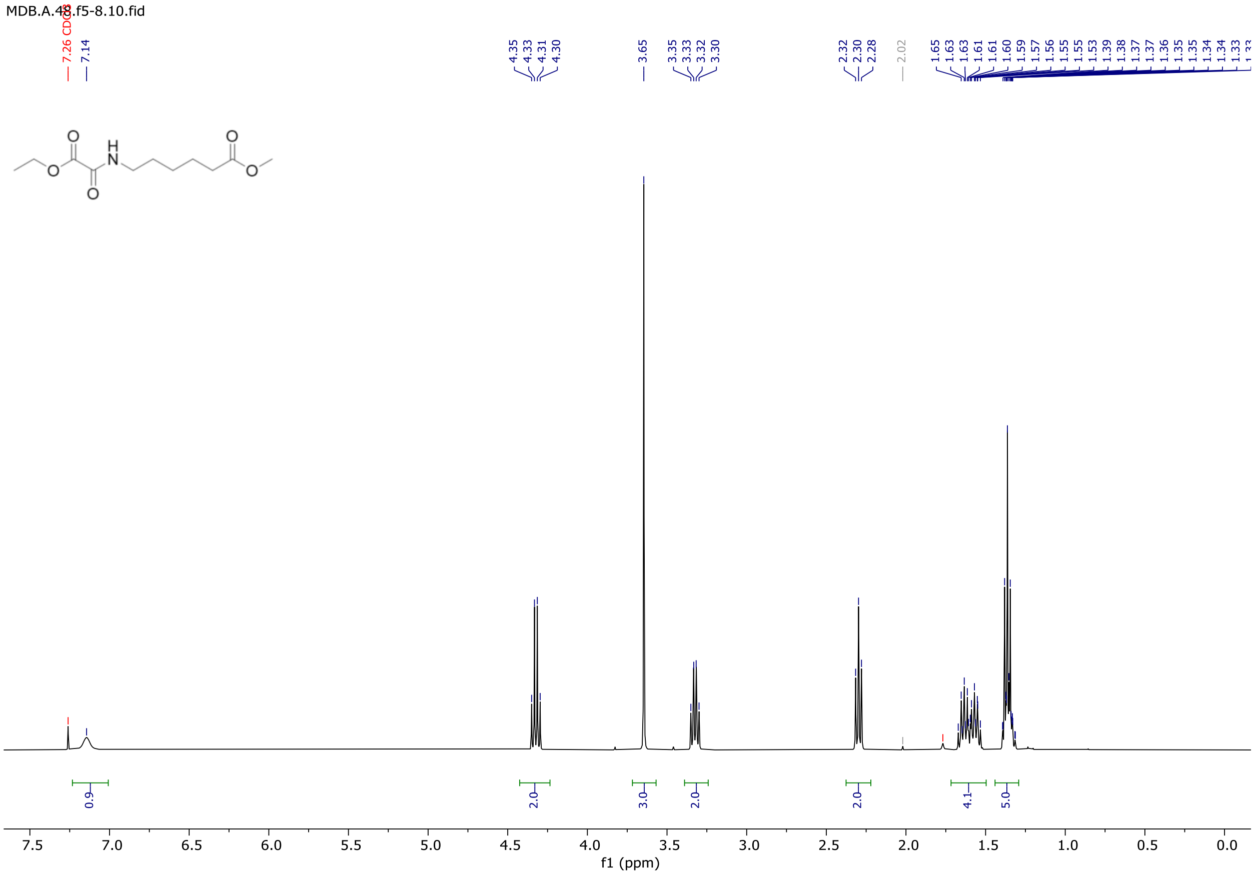

Figure S-47 ¹H NMR spectrum of **25a**.

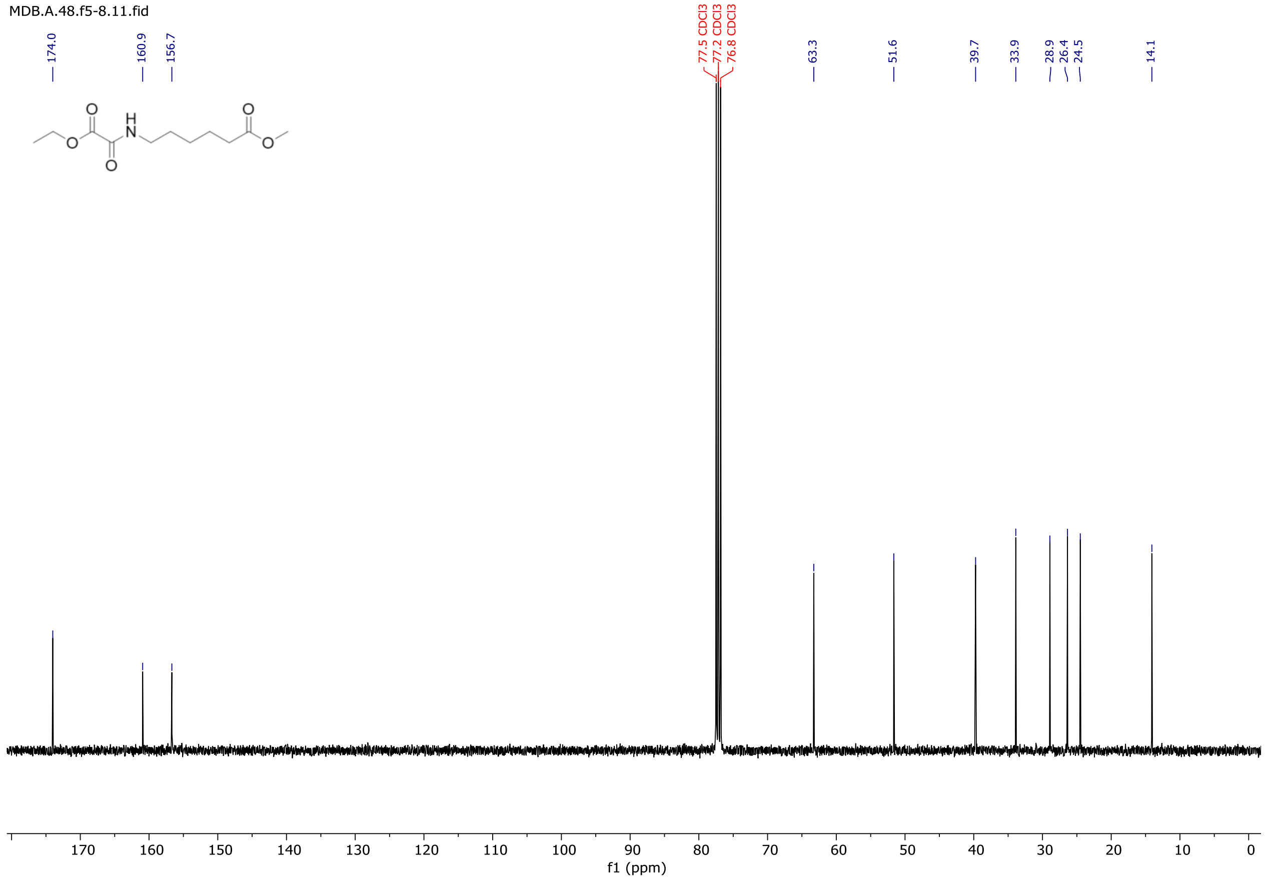

Figure S-48 ^{13}C NMR spectrum of **25a**.

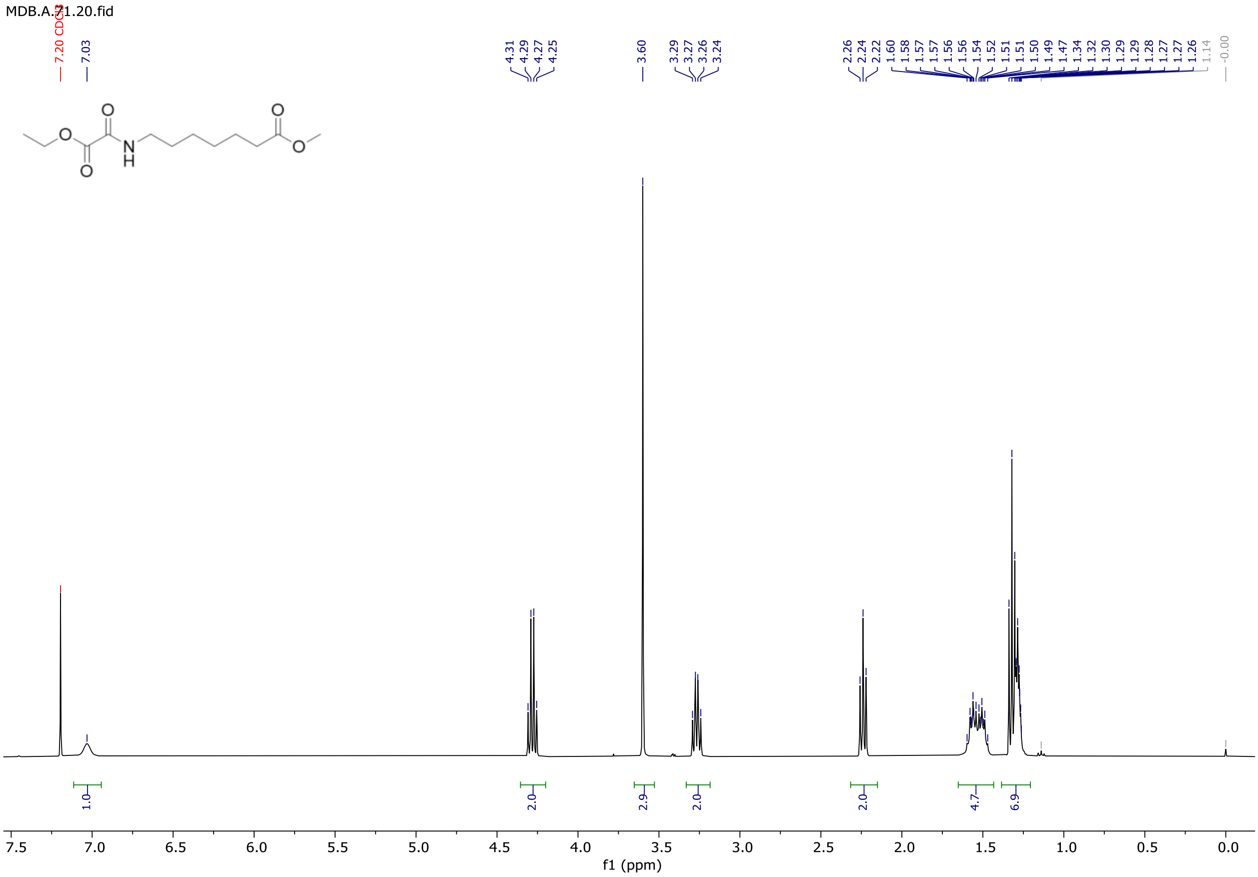

Figure S-49 ^{1}H NMR spectrum of **25b**.

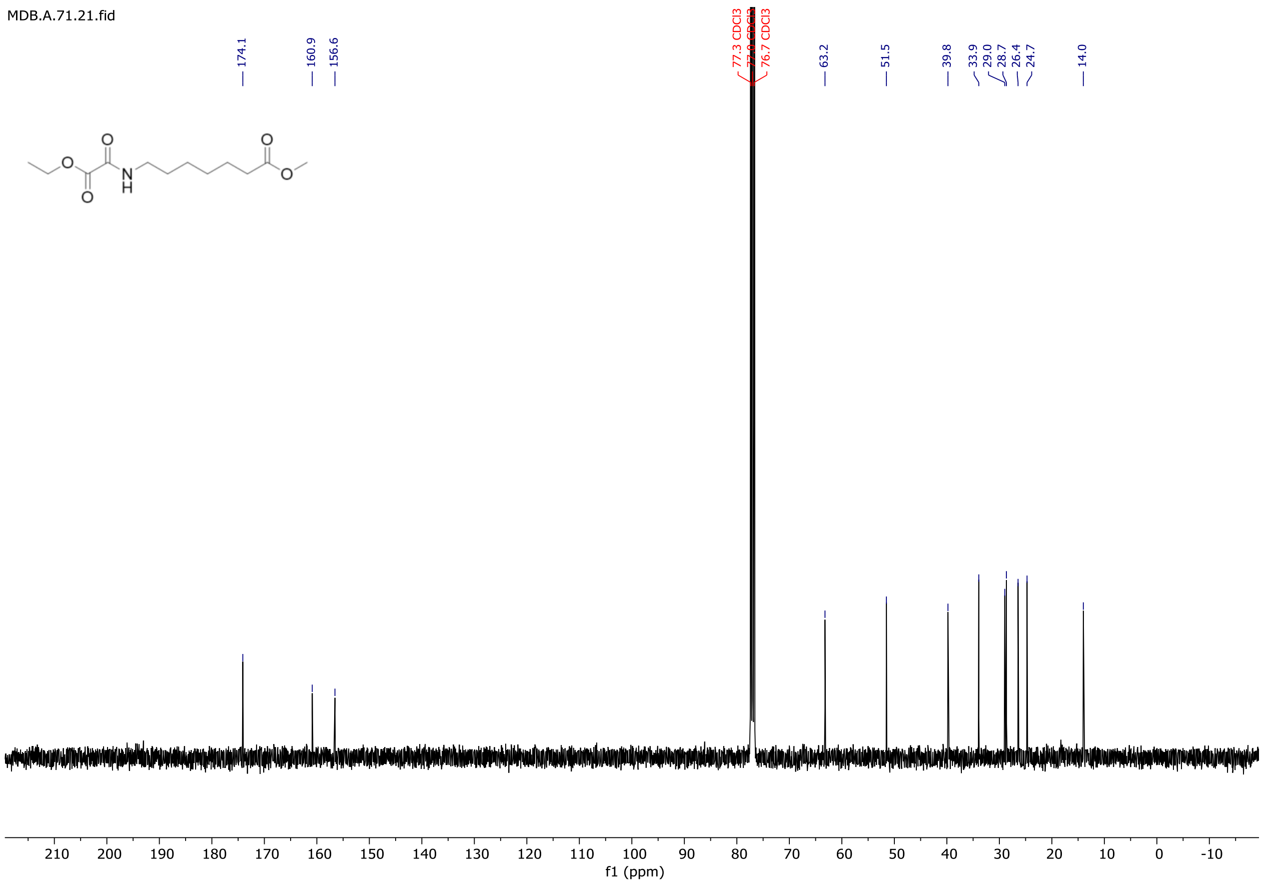

Figure S-50 ^{13}C NMR spectrum of **25b**.

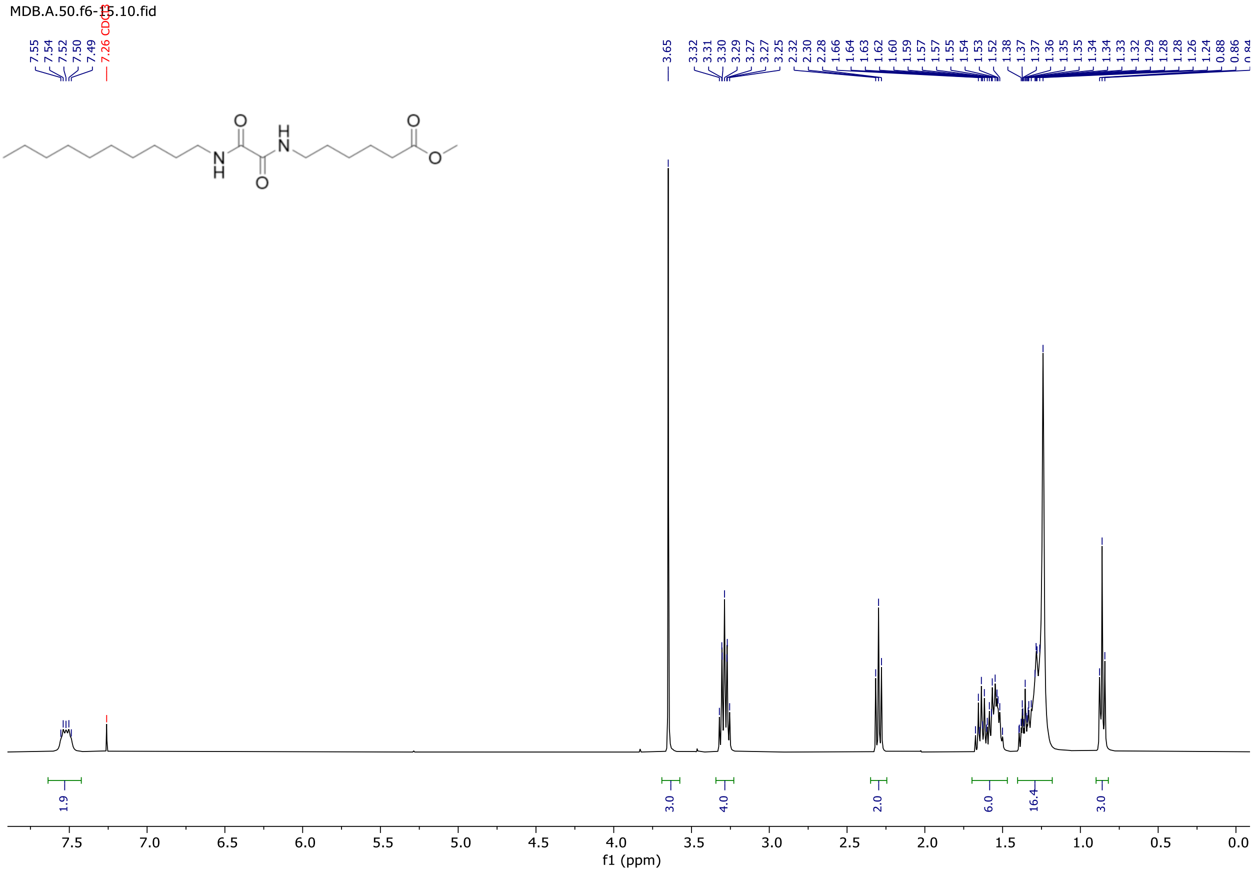

Figure S-51 ^{1}H NMR spectrum of **26a**.

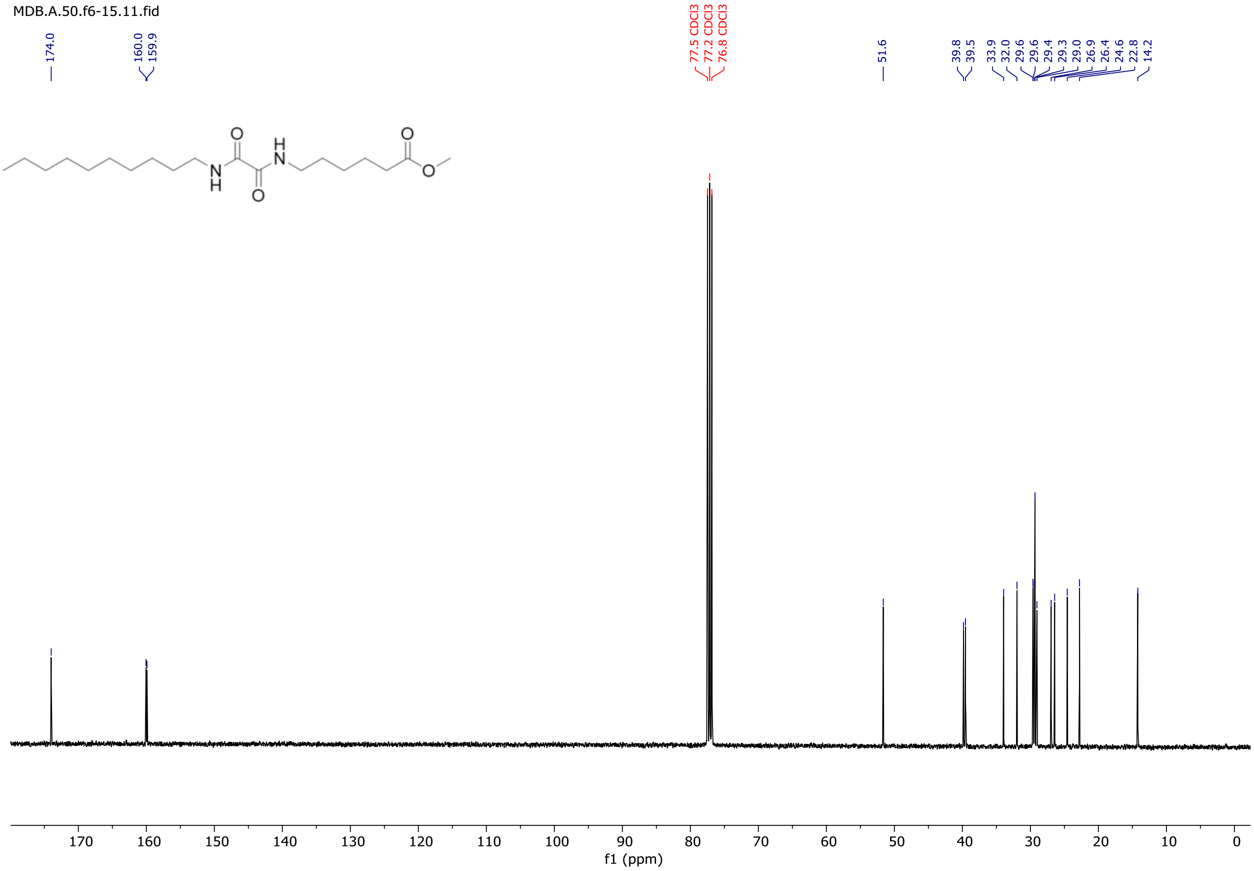

Figure S-52 ^{13}C NMR spectrum of **26a**.

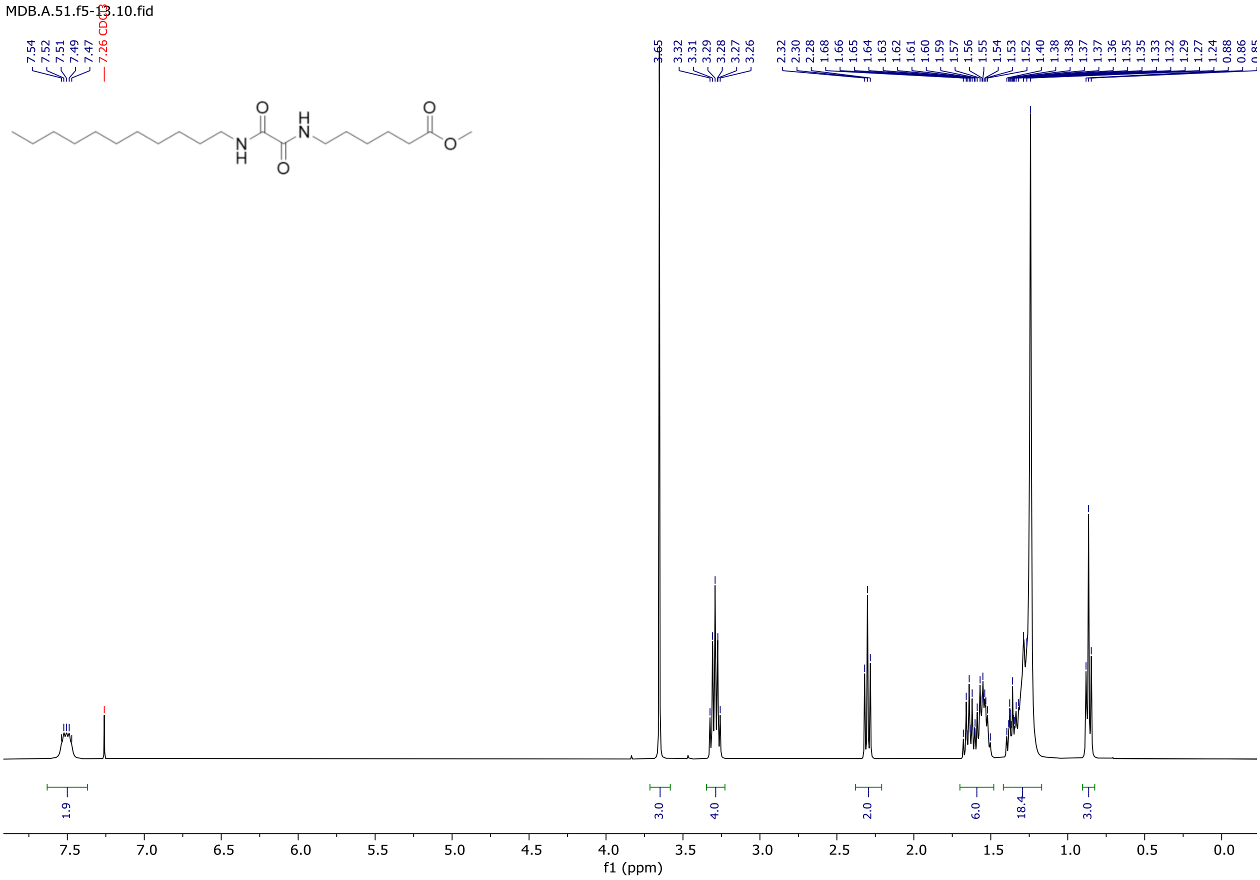

Figure S-53 ^{1}H NMR spectrum of **26b**.

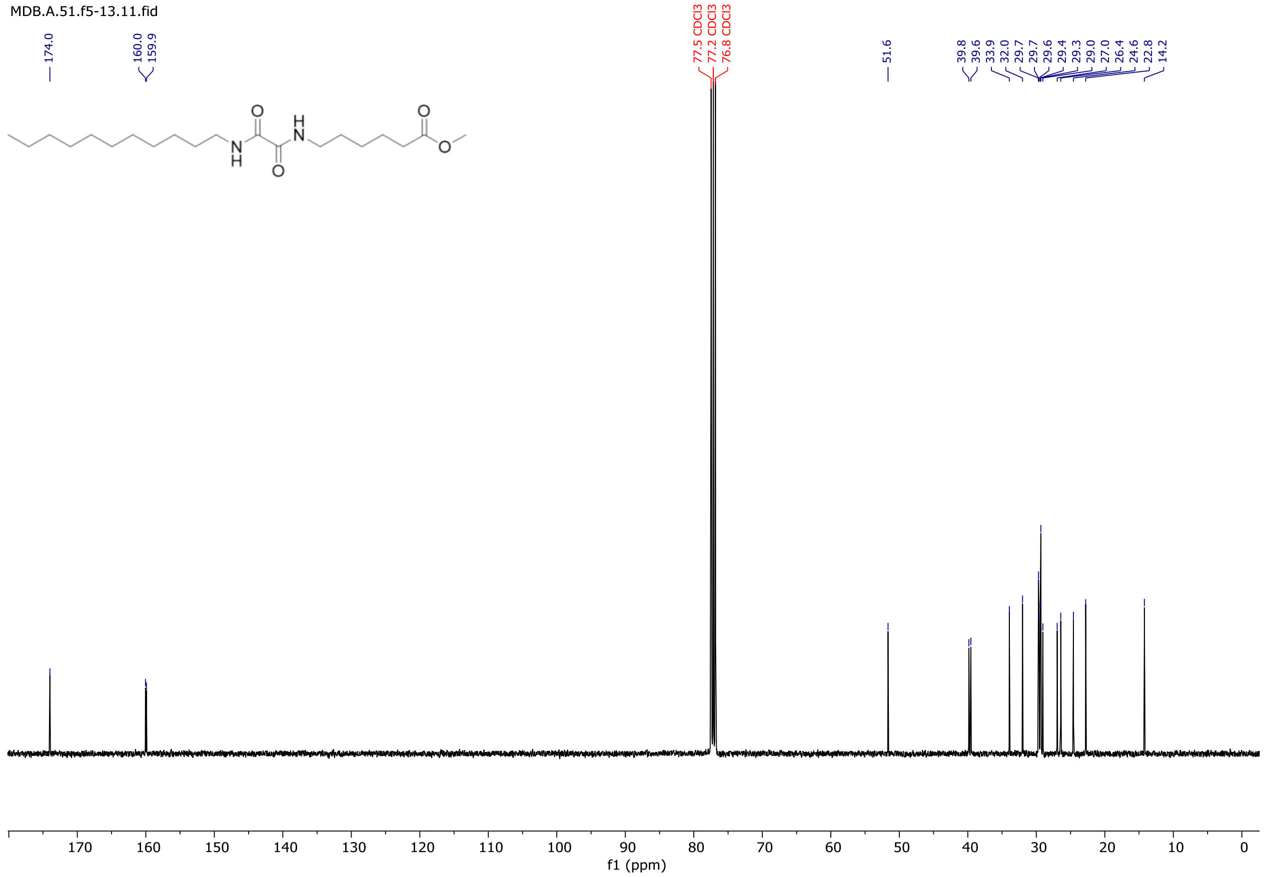

Figure S-54 ^{13}C NMR spectrum of **26b**.

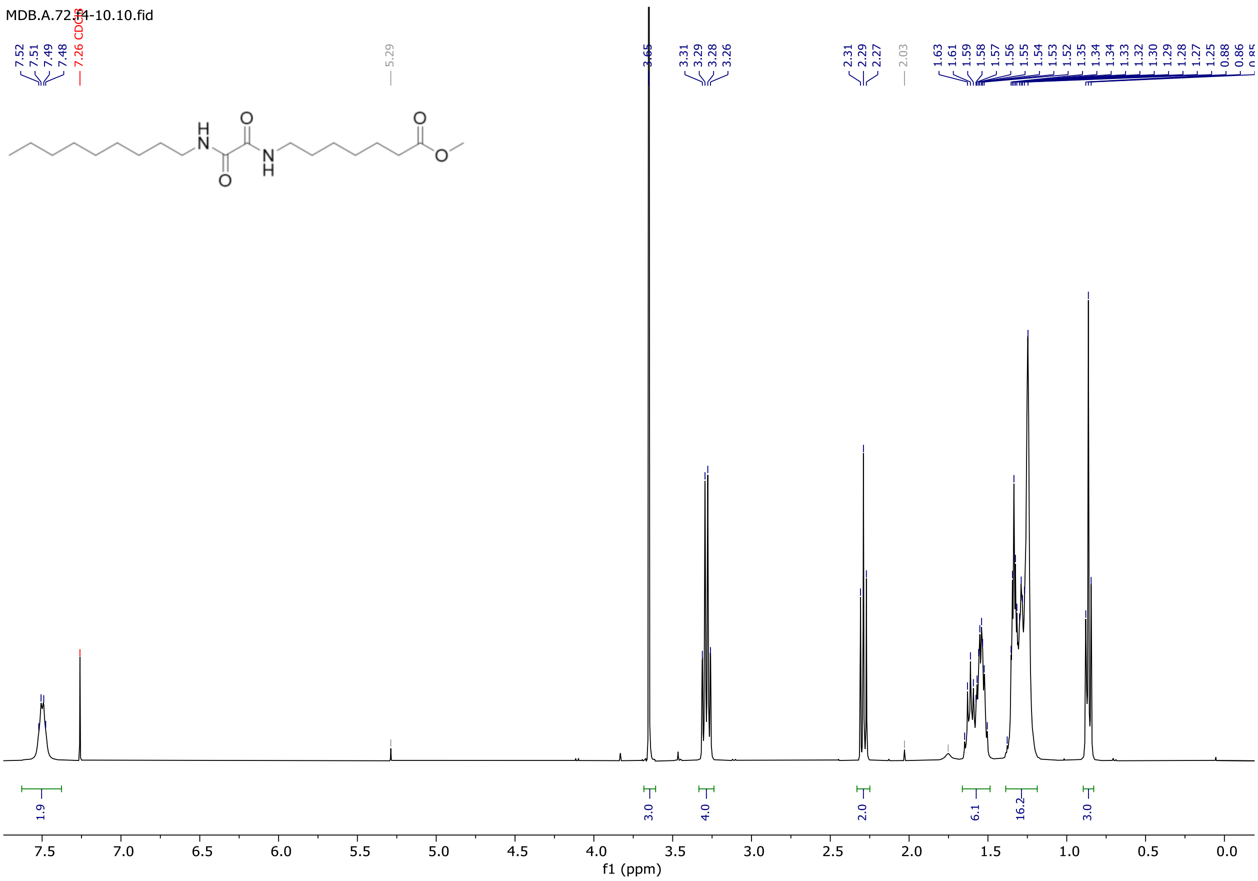

Figure S-55 ^{1}H NMR spectrum of **26c**.

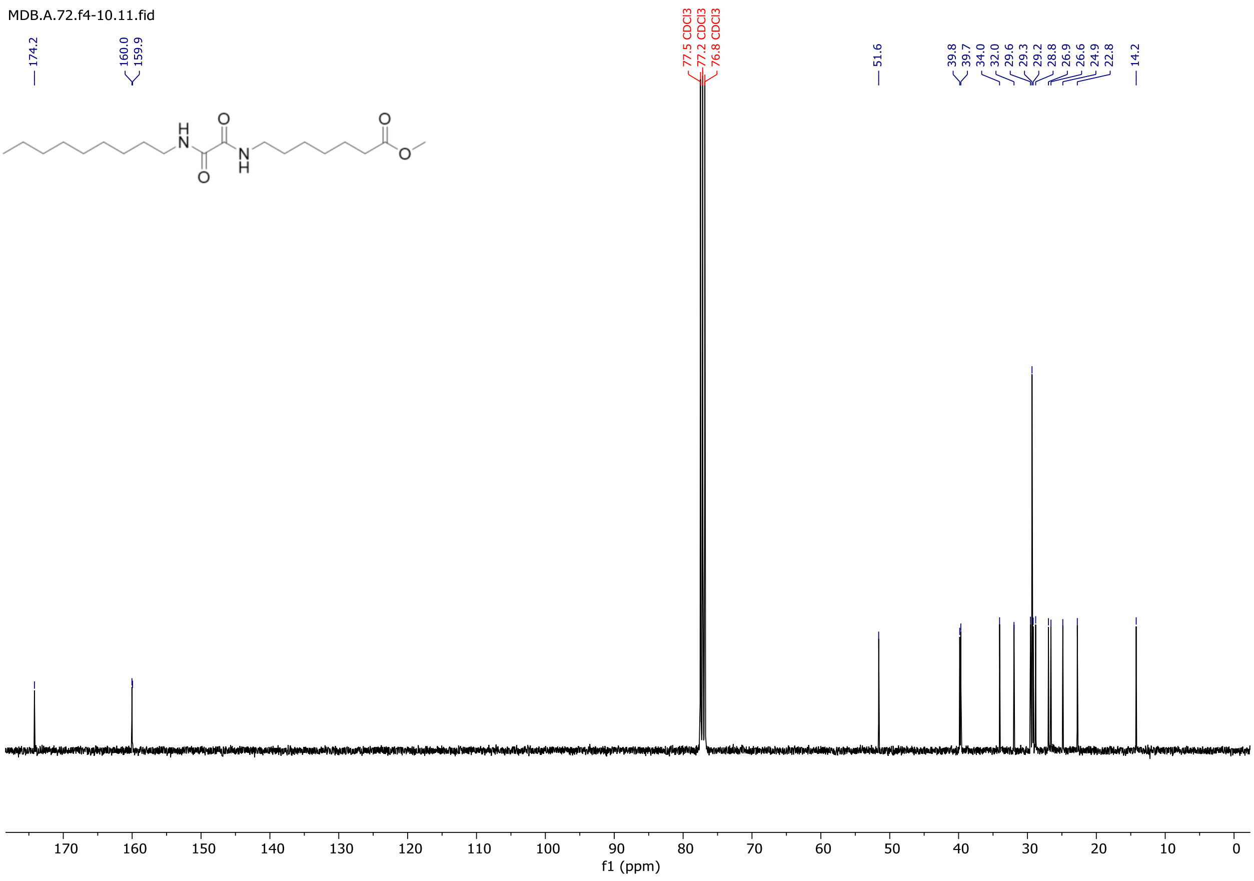

Figure S-56 ^{13}C NMR spectrum of **26c**.

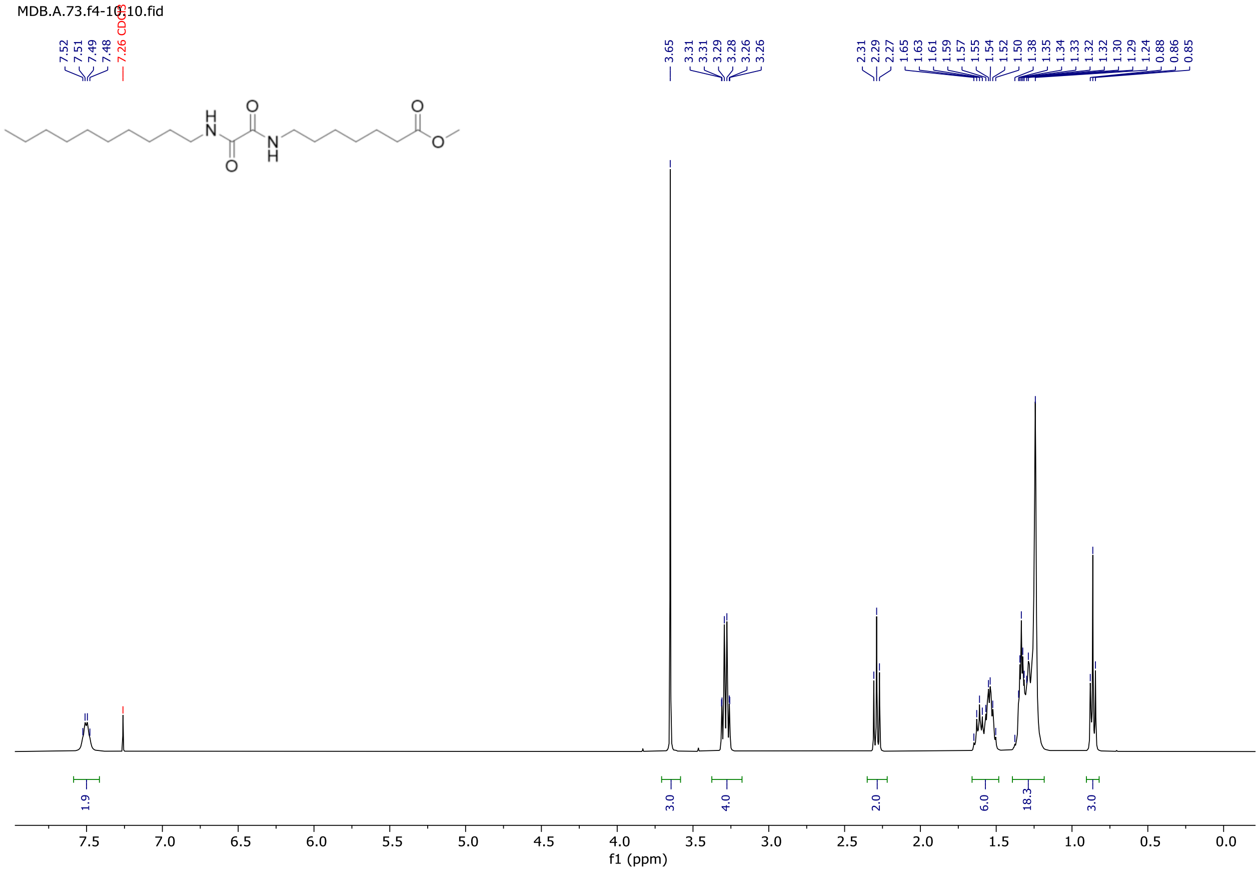

Figure S-57 ^{1}H NMR spectrum of **26d**.

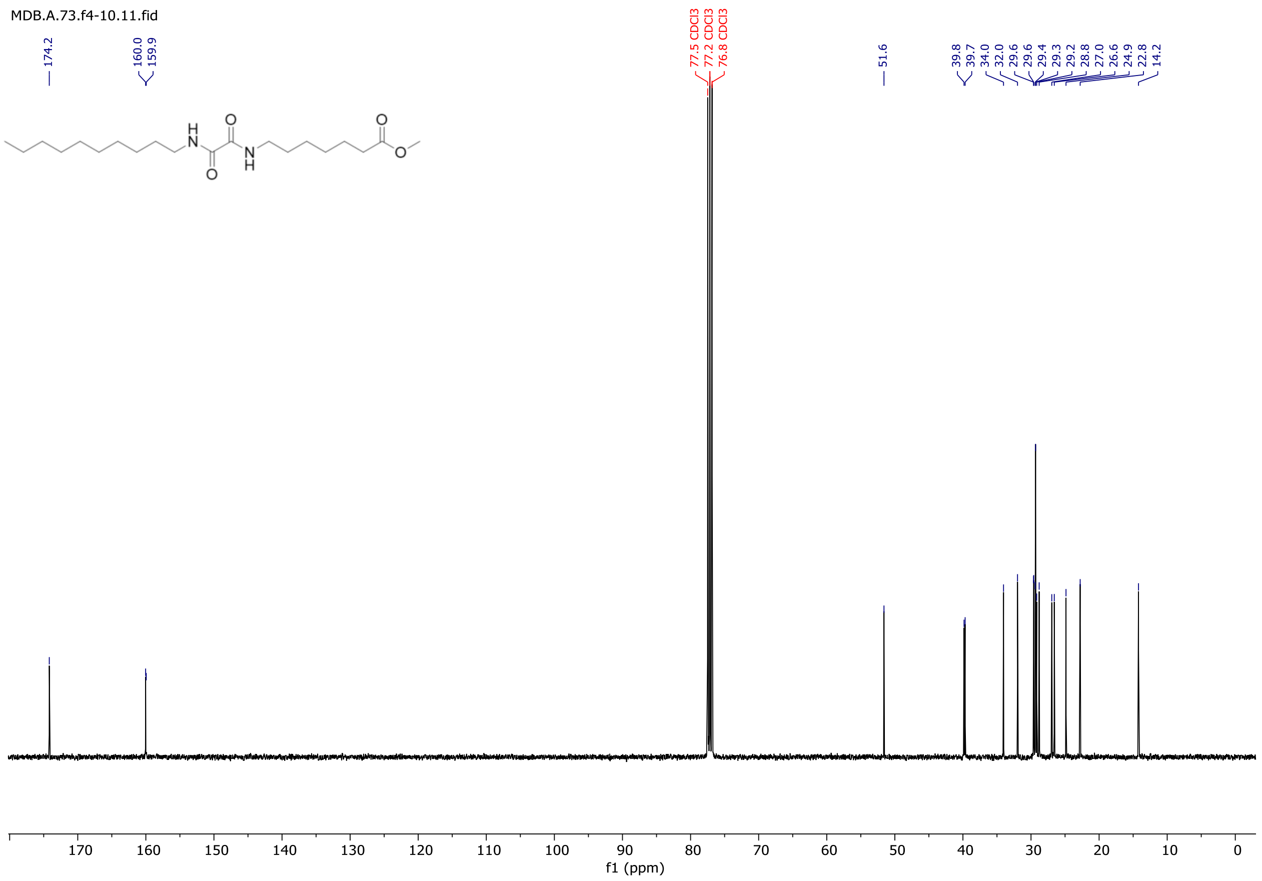

Figure S-58 ^{13}C NMR spectrum of **26d**.

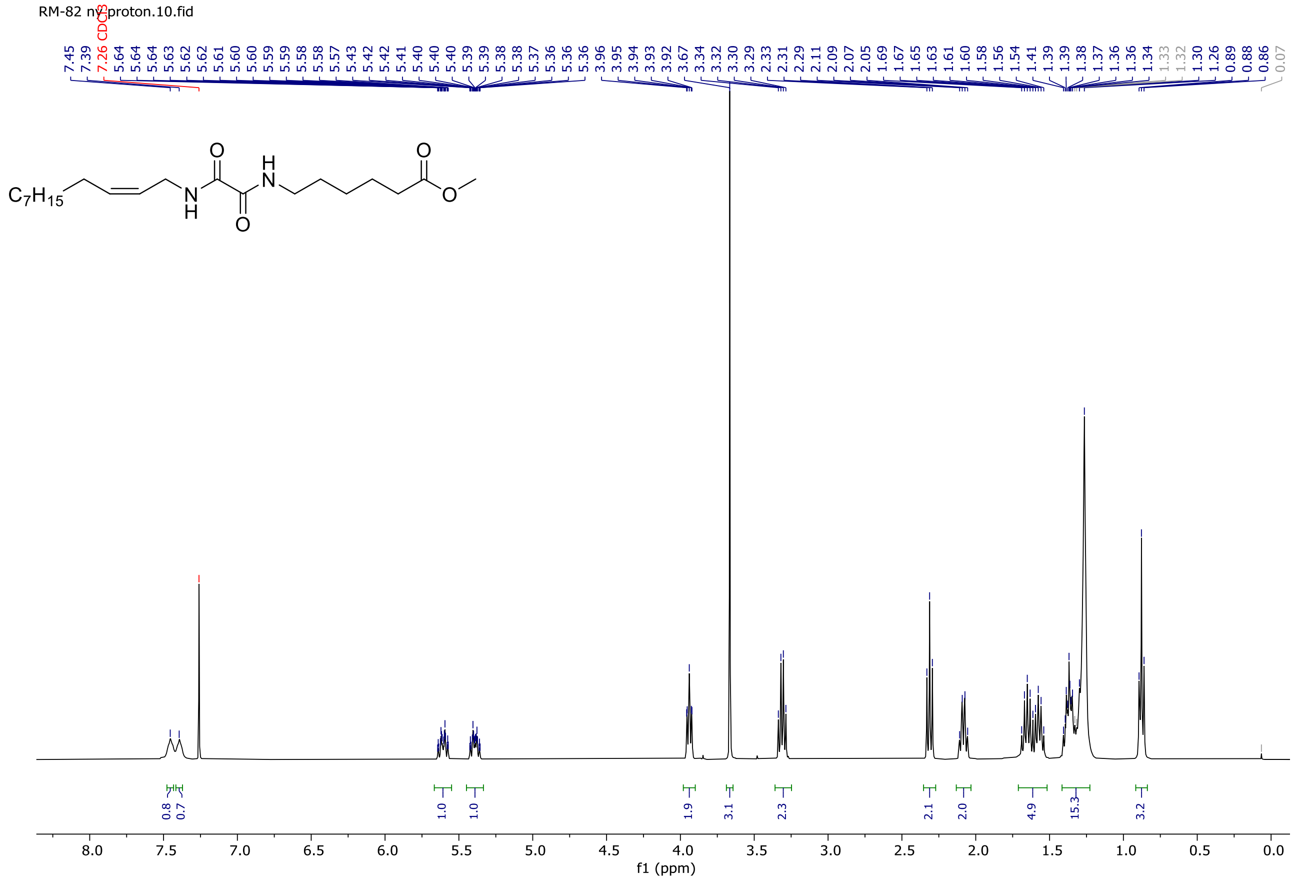

Figure S-59 ^{1}H NMR spectrum of **26e**.

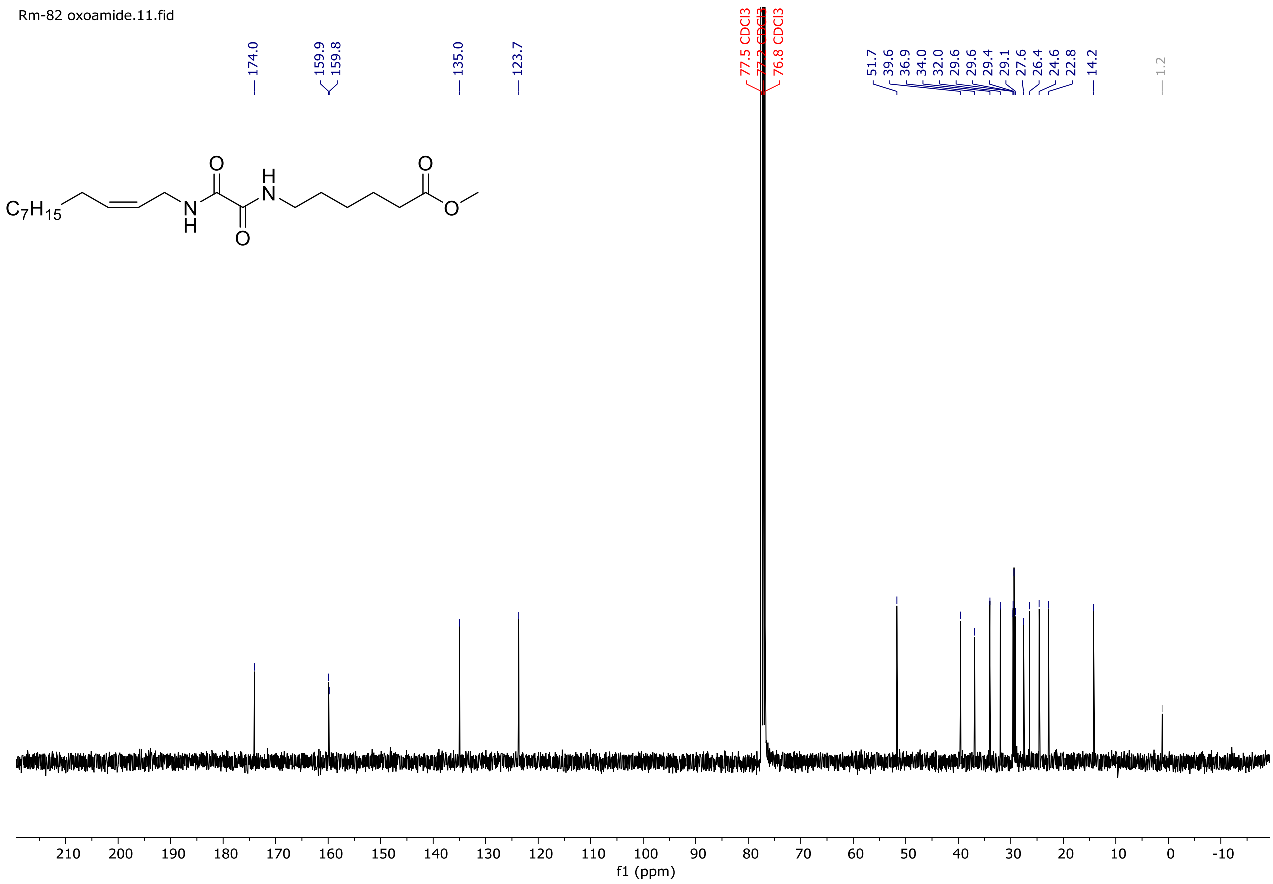

Figure S-60 ^{13}C NMR spectrum of **26e**.

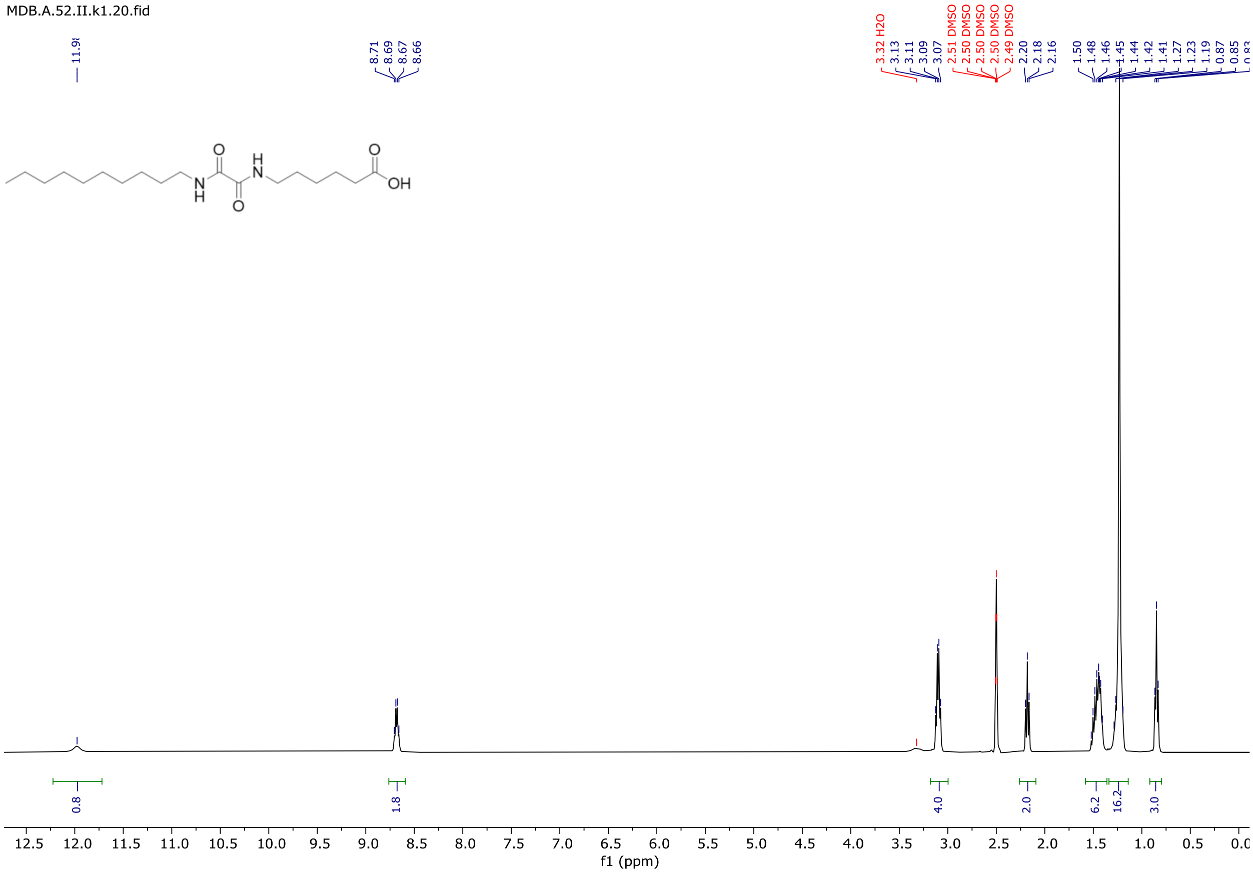

Figure S-61 ^{1}H NMR spectrum of **8a**.

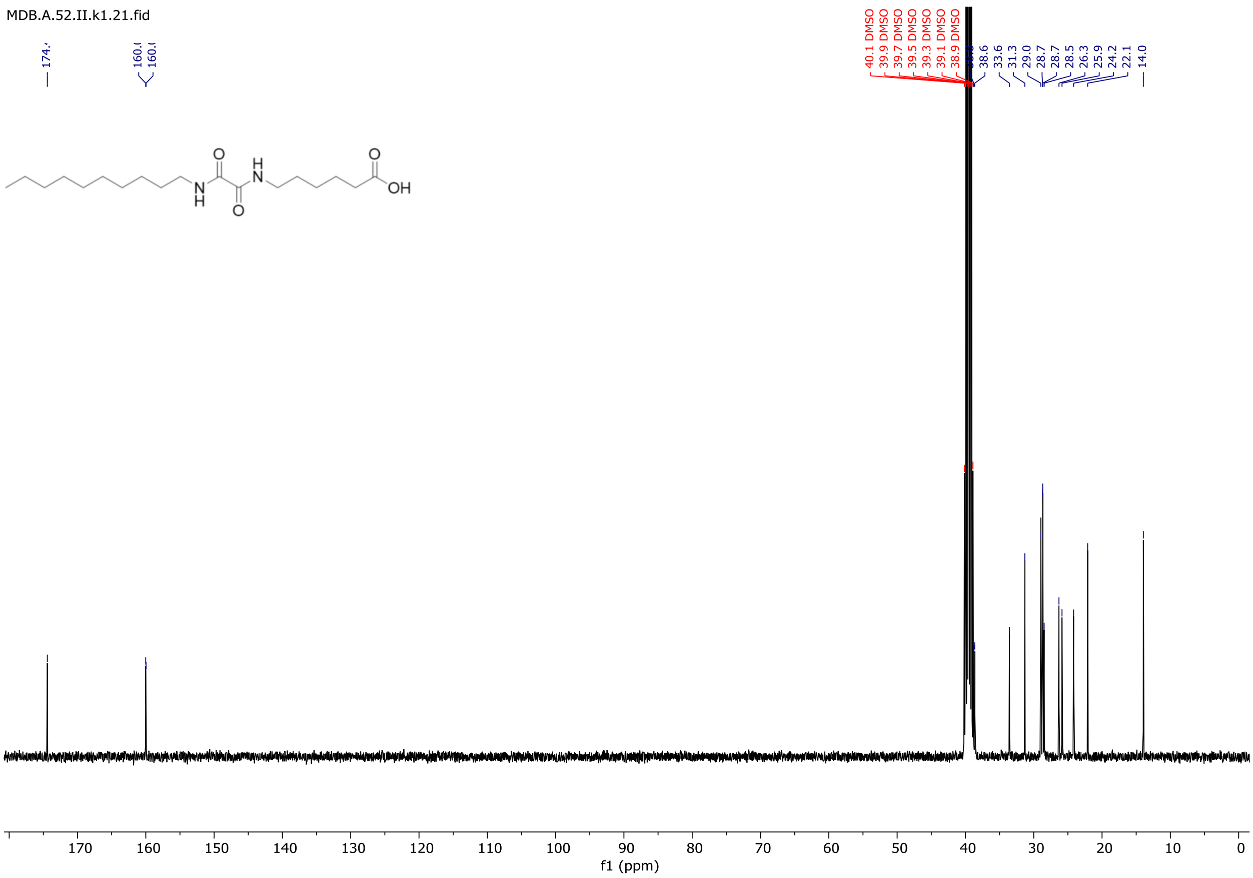

Figure S-62 ^{13}C NMR spectrum of **8a**.

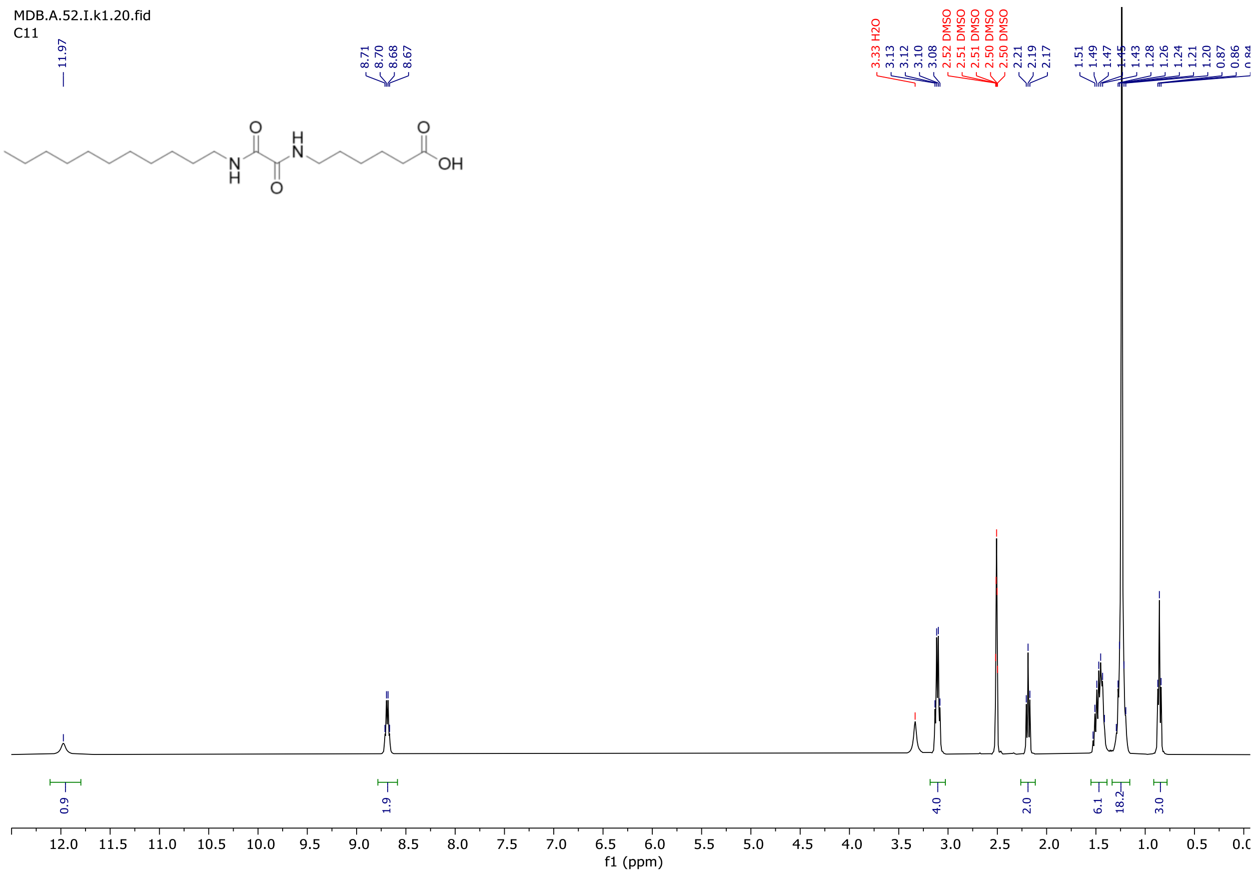

Figure S-63 ^{1}H NMR spectrum of **8b**.

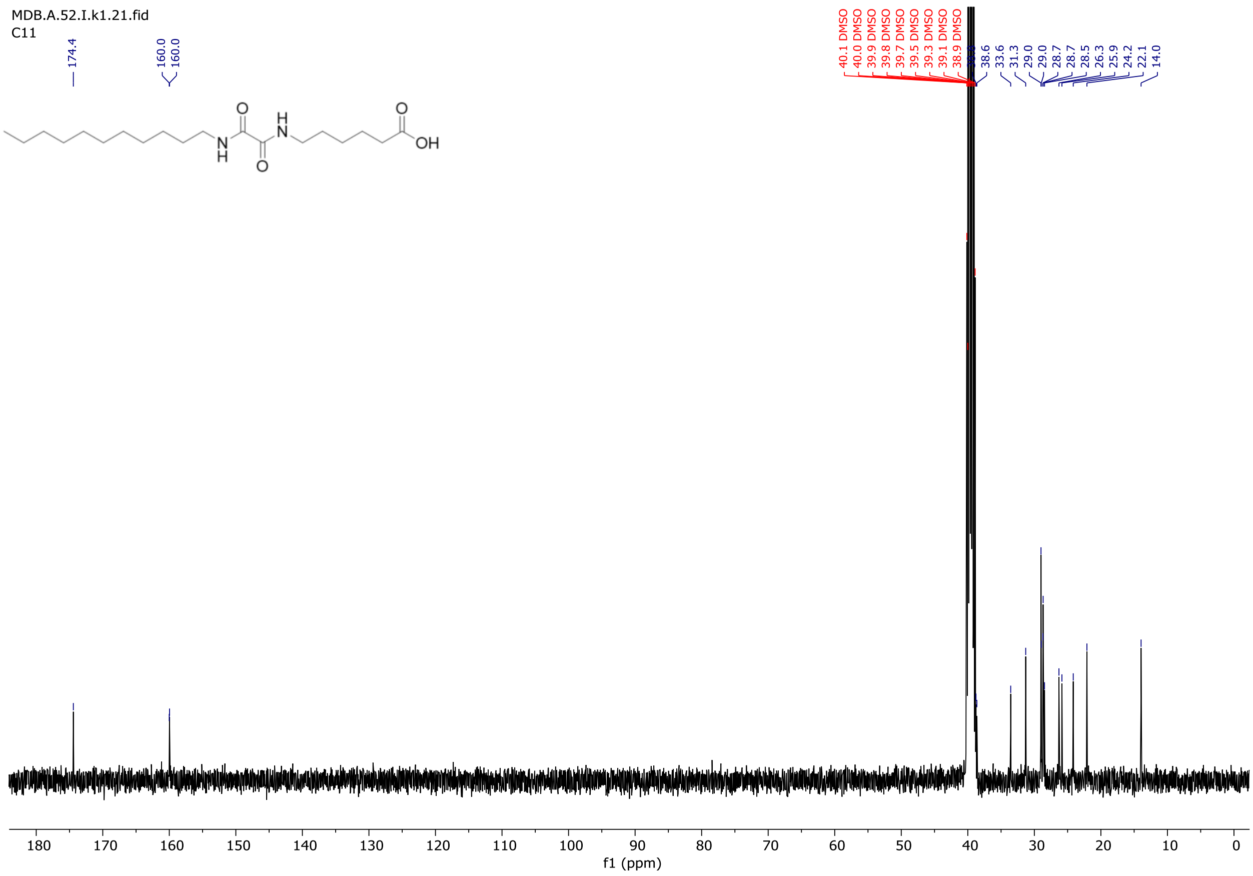

Figure S-64 ^{13}C NMR spectrum of **8b**.

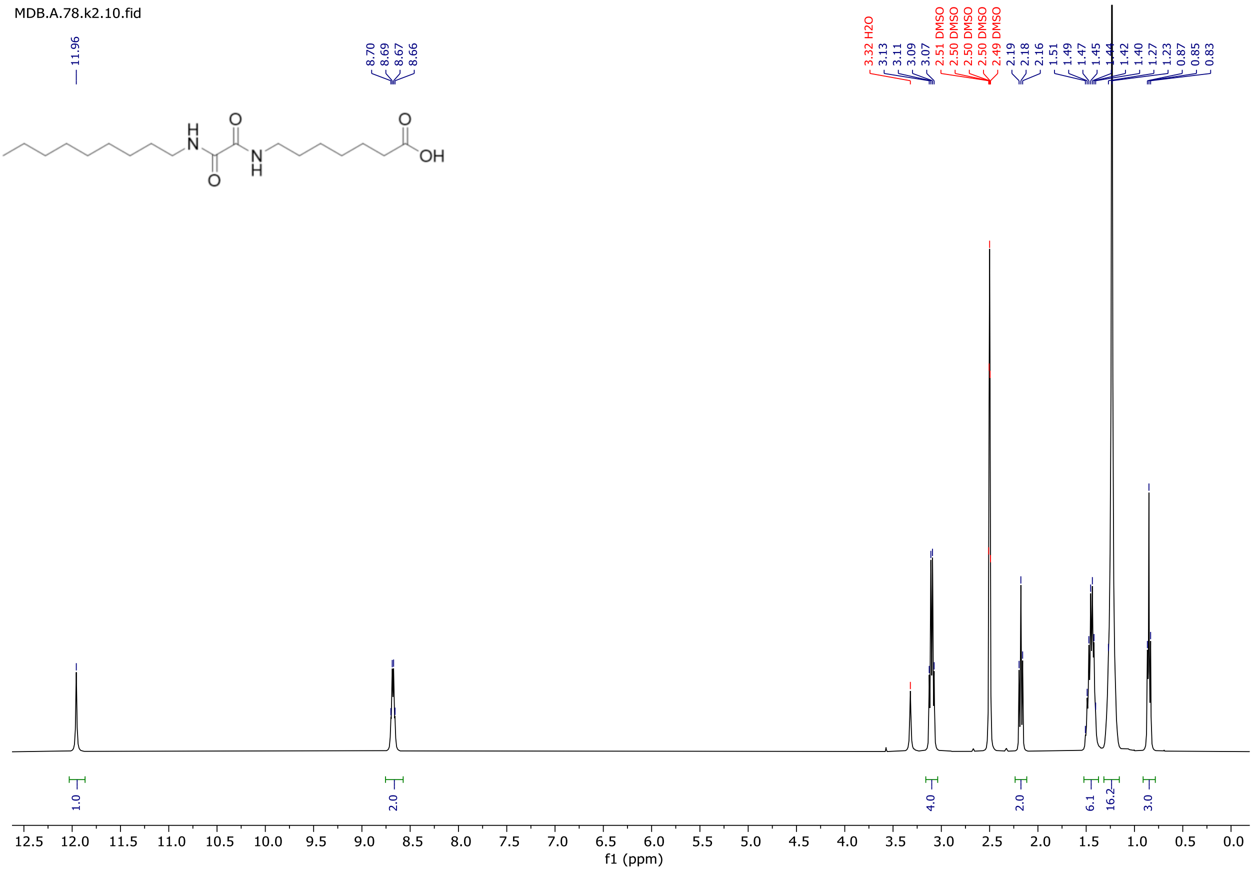

Figure S-65 ^{1}H NMR spectrum of **8c**.

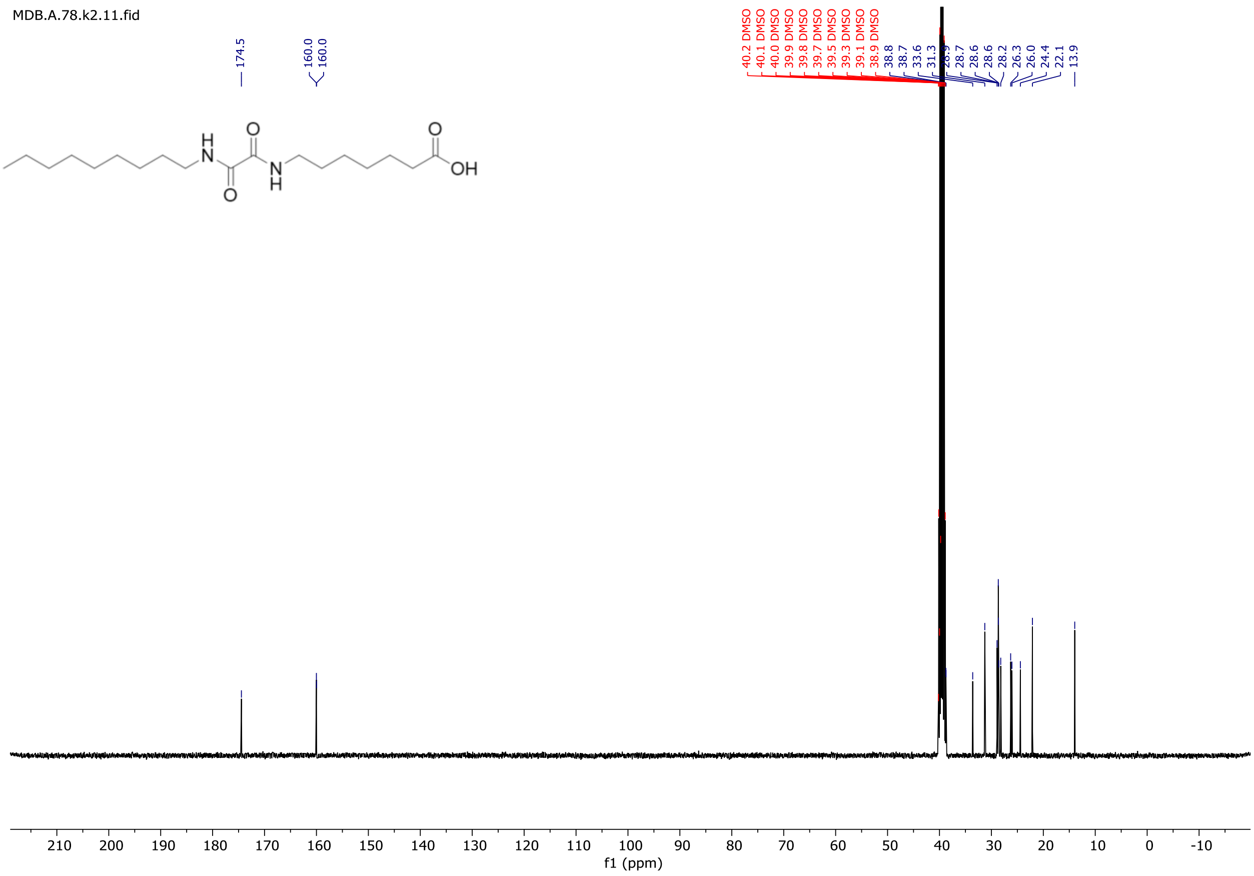

Figure S-66 ^{13}C NMR spectrum of **8c**.

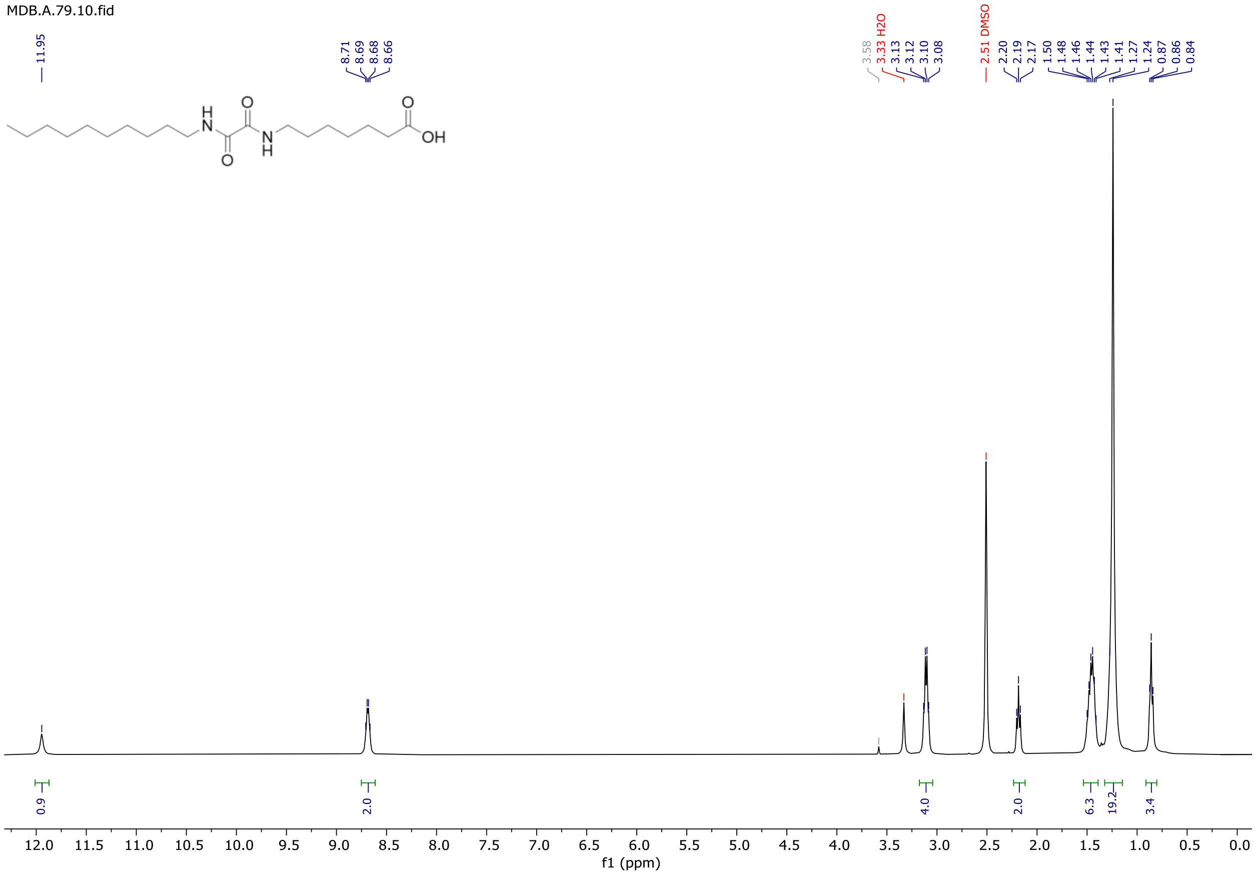

Figure S-67 ^{1}H NMR spectrum of **8d**.

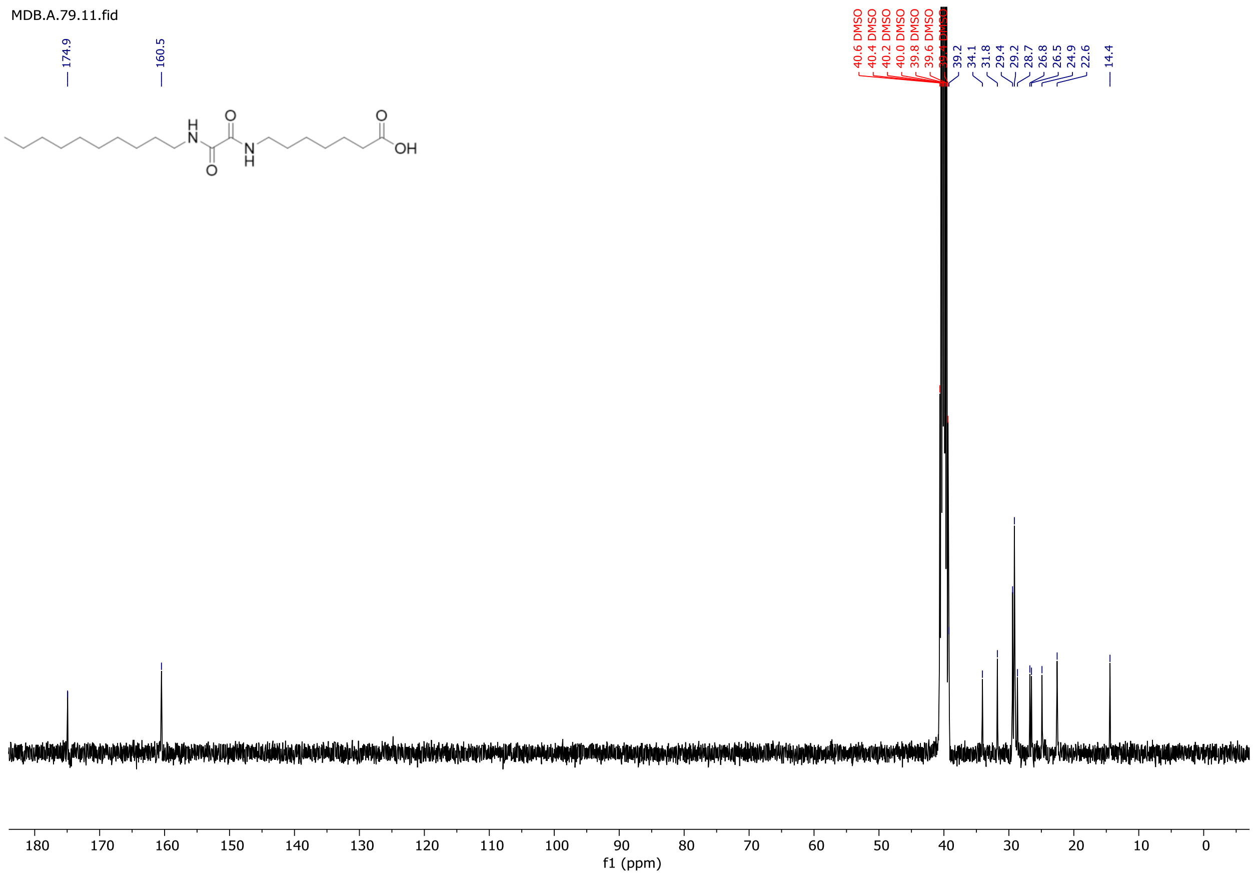

Figure S-68 ^{13}C NMR spectrum of **8d**.

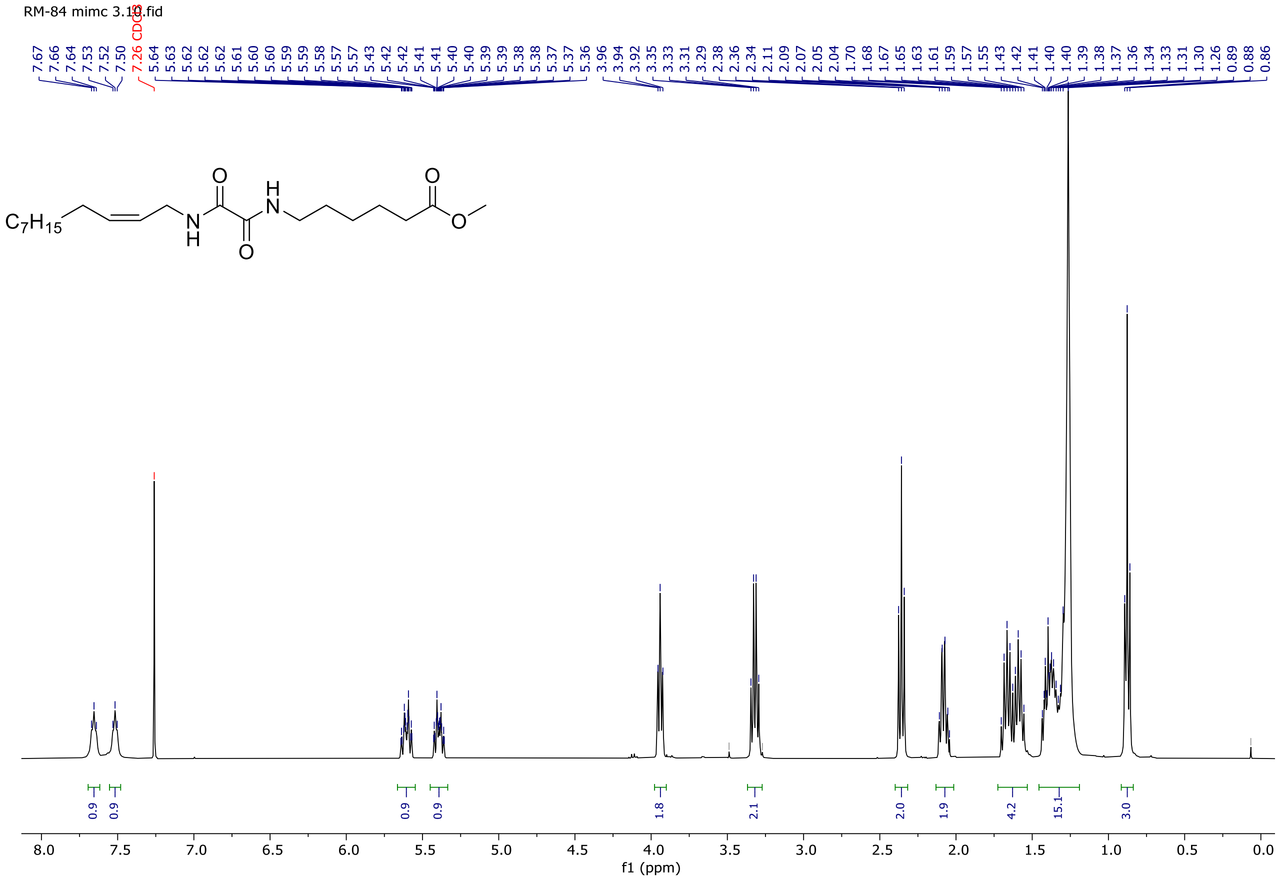

Figure S-69 ^{1}H NMR spectrum of **8e**.

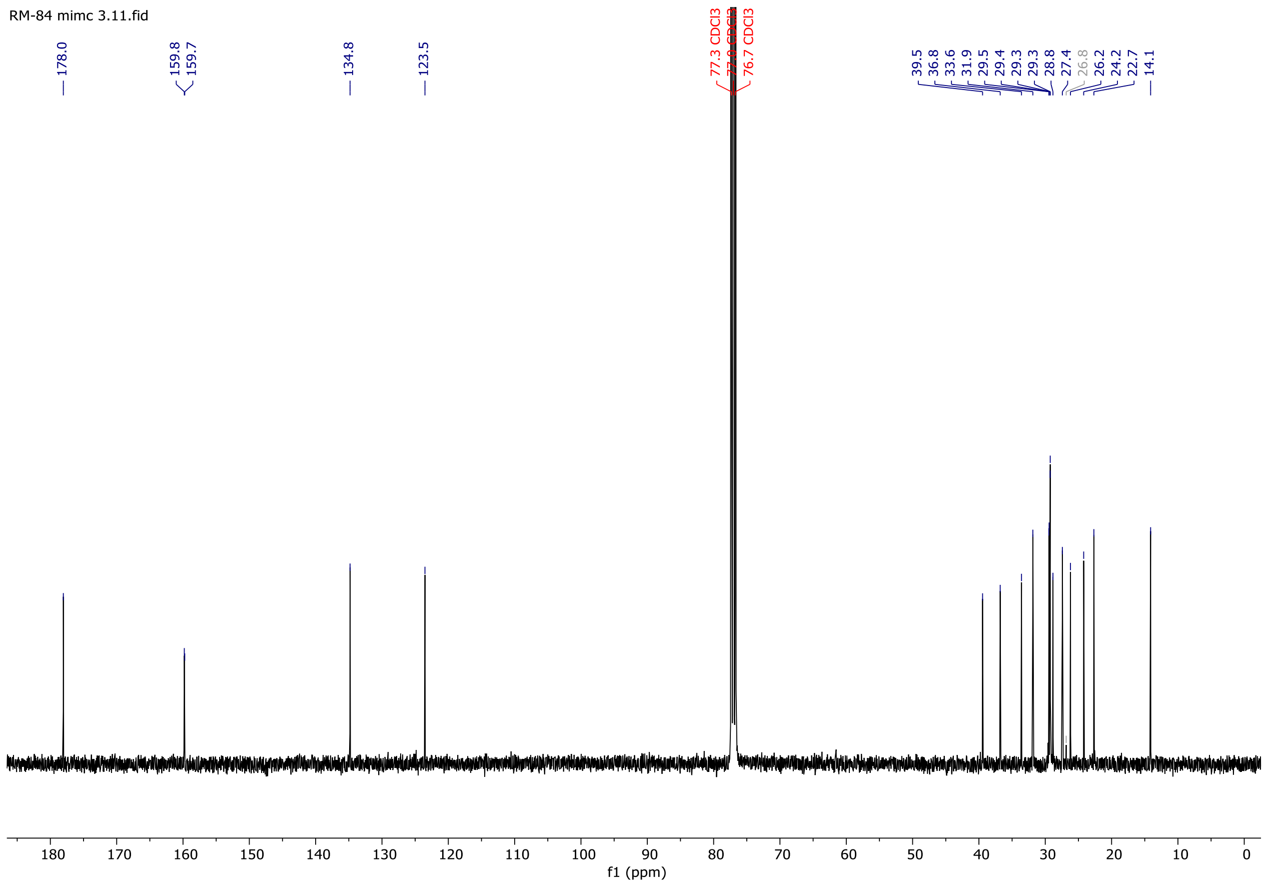

Figure S-70 ^{13}C NMR spectrum of **8e**.

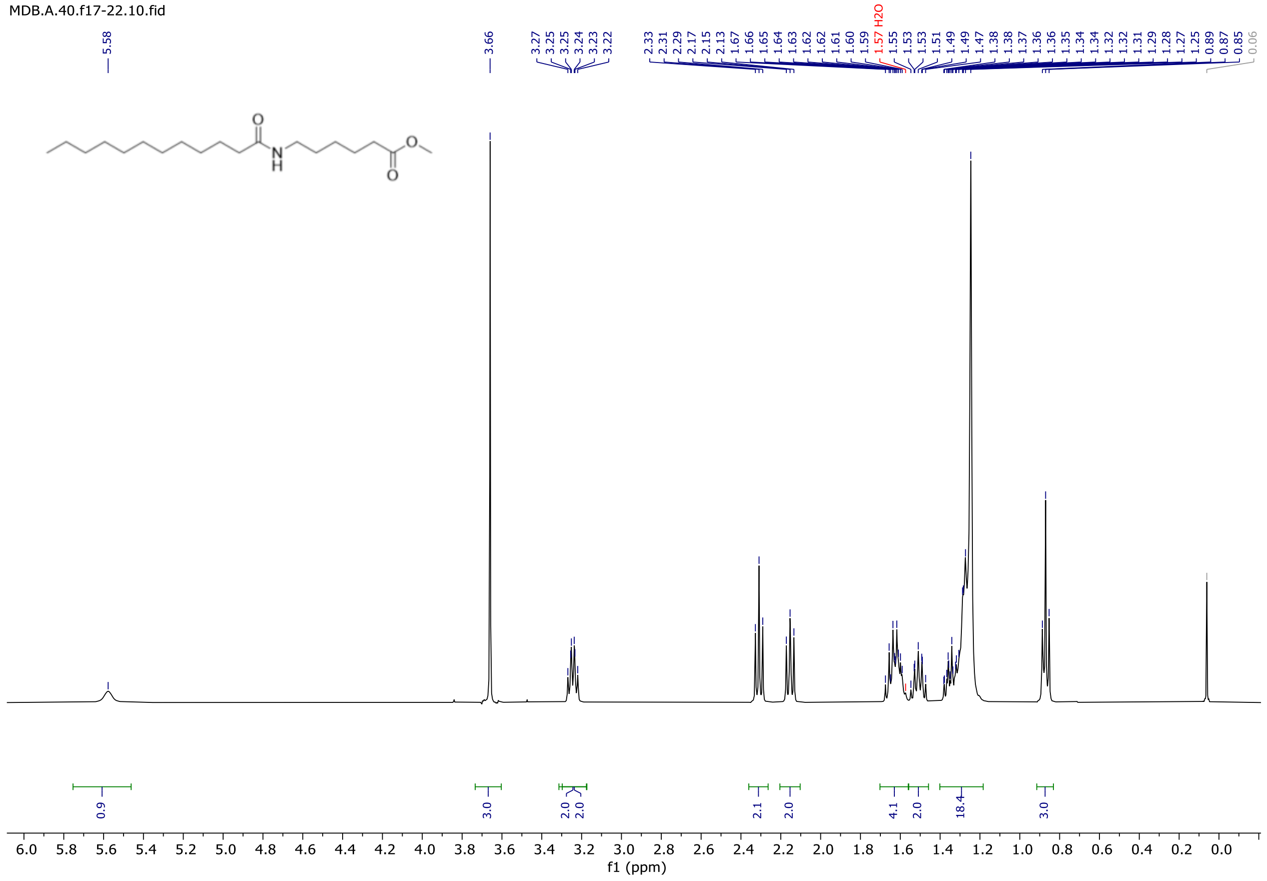

Figure S-71 ^{1}H NMR spectrum of **28a**.

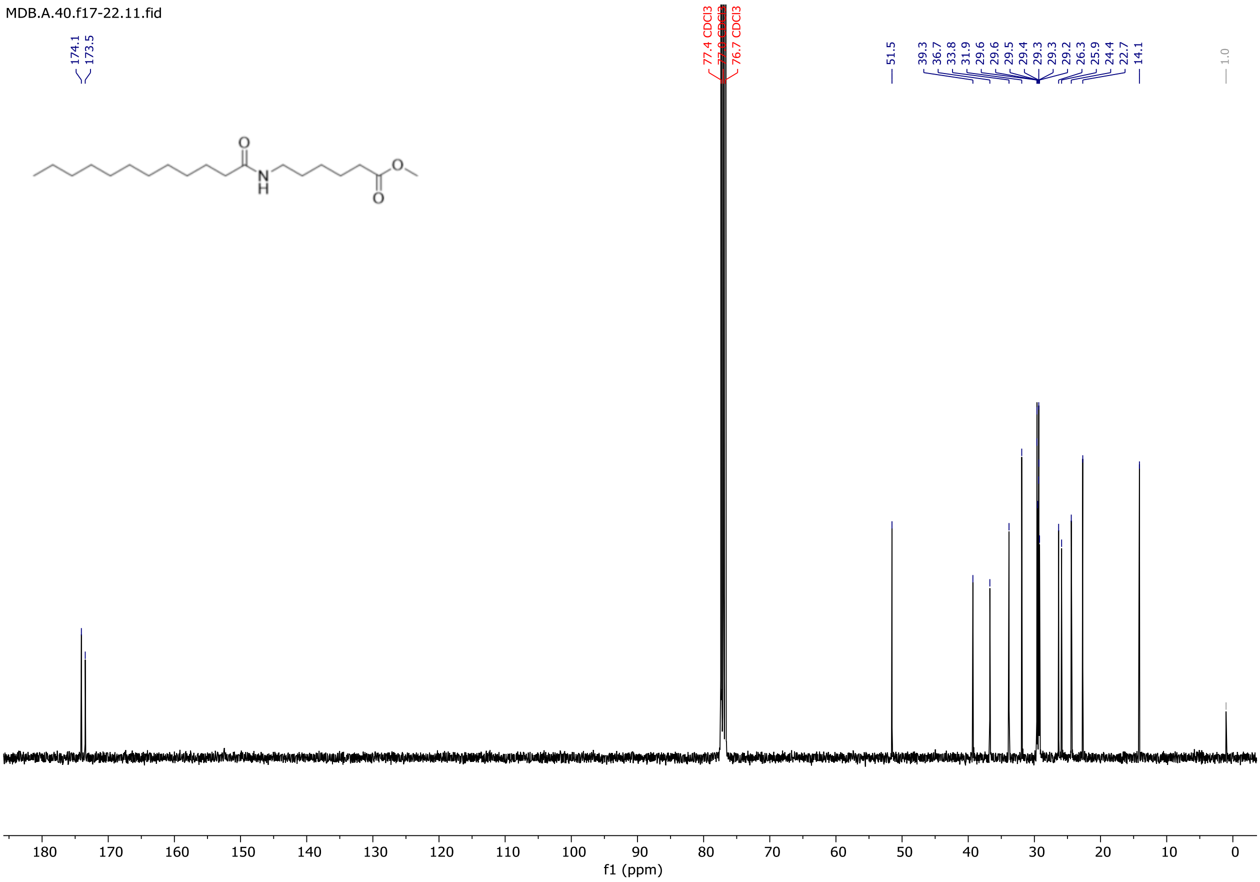

Figure S-72 ^{13}C NMR spectrum of **28a**.

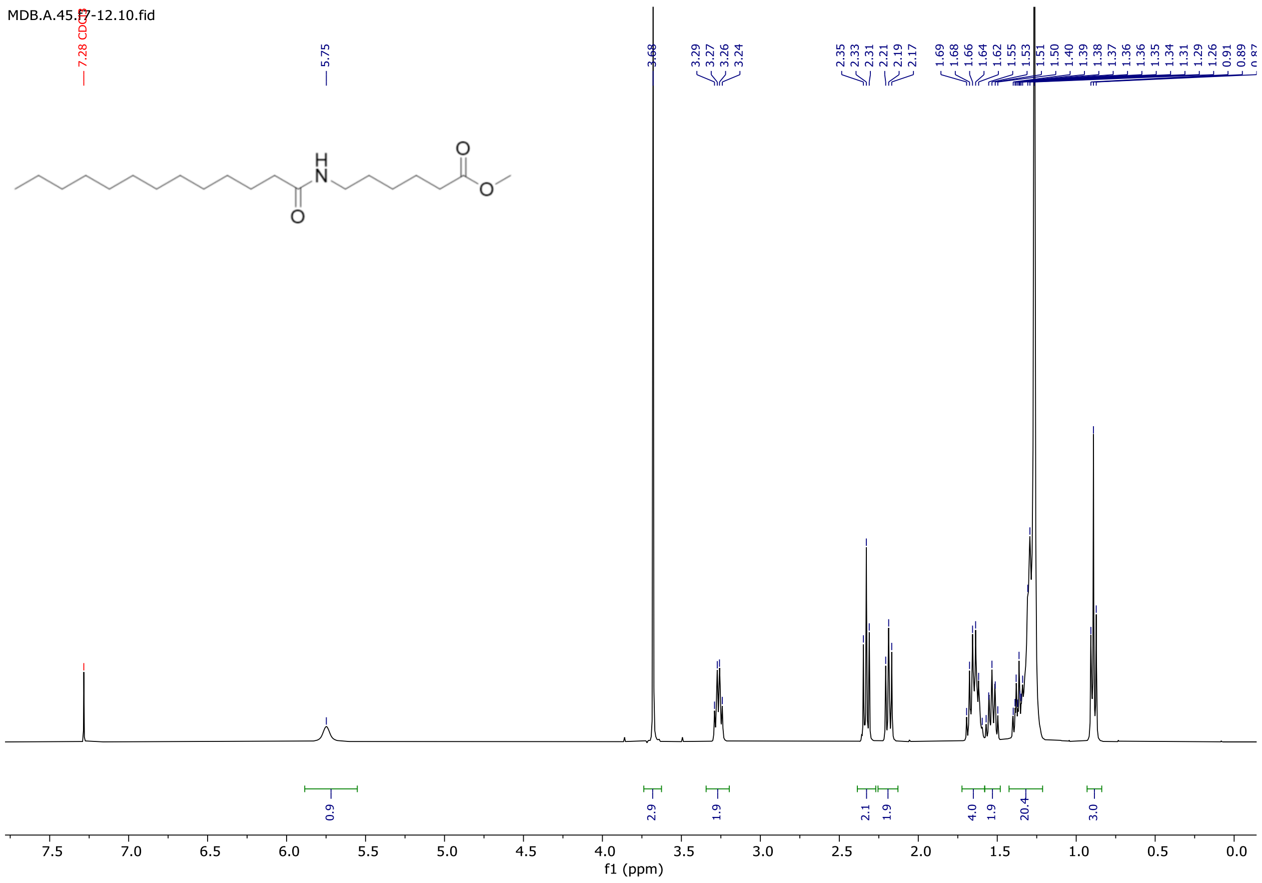

Figure S-73 ^{1}H NMR spectrum of **28b**.

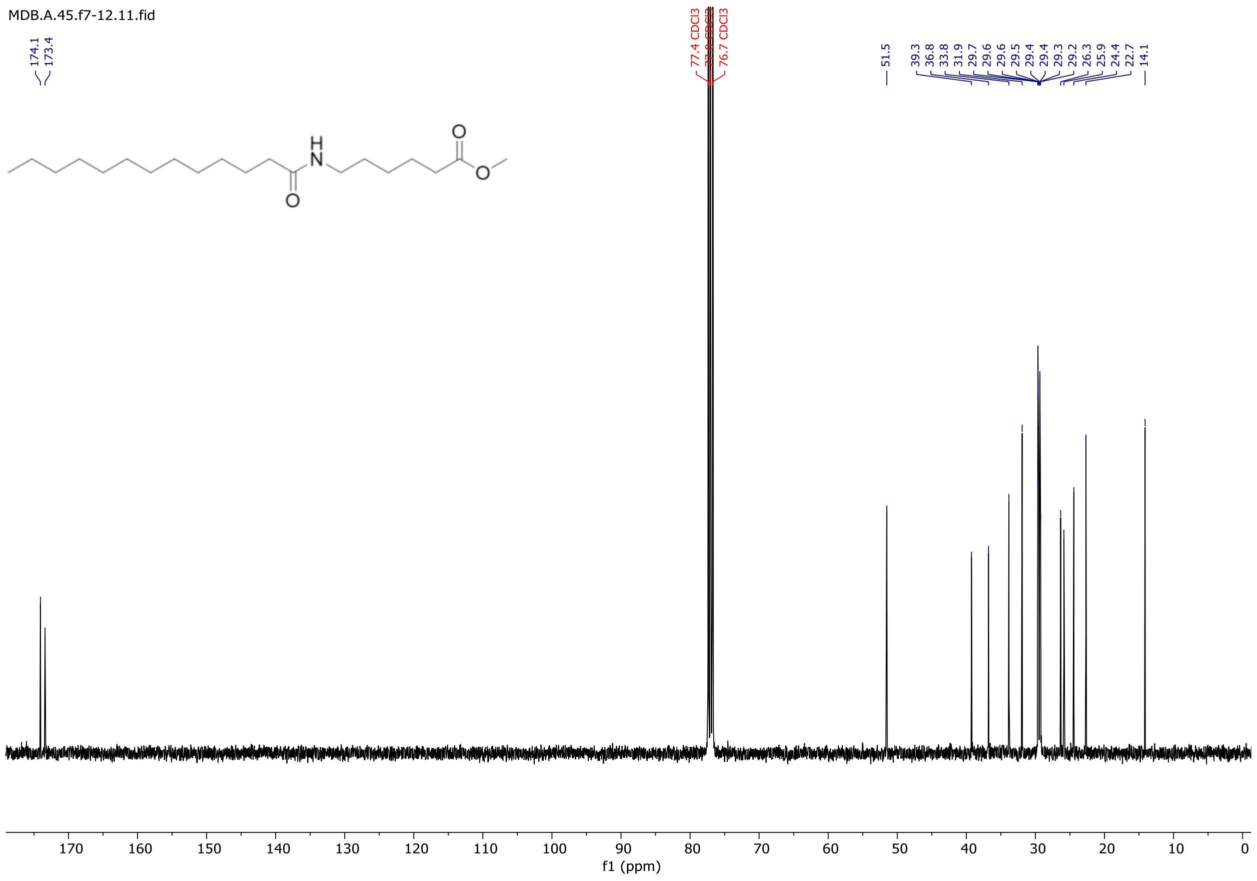

Figure S-74 ^{13}C NMR spectrum of **28b**.

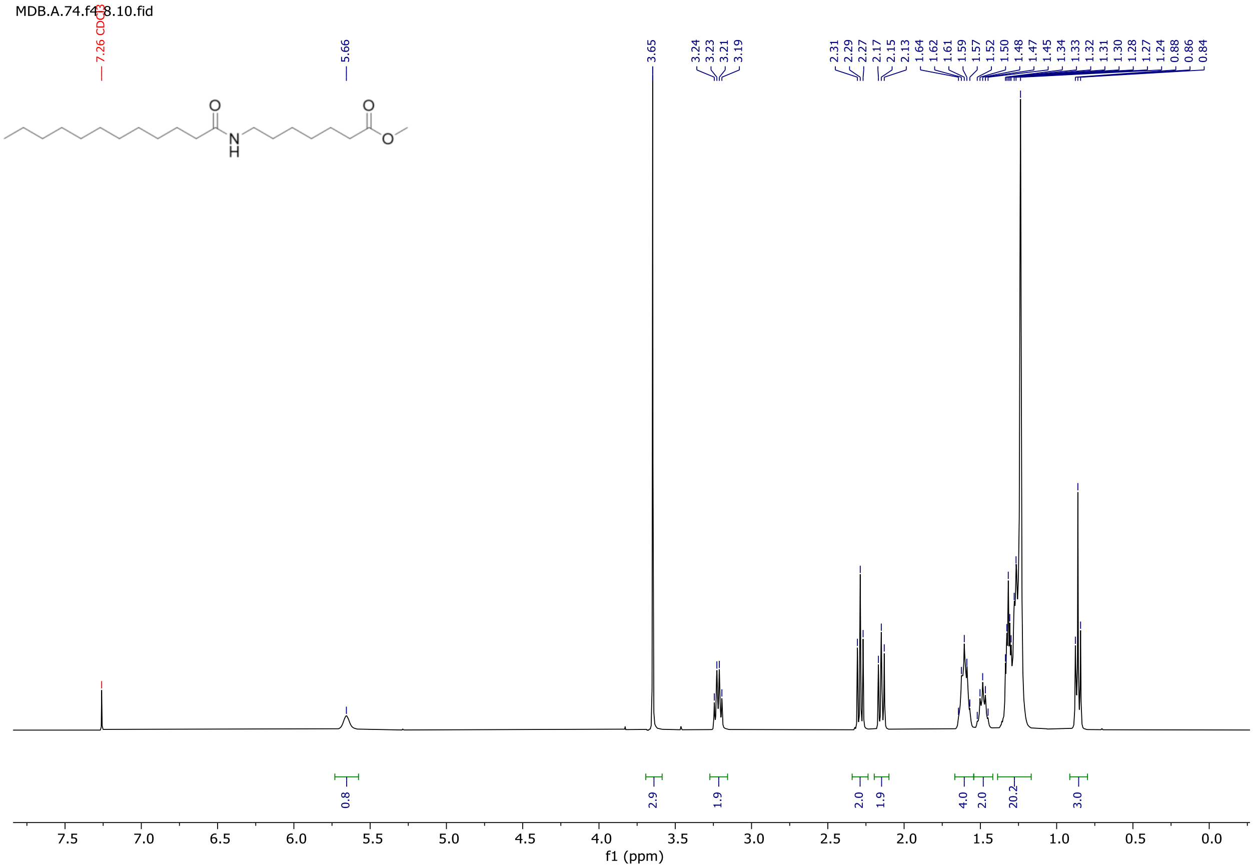

Figure S-75 ^{1}H NMR spectrum of **28c**.

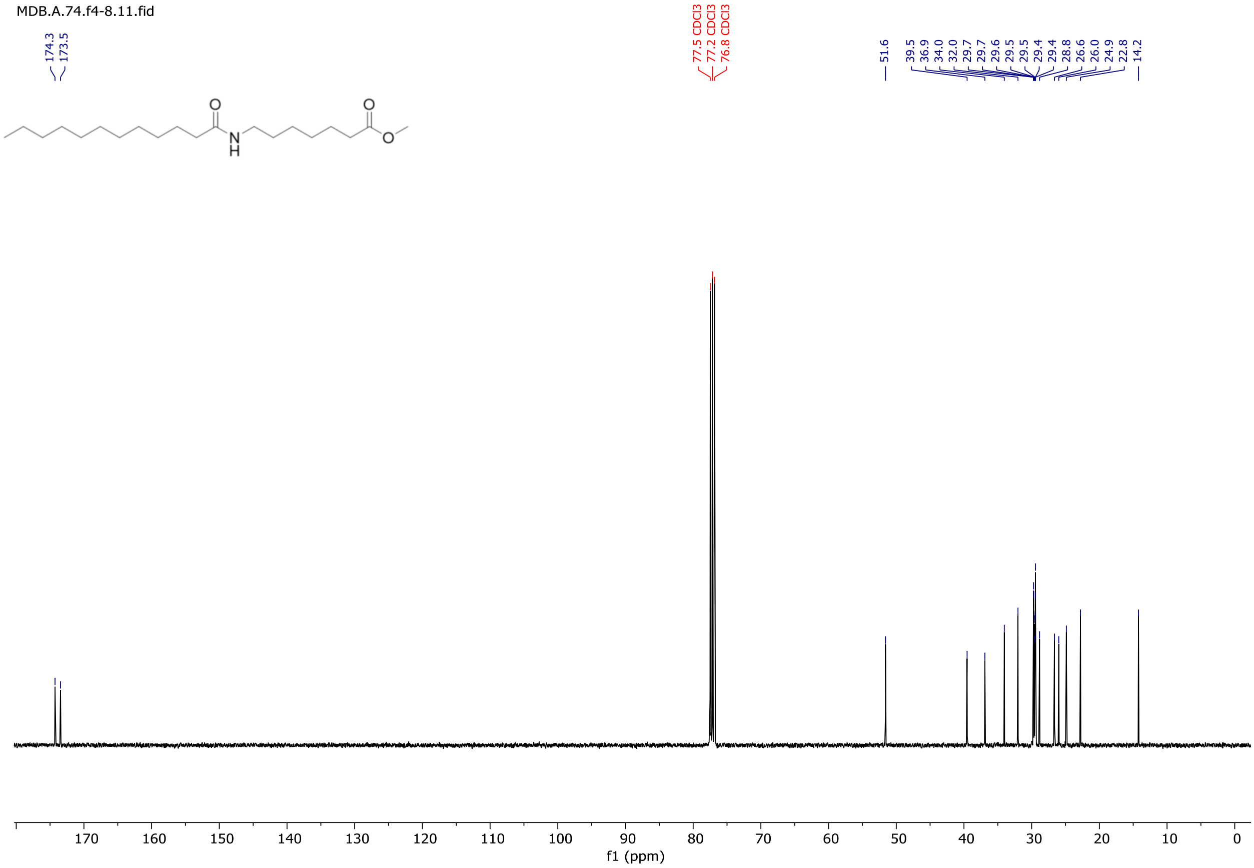

Figure S-76 ^{13}C NMR spectrum of **28c**.

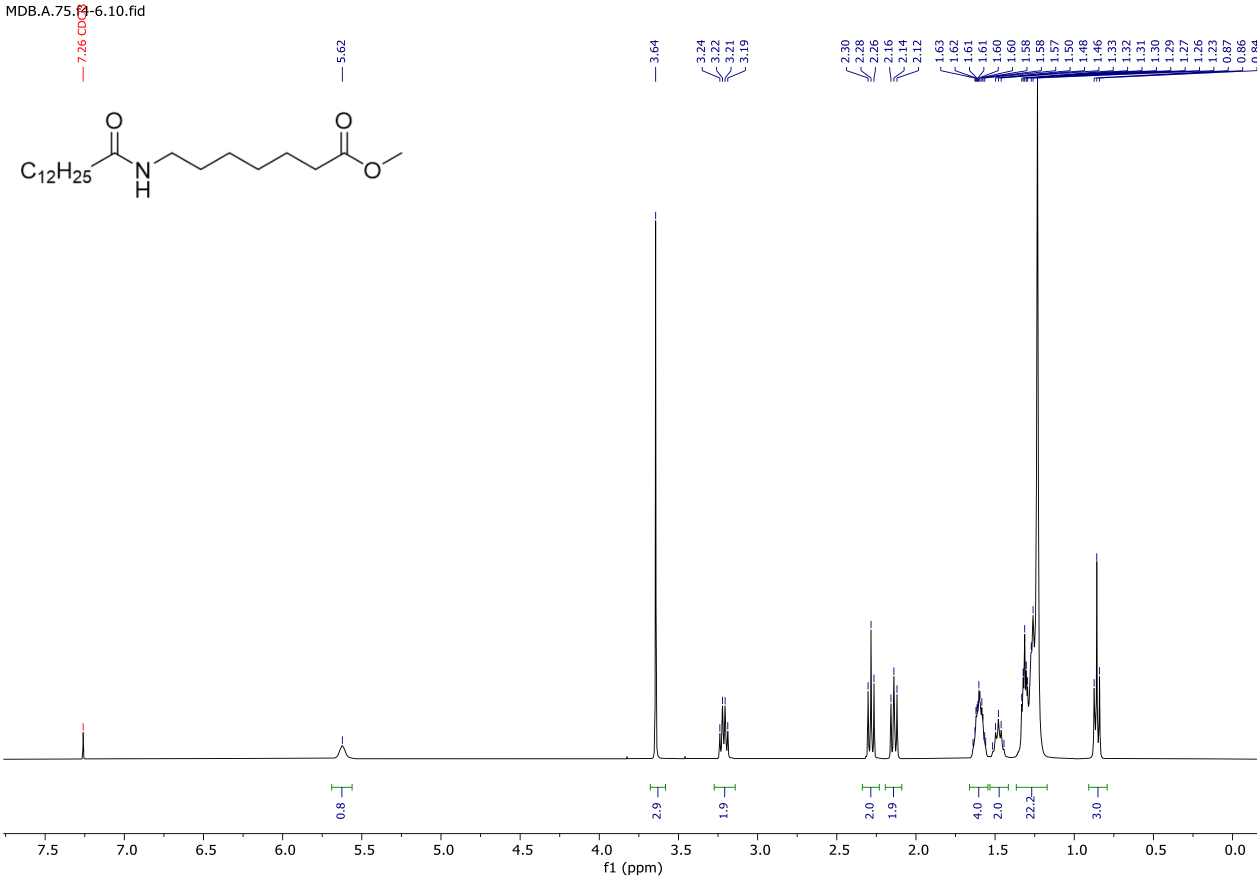

Figure S-77 ^{1}H NMR spectrum of **28d**.

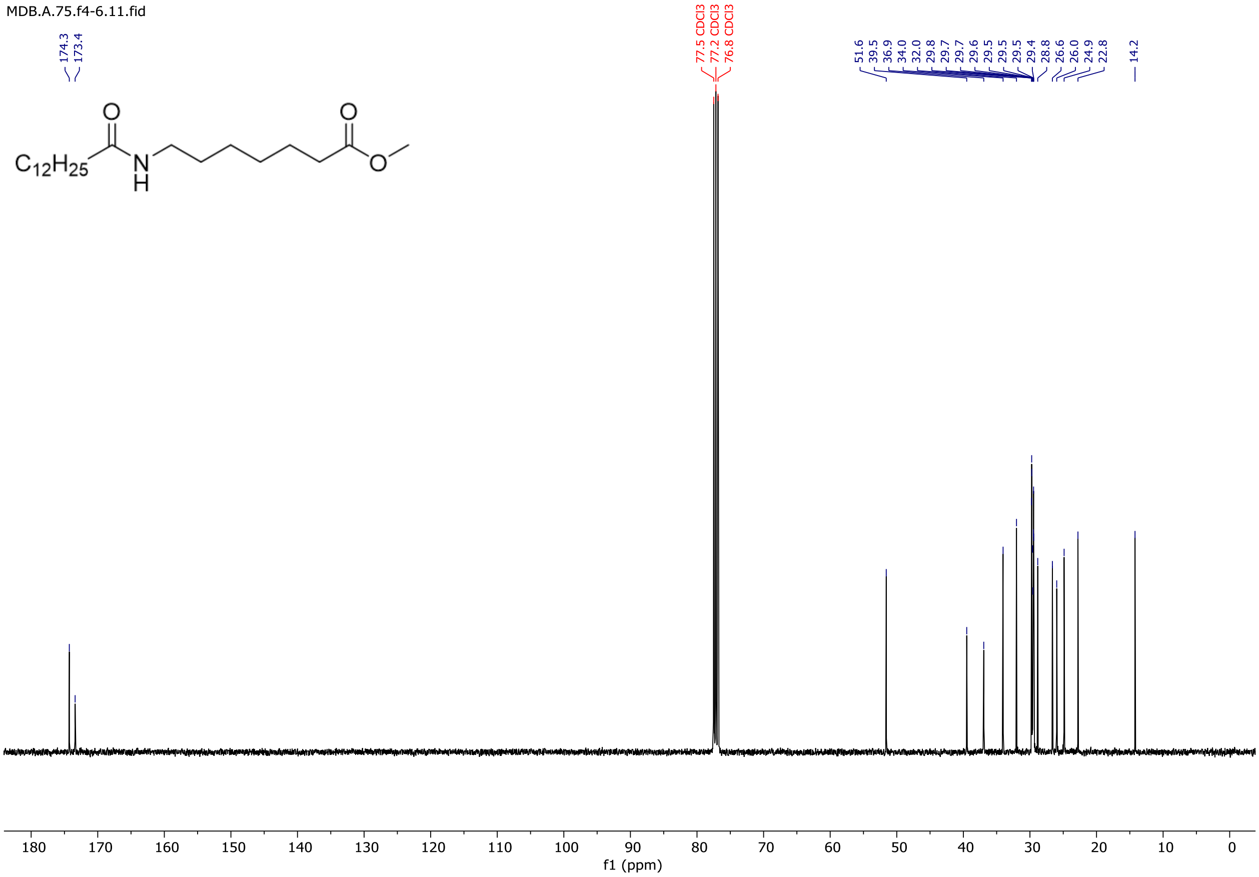

Figure S-78 ^{13}C NMR spectrum of **28d**.

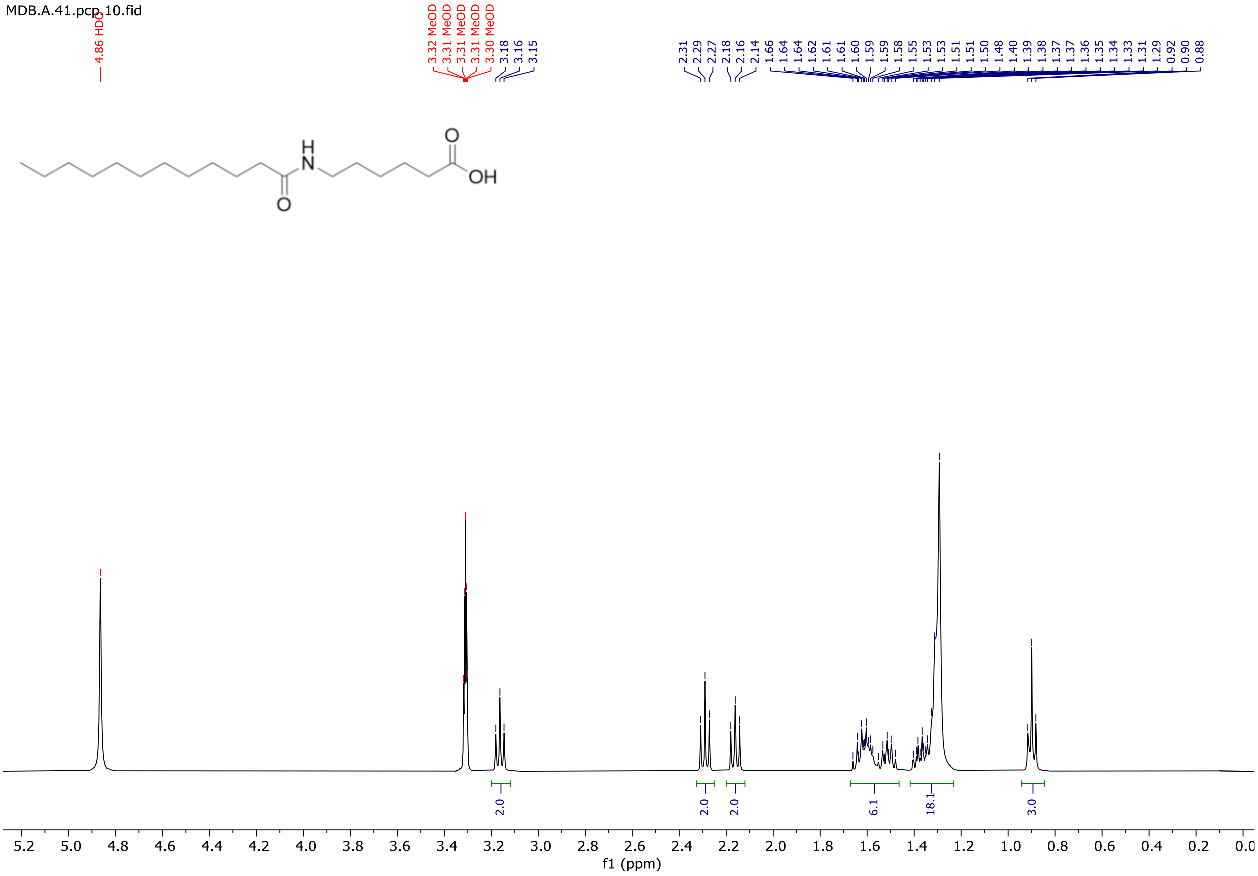

Figure S-79 ^{1}H NMR spectrum of **9a**.

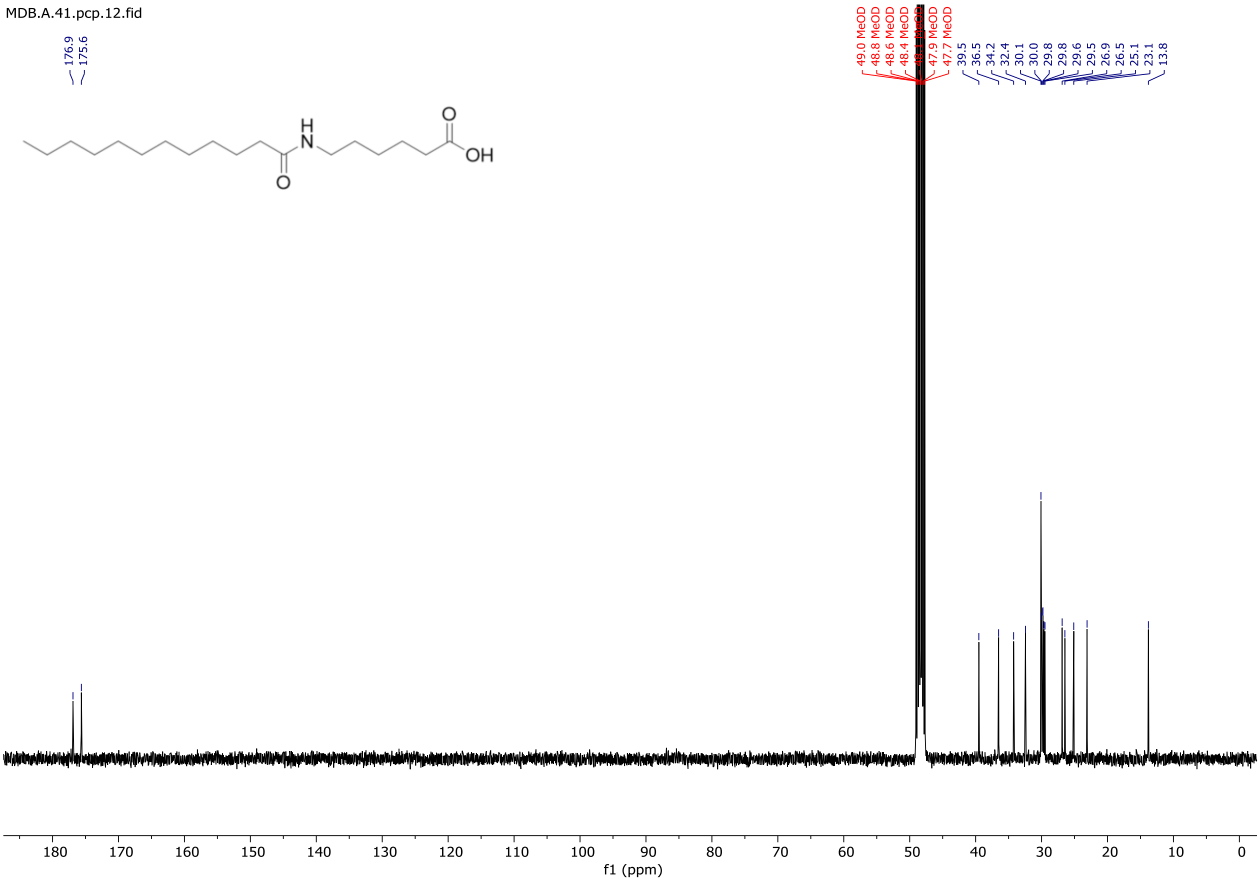

Figure S-80 ^{13}C NMR spectrum of **9a**.

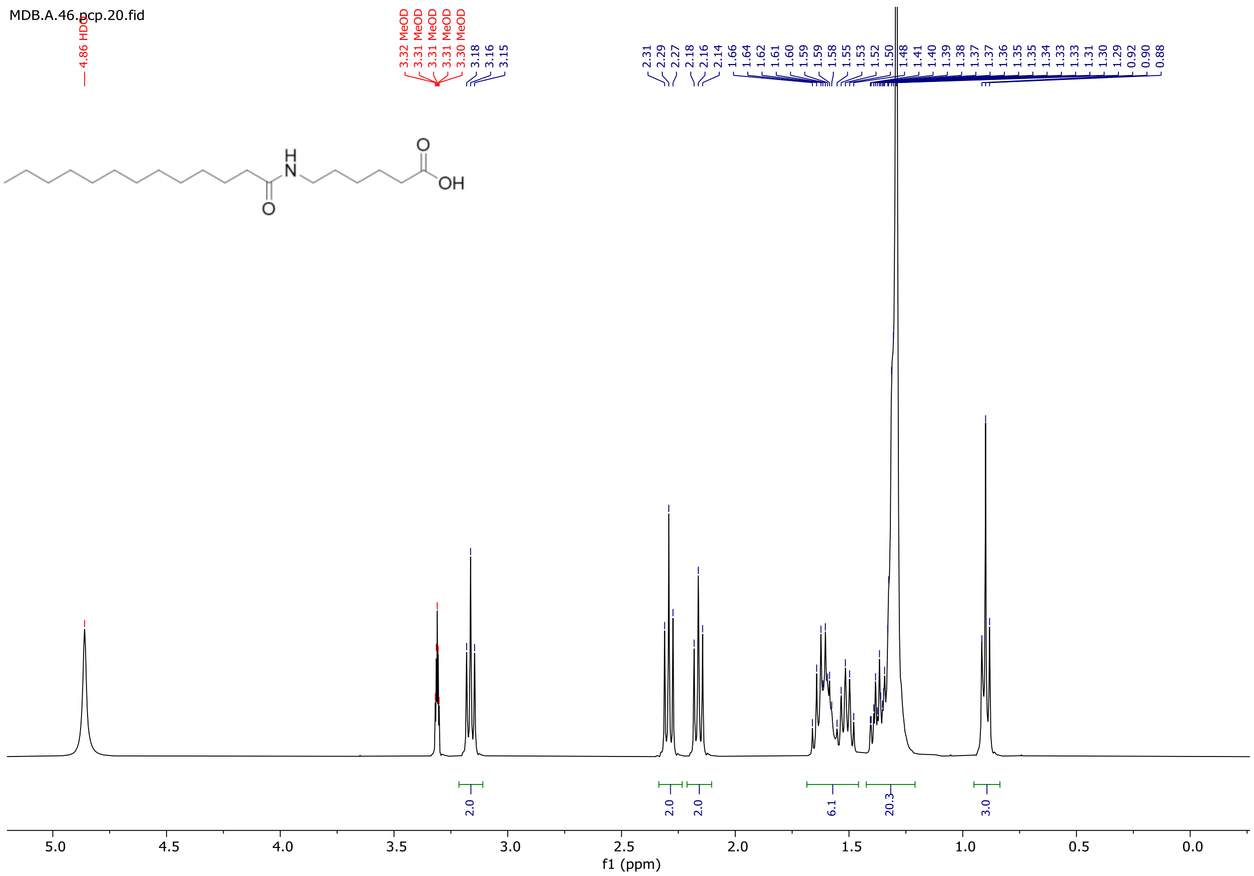

Figure S-81 ^{1}H NMR spectrum of **9b**.

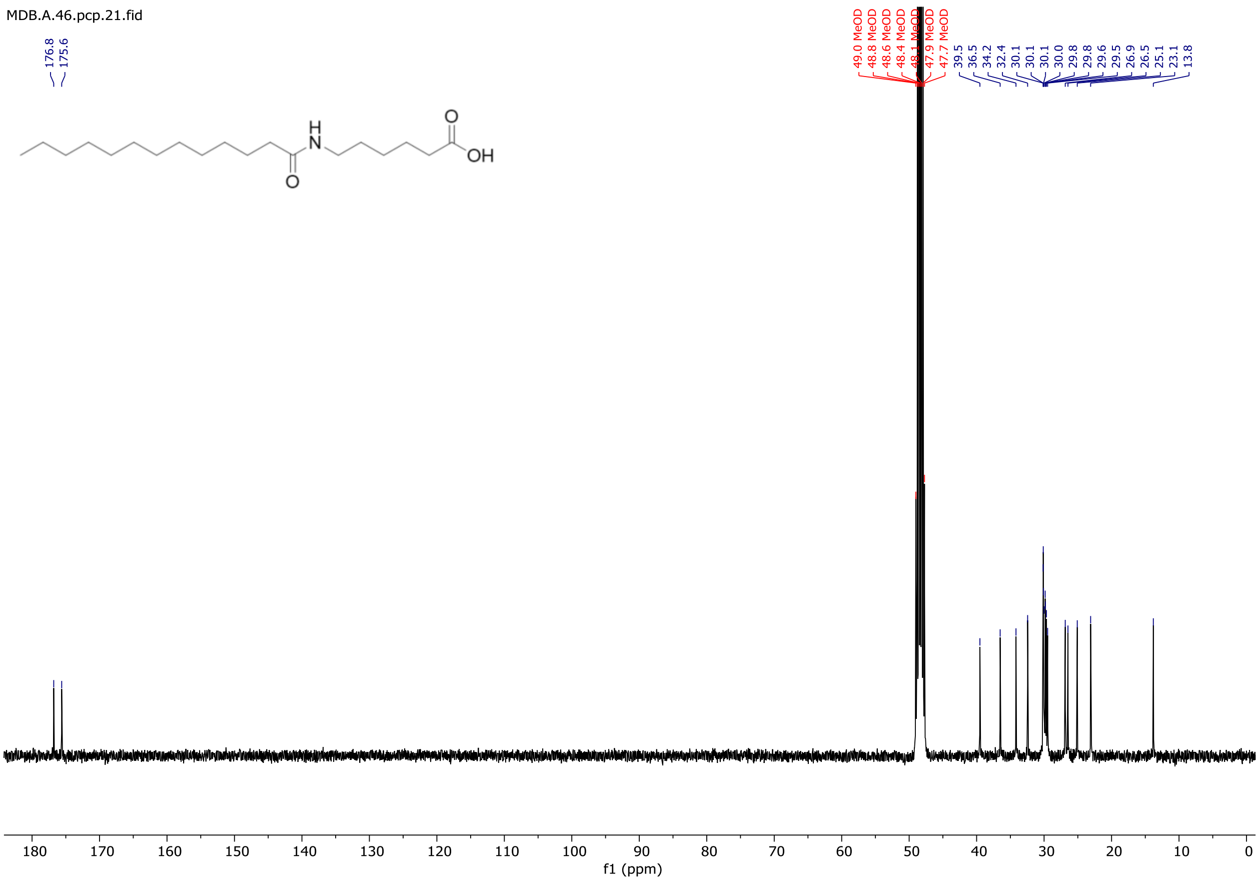

Figure S-82 ^{13}C NMR spectrum of **9b**.

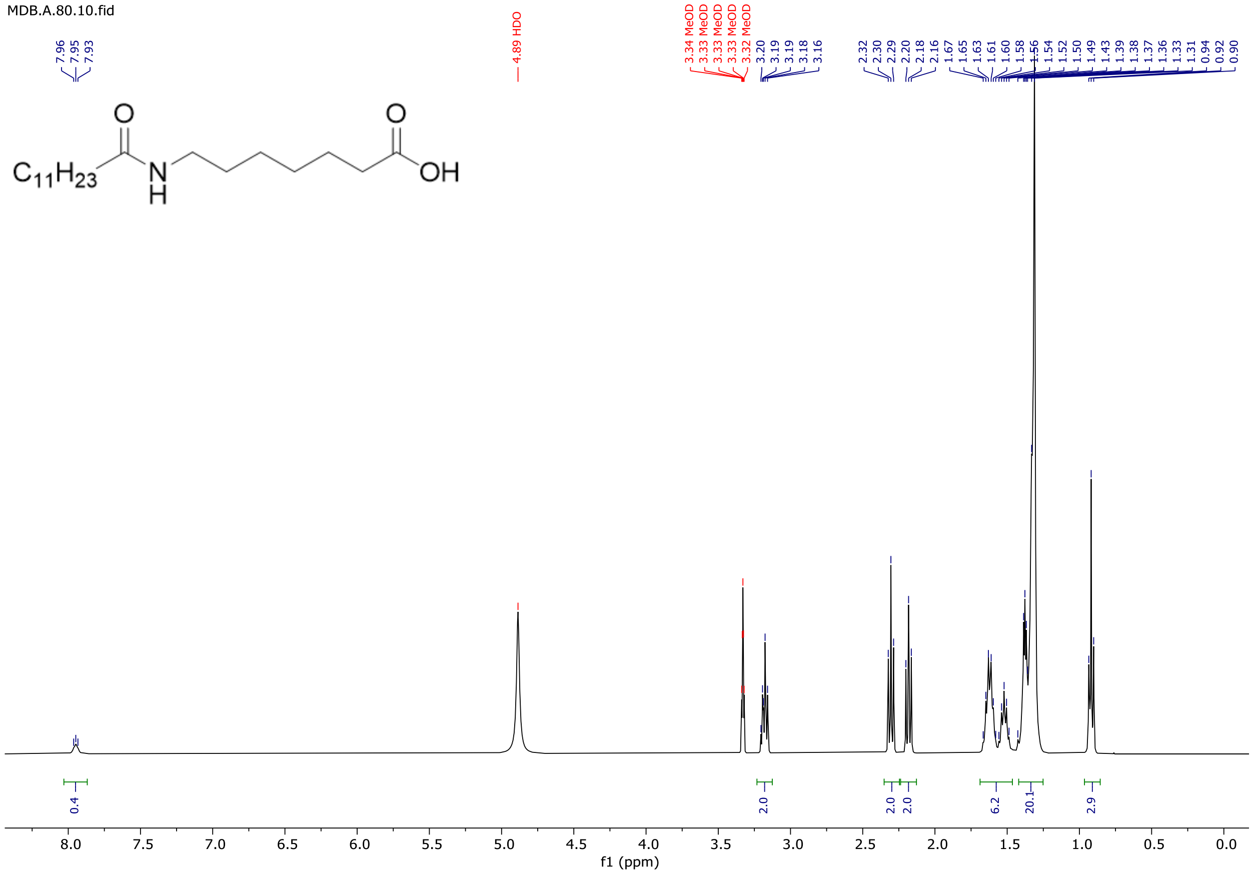

Figure S-83 ^{1}H NMR spectrum of **9c**.

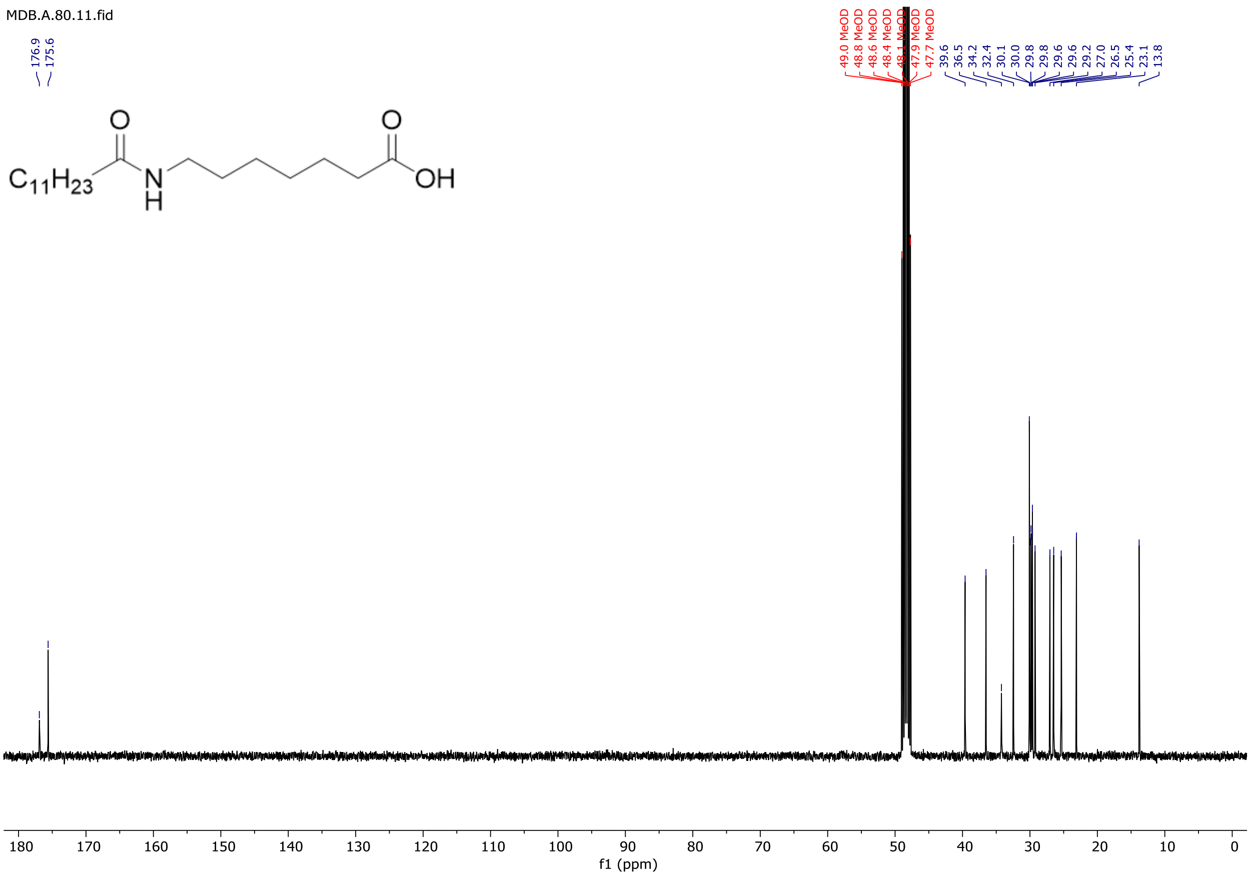

Figure S-84 ^{13}C NMR spectrum of **9c**.

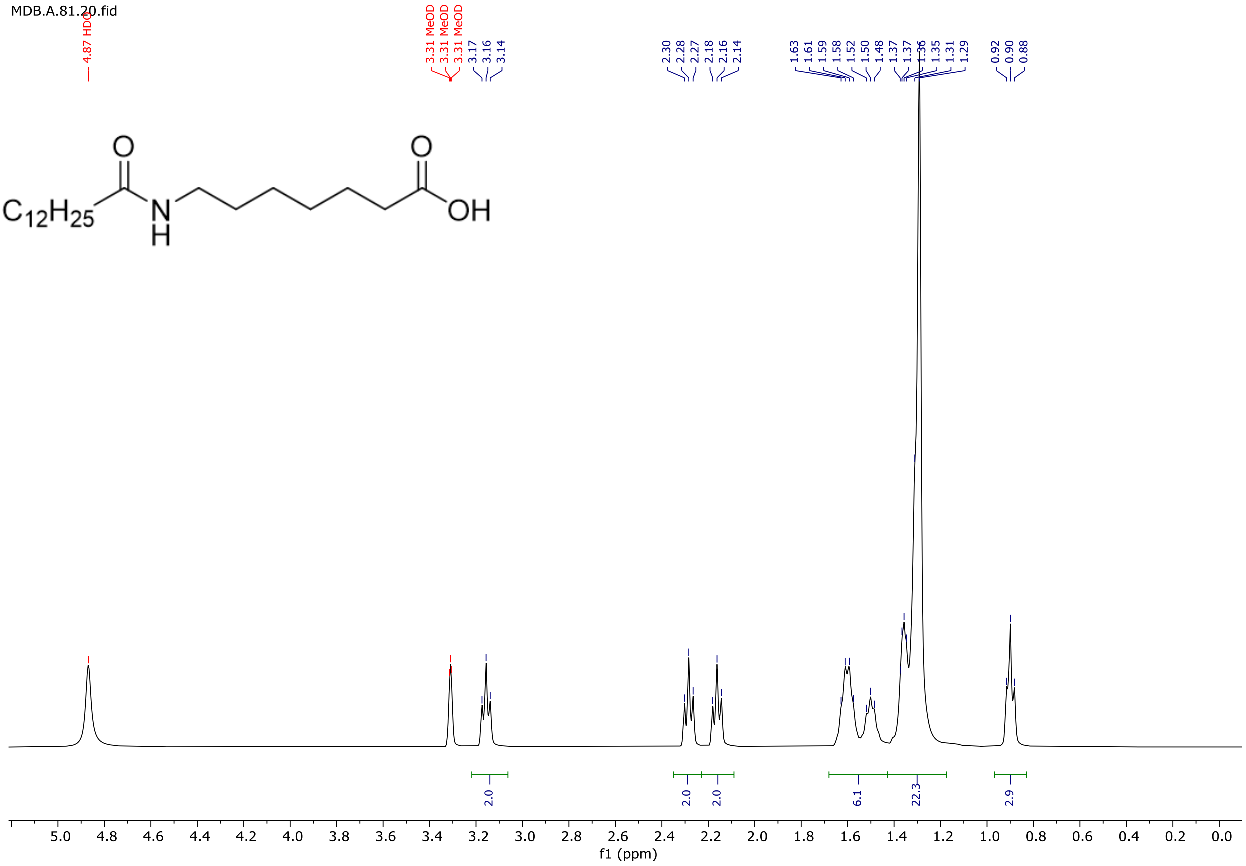

Figure S-85 ^{1}H NMR spectrum of **9d**.

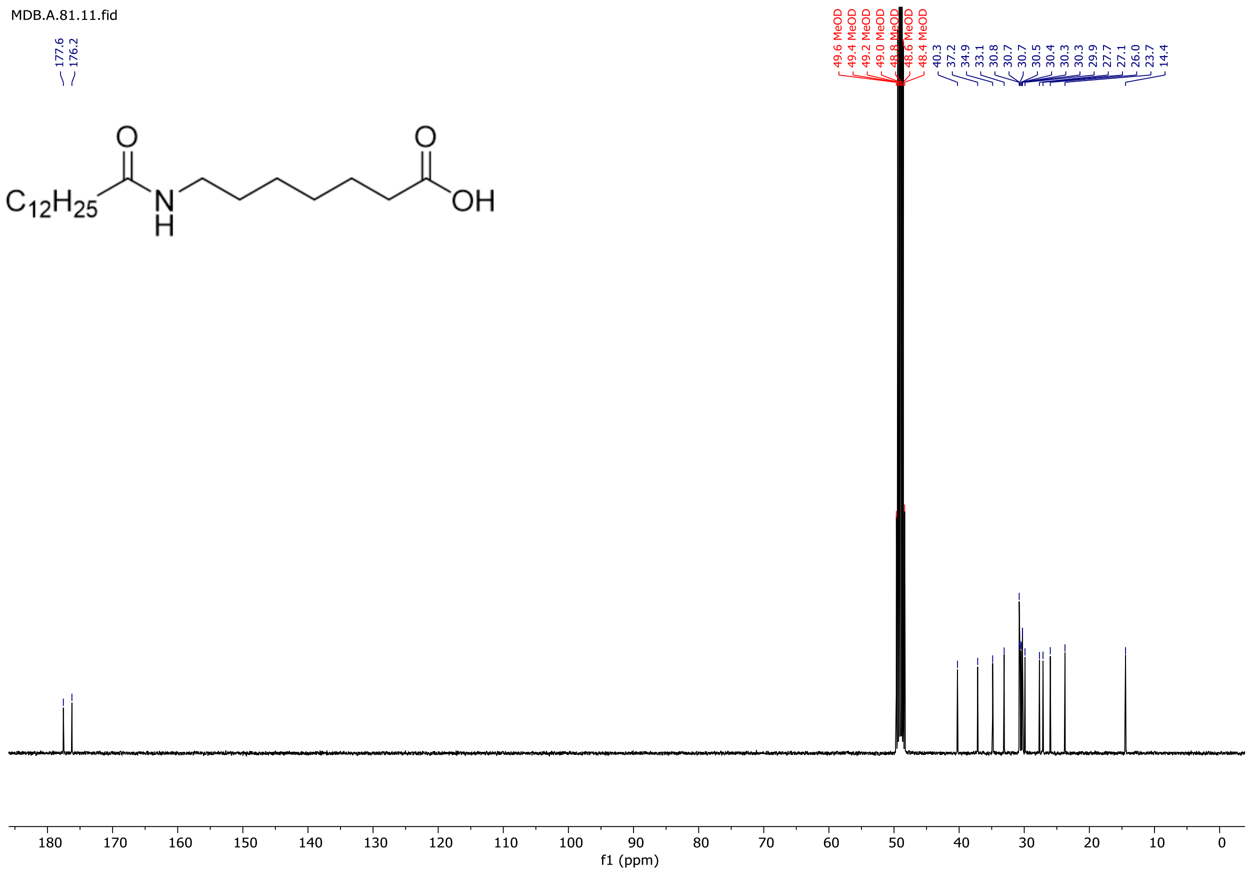

Figure S-86 ^{13}C NMR spectrum of **9d**.

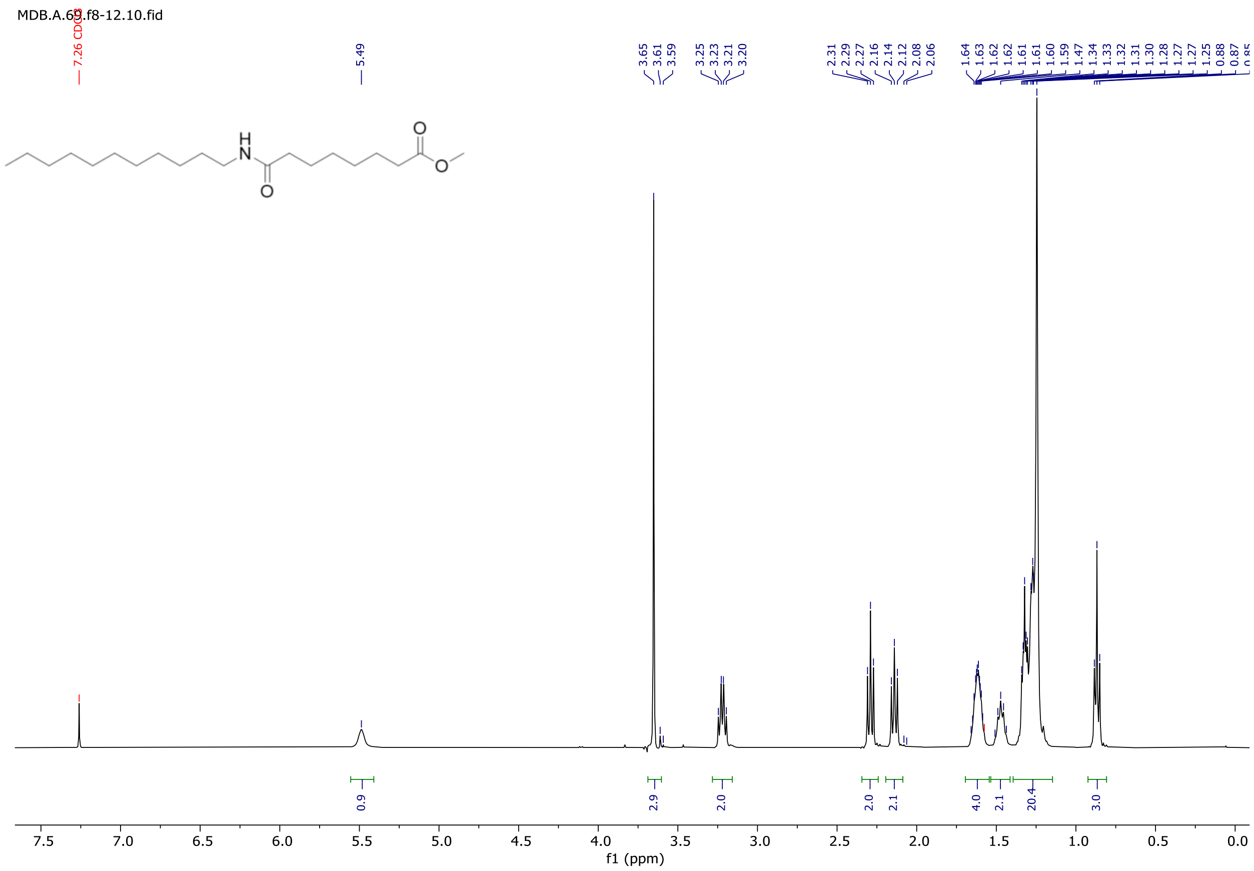

Figure S-87 ^{1}H NMR spectrum of **30a**.

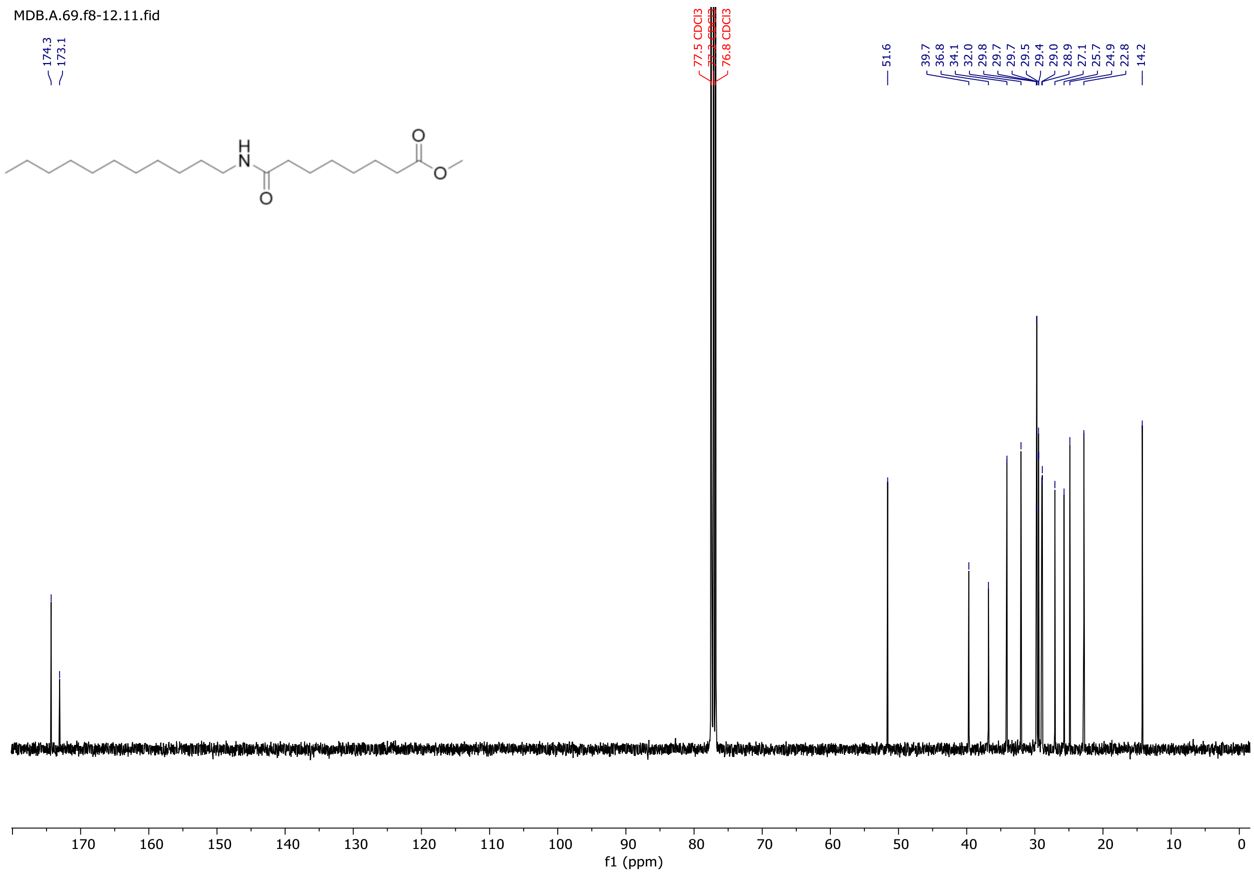

Figure S-88 ^{13}C NMR spectrum of **30a**.

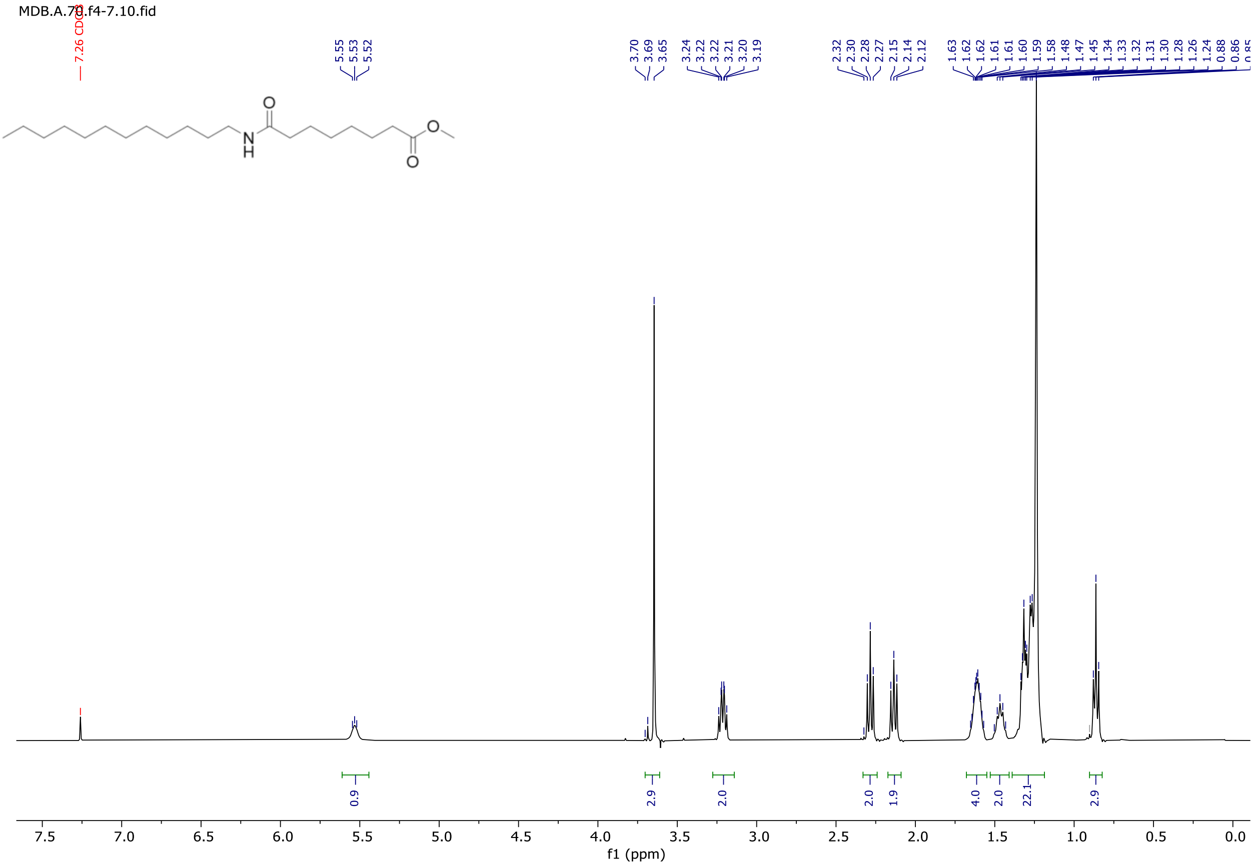

Figure S-89 ^{1}H NMR spectrum of **30b**.

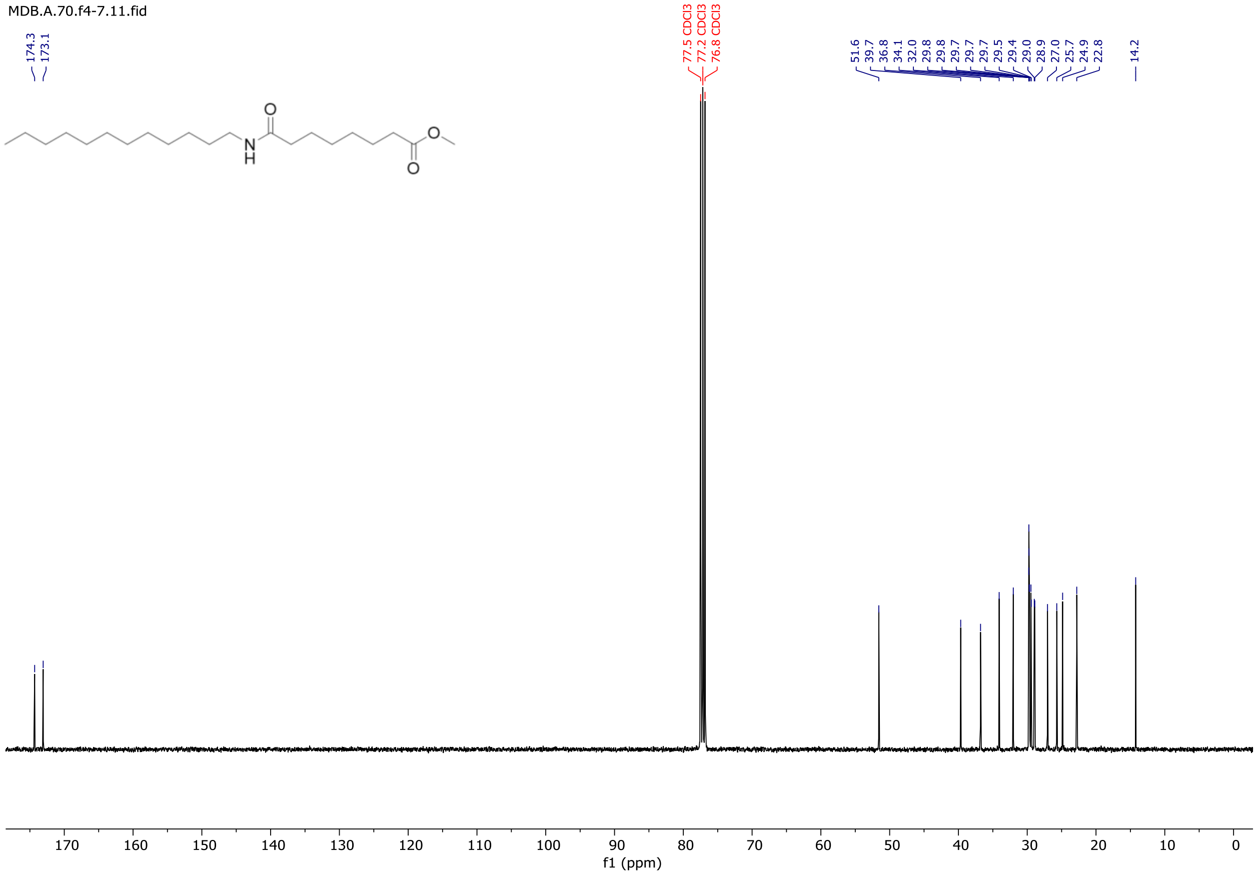

Figure S-90 ^{13}C NMR spectrum of **30b**.

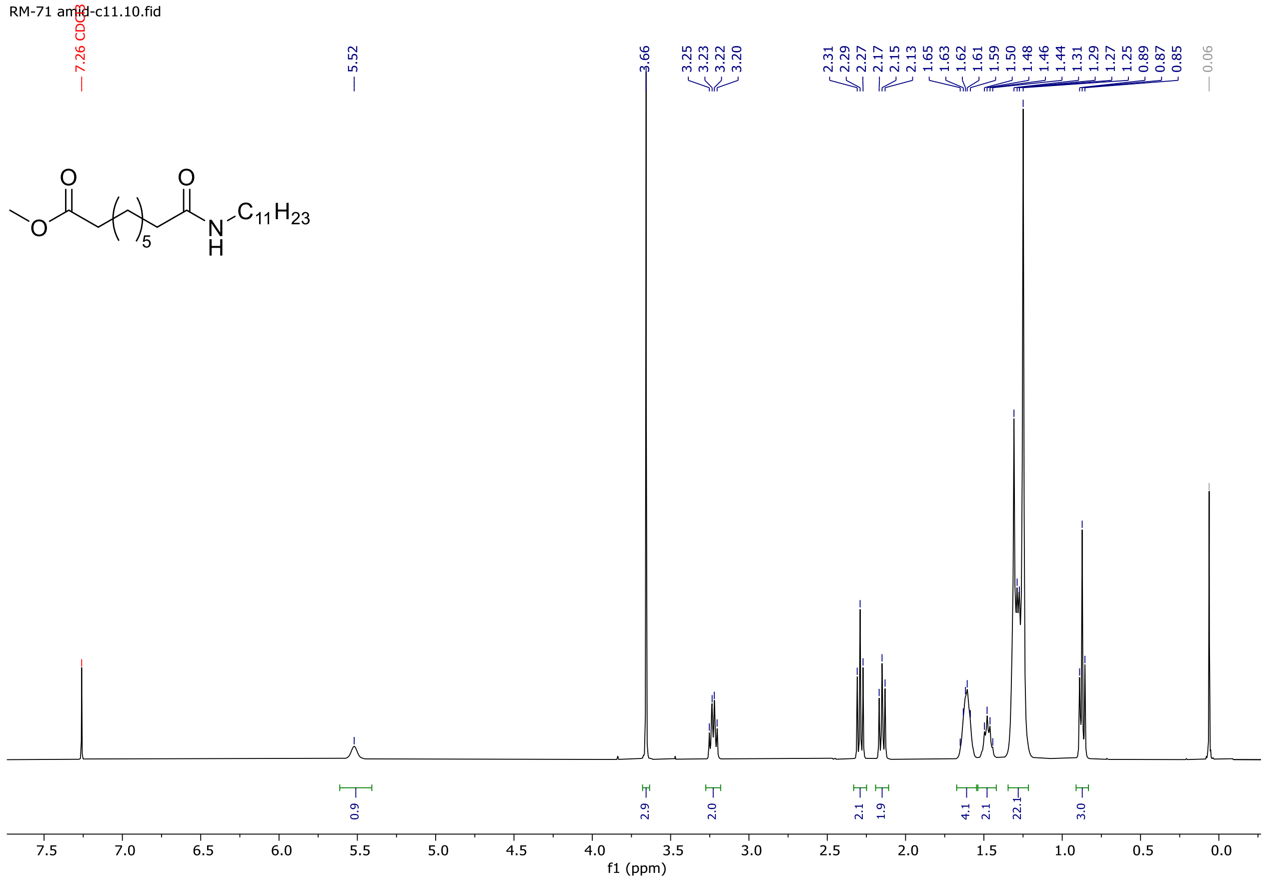

Figure S-91 ^{1}H NMR spectrum of **30c**.

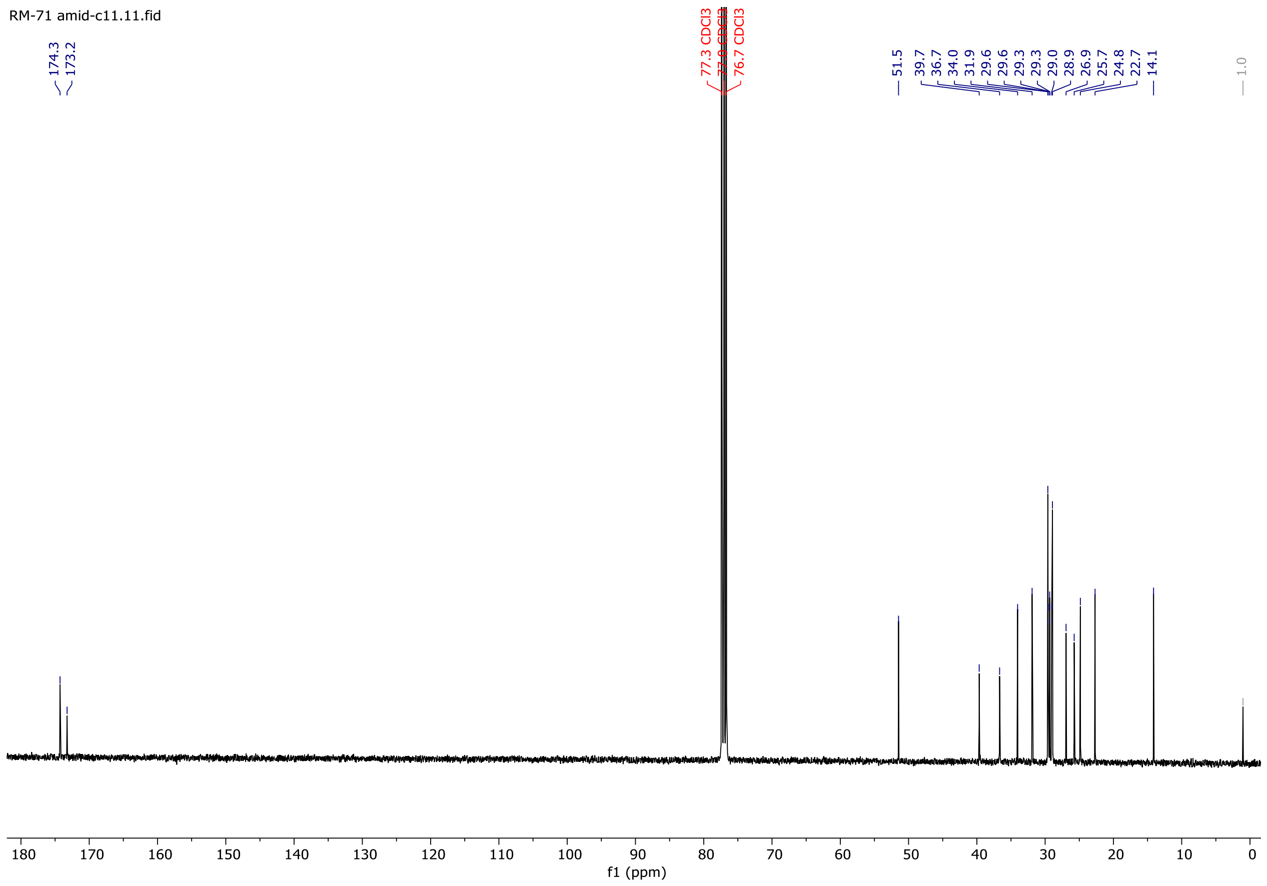

Figure S-92 ^{13}C NMR spectrum of **30c**.

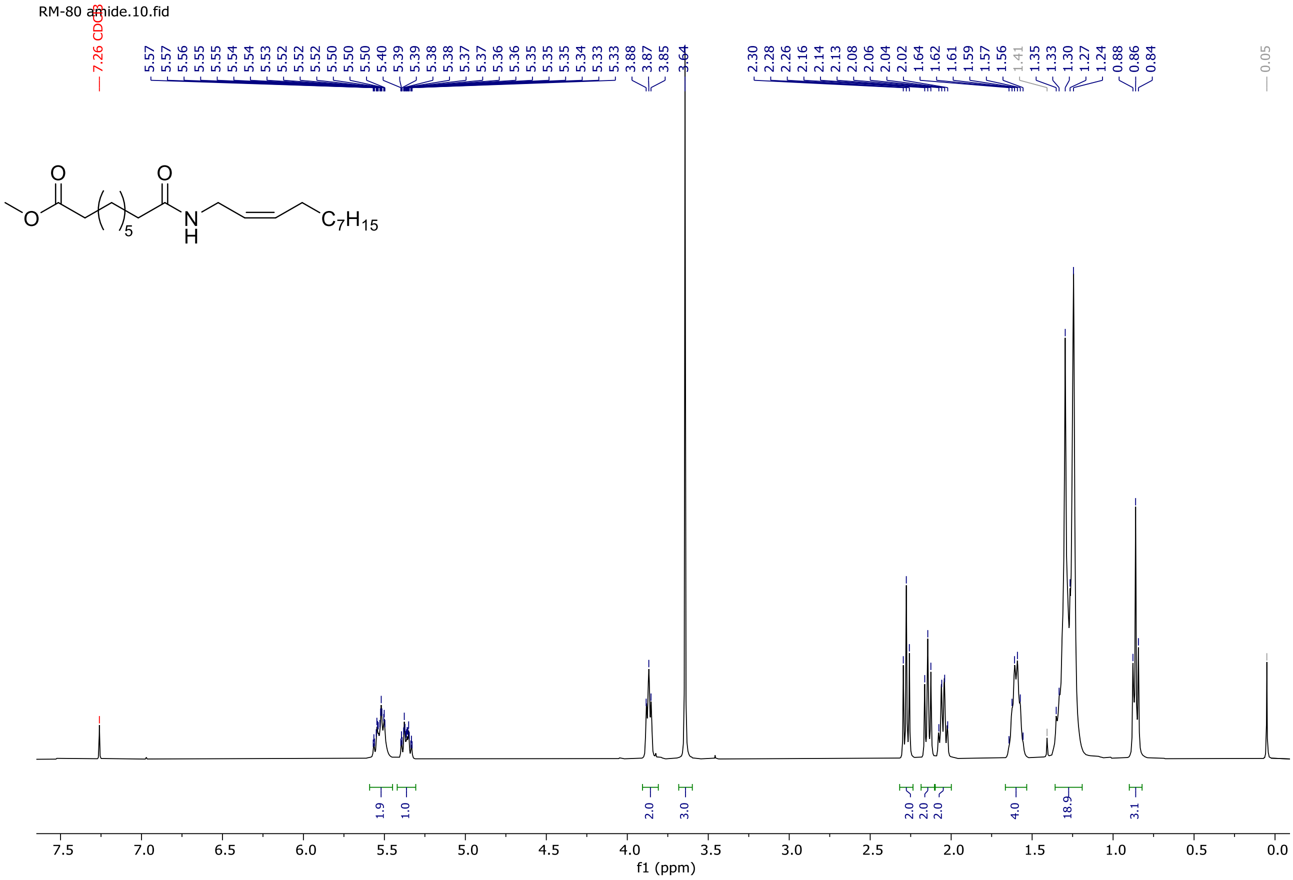

Figure S-93 ^{1}H NMR spectrum of **30d**.

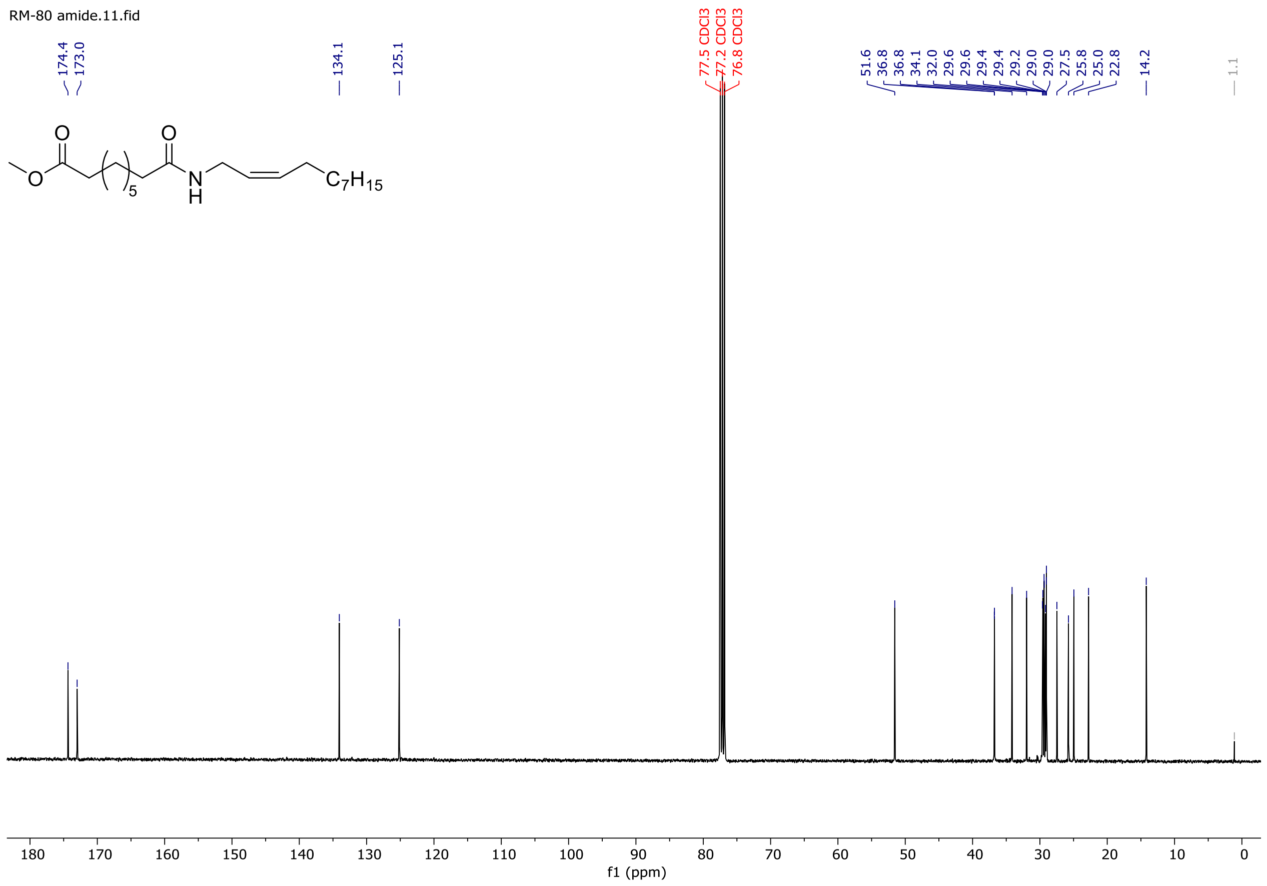

Figure S-94 ^{13}C NMR spectrum of **30d**.

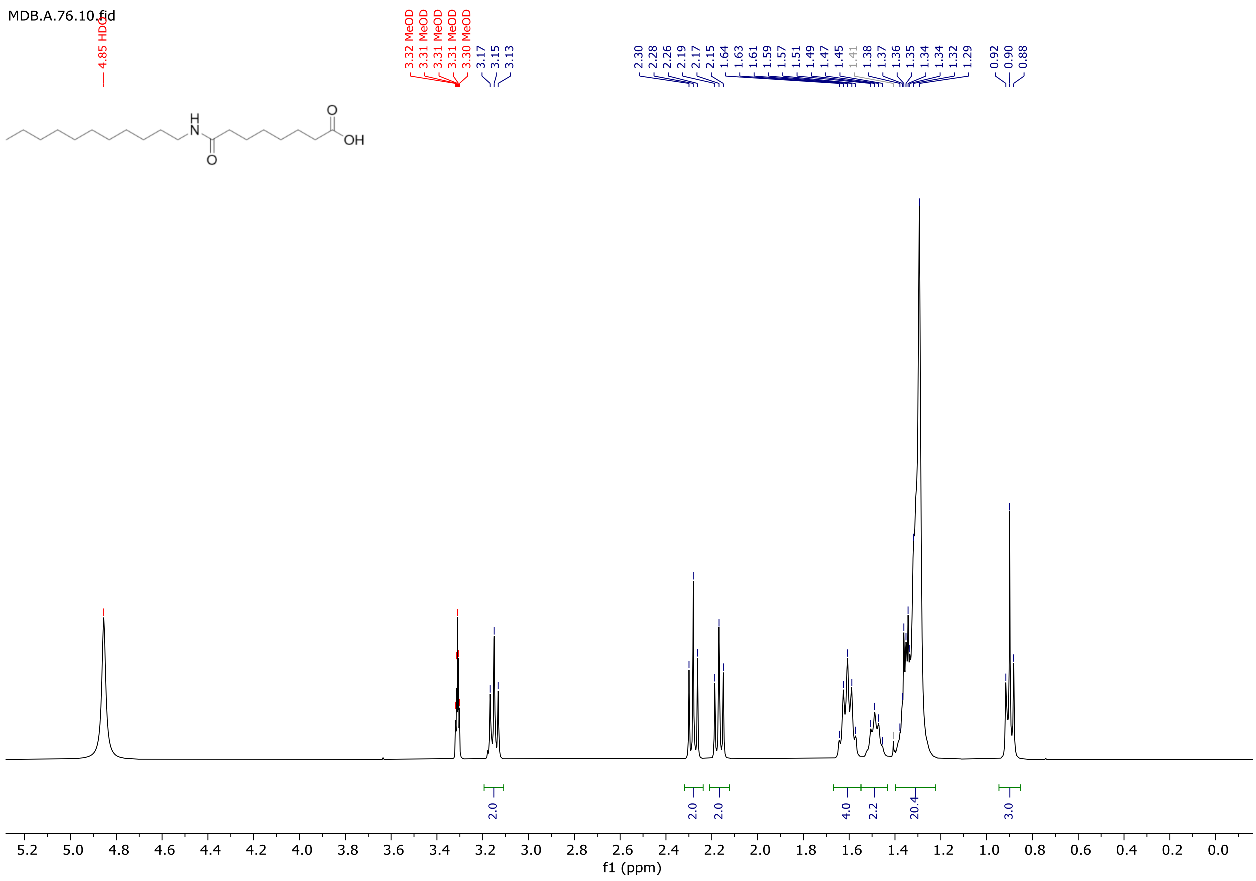

Figure S-95 ^{1}H NMR spectrum of **10a**.

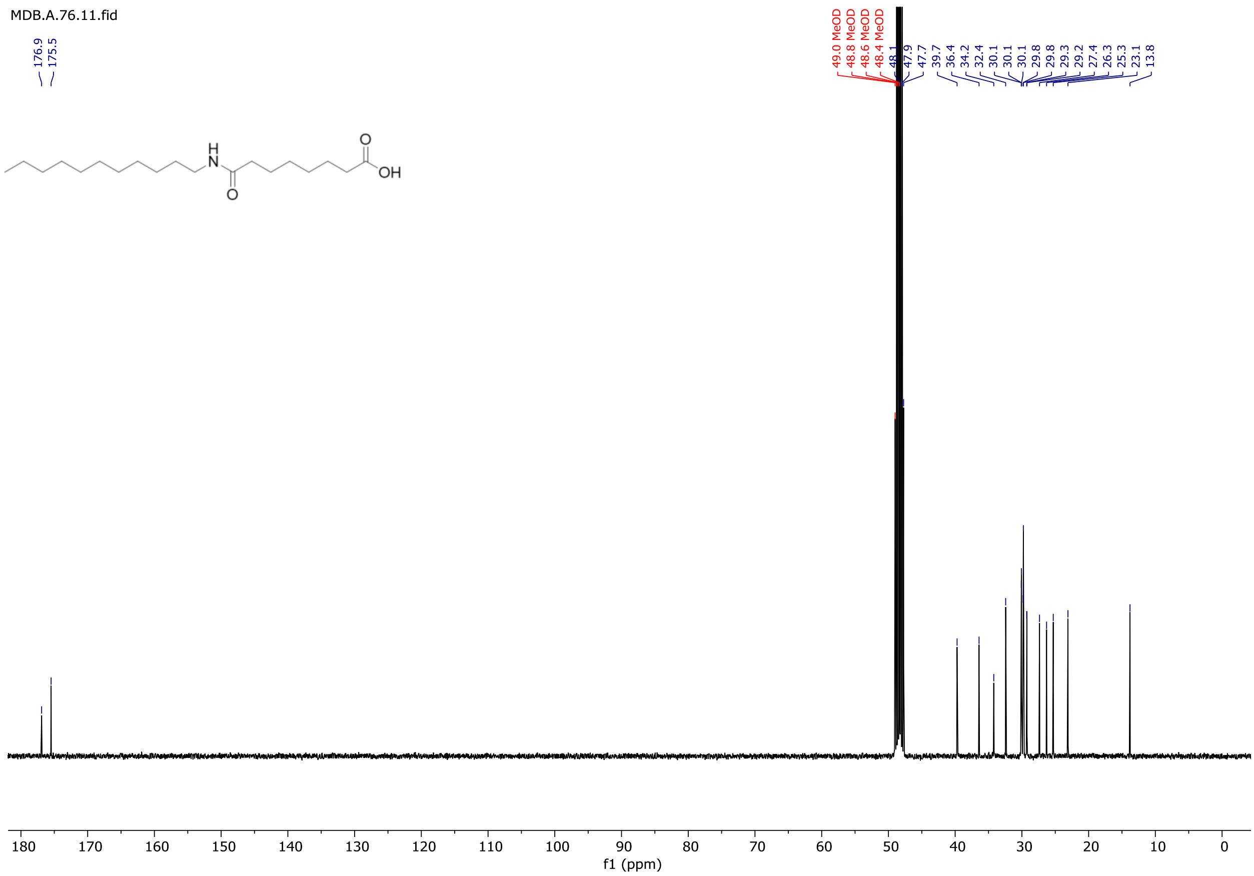

Figure S-96 ^{13}C NMR spectrum of **10a**.

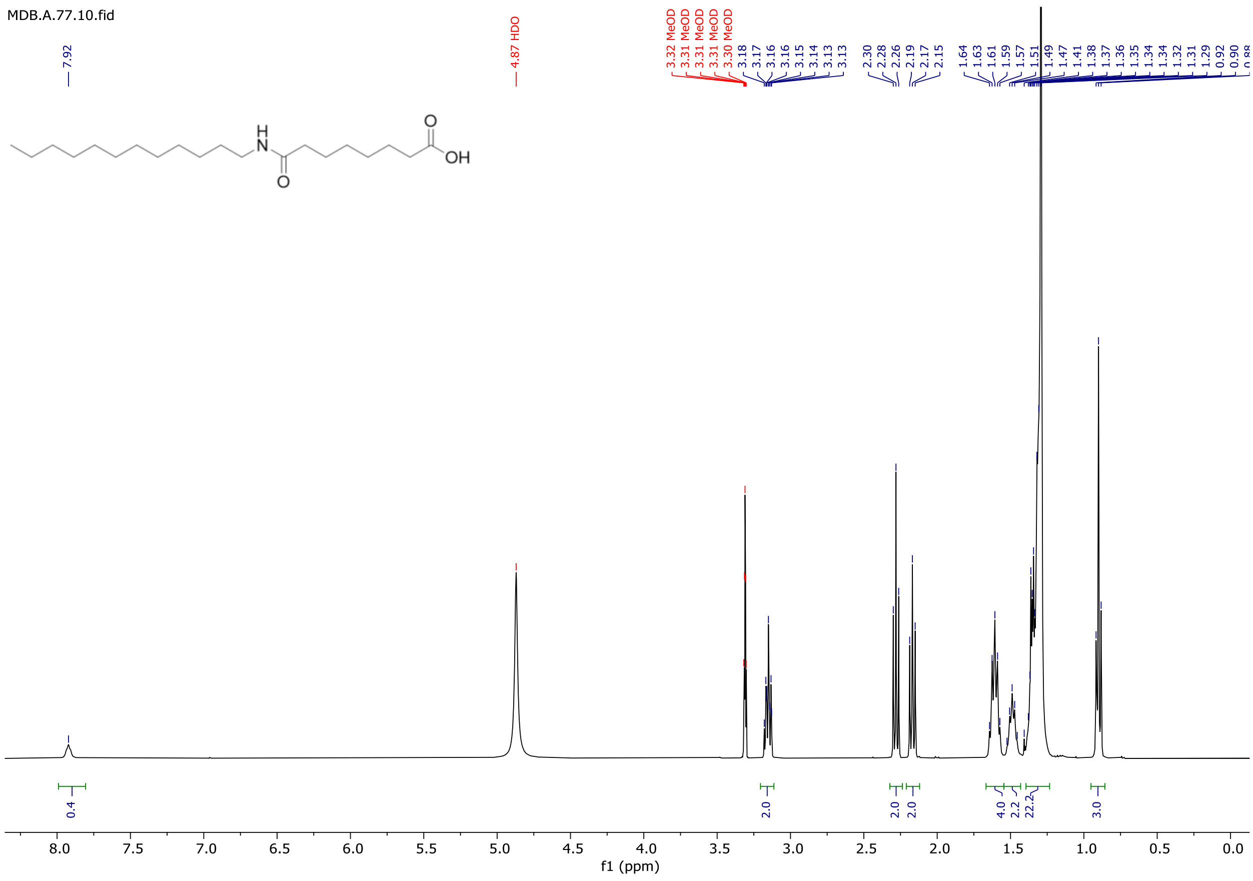

Figure S-97 ^{1}H NMR spectrum of **10b**.

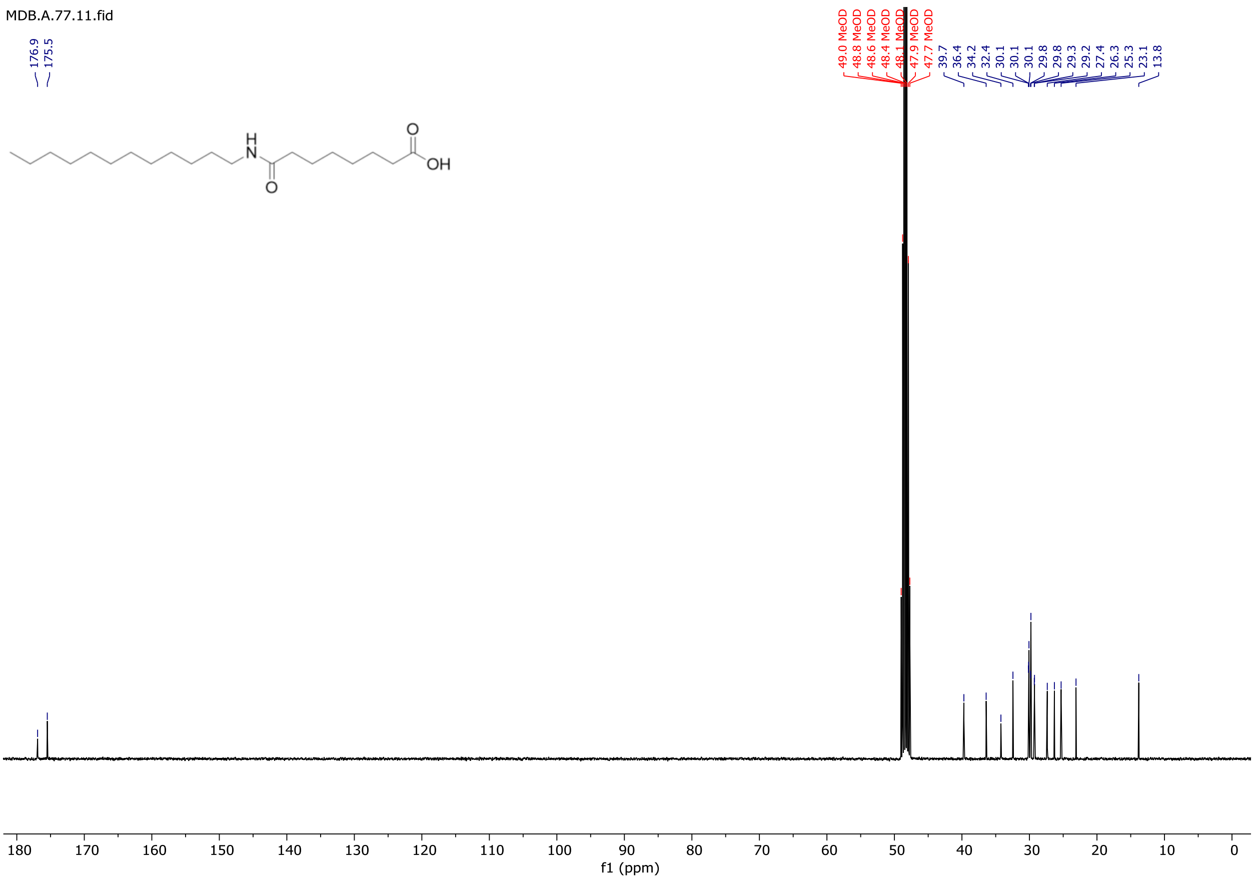

Figure S-98 ^{13}C NMR spectrum of **10b**.

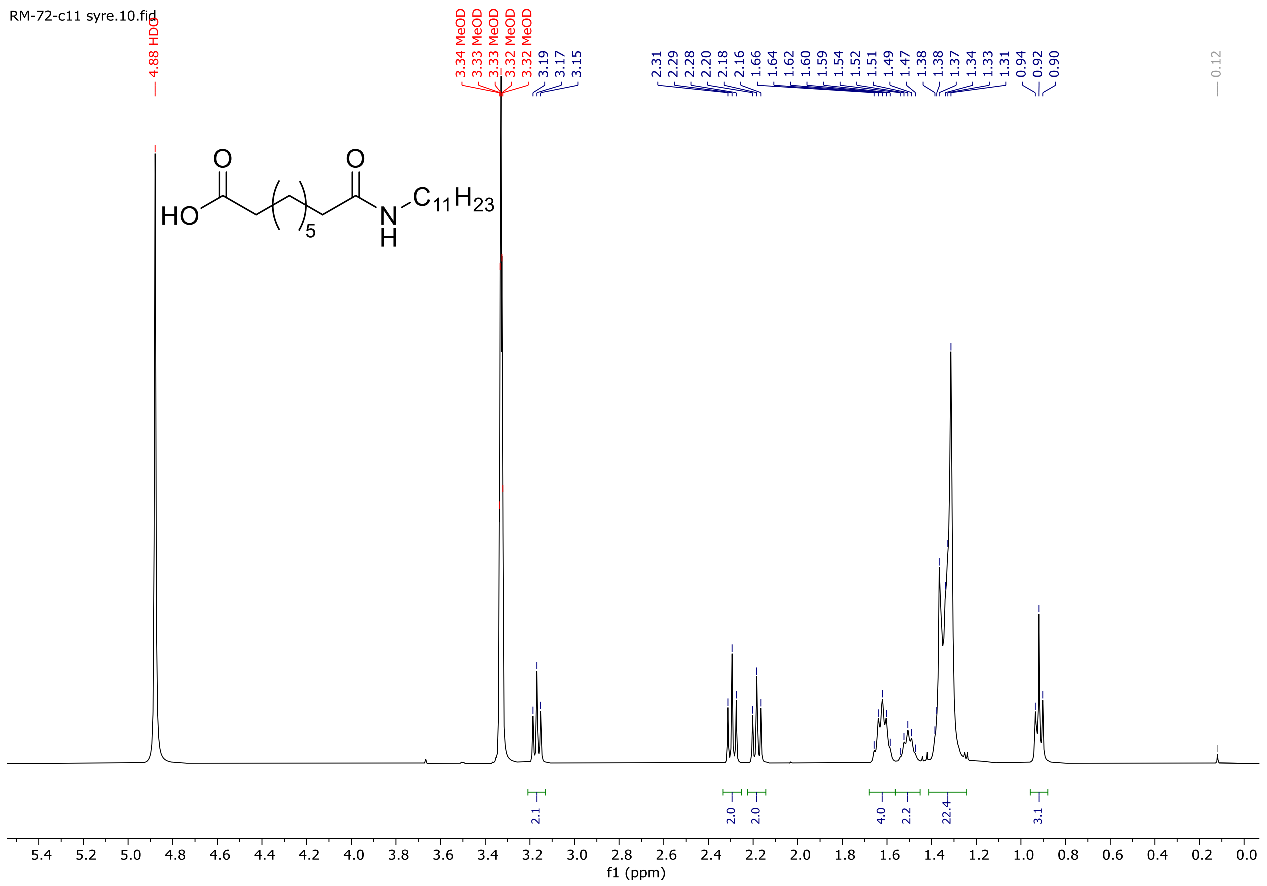

Figure S-99 ^{1}H NMR spectrum of **10c**.

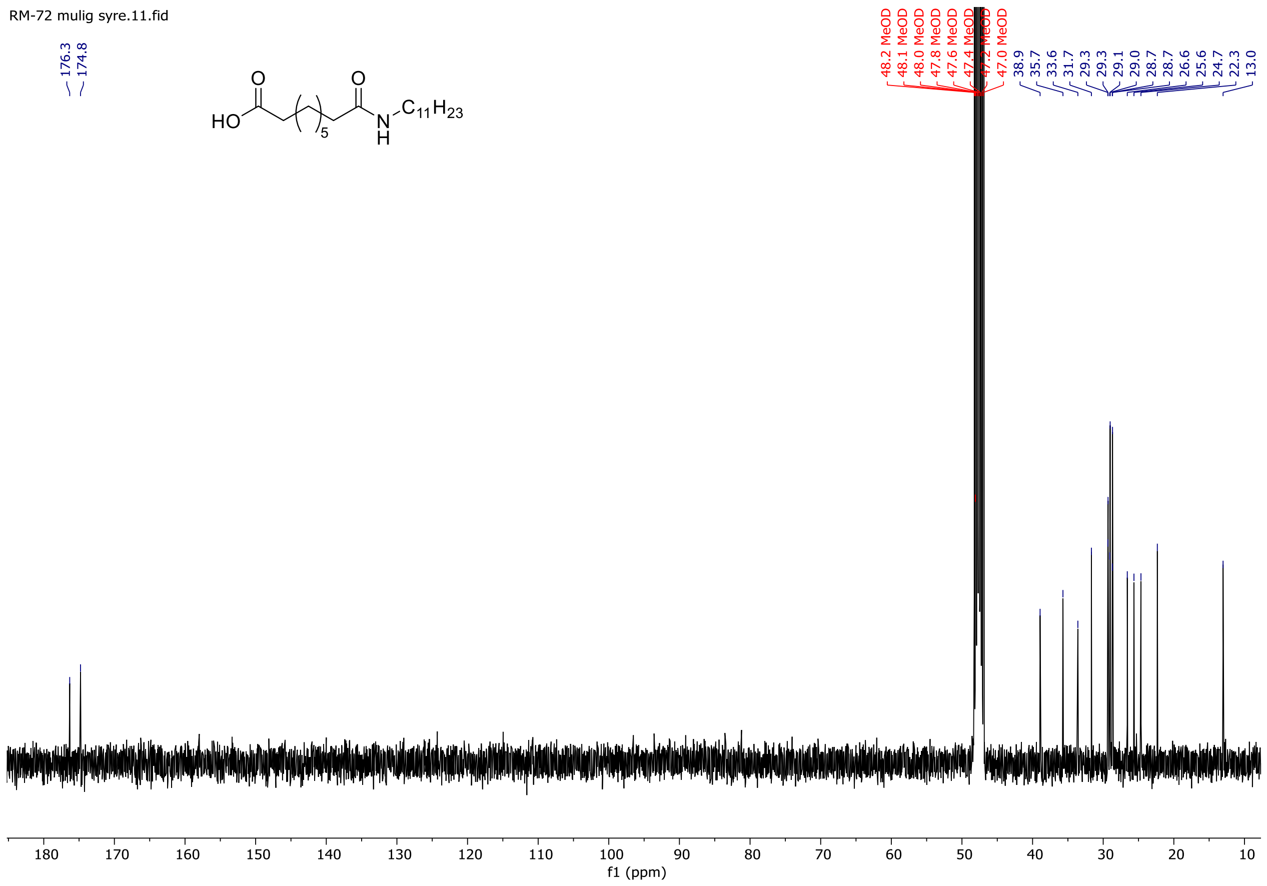

Figure S-100 ^{13}C NMR spectrum of **10c**.

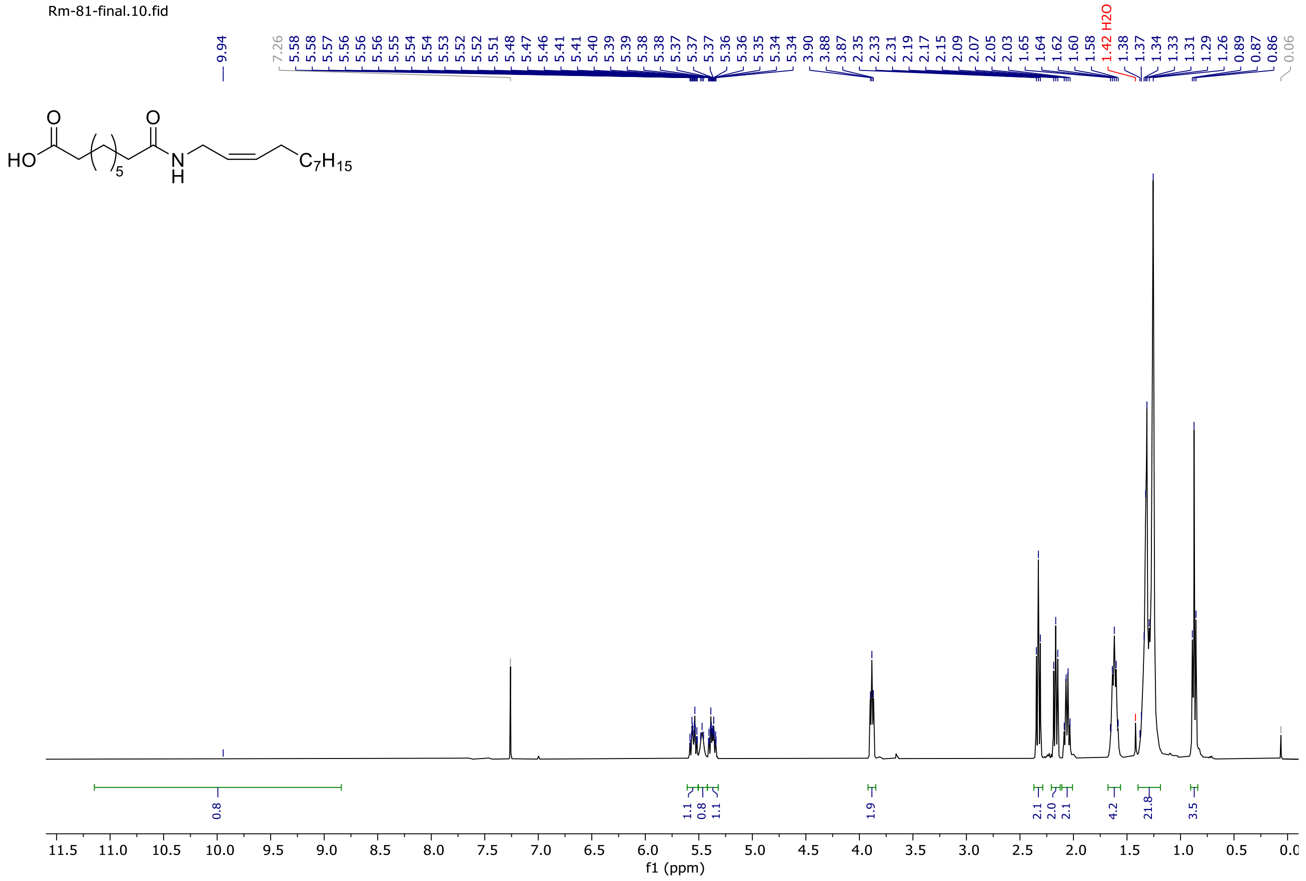

Figure S-101 ^{1}H NMR spectrum of **10d**.

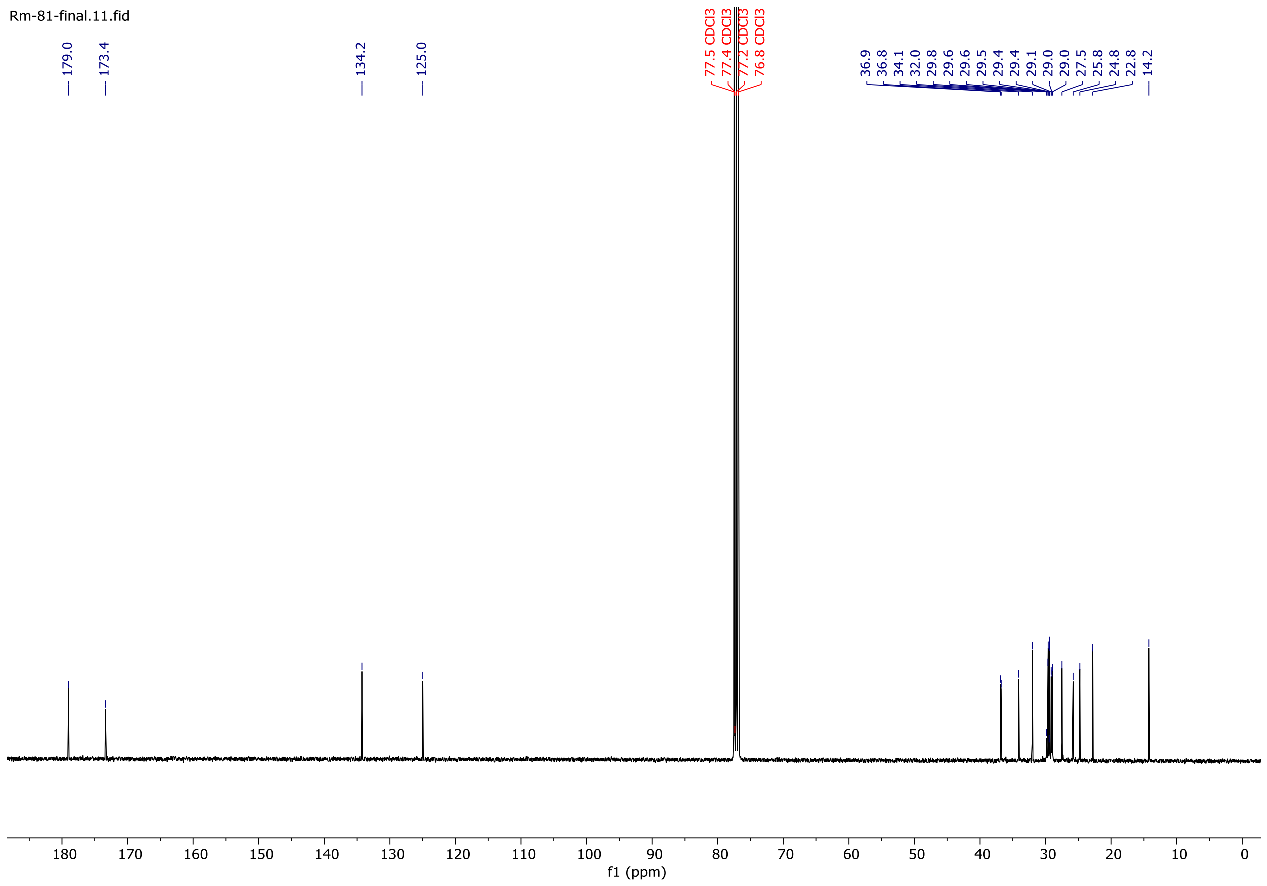

Figure S-102 ^{13}C NMR spectrum of **10d**.

Cell viability assays (WST)

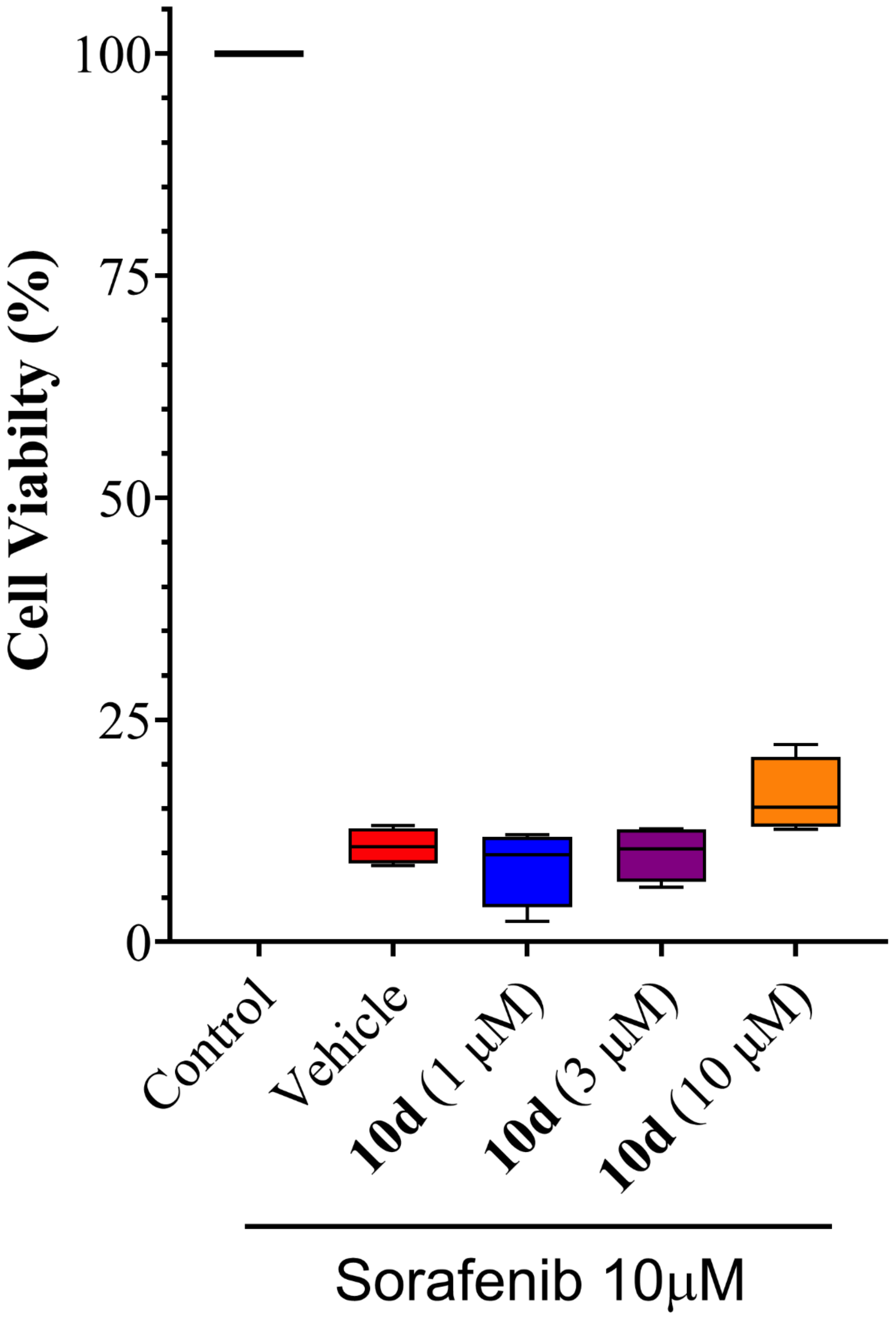

Figure S-103 WST-8 assay of human renal mesangial cells treated with sorafenib and 8,9-EET mimics at indicated concentrations after 48 h incubation, as percentages relative to control. Bars represent mean ± SEM (n = 4).

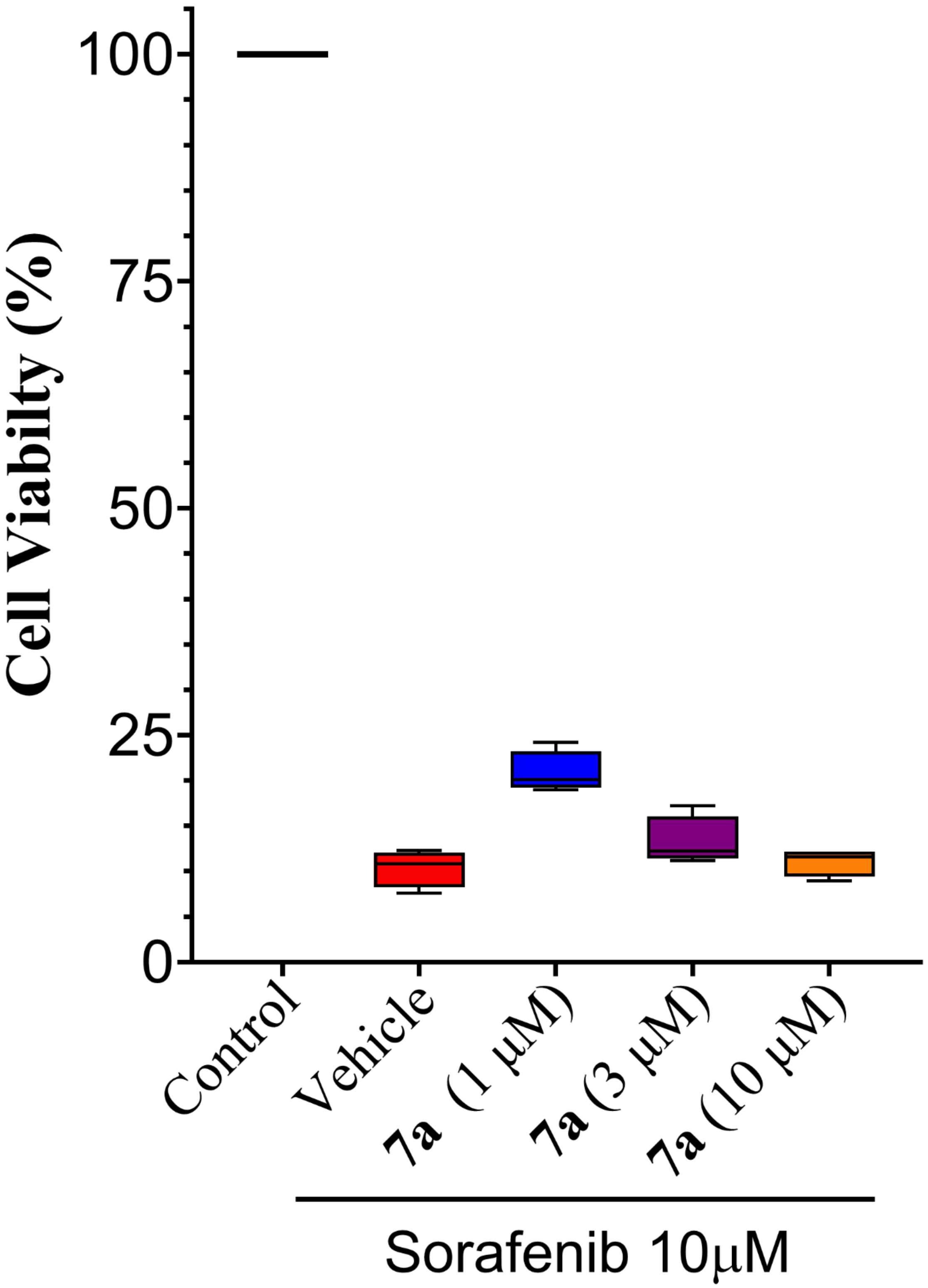

Figure S-104 WST-8 assay of human renal mesangial cells treated with sorafenib and 8,9-EET mimics at indicated concentrations after 48 h incubation, as percentages relative to control. Bars represent mean ± SEM (n = 4).

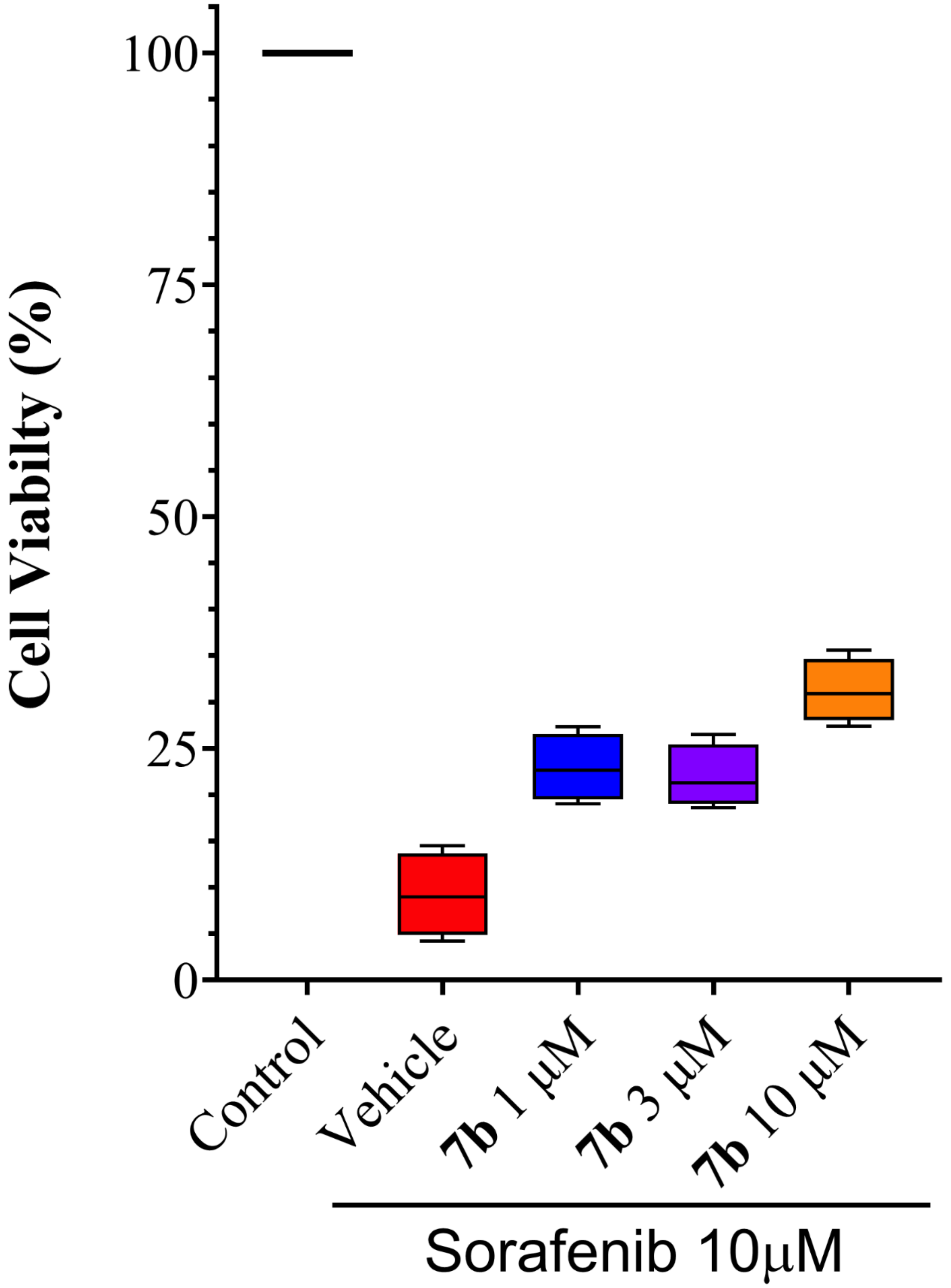

Figure S-105 WST-8 assay of human renal mesangial cells treated with sorafenib and 8,9-EET mimics at indicated concentrations after 48 h incubation, as percentages relative to control. Bars represent mean ± SEM (n = 4).

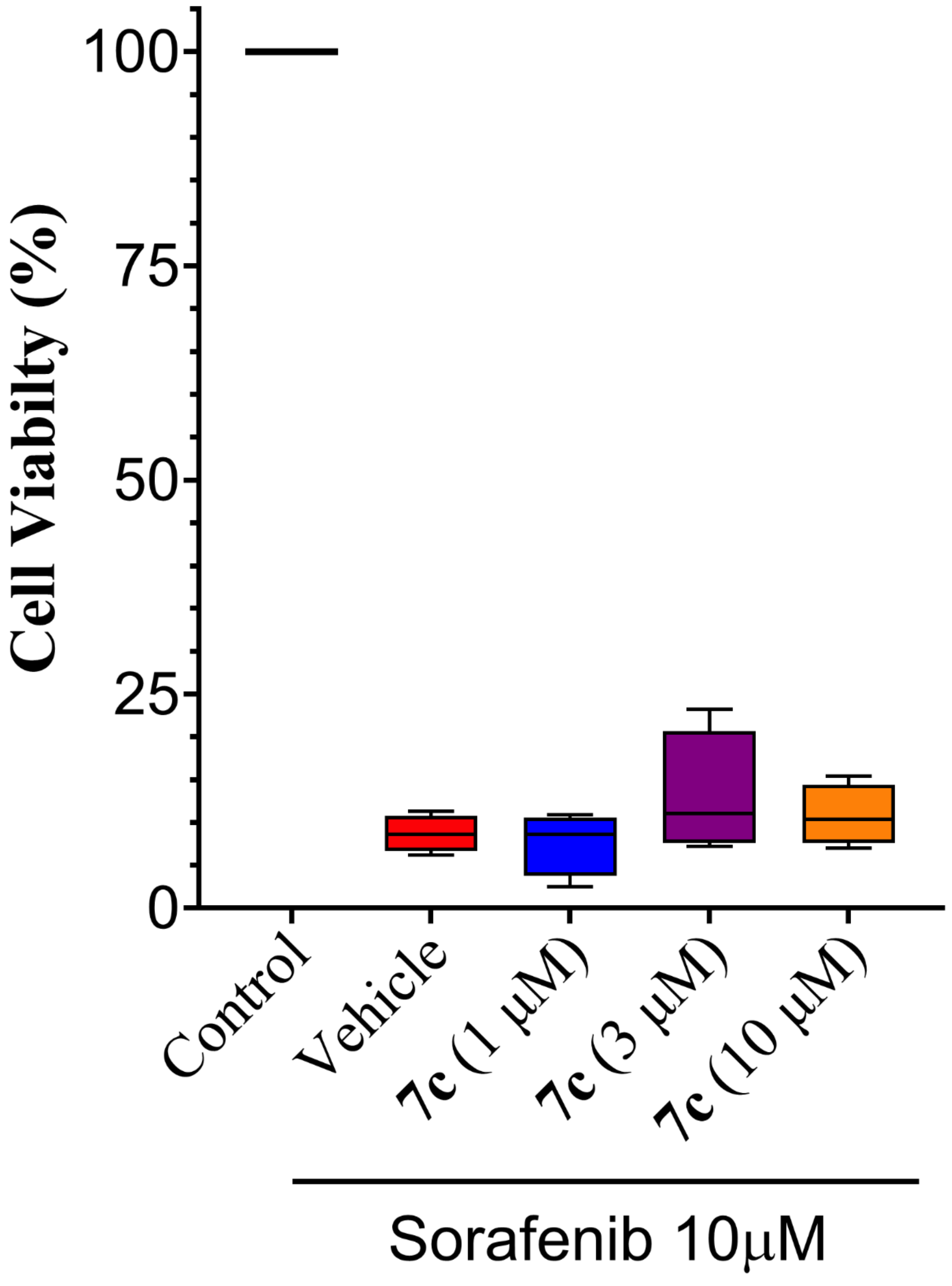

Figure S-106 WST-8 assay of human renal mesangial cells treated with sorafenib and 8,9-EET mimics at indicated concentrations after 48 h incubation, as percentages relative to control. Bars represent mean ± SEM (n = 4).

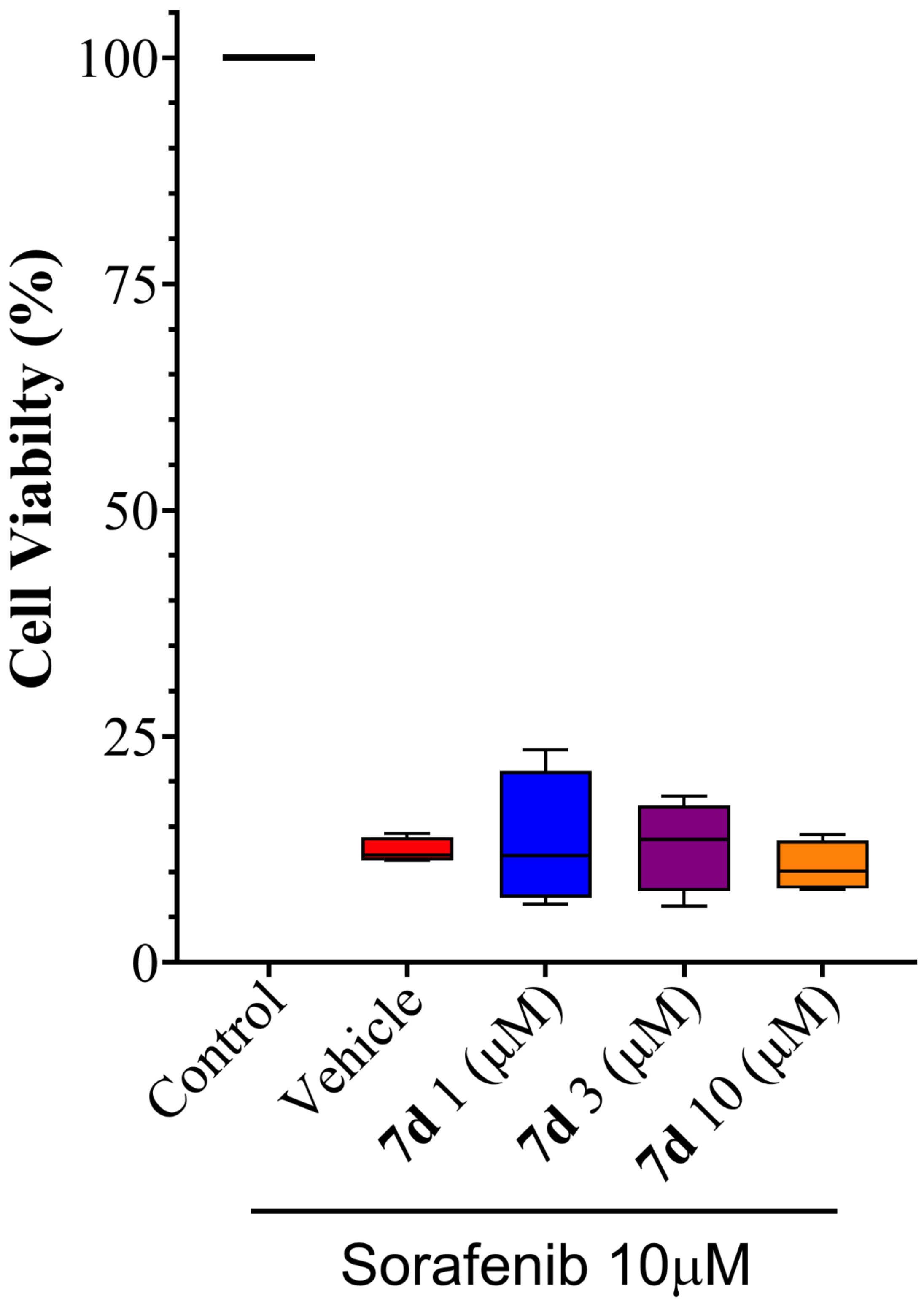

Figure S-107 WST-8 assay of human renal mesangial cells treated with sorafenib and 8,9-EET mimics at indicated concentrations after 48 h incubation, as percentages relative to control. Bars represent mean ± SEM (n = 4).

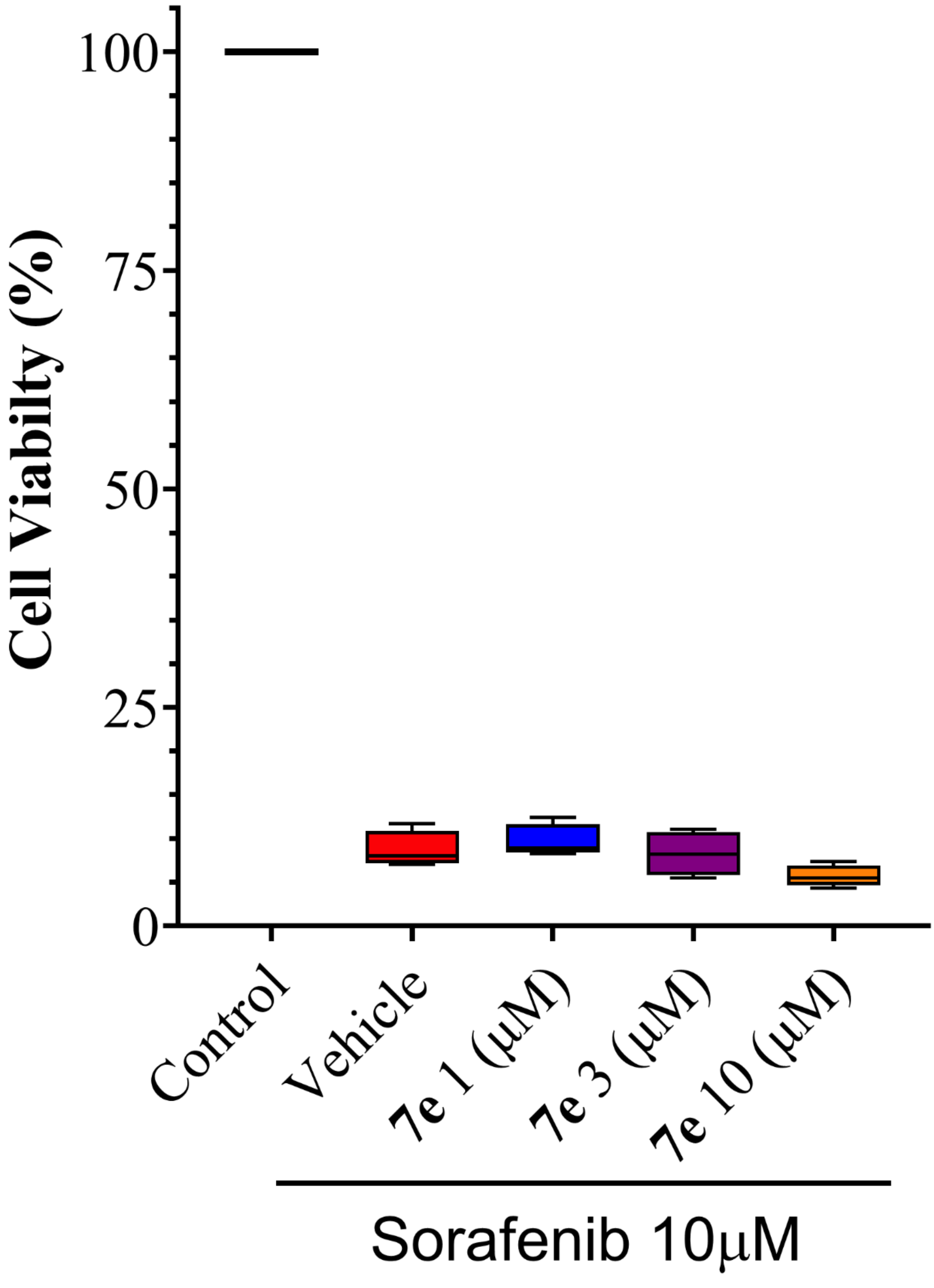

Figure S-108 WST-8 assay of human renal mesangial cells treated with sorafenib and 8,9-EET mimics at indicated concentrations after 48 h incubation, as percentages relative to control. Bars represent mean ± SEM (n = 4).

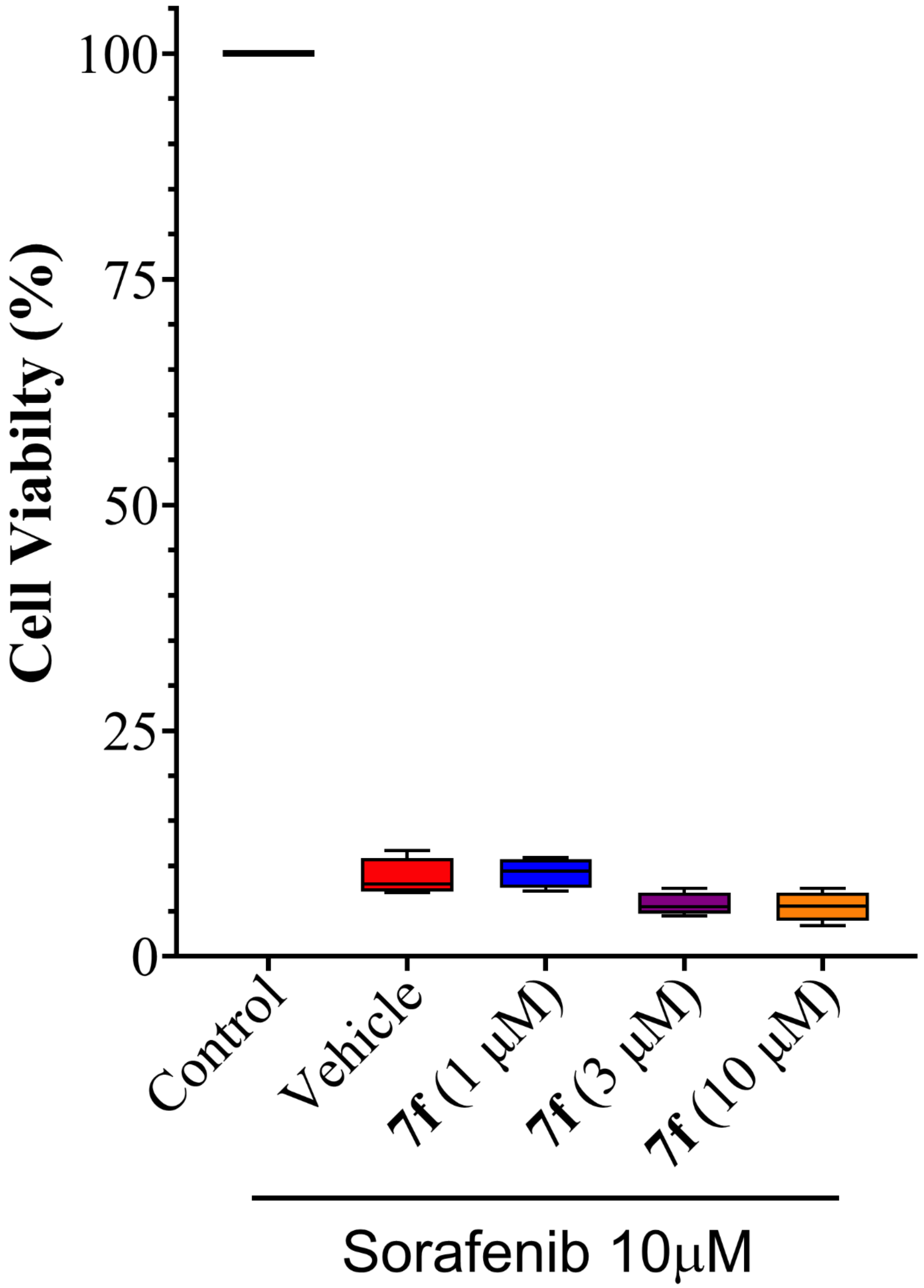

Figure S-109 WST-8 assay of human renal mesangial cells treated with sorafenib and 8,9-EET mimics at indicated concentrations after 48 h incubation, as percentages relative to control. Bars represent mean ± SEM (n = 4).

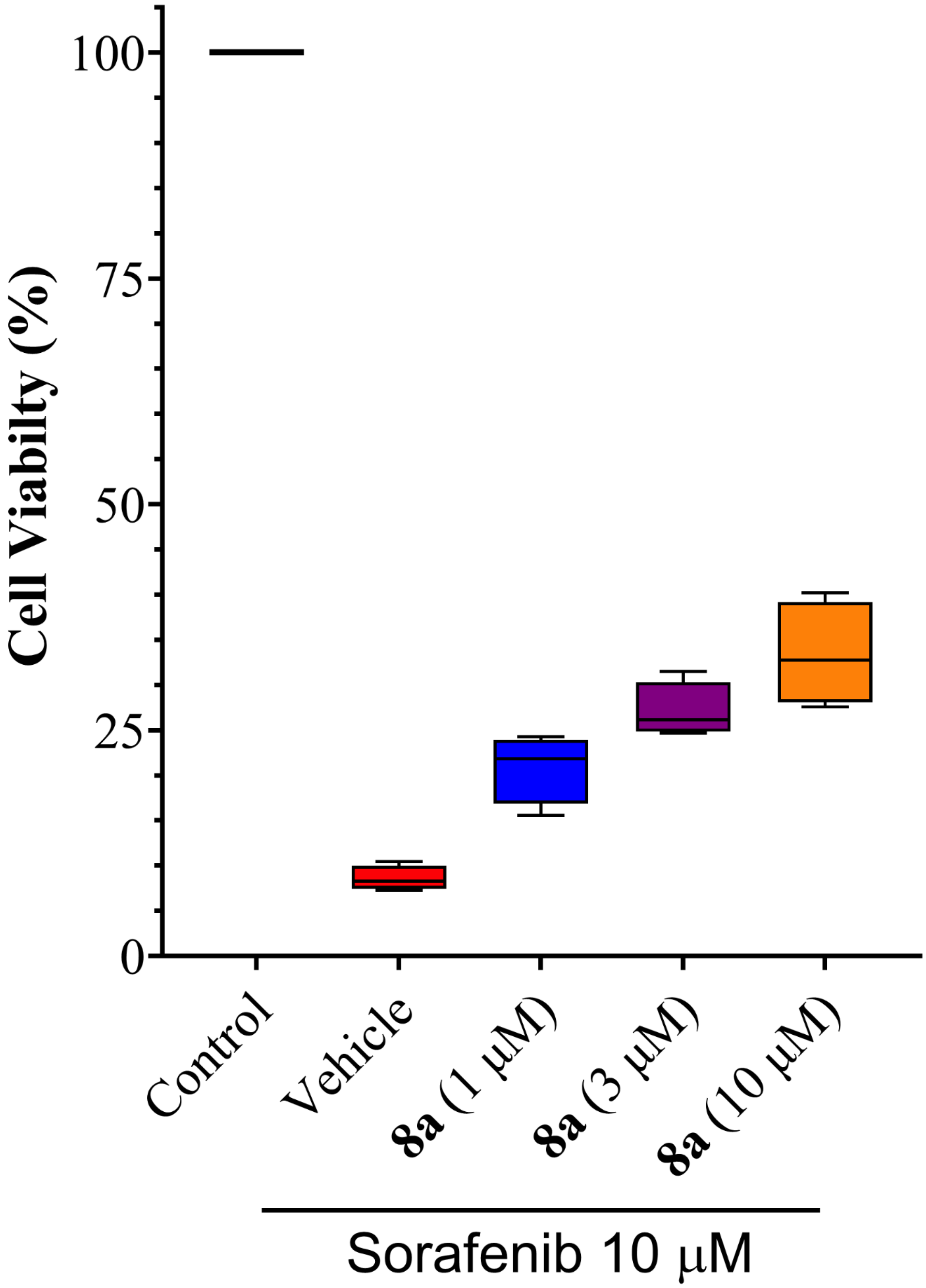

Figure S-110 WST-8 assay of human renal mesangial cells treated with sorafenib and 8,9-EET mimics at indicated concentrations after 48 h incubation, as percentages relative to control. Bars represent mean ± SEM (n = 4).

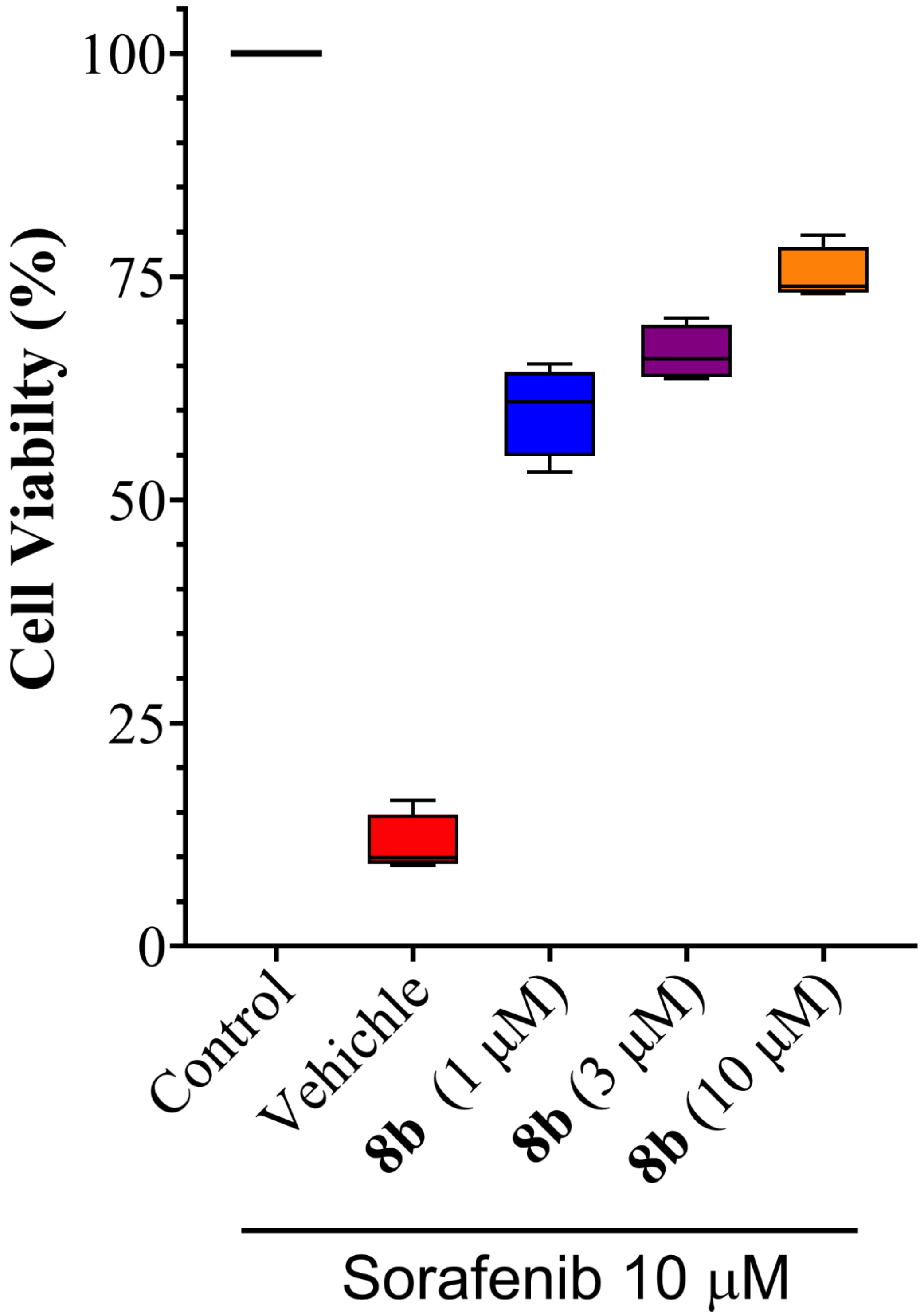

Figure S-111 WST-8 assay of human renal mesangial cells treated with sorafenib and 8,9-EET mimics at indicated concentrations after 48 h incubation, as percentages relative to control. Bars represent mean ± SEM (n = 4).

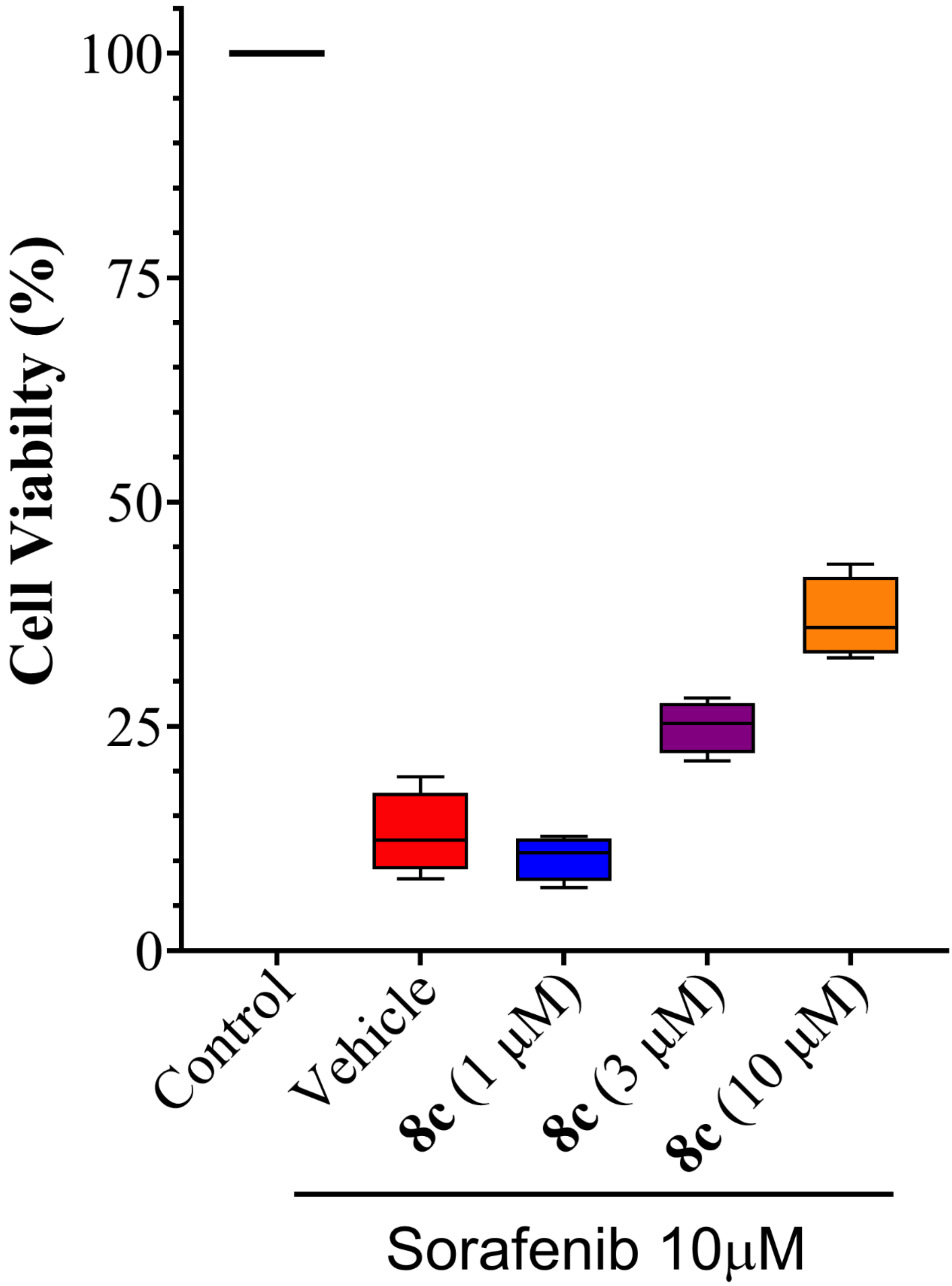

Figure S-112 WST-8 assay of human renal mesangial cells treated with sorafenib and 8,9-EET mimics at indicated concentrations after 48 h incubation, as percentages relative to control. Bars represent mean ± SEM (n = 4).

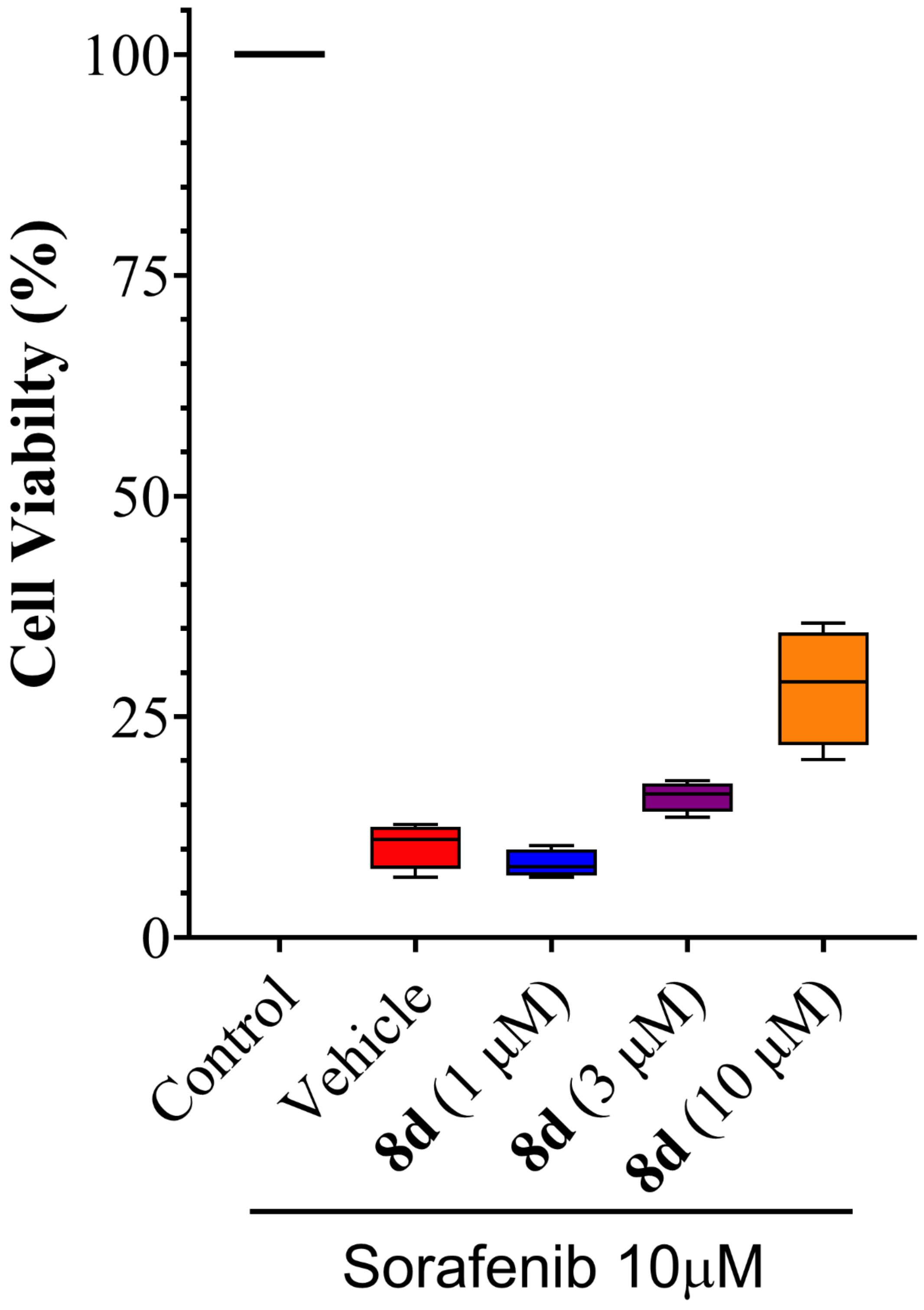

Figure S-113 WST-8 assay of human renal mesangial cells treated with sorafenib and 8,9-EET mimics at indicated concentrations after 48 h incubation, as percentages relative to control. Bars represent mean ± SEM (n = 4).

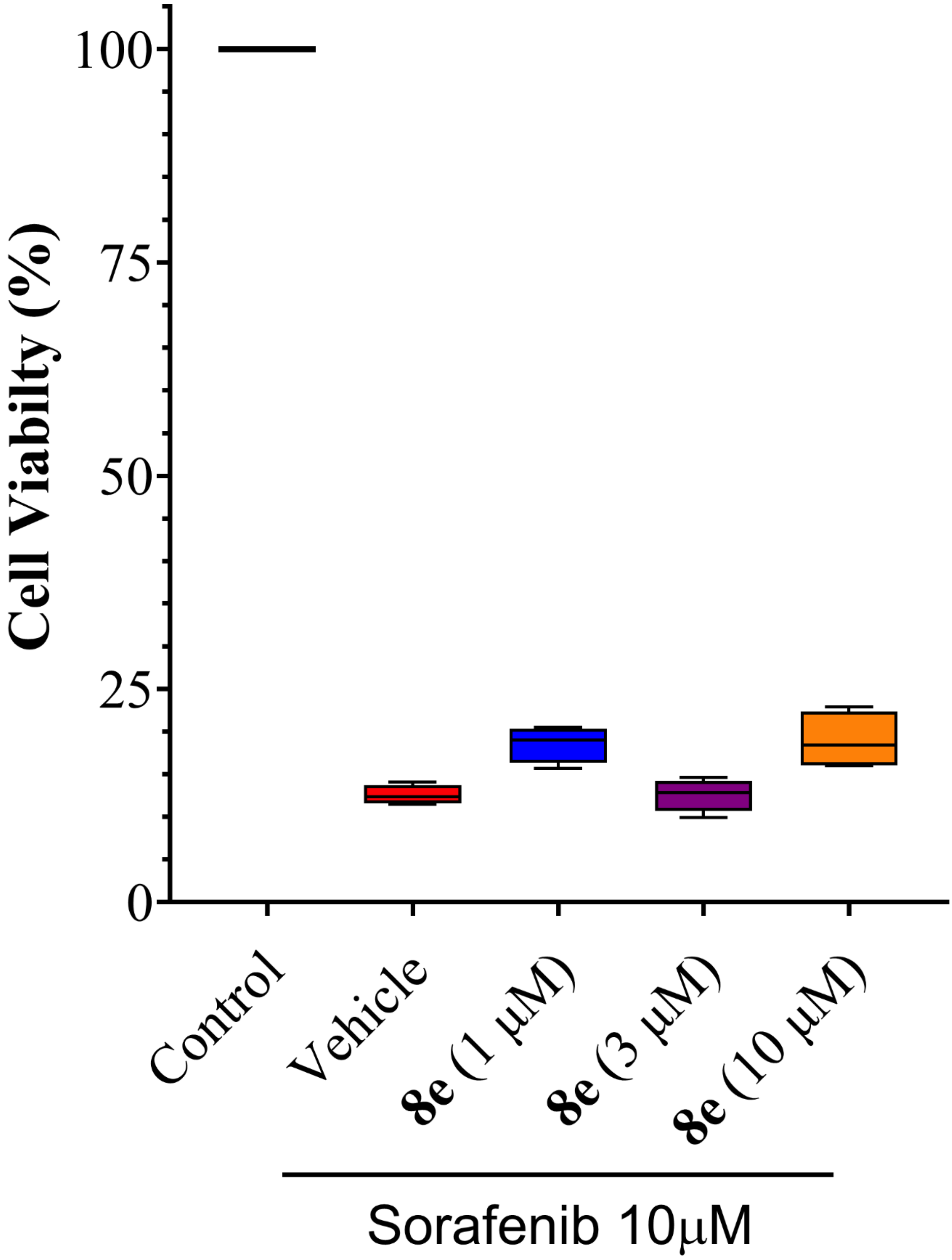

Figure S-114 WST-8 assay of human renal mesangial cells treated with sorafenib and 8,9-EET mimics at indicated concentrations after 48 h incubation, as percentages relative to control. Bars represent mean ± SEM (n = 4).

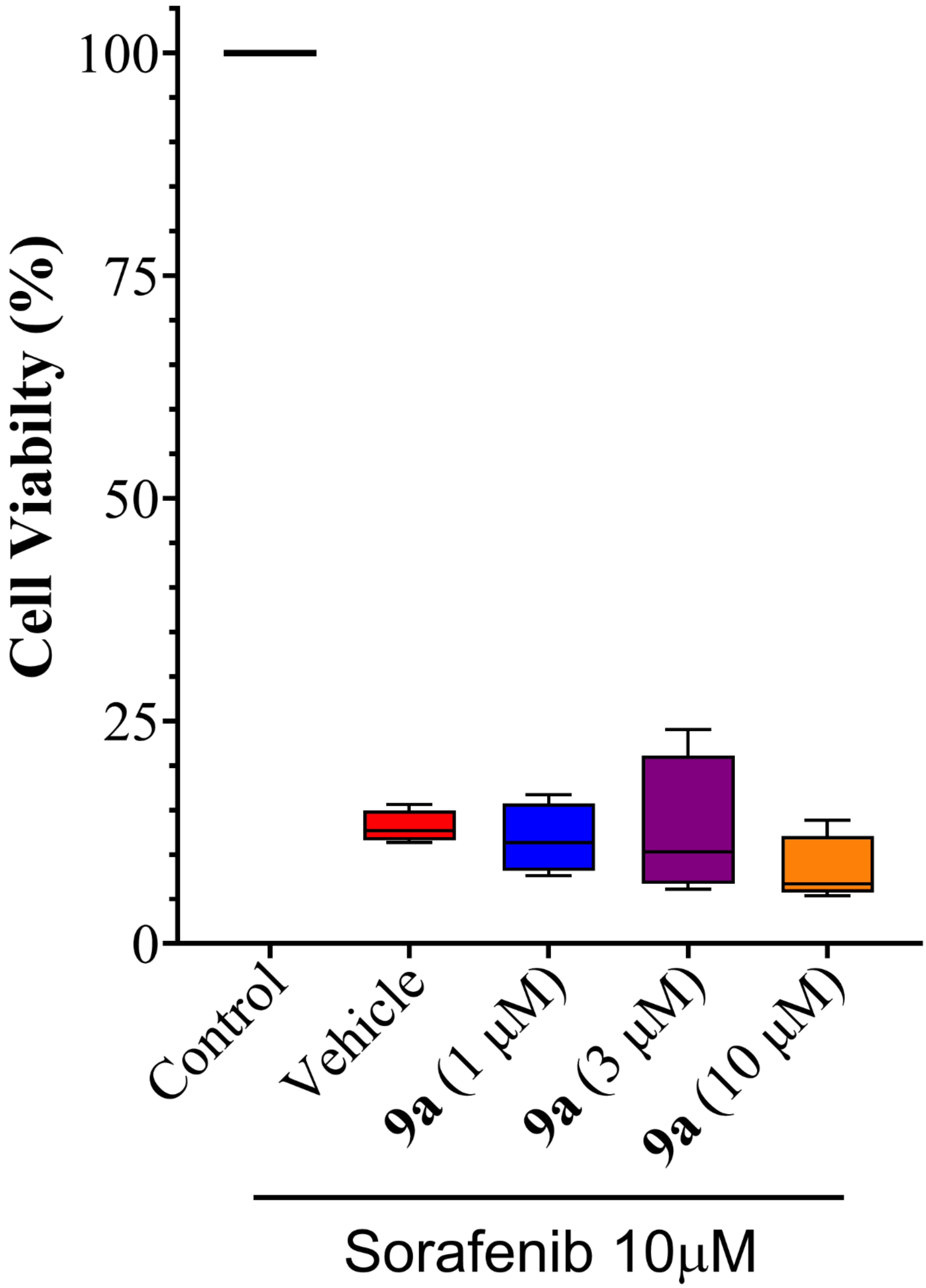

Figure S-115 WST-8 assay of human renal mesangial cells treated with sorafenib and 8,9-EET mimics at indicated concentrations after 48 h incubation, as percentages relative to control. Bars represent mean ± SEM (n = 4).

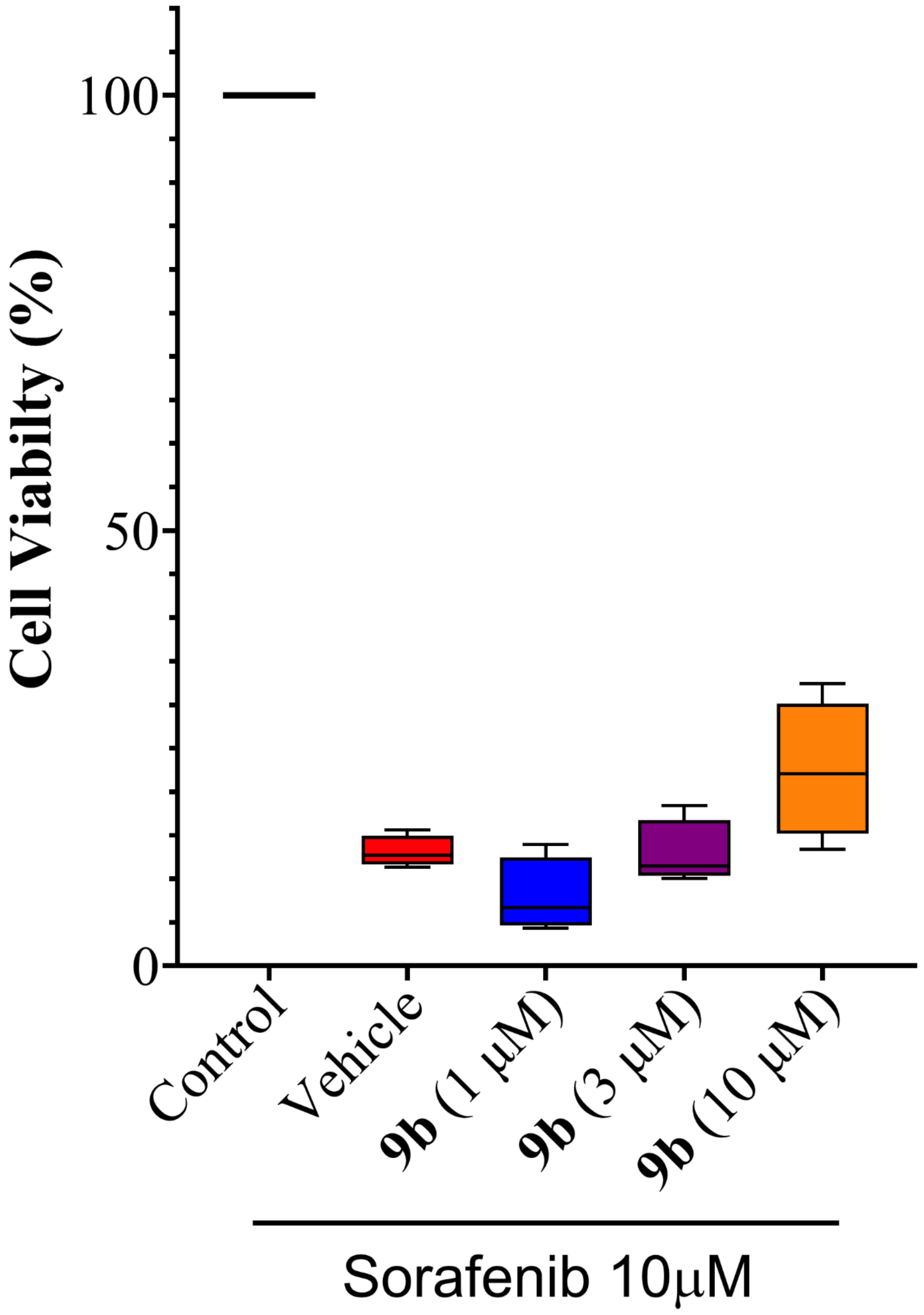

Figure S-116 WST-8 assay of human renal mesangial cells treated with sorafenib and 8,9-EET mimics at indicated concentrations after 48 h incubation, as percentages relative to control. Bars represent mean ± SEM (n = 4).

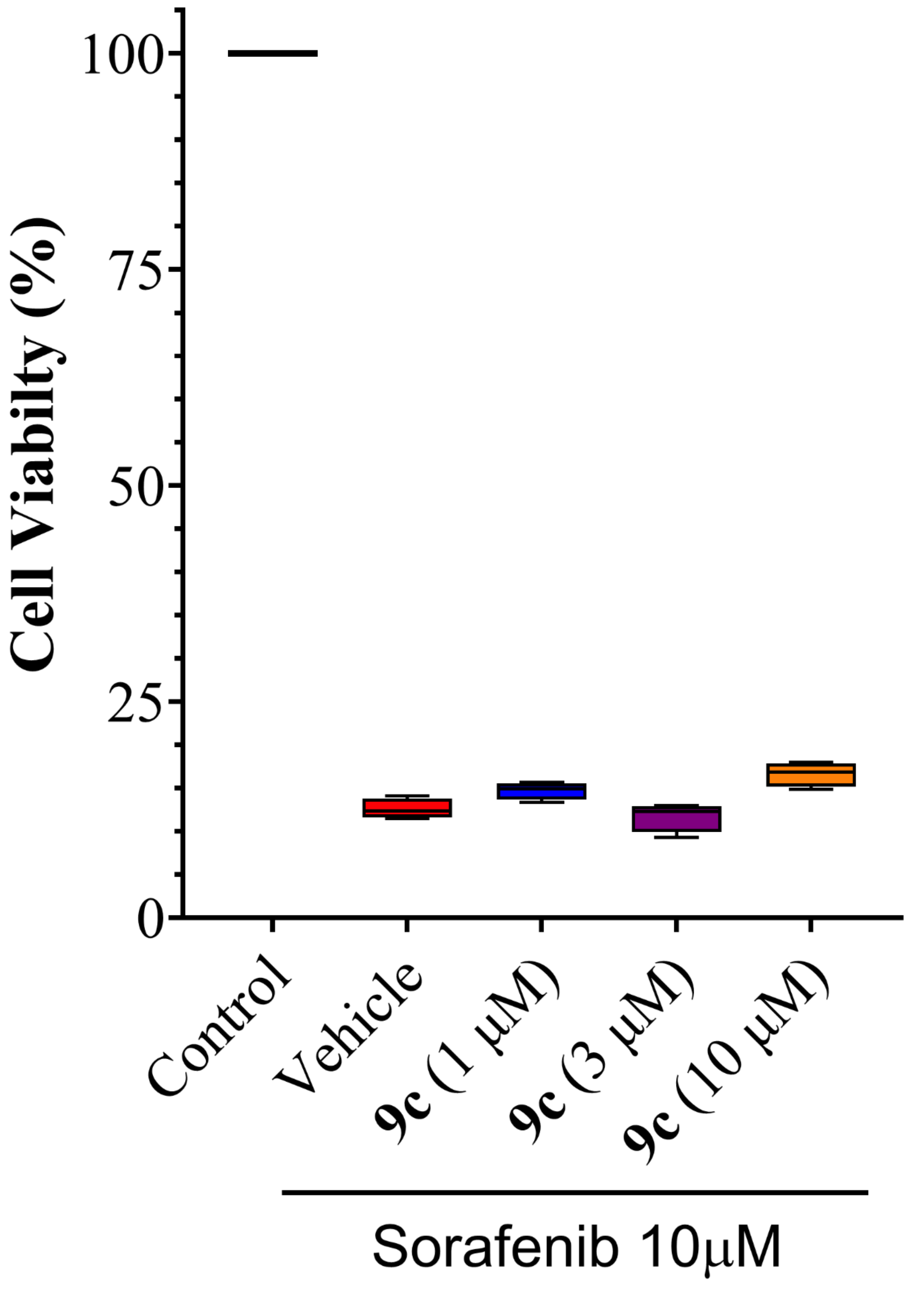

Figure S-117 WST-8 assay of human renal mesangial cells treated with sorafenib and 8,9-EET mimics at indicated concentrations after 48 h incubation, as percentages relative to control. Bars represent mean ± SEM (n = 4).

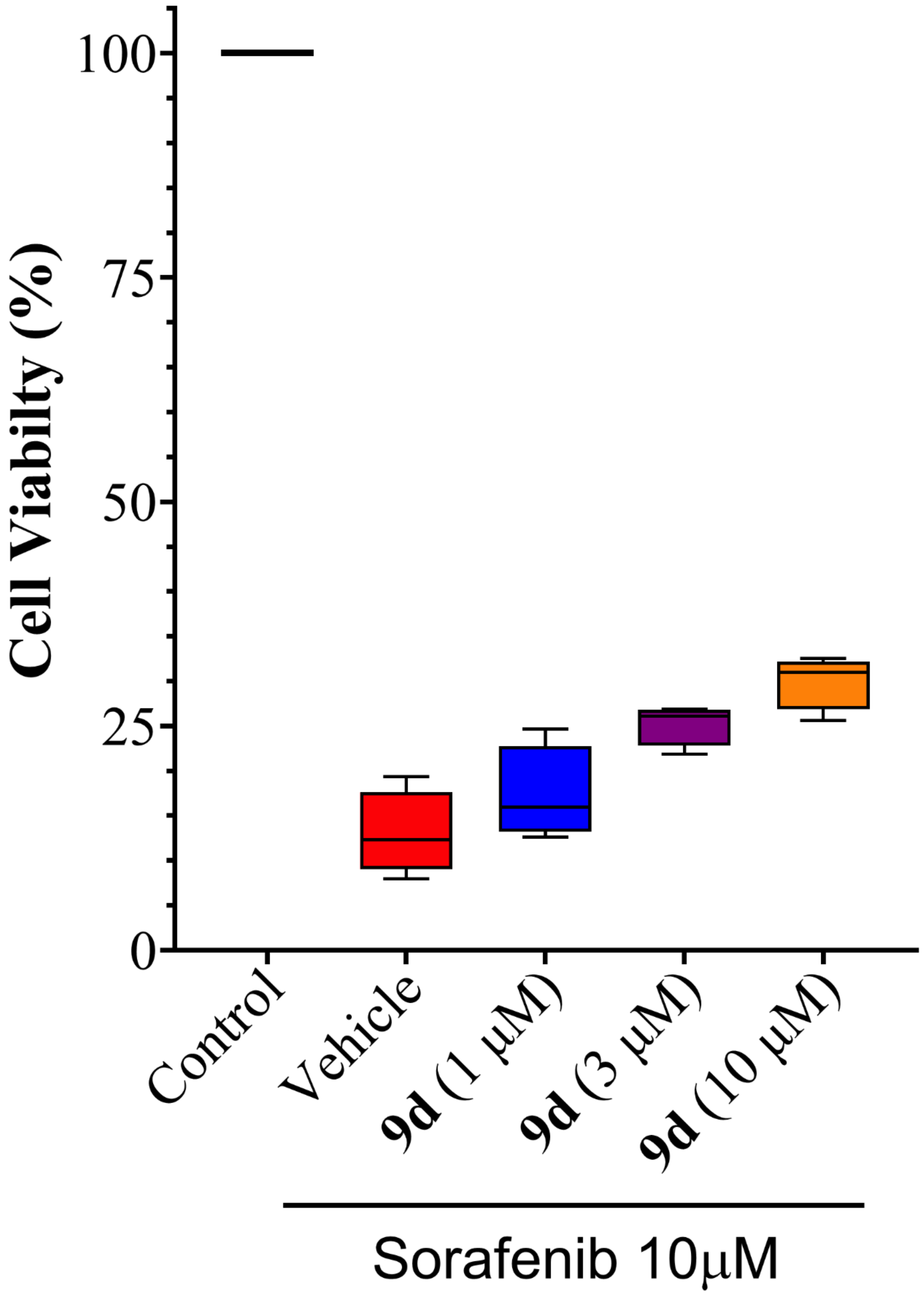

Figure S-118 WST-8 assay of human renal mesangial cells treated with sorafenib and 8,9-EET mimics at indicated concentrations after 48 h incubation, as percentages relative to control. Bars represent mean ± SEM (n = 4).

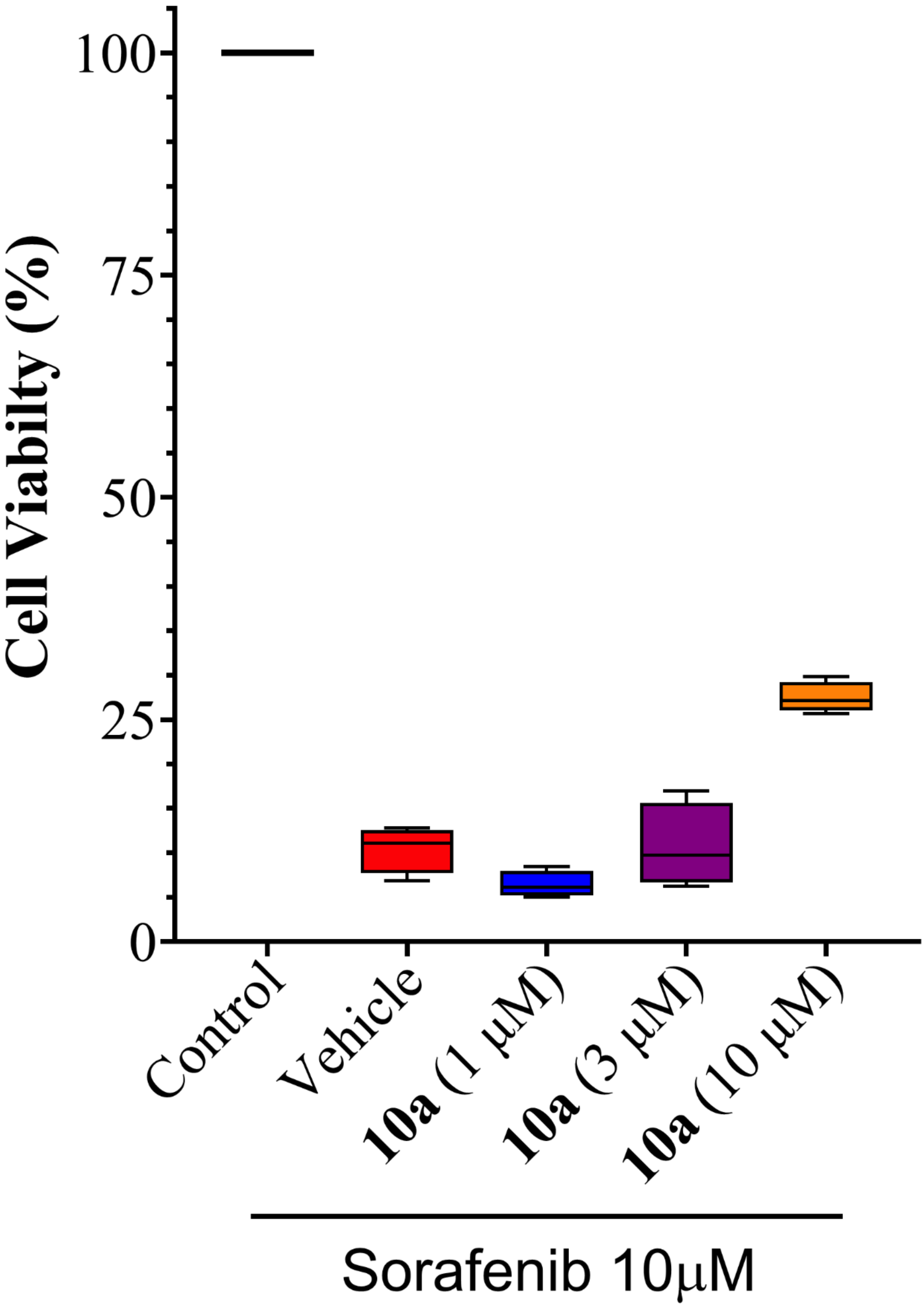

Figure S-119 WST-8 assay of human renal mesangial cells treated with sorafenib and 8,9-EET mimics at indicated concentrations after 48 h incubation, as percentages relative to control. Bars represent mean ± SEM (n = 4).

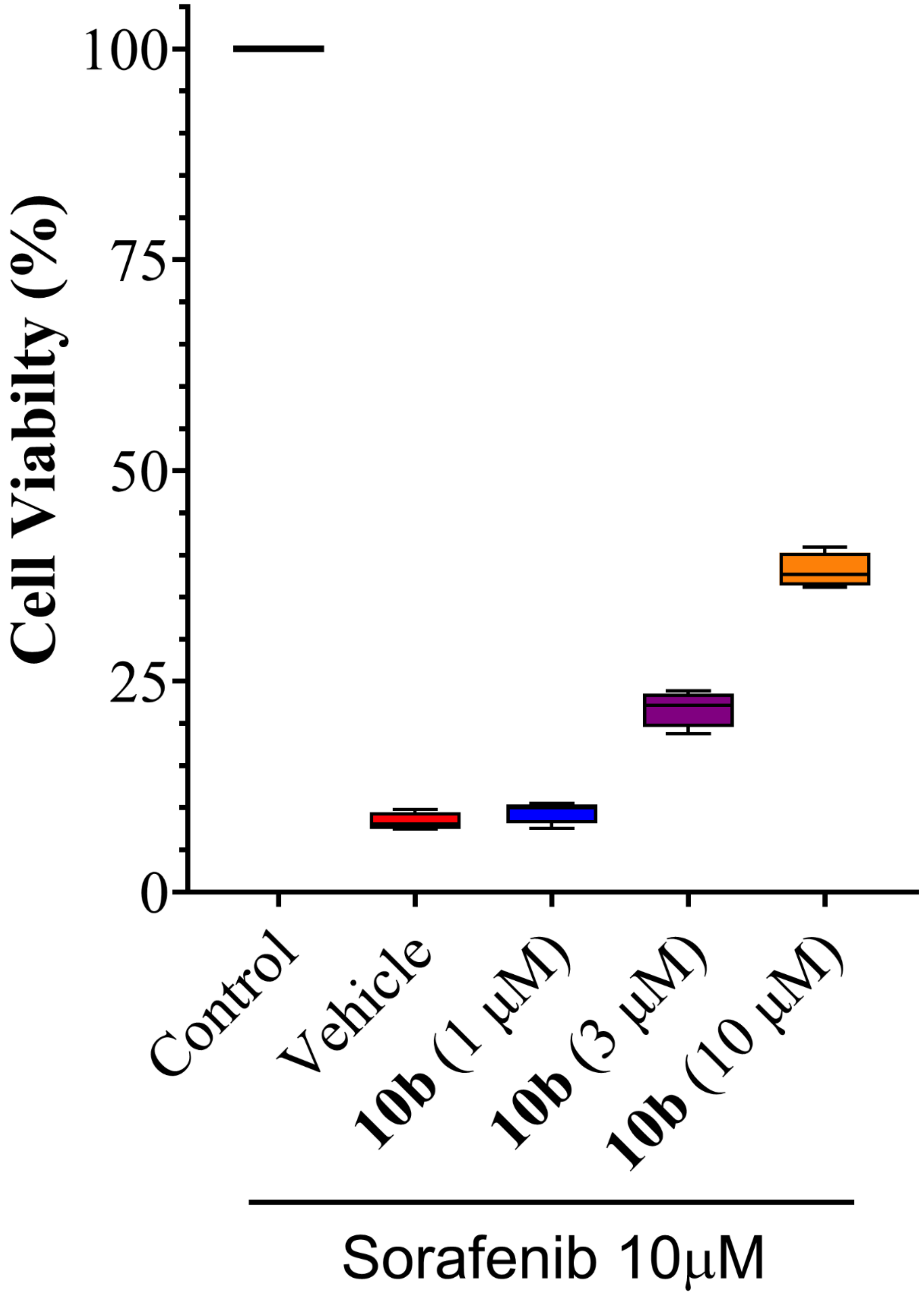

Figure S-120 WST-8 assay of human renal mesangial cells treated with sorafenib and 8,9-EET mimics at indicated concentrations after 48 h incubation, as percentages relative to control. Bars represent mean ± SEM (n = 4).

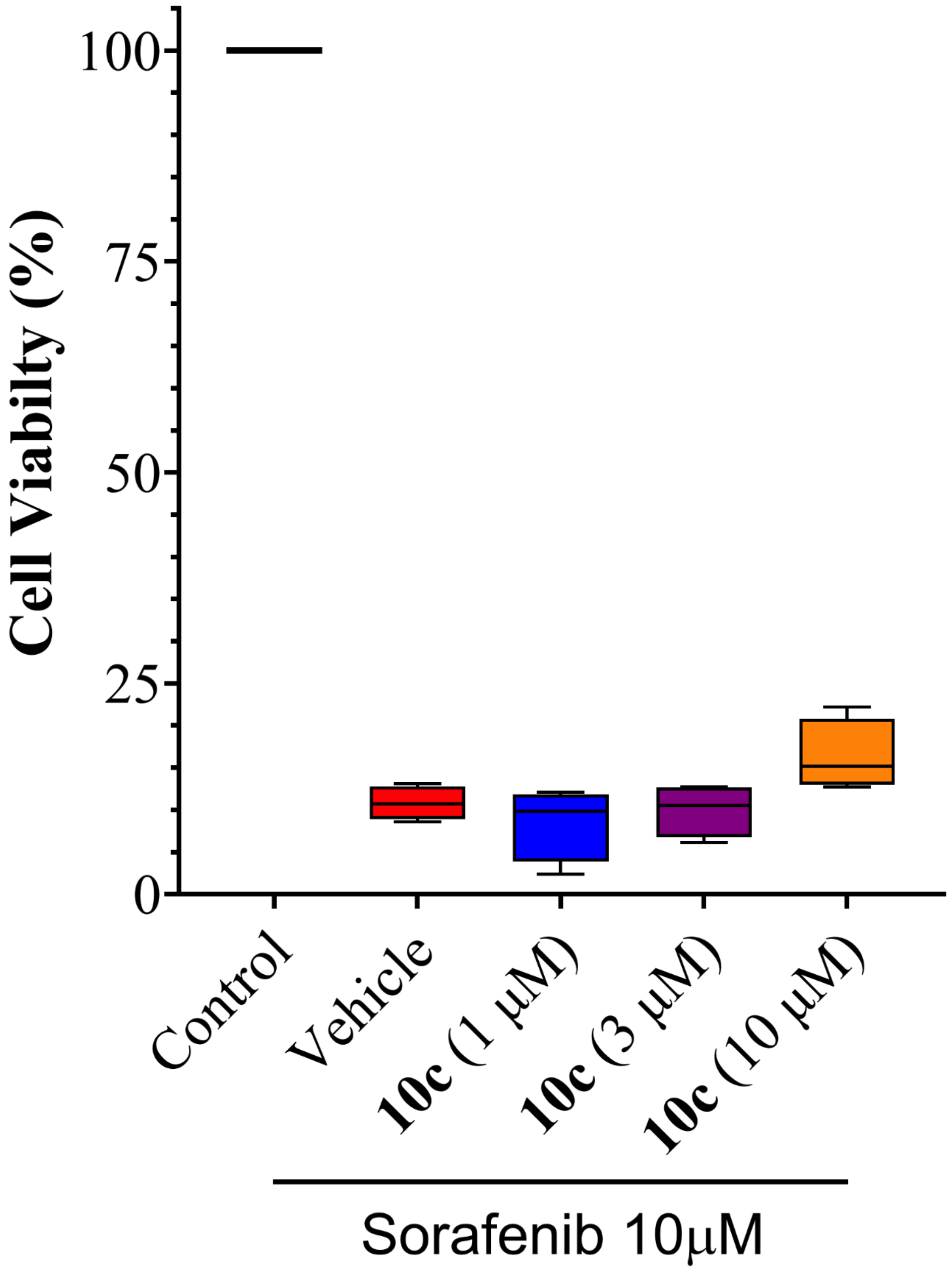

Figure S-121 WST-8 assay of human renal mesangial cells treated with sorafenib and 8,9-EET mimics at indicated concentrations after 48 h incubation, as percentages relative to control. Bars represent mean ± SEM (n = 4).
